# Lexicality effects on orthographic learning in beginning and advanced readers of Dutch: An eye-tracking study

**DOI:** 10.1177/17470218211047420

**Published:** 2021-09-28

**Authors:** Sietske van Viersen, Athanassios Protopapas, George K Georgiou, Rauno Parrila, Laoura Ziaka, Peter F de Jong

**Affiliations:** 1 Department of Special Needs Education, University of Oslo, Oslo, Norway; 2 Research Institute of Child Development and Education, University of Amsterdam, Amsterdam, The Netherlands; 3 Department of Educational Psychology, University of Alberta, Edmonton, Alberta, Canada; 4 Department of Educational Studies, Macquarie University, Sydney, NSW, Australia

**Keywords:** Eye tracking, lexicality, literacy development, orthographic learning, reading fluency

## Abstract

Orthographic learning is the topic of many recent studies about reading, but much is still unknown about conditions that affect orthographic learning and their influence on reading fluency development over time. This study investigated lexicality effects on orthographic learning in beginning and relatively advanced readers of Dutch. Eye movements of 131 children in Grades 2 and 5 were monitored during an orthographic learning task. Children read sentences containing pseudowords or low-frequency real words that varied in number of exposures. We examined both offline learning outcomes (i.e., orthographic choice and spelling dictation) of target items and online gaze durations on target words. The results showed general effects of exposure, lexicality, and reading-skill level. Also, a two-way interaction was found between the number of exposures and lexicality when detailed orthographic representations were required, consistent with a larger overall effect of exposure on learning the spellings of pseudowords. Moreover, lexicality and reading-skill level were found to affect the learning rate across exposures based on a decrease in gaze durations, indicating a larger learning effect for pseudowords in Grade 5 children. Yet, further interactions between exposure and reading-skill level were not present, indicating largely similar learning curves for beginning and advanced readers. We concluded that the reading system of more advanced readers may cope somewhat better with words varying in lexicality, but is not more efficient than that of beginning readers in building up orthographic knowledge of specific words across repeated exposures.

Orthographic learning is one of the main mechanisms underlying literacy development (Apel, 2009; Bowey & Muller, 2005; Castles & Nation, 2010; Share, 2008). It is defined as the acquisition of knowledge about sequences of graphemes representing spoken words (i.e., word-specific orthographic representations) and orthographic patterns that guide how units of speech, parts of spoken words, are generally represented in writing (i.e., language-specific orthographic pattern knowledge; Apel, 2011). Both types of orthographic knowledge are essential for literacy, but acquiring the former type, that is, detailed word-specific orthographic representations, is particularly crucial for developing reading fluency (Apel, 2009; Share, 2008). Although orthographic learning has been the focus of several studies (see Nation & Castles, 2017, for an overview), much is still unknown about the conditions which affect orthographic learning. In addition, relatively little attention has been paid to developmental changes in orthographic learning as children’s reading processes become more efficient and reading more fluent.

In this study, we assessed person- and word-level factors relevant for orthographic learning in a systematic way in a standard orthographic learning paradigm with beginning and more advanced readers. We combined offline learning outcomes and online reading measures obtained through eye tracking to reveal changes in reading processes during orthographic learning.

## Orthographic learning

According to the self-teaching hypothesis (Share, 1995), orthographic representations of specific words are built when the written forms of words are mapped onto their spoken forms through phonological decoding (Ehri, 1995; Share, 2008). Through successful activation of written word forms and their phonological representations, orthographic representations can be acquired, which, in turn, enables increasing word reading fluency during future encounters (Ehri, 2014; Share, 2008). In the standard orthographic learning paradigm, children generally read novel words (i.e., decodable pseudowords) embedded in sentences or text across multiple exposures. The quality of newly formed orthographic representations, the outcome of orthographic learning, is then evaluated with orthographic choice or spelling dictation tasks (Nation & Castles, 2017).

There are multiple factors that can facilitate orthographic learning (see Nation & Castles, 2017, for an overview). Exposure to print is well established as a factor contributing to orthographic processing ability and becoming a fluent reader, as it shows strong links with high word recognition skills (e.g., Chateau & Jared, 2000; Cunningham & Stanovich, 1990; see also Mol & Bus, 2011). In addition, several studies have confirmed the importance of explicit phonological recoding for orthographic learning (de Jong et al., 2009; Kyte & Johnson, 2006; Share, 1999), although orthographic learning can also occur without explicit reading (e.g., Protopapas et al., 2017). The availability of word meaning, however, does not seem to play a role in orthographic learning, as studies have shown that it does not lead to the formation of more detailed orthographic representations (e.g., Hogaboam & Perfetti, 1978; Share, 2004, but see Ouellette & Fraser, 2009). Studies of context as a facilitating factor have likewise found no effects (e.g., Cunningham, 2006; Nation et al., 2007), although selective facilitating effects only for irregular words have been found (Wang et al., 2011) and even negative effects in general (Landi et al., 2006), suggesting that the role of context in orthographic learning may be more nuanced.

Recently, the focus of orthographic learning studies has shifted towards facilitation through available phonological representations of specific words that are already present in the child’s mental lexicon. As beginning readers often learn to read words that are already part of their spoken vocabulary (Chalmers & Burt, 2008), a more reasonable approach to orthographic learning may be to assess known words instead of novel words. According to the orthographic skeleton hypothesis (Stuart & Coltheart, 1988), a child that is familiar with the spoken form of a word and has adequate letter-sound knowledge will also have an initial orthographic representation of the word. This representation is then used as a starting point for orthographic learning, meaning that an existing phonological representation could get orthographic learning started even *before* the child has encountered the word in print (Wegener et al., 2018; see also McKague et al., 2008). Recent findings in adults have provided further evidence that initial orthographic representations of embedded stems are formed during spoken word learning of morphologically complex words (Beyersmann et al., 2021). These findings are expected to generalise to developing readers as well (e.g., Beyersmann et al., 2019; Bowers et al., 2010; Grainger & Beyersmann, 2017).

This shift in research focus illustrates the need to systematically tease apart word-level and person-level factors that may influence orthographic learning and also requires more sensitive tasks and measures to assess reading processes during orthographic learning. Eye-tracking studies allow for more ecologically valid designs and sensitive measures that help gain deeper insights into how online reading processes may change during orthographic learning (Rayner, 1998, 2009). Whereas offline learning outcomes mainly provide more insight into long-term representation learning, as spelling dictation or orthographic choice tasks are often administered after a retention period of multiple days, eye-tracking measures mainly provide information about short-term activation of orthographic word forms and changes therein as the number of encounters with a word increases. Combining online and offline measures of orthographic learning can reveal more about actual learning processes involved in going from transient activation of word forms to consolidated orthographic knowledge (see, for example, Protopapas & Kapnoula, 2016, for an overview and discussion on assessing and combining short-term and long-term effects in visual word recognition). Therefore, in the following, we provide an overview of what studies with offline measures have taught us so far, grouped by conditions that are relevant in our study (i.e., number of exposures, lexicality, and reading-skill level), and consider the benefit of eye-tracking studies using online measures for further refining our current understanding of orthographic learning.

## Exposures

Studies using offline learning outcomes have shown that orthographic learning occurs upon exposure to written words almost without exception (Nation & Castles, 2017; but see Share, 2004; Share & Shalev, 2004, for findings on pointed Hebrew). However, less is known about how fast children form orthographic representations in terms of the required number of exposures. In most studies, the number of exposures is either equal across targets or the minimum number is too high to determine how many exposures are needed for learning to occur (Cunningham, 2006; Cunningham et al., 2002; Ehri & Saltmarsh, 1995; Wang et al., 2011, 2012), or even to discern between early learning (i.e., after one or two exposures) and late learning (i.e., after three or more exposures). Studies that did include a range of exposures indicate that orthographic learning might occur after just one exposure (Nation et al., 2007; Share, 2004) and generally point towards early learning once basic reading skills are in place (de Jong & Share, 2007; Share & Shalev, 2004; but see Reitsma, 1983, using naming as an outcome). In addition, most studies indicate no further learning with additional exposures after orthographic learning has taken place (de Jong & Share, 2007; Share, 1999), except in readers of English (Nation et al., 2007; and see de Jong et al., 2009, for further learning between three and six exposures).

Eye-tracking studies have the potential to provide more detailed information about orthographic learning from exposure to exposure. Studies of word learning in adults and children have already shown general learning effects as indicated by decreasing viewing times on words with increased exposure (e.g., Blythe et al., 2012; Joseph & Nation, 2018; Liang et al., 2015, 2017; Pagán & Nation, 2019). Several studies with more detailed exposure data on adults have shown that viewing times on targets start to decrease immediately after the first exposure. In addition, viewing times level out after about 5–6 exposures (Kamienkowski et al., 2018; Parrila & Barber, 2011; see also Hung et al., 2013; Joseph et al., 2014; Parrila & Turgeon, 2012), indicating that words should be read at least four times before their orthographic representation is sufficiently detailed to facilitate instant word recognition within one experimental session. These findings have recently been replicated in children (i.e., third grade; Liang et al., 2021). However, studies with such a detailed experimental set-up that allows for monitoring of continuous learning over repeated exposures are rare, and there are, to our knowledge, currently no eye-tracking studies modelling the time course of orthographic learning across exposures in children during different stages of reading development.

## Lexicality

Besides the knowledge gap about the required numbers of exposures, we also lack systematic knowledge about how the time course of forming orthographic representations is influenced by the child’s familiarity with a word (Apel, 2009). Multiple studies have provided evidence for orthographic learning of novel words (e.g., Share, 1999; Share & Shalev, 2004) as well as known words (e.g., Cunningham, 2006; Tamura et al., 2017). We also know that orthographic learning of novel words may even be evident after as few as one exposure (Nation et al., 2007; Share, 2004). However, such an early learning effect has not yet been observed for known words (Reitsma, 1983, 1989), although this would be expected based on the pre-existing phonological representations (Stuart & Coltheart, 1988). Only one study has directly compared orthographic learning of novel and (orally) known words in children, finding no facilitation for words already in the child’s mental lexicon (Share, 2004).

Eye-tracking studies assessing the influence of lexicality on orthographic learning have revealed a novel-word effect in adults, with longer viewing times for novel words than for known words (Brusnighan et al., 2014; Chaffin et al., 2001; Lowell & Morris, 2014; Williams & Morris, 2004; Wochna & Juhasz, 2013). In addition, studies with adults have also documented a word-frequency effect, with longer viewing times for infrequent words than for frequent words (Kamienkowski et al., 2018; see also Rayner, 1998, for a review). This word-frequency effect has also been found in children (Hyönä & Olson, 1995; Joseph et al., 2013; Rau et al., 2014, 2015; Tiffin-Richards & Schroeder, 2015).

To date, only Wegener et al. (2018) have directly compared orthographic learning of trained and untrained novel words in children using eye tracking, aiming to assess the influence of available phonological representations. They manipulated word familiarity by training children on spoken vocabulary for one set of novel words and providing no training for another set. They found shorter viewing times for trained words, indicating a different starting point for orthographic learning of words with an available phonological (and possibly semantic) representation in the child’s mental lexicon. However, it remains unclear how this processing benefit may vary across exposures or across reading-skill levels and how it may affect online reading processes.

## Reading-skill level

Despite a growing research base in recent years, knowledge about developmental changes in orthographic learning across literacy development is still limited (Apel, 2009, 2011). Experimental studies on orthographic learning in children have typically examined a single grade level. Thus, beginning (i.e., Grades 1 and 2) and more advanced readers (i.e., Grade 4 and up) are not directly compared within the same experiment, although one might expect that having more orthographic knowledge would facilitate further orthographic learning. Taken together, studies with repeated exposures can provide some insight into developmental differences in terms of early versus late learning effects. Indeed, findings suggest more late learning (i.e., requiring three or more exposures) in beginning readers (Reitsma, 1983, 1989), compared to more early learning (i.e., within one or two exposures) in advanced readers (de Jong & Share, 2007; Nation et al., 2007; Share, 2004; Share & Shalev, 2004). These effects seem to be consistent across orthographies (but see Share, 2004; Share & Shalev, 2004, on beginning readers of pointed Hebrew), but could turn out differently when lexicality is also considered.

Eye-tracking studies have consistently shown that eye movements during reading change with increasing reading experience (Blythe & Joseph, 2011; Reichle et al., 2013; Schroeder et al., 2015). In general, viewing times on words as well as reading times on sentences decrease as children become better readers (Blythe et al., 2009; Buswell, 1922; McConkie et al., 1991; Vorstius et al., 2014; see Rayner, 1998, for a detailed overview of early findings; see Liang et al., 2021, for differences between children and adults on novel words). Some studies on word processing using online measures have shown that lexicality effects differ based on reading skill (e.g., Huestegge et al., 2009; Joseph et al., 2013; see Blythe et al., 2009, for differences between children and adults). Rau et al. (2014), for example, found comparable viewing times across familiar and unfamiliar words in beginning readers (Grade 2), suggesting the application of serial decoding irrespective of lexicality, and increasing viewing times with decreasing word familiarity in more advanced readers (Grades 3 and 4), suggesting dominance of reading by direct recognition of words in experienced readers. The study by Wegener et al. (2018) illustrates how using advanced methods to assess specifically orthographic learning can inform knowledge about literacy development in children. To better understand how children become fluent readers over time, we need more such studies, with special attention paid to differences in online reading processes from a developmental perspective.

## The present study

In this study, we systematically examined the influence of exposure (i.e., number of repetitions) and lexicality (i.e., pseudowords vs. low-frequency words) on orthographic learning across two levels of reading skill (i.e., Grades 2 and 5). Standard offline learning outcomes from traditional studies of orthographic learning are validated and extended using online reading measures from eye-tracking studies, in an attempt to bridge the gap between different approaches and illustrate the benefit of modelling learning in a continuous way.

This study is conducted with children learning to read in Dutch. The Dutch language has a complex syllable structure, but its orthography can be categorised as semi-transparent (Seymour et al., 2003). The majority of words can be read using the dominant grapheme-phoneme correspondences. However, the orthography is less consistent in terms of spelling (Bosman et al., 2006). Especially vowels (e.g., a, o, e, u, i, y, ie) and vowel diphthongs (e.g., ou, au, ei, ij) can be written in multiple ways, but irregularities can occur in other cases as well, for example, final devoicing of plosives and fricatives (e.g., b/p, d/t, f/v, s/z), silent “h,” and *schwa* (e.g., de Bree et al., 2017; Patel et al., 2004). These inconsistencies have to be learned to establish correct and detailed orthographic representations of specific words. As such, Dutch is a suitable starting point for investigating orthographic learning in a semi-transparent orthography. As with other semi-transparent orthographies, Dutch children typically attain high reading accuracy by the end of Grade 2 (van Viersen et al., 2018), after which reading fluency rapidly increases. Accordingly, the Grade 2 children in this study can be considered beginning readers and the Grade 5 children relatively advanced readers.

### Hypotheses

As this is the first study to take such a systematic approach, combining three separate conditions and offline as well as online outcomes, and due to the scarcity of studies on orthographic learning in children using eye tracking, parts of the study are necessarily exploratory in nature. However, several hypotheses can be formulated regarding the specific conditions included in the present study based on previous findings from standard orthographic learning paradigms as well as recent eye-tracking literature.

For lexicality, we hypothesised shorter viewing times on known words than novel words, translating into early learning (i.e., after one or two exposures) of known words on offline outcomes, possibly due to the availability of phonological representations, for both beginning and more advanced readers. Likewise, we hypothesised longer viewing times on novel words than known words, translating into late learning (i.e., after three or more exposures) of novel words on offline outcomes for beginning readers, and possibly protracted learning (i.e., starting early, but continuing across multiple exposures) for more advanced readers. In addition, we explored how fast viewing times would decrease across exposures (providing an indication of the learning rate based on short-term activation), and possible lexicality effects therein. In general, the effect of lexicality and interactions with exposure are expected to be stronger for offline learning outcomes on spelling dictation than orthographic choice tasks, as providing the correct spelling requires more detailed orthographic representations to be formed than just recognising the correct irregularity.

For reading skill, there are two possibilities; despite possible differences regarding lexicality, (1) the reading system of beginning readers in Grade 2 could be equally effective in the building-up of orthographic representations, or (2) orthographic representations could be more rapidly acquired in the better developed reading system of more advanced readers in Grade 5. Both options are equally plausible, as there is no literature available regarding the influence of literacy development on orthographic learning.

As a general note, although our hypotheses on lexicality and reading skill are formulated equally strongly for online and offline measures, it is likely that the transient effect of priming of orthographic word forms only partially leads to permanent effects in terms of learning. As such, it is likely that not all findings are reflected in both the online and offline outcomes of orthographic learning, but clear contradictions are not to be expected.

## Methods

### Participants

A total of 131 Dutch primary school children from Grades 2 (*n* = 75, 49.3% girls, *M*_age_ = 92.8 months, *SD*_age_ = 4.5 months) and 5 (*n* = 56, 60.7% girls, *M*_age_ = 128.9 months, *SD*_age_ = 4.4 months) were included in this study. They were recruited from four different public schools that participated. Two schools were located in a large urban area in the west of the Netherlands and two schools were located in a smaller town in the central part of the country. Ethical approval for the study was provided by the Ethics Committee of the University of Amsterdam (case no. 2017-CDE-8332). Initially, parents of 147 children were notified of the school’s participation in the study. Six children were excluded from the study before it started because parents objected to their child’s participation. One child withdrew its consent during the study and was removed from the sample. Data were collected from children of all reading levels, but nine children were excluded after data screening because their reading scores were considered outliers (see section “Data screening”).

### Exposure phase

A computerised orthographic learning task was administered, programmed in Experiment Builder version 2.1.40 (SR Research, 2016), in which children were exposed to target items during a natural sentence reading task. The task had a Minions theme to engage the children. Children were instructed to look for the minion to the middle-left of the screen (i.e., disguised calibration point for eye-tracking, see below), read the sentence aloud, search for the minion at the bottom right of the screen when done reading, and use the mouse to answer questions (i.e., yes/no response boxes) that might appear after some of the sentences (see below). Sentences were displayed centred, at the middle of the screen and always fit on a single line, with margins on both sides. Progression to the next sentence was experimenter-triggered. Children received three practice trials, one of which had a question afterwards, followed by 80 experimental trials divided into blocks of 20 sentences.

### Materials

#### Targets

Targets were decodable low-frequency mono- and bisyllabic words and matched pseudowords with irregular spellings. Irregularities could involve vowels (i.e., y/ie/i), vowel digraphs (i.e., ou/au, ei/ij), voiced and voiceless stops (e.g., d/t), double consonants (i.e., cc/k), silent “h,” and *schwa*. Words were initially selected using Dutch norms for age of acquisition (Brysbaert et al., 2014). Children were expected to be familiar with the words’ meanings, but not yet with their spellings. Word frequencies were checked in the SUBTLEX-NL database (Keuleers et al., 2010). Half of the targets were tools and the other half animals, to maximise the range of usable verbs and adjectives to create the accompanying sentence templates (see below). Pseudowords were matched to the words in terms of number of letters and syllable structure where possible and were meant to represent fake tools and fake animals. Linguistic characteristics of the targets are displayed in Table 1.

**Table 1.** Word targets (16) with linguistic characteristics and pseudoword targets (16).

Target	Translation	Age of acquisition^a^	SUBTLEX freq. per million^b^	BasiLex freq. per million^c^	Letters (syllables)	Pseudoword
geit	goat	5.02	8.0724	28.1260	4 (1)	sijf
kauw	jackdaw	8.63	1.5093	1.3560	4 (1)	froun
lynx	lynx	11.35	0.7089	0.1569	4 (1)	kryn
griend^d^	pilot whale	14.71	0.0457	0.0071	6 (1)	peid
zeis	scythe	9.58	0.5717	0.8560	4 (1)	wijr
bout	bolt	9.84	0.9376	0.2140	4 (1)	raud
vijl	file	9.55	0.3202	0.8560	4 (1)	reil
klauw^e^	claw	7.12	3.2701	4.4970	5 (1)	souw
reiger	heron	9.04	0.1829	0.4997	6 (2)	bijfler
lijster	redwing	9.89	0.5031	1.4990	7 (2)	feistar
python	python	10.83	1.4407	3.3550	6 (2)	rylet
kievit	lapwing	10.97	0.1143	1.7130	6 (2)	wauchol
vijzel^f^	mortar	13.40	0.0457	0.0928	6 (2)	lijtal
accu^g^	battery	11.43	3.9561	6.9950	4 (2)	occa
beitel	chisel	9.26	0.7089	2.9260	6 (2)	fleiper
krauwel^h^	rake	–	–	–	7 (2)	spoukel

freq.: frequency

a Brysbaert et al. (2014).

b Keuleers et al. (2010).

c Tellings et al. (2015). The correlation between SUBTLEX and BasiLex frequencies per million is .94.

d Age of acquisition (AoA) expected to be lower for Dutch children, as a Griend is the only type of whale that washes up on Dutch beaches and it is often reported on the children’s news.

e Tool version of this word may have a higher AoA.

f Compound words including “vijzel” with a related meaning have lower AoA.

g AoA is probably lower than in 2010 because of children’s familiarity with cell phones.

h No AoA or frequency available: children may not be familiar with this specific type of rake for clearing a pond, but children may know this word from other contexts (e.g., Dutch version of Harry Potter) in which it occurs more frequently.

An extended set of possible targets was piloted using a dictation task in two Grade 2 and two Grade 5 classrooms that were not part of the current sample. For the words, error analyses indicated the target items with which the children were least familiar in terms of their spelling. These were selected as experimental targets (e.g., klauw/klouw, vijzel/veizel). For the pseudowords, pairs were selected with an error ratio close to 50/50 for the targeted irregularities (e.g., reil/rijl, wauchol/wouchol). For each selected pair, the pseudoword with the less likely spelling was included as an experimental target and the more likely spelling was used as a homophone in the orthographic choice task (see below). Targets were allowed to have spelling irregularities in addition to those manipulated in the orthographic choice task, aiming to increase target complexity and maximise the need for orthographic learning. The final set of items used in the experiment contained 32 targets, including 16 words and 16 pseudowords (see Table 1).

#### Sentences

A set of 64 experimental sentences was constructed to incorporate the target items. Each sentence followed a fixed template around the position (*n*) where the target would be placed. The word before the target (*n* − 1) was always an adjective and the word after the target (*n* + 1) was always a meaningful verb. As such, targets were never placed at the beginning or end of the sentence and were instead placed towards the middle as much as possible (i.e., target position *M* = 4.69, *SD* = 1.64, Min = 3, Max = 9) to avoid fixation times being affected by target position (Kuperman et al., 2010). Sentence context was as neutral as possible. Half of the sentences were constructed to fit tool-like targets (e.g., *De rode ___ ligt ergens in huis verstopt* [The red ___ is hidden somewhere in the house]) and the other half fit animal-like targets (e.g., *Naar een jonge ___ kijken is altijd leuk* [Watching a young ___ is always fun]). Sentence semantics and structure were aligned with the level of complexity generally present in reading materials used in the middle grades of primary education. All initially constructed sentence frames were graded by a group of six educational researchers to evaluate the appropriateness of the required reading level and their suitability to fit the targets (i.e., scale 1–10). The 64 sentence templates with the highest scores were selected for the experiment (see Online Supplement A, Table S.1).

The experimental sentences were complemented by 16 filler sentences, following the experimental sentence template and containing a high-frequency word with regular spelling at the target position (e.g., *Het gevlekte kalf sprong vrolijk door de wei* [The spotted calf jumped happily through the meadow]). Filler sentences were followed by a simple comprehension question with a yes/no answer (e.g., *Was het kalf buiten?* [Was the calf outside?]). The filler sentences and questions were included to ensure that children were reading for meaning and not merely scanning the sentences.

#### Trials

Targets and experimental sentences were combined to form the trials in the orthographic learning task. Targets were fully counterbalanced across conditions (i.e., exposure and lexicality, and also taking into account word length), randomly paired with sentence templates, and shuffled into a random order using a Python script (version 2.7; van Rossum & Drake, 2002) to produce a pre-arranged trial list for each individual participant. Each participant thus received a unique combination of targets, covering four words (two tools and two animals) and four pseudowords—a total of eight target items to which the participant was exposed twice, and an identically structured set of eight items to which the participant was exposed six times. Each participant received the targets in unique target-sentence pairs, as all sentences were suitable for all targets in terms of semantics and structure (i.e., for tools and animals separately), and placement of targets in sentences was also randomised. The 16 target items to which the child was not exposed functioned as an untrained baseline for the offline outcomes of orthographic learning for this particular child. In this way, all 32 target items served as both untrained and trained items (for both two and six exposures) equally when considered across the entire sample of children.

### Screening measures

To obtain an objective measure of the children’s reading level, word-list reading fluency was measured using the *Een Minuut Test* (EMT; Brus & Voeten, 1999) and pseudoword-list reading fluency using the *Klepel* (van den Bos et al., 1994). Children had one (for words) and two (for pseudowords) minutes to read as many items as possible. Item difficulty increased from one to four syllables in both tests. The raw score is the number of correctly read items within the time limit, with a maximum of 116 on each test. Standard scores are available using grade-level norms per semester (*M* = 10, *SD* = 3). Test–retest reliability is .90 for EMT and .92 for Klepel (Evers et al., 2009–2012).

### Offline outcome measures

#### Orthographic choice

Recognition of correct spellings of target items from the orthographic learning task was measured with an orthographic choice task. All 32 target items and their homophone alternatives (i.e., differing in spelling at only one phoneme position) were presented to the child in a paper-and-pencil task. Target-homophone pairs were displayed in two columns (each row containing one target and the corresponding homophone in random left-right arrangement) and covered front and back of one A4 sheet. The task started with three practice trials in which the child had to recognise and underline the words with the correct spelling. Subsequently, the child had to do the same for the 32 target-homophone pairs as fast as possible. The raw score was the number of correctly recognised target items, which was used in the analyses. The same sheet was used for all children; assignment of particular items to trained (2 or 6 exposures) and untrained (0 exposures) conditions was determined individually after scoring.

#### Spelling

Production of correct spellings of target items from the orthographic learning task was measured with a spelling dictation task. All 32 target items were read aloud twice in isolation and children had to write down the correct spelling of the (pseudo)words. The raw score was the number of (completely) correctly produced target items. Item-condition assignment was individually determined after scoring.

### Eye tracking

Eye movements were recorded using an EyeLink 1000Plus eye tracker (SR Research; Mississauga, Canada), with a 25 mm lens and sampling rate of 500 Hz in “remote” mode, i.e., participants were free to move, within reasonable boundaries, without any form of head stabilisation. A target sticker was attached to the participant’s forehead to allow eye tracking in remote mode. Children read sentences from a 22″ Dell computer monitor (1,680 × 1,050 pixels) at a viewing distance of approximately 70 cm. Sentences were presented in black, Verdana Normal 20 font, on a white screen so that three characters of text would fall within 1° of visual angle. Viewing was binocular, but eye movements were monitored only from the dominant eye. The dominant eye was selected after initial calibration. Initial calibration before the practice trials was performed using 13 calibration points. Subsequently, a 5-point calibration was used before each set of 20 trials. Validation followed calibration and had to be evaluated as “GOOD” to move on with the experiment (i.e., using boundaries provided by SR; worst point error < 1.5 degree AND average error < 1.0 degree). Calibration accuracy was also checked before each trial (using a drift-check point—same as the calibration point—to the mid-left of the screen, see above). In the case of significant drift (i.e., larger than two degrees of visual angle), the experiment would not move to the next trial and recalibration was initiated (see Online Supplement A, Figure S.2 and Table S.3 for drift error statistics and visual display per grade). In addition, recalibration was possible at any point during the experiment and occurred occasionally when a participant had shifted seating position in between trials (<0.5% of trials), causing the eye tracker to lose connection.

In line with other eye-tracking studies on orthographic learning (Joseph & Nation, 2018; Wegener et al., 2018), the main outcome used for the analyses reported below was gaze duration (i.e., the sum of all fixations made on the target before any fixations on subsequent words; first-pass viewing time). This measure is thought to cover all aspects of word recognition, from decoding to meaning activation (e.g., Juhasz & Rayner, 2003; Reichle et al., 1998). In addition, findings on the total reading time (i.e., the sum of all fixations made on the target, including re-reading; total viewing time) are also reported. Total reading time can be considered as a general comprehension measure encompassing all aspects of reading (e.g., Boston et al., 2008) and is expected to show the largest effects (see Liversedge et al., 1998, for an overview).

### Procedure

Testing took place at the start of the second half of the school year in January and February. Children were tested individually in two sessions at their schools. In the first session, trained research assistants conducted the eye-tracking session implementing the orthographic learning task, during which the online measures were collected. This session lasted 30–60 min for the Grade 2 children and 20–35 min for the Grade 5 children. The second session was scheduled between 2 and 5 days (*M* = 3.23, *SD* = 1.23 days) after the orthographic learning task. This session lasted about 45 min, during which trained and supervised undergraduate students administered the offline measures and reading-related tasks. Tasks were part of a larger test battery and administered in a fixed order (i.e., spelling to dictation, orthographic choice, word reading, pseudoword reading, followed by other tests not reported here). If children were absent due to illness, their second session was scheduled to take place as soon as possible after their return. Schools were informed about the results of the standardised tests from the second session in a class-level report after data collection was finished.

### Data preparation

Decoding errors were marked for the target (*n*), preceding word (*n* − 1), following word (*n* + 1), and the rest of the sentence by offline listening to the audio files saved during the orthographic learning task. Trials with empty or incomplete vocal responses were excluded. For the eye-tracking data, each individual word in each sentence was defined as a separate interest area with a height of 60 pixels. As the full vertical extent of the row could range between 15 and 20 pixels (e.g., including capital letters, small letters, and quotation marks), margins amounted to one max row height above and below the main row for all interest areas. Interest area reports, excluding fixations immediately before and/or after blinks and with fixations shorter than 80 ms merged into longer fixations within a distance of 0.5 degree of visual angle (within the same area of interest; Pagán & Nation, 2019), were generated with Data Viewer 3.1.97 (SR Research, 2017).

## Results

### Data screening

Children with very long median word viewing times (i.e., G2 > 2,500 ms; G5 > 1,000 ms) or many decoding errors (i.e., > 150 taking into account all words in the sentences of the orthographic learning trials) were excluded from the study (*n* = 9, 3.8% of the trials). All of these children also showed raw (pseudo)word reading scores towards the lower end of the sample distribution per grade (see Online Supplement A, Figures S.4 and S.5). Four children who participated in the eye-tracking session could not attend the second (behavioural) test session due to sickness. Therefore, the total sample for the offline measures is 127 (i.e., G2: *n* = 73, *M*_age_ = 92.8 months, *SD*_age_ = 4.6 months; G5: *n* = 54, *M*_age_ = 129.0 months, *SD*_age_ = 4.5 months). Descriptives are provided in Table 2.

**Table 2.** Descriptive statistics background measures.

Variable	Grade 2				Grade 5			
	*M*	*SD*	Min	Max	*M*	*SD*	Min	Max
WR raw score^a^	48.7	13.0	23	101	77.2	11.8	57	103
WR standard score^b^	13.2	2.3	7	19	11.5	2.7	7	18
PWR raw score^a^	39.1	14.4	10	82	66.1	15.2	29	97
PWR standard score^b^	12.6	2.8	5	19	11.7	2.8	5	18

*SD*: standard deviation; WR: word reading; PWR: pseudoword reading.

*N* = 127.

a Maximum score is 116.

b *M* = 10, *SD* = 3.

### Offline outcomes

**Figure 1.**
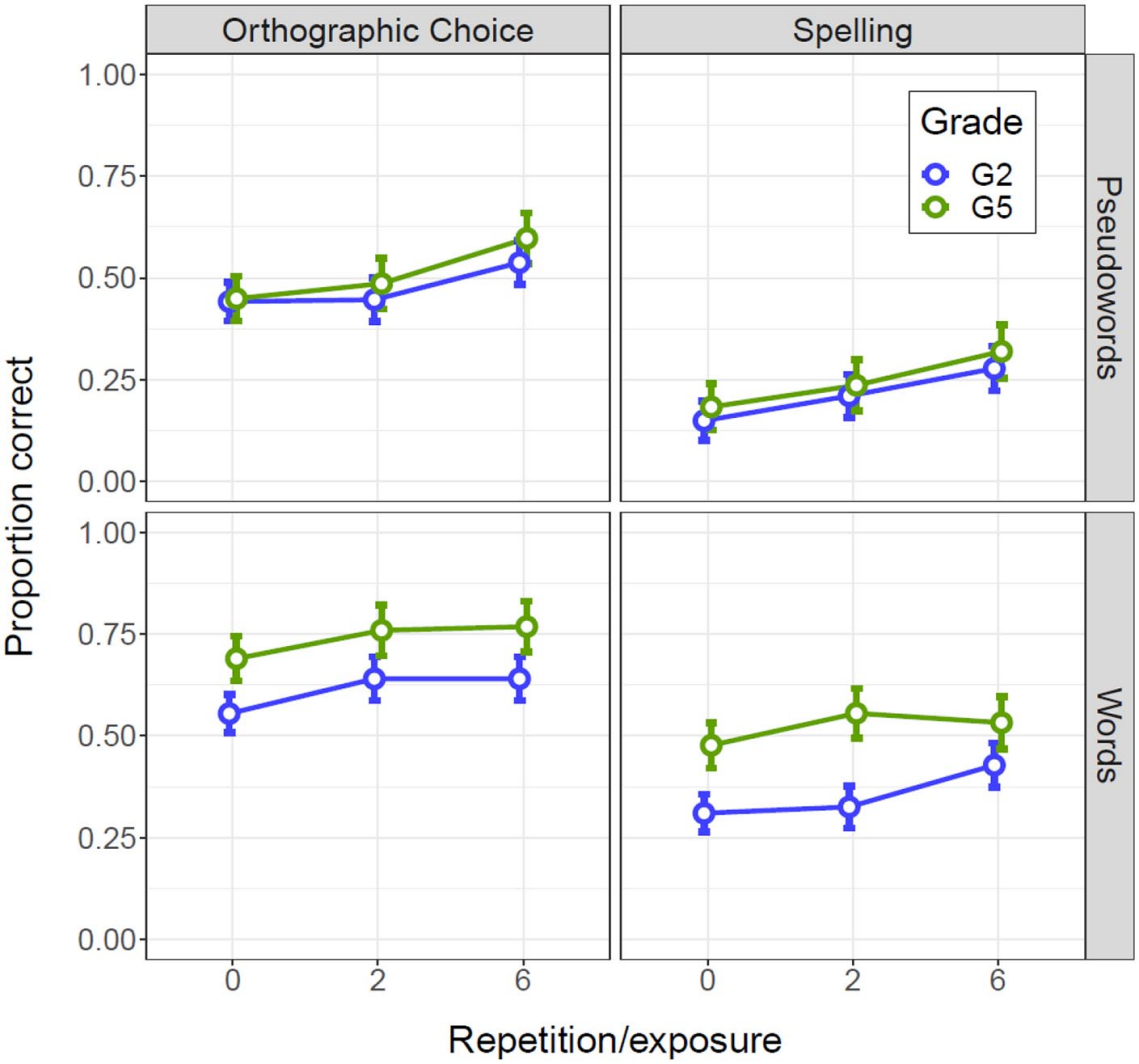
Orthographic choice (left) and spelling (right) results for orthographic learning, split by Grade and Lexicality. The error bars show the 95% within-participant confidence intervals (Baguley, 2012). For Grade, blue = Grade 2, green = Grade 5; for Lexicality, top panel = pseudowords, bottom panel = words.

Descriptives for offline outcomes are reported in Table 3. Figure 1 shows the performance of the Grade 2 and Grade 5 children on the orthographic choice and spelling tasks across exposures split by lexicality. The zero-exposures condition refers to target items not encountered during the orthographic learning task.

**Table 3.** Descriptive statistics for offline outcomes.

Variable	Grade 2				Grade 5			
	*M*	*SD*	Min	Max	*M*	*SD*	Min	Max
Orthographic choice^a^	0.53	0.09	0.28	0.78	0.61	0.09	0.41	0.78
Spelling dictation^a^	0.27	0.08	0.09	0.44	0.37	0.10	0.19	0.75

*SD*: standard deviation.

*N* = 127.

a Proportion correct.

Accuracy in the orthographic choice and spelling of target items for orthographic learning was analysed with generalised linear mixed-effects (LME) models (Baayen et al., 2008) using function glmer (binomial family with a logit link) of the lme4 package v. 1.1-27 (Bates et al., 2015) in R version 4.1.0 (R Core Team, 2021). Grade and lexicality were difference-coded (i.e., −0.5 vs. +0.5) and the number of exposures was coded as successive differences using function contr.sdif from the MASS package (Venables & Ripley, 2002), so that effects would be evaluated at the mean and slope estimates would correspond to the difference between levels (in two-level factors) or between successive levels (in the ordered three-level factor), thus being interpretable as simple main effects. We applied a model trimming approach, starting with a maximal model including number of exposures (0-2-6), grade level (G2-G5), lexicality (words-pseudowords), and their interactions as fixed-effects factors, and random intercepts for participants and target items and all applicable random slopes (i.e., exposure and lexicality, for participants, and exposure and grade, for items), including their interactions, in the random-effects structure.

Models generally converged, with appropriate choice of optimisers. However, maximal models resulted in singular convergence, consistent with overparameterisation, that is, too rich random structure that was unsupported by the available data (Matuschek et al., 2017). Subsequently, models were simplified, first by forcing random effects to be uncorrelated and then by iteratively removing negligible random effects, until nonsingular convergence was achieved. Resulting models were compared with the full model (with maximal random structure) using the chi-square difference test and Akaike’s information criterion (AIC), for which a lower value indicates better model fit (Kline, 2011). The complete model trimming procedure, along with full model outputs and comparison tests, is documented in Online Supplement B,^1^ sections 3.3 and 4.2. Model parameter estimates for the final models of both orthographic choice and spelling are displayed in Table 4.

**Table 4.** Linear mixed effects for orthographic choice and spelling outcomes including exposures, grade, and lexicality of the final model.

Random effects	Orthographic choice		Spelling	
	Variance	*SD*	Variance	*SD*
Participant				
(Intercept)	0.03	0.18	0.15	0.39
Exposure (2−0)			0.09	0.30
Exposure (6−2)	0.18	0.42	0.06	0.24
Lexicality			0.08	0.27
Target				
(Intercept)	0.70	0.84	2.29	1.51
Exposure (2−0)	0.05	0.23		
Exposure (6−2)	0.15	0.39		
Grade (G5−G2)	0.76	0.87	0.81	0.90
Fixed effects	Estimate	*z*	Estimate	*z*
(Intercept)	0.49	3.15**	−1.08	−3.91***
Exposure (2−0)	0.22	2.39*	0.27	2.55*
Exposure (6−0)	0.26	2.02*	0.38	3.28**
Lexicality (p−w)	−1.03	−3.34***	−1.47	−2.69**
Grade (G5−G2)	0.49	2.75**	0.73	3.64***
Exp (2−0) × Lex (p−w)	−0.34	−1.72	0.20	0.98
Exp (6−2) × Lex (p−w)	0.39	1.56	0.25	1.11
Exp (2−0) × Grade (G5−G2)	0.12	0.65	0.18	0.85
Exp (6−2) × Grade (G5−G2)	0.12	0.55	−0.34	−1.48
Lex (p−w) × Grade (G5−G2)	−0.65	−1.86	−0.63	−1.67
Exp (2−0) × Lex (p−w) × Grade (G5−G2)	0.10	0.27	−0.49	−1.19
Exp (6−2) × Lex (p−w) × Grade (G5−G2)	0.03	0.07	0.84	1.87
Analysis of deviance table^a^	*df*	χ^2^	*df*	χ^2^
Exposure	2	14.50***	2	36.16***
Lexicality	1	10.32**	1	7.53**
Grade	1	6.44*	1	13.70***
Exposure × Lexicality	2	4.01	2	4.74
Exposure × Grade	2	1.68	2	2.63
Lexicality × Grade	1	3.75	1	2.61
Exposure × Lexicality × Grade	2	0.15	2	3.52

*SD*: standard deviation; Exp: exposure; Lex: lexicality.

For Exposures, 0 = 0 repetitions, 2 = 2 repetitions, 6 = 6 repetitions; for Lexicality, w = words, p = pseudowords; for Grade, G2 = Grade 2, G5 = Grade 5.

a Type II Wald chi-square tests produced by function Anova from library car v. 3.0-10 (Fox & Weisberg, 2019).

* p < .05. **p < .01. ***p < .001.

The results showed an average effect of Exposure (zero vs. two repetitions and two vs. six repetitions), indicating significant early and late learning, an average effect of Lexicality, indicating higher performance on words, and an average effect of Grade, indicating higher performance in Grade 5, on both outcomes. There were no significant two-way interactions. This indicates that neither grade nor lexicality affects orthographic learning very much. To increase the power to detect two-way interactions, that is, factors affecting the amount of learning across exposures, we also modelled the total learning effect (i.e., zero vs. six exposures) by discarding the two-exposures condition (see Online Supplement B, sections 3.7 and 4.6). For orthographic choice, this did not result in any significant interactions between Exposure and Lexicality or Grade. For spelling, this resulted in a significant interaction between Exposure and Lexicality, suggesting that lexicality affects the amount of orthographic learning such that there is a larger overall effect of exposure on learning the spelling of pseudowords than on learning the spelling of (partially) known words.

#### Power considerations

These analyses involve 127 participants and 32 target items (counterbalanced among participants and exposure conditions). In a conservative approach to estimating power, we consider the standard errors of the effect estimates in the full model for orthographic choice, which are around 0.125 for each exposure contrast (i.e., early and late learning effect), 0.250 for interactions with exposure (lexicality and grade modulating learning), and 0.500 for three-way interactions. In this exploratory study, we are primarily interested in factors affecting learning, that is, the two-way interactions. In general, the power to detect an effect as large as 3 standard errors is about 85%. This means that we can expect to detect interaction effects of 0.75 or greater. Over a grand-mean intercept of about 0.5 (hence average accuracy 62%; ignoring other effects for the purpose of illustration), this means that a two-way interaction effect would have to bring accuracy up to 75% or down to 48% to be reasonably detectible.

To validate this approach (and its conservativeness), we used library simr (version 1.0.5; Green & MacLeod, 2016) to sample from the random and fixed effects structure of the overall-effects model (zero vs. six exposures, difference-coded), due to its more rapid convergence. In this model as well, SEs for two-way interactions did not exceed 0.25, leading to a prediction of at least 50% power to detect an effect of 0.50 (i.e., 2 SE). Based on 1,000 simulated samples, the lowest estimated power was 61%, exceeding the predicted value, as expected. We can thus conclude that our study is moderately powered to detect large two-way interactions indicating modulation of orthographic learning rate by grade or lexicality (see Online Supplement B, sections 3.6, 4.5, 5.1, and 5.2, for more details).

### Online measures

#### Data clean up

Learning across exposures is modelled using all available data points for all items. This means that, as half of the items were encountered twice and half were encountered six times, there are twice as many data points for the first and second exposure as there are for exposures three to six. In line with previous studies on eye movements of beginning readers (Rau et al., 2015, 2016; Vorstius et al., 2014), remaining viewing times on the target shorter than 80 ms were removed from the data (9.8%). In addition, gaze durations longer than 4,000 ms and total reading times longer than 6,000 ms (0.4%) on the target were excluded. Trials in which the target item was skipped or incorrectly decoded (11.8%) were also not taken into account. Combined this resulted in a total data loss of 22.0% on the main outcome (see Online Supplement A, Table S.6, for an overview of trial counts and percentages). Gaze duration and total reading times were log transformed to reduce skewness. Descriptives for online outcomes are reported in Table 5.

**Table 5.** Descriptive statistics for online outcomes.

Variable	Grade 2				Grade 5			
	*M*	*SD*	Min	Max	*M*	*SD*	Min	Max
Gaze duration^a^	792.3	674.8	80	3,984	435.1	344.6	80	3,408
Total reading time^a^	1,173.0	912.6	82	5,886	617.0	443.5	80	3,671

*SD*: standard deviation.

*N* = 131.

a On target in milliseconds (untransformed).

#### Gaze duration on target

Results are reported here in full for the main online outcome measure of gaze duration. Corresponding results for total reading time are mentioned in a separate section below. Because of the obviously nonlinear relationship between number of exposures and gaze duration, gaze durations were analysed with generalised additive mixed-effects modelling (GAMM) using the mgcv package v. 1.8-35 (Wood et al., 2016) in R version 4.1.0 (R Core Team, 2021). GAMM is a relatively recent addition to the researcher’s arsenal, but several guides and tutorials have by now been made available to make this approach more accessible (e.g., Baayen et al., 2017; Pedersen et al., 2019; Porretta et al., 2017; van Rij, 2015; Wieling, 2018; Wood, 2006). GAMM provides the possibility to estimate nonlinear regression curves and assess the influence of fixed effects on the shape of the curves (Baayen et al., 2017). We used GAMM rather than a generalised linear model to avoid the limitations arising from having to choose a specific distribution and link function, that is, from a priori committing to a specific curve shape without a concrete model of the learning effects. The more traditional approach to nonlinear effects, namely treating exposure as an ordinal factor with each repetition being a different level, was not preferred due to the limited amount of data (few data points per participant per cell), which would lead to overly noisy estimates and potentially uninterpretable results. In comparison, the GAMM approach fits a curve constrained to be smooth across repetitions, in effect taking all levels simultaneously into account and thereby producing a more reliable estimate of the relationship between exposure and gaze duration.

Notably, in GAMM one fits a single curve for each modelled term. Thus, differences in the shape of the curve can be modelled as additional terms, which can be formally tested for statistical significance by comparing against a straight line. In contrast, in the (more traditional) polynomial approaches, the outcome is modelled as a sum of terms (linear, square, cube, etc.). Many such terms are required to adequately model arbitrary curve shapes, but each term is individually uninterpretable. Differences between curves can be modelled as interactions between these individual terms and other factors, which can be formally tested for significance (with very low power) but do not exactly amount to the question of interest, namely whether two curves differ in shape or not.

Within the context of our study, learning can be modelled as a continuous curvilinear relationship (a “smooth term”) between an outcome variable (here, gaze duration) and number of exposures. Testing whether a factor affects learning rate amounts to testing whether two such smooth terms are identical in shape or not. In other words, testing for an interaction between a smooth term and a factor refers to testing whether multiple smooth terms (i.e., one for each level of the factor) are statistically justified. When more than two are involved, it is clearer to model an overall “learning curve” and then testing whether condition-specific curves are statistically distinguishable from straight lines. In addition to these smooth terms, GAMMs also include fixed and random effects as commonly understood in standard regression.

**Figure 2.**
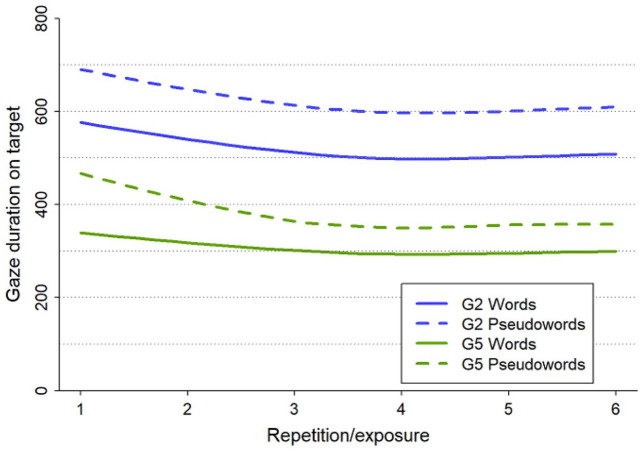
Summed smooth effects of exposure on gaze duration on target, split by Grade and Lexicality, back-transformed to the original time scale in milliseconds.

We applied a model trimming approach starting with a maximal model including all fixed effects and their interactions, smooth terms and their interactions with the fixed factors, random intercepts, and random slopes. The whole procedure from initial to final models is fully documented in Online Supplement C, section 2. Here, we only report the resulting simplified model (see Table 6). There were significant effects of Grade and Lexicality, indicating longer gaze durations for Grade 2 children and longer gaze durations for pseudowords, respectively. Turning to the smooth effects of exposure, there was of course a significant nonlinear overall smooth term indicating an effect of Exposure. Importantly, there was a significant interaction between Exposure and Grade and Lexicality, evident in a significant additional smooth for exposure to pseudowords in Grade 5. As displayed in Figure 2, plotted using function plot_smooth of package itsadug v. 2.4 (van Rij et al., 2020), there were clear learning effects across exposures, with decreasing gaze durations after each exposure. This trend was decelerating, reaching an asymptote after 3–4 exposures. However, specifically in Grade 5, the learning effect (i.e., the slope of the curve) was larger for pseudowords than for words, especially during the first couple of exposures.

**Table 6.** Generalised mixed additive model fitted to log-transformed gaze durations on target.

Fixed effects	Estimate	*t*	
(Intercept)	6.11	160.58***	
Grade	−0.50	−11.20***	
Lexicality	0.21	3.40***	
Grade × Lexicality	0.06	1.41	
Smooth terms for exposure	Estimated *df*	Reference *df*	*F*
Overall term	2.6	3.1	11.31***
Grade 2 Pseudowords	<0.001	5	<0.001
Grade 2 Words	<0.001	5	<0.001
Grade 5 Pseudowords	2.5	5	2.20**
Grade 5 Words	<0.001	5	<0.001
Random intercepts	Estimated *df*	Reference *df*	*F*
Participants	102.5	129	7.28***
Sentences	38.7	63	1.79***
Targets	27.0	30	13.51***
Random slopes	Estimated *df*	Reference *df*	*F*
Lexicality (Participants)	39.0	258	0.26*

Lex: lexicality.Fixed effects include linear predictors. Smooth terms include nonlinear predictors and interactions.

* *p* < .05. **p < .01. ***p < .001.

#### Total reading time

The findings for total reading time on the target are similar to those for gaze duration. The only difference is that, as expected, effects are larger and total viewing times are longer. Model parameter estimates for the total reading time are provided in Online Supplement C section 4.2 and graphically displayed in section 4.4.

## Discussion

In this study, we used a standard orthographic learning paradigm to systematically assess word- and person-level factors relevant for establishing orthographic representations and gain more insight into reading fluency development. Traditional offline learning outcomes were combined with online reading measures obtained through eye tracking to reveal changes in reading processes associated with orthographic learning. Our hypothesis regarding lexicality as a word-level factor was only partly confirmed. The findings indicated that lexicality (i.e., words vs. pseudowords) may be important to consider when investigating the time course of forming orthographic representations in terms of the required number of repetitions (i.e., exposures). Moreover, the influence of lexicality was found to partly differ across development, that is, between beginning and more advanced readers. Yet, concerning person-level factors, beginning readers and more advanced readers seem equally efficient at building up (offline) orthographic knowledge of specific words across repeated exposures, despite some difference in dealing with varying lexicality. Overall, orthographic learning was found to increase the (online) reading speed of initially unfamiliar but partly known target words throughout exposure equally across reading-skill levels, while more advanced readers show an advantage when learning pseudowords.

One of the assets of this study is that it combines offline measures of orthographic learning (i.e., orthographic choice and spelling to dictation) and online measures (i.e., eye tracking). As stated earlier, the nature of the two types of measures is inherently different and warrants a different interpretation regarding reading processes and learning outcomes for orthographic learning (see, for example, Protopapas & Kapnoula, 2016). The online measures offer information about changes in short-term activation of orthographic word forms during repeated reading of words, whereas the offline measures provide insight into long-term representation learning, that is, the outcome of orthographic learning, measured after a retention period of multiple days. As expected, our findings show that the transient effect of priming of orthographic word forms only partially led to permanent effects in terms of learning of orthographic representations. Therefore, in the following we discuss the findings for both types of outcomes together, but separately per condition, aiming to highlight both concordances and contradictions.

### Exposure

Our findings on the required amount of exposure for orthographic learning to occur, in terms of the number of repetitions, fill some important gaps in the literature concerning the time course and conditions under which orthographic representations of specific words are formed. By applying a design including a range of exposures (i.e., from zero to six), we were able to show that a sharp decrease in viewing time occurred as early as after one exposure, indicating efficient short-term activation of orthographic forms. Viewing times continued to decline with subsequent exposures and levelled out after four exposures, in line with the findings of a previous study in adults (Kamienkowski et al., 2018; see also Hung et al., 2013; Parrila & Barber, 2011; Parrila & Turgeon, 2012). Although our study is one of the first to assess this in children (see also Liang et al., 2021), the comparison with adults suggests that the required amount of exposure for short-term activation of orthographic representations is similar across development. This is further confirmed by the statistically indistinguishable learning curves on orally known words for beginning and advanced readers on online measures in our study.

In comparison, the offline outcomes show that consolidated orthographic knowledge, as measured by orthographic choice and spelling several days post exposure, is already established after two repetitions (i.e., early learning), but also continues to grow between three and six exposures (i.e., late learning; combined an indication of protracted learning). Although the former is in line with other studies (e.g., de Jong & Share, 2007; Share, 1999), the study by Nation et al. (2007) was, to our knowledge, the only one to find further learning after the initial exposure (but see de Jong et al., 2009). The general absence of interactions between the number of exposures and reading-skill level for offline learning outcomes further suggests that the reading systems of beginning and more advanced readers seem equally effective in building up orthographic representations of new written word forms in the long run, although their systems might be less efficient given their longer viewing times. However, some developmental differences may surface depending on word-level characteristics.

### Lexicality

Concerning the influence of lexicality, the online measures in our study showed that short-term activation across exposures differs between words and pseudowords. A decrease in viewing times occurred from the first exposure for both words and pseudowords, but learning effects turned out to be larger for pseudowords, especially in more advanced readers during the first couple of exposures. These findings are in line with novel-word effects found in previous eye-tracking studies in adults (e.g., Brusnighan et al., 2014; Lowell & Morris, 2014; Wochna & Juhasz, 2013) and children (e.g., Hyönä & Olson, 1995; Joseph et al., 2013; Liang et al., 2021), indicating longer viewing times for pseudowords than words.

The offline measures confirmed that the time course for long-term retention of formed orthographic representations can be different for novel words (i.e., pseudowords) and orally known words (i.e., here low-printed-frequency words), but may depend on the level of detail of orthographic representations that is required. The results suggest that learning of novel words and orally known words can already happen after one or two exposures when less detailed orthographic representations are required (i.e., for orthographic choice, where only the targeted irregularity has to be stored and recognised correctly). This finding is new for orally known words, as previous studies only confirmed orthographic learning of words after a threshold of four exposures (Reitsma, 1983, 1989), but in line with other studies finding evidence that orthographic learning of novel words can already happen after one exposure (e.g., Nation et al., 2007; Share, 2004).

However, when more detailed orthographic representations are required (i.e., for spelling, where every letter in the target item has to be stored and reproduced correctly), lexicality does seem to play a role in orthographic learning. Although also for spelling orthographic learning was found to happen already after one or two exposures, and results indicated prolonged learning between three and six exposures, overall learning effects across all exposures were found to be larger for novel words than for orally known words. In other words, learning amounts are higher when there is more to learn.

The latter is not in line with our expectations. We hypothesised that shorter viewing times on known words would translate into more early learning on offline outcomes due to the availability of a phonological representation. Yet, it seems to work the other way around. The availability of an orthographic skeleton for orally known words provides a different starting point for orthographic learning, which seems to require less learning and at a lower rate (given the slower decrease in viewing times). In contrast, children start from scratch when they encounter novel words and may have to update their orthographic representations more radically across exposures, resulting in a higher rate of learning (as indicated by faster decreasing viewing times).

Taken together, more advanced readers might show stronger short-term priming effects for pseudowords already early on, but they do not bridge the gap with words in terms of how activated their orthographic forms become during repeated reading within the full range of six exposures. In that regard, the changes in online reading processes seem to point largely in the same direction as the long-term retention outcomes for learning orthographic representations of known and novel words.

### Reading skill

Including both beginning and more advanced readers in the current study has provided a unique opportunity to observe differences between and changes in reading processes across different levels of reading skill. As stated before, there are two possibilities regarding the influence of reading skill on orthographic learning: (1) despite possible differences regarding lexicality, the reading system of beginning readers in Grade 2 could be equally effective in the building-up of orthographic representations, or (2) orthographic representations could be more rapidly acquired in the better developed reading system of more advanced readers in Grade 5. The general absence of interactions between the number of exposures and grade for offline learning outcomes suggests that the rate of orthographic learning is likely not greatly influenced by reading-skill level. For the online measures, we observe largely the same. There are no differences between beginning and advanced readers regarding the short-term activation of written word forms for orally known words. For novel words, advanced readers do show more efficient short-term activation of written word forms, but this does not translate to higher performance on long-term retention of word-specific orthographic information (given the absence of an interaction with grade for offline outcomes). Hence, we can conclude that our findings suggest that, overall, the reading systems of beginning and more advanced reads seem equally efficient at building up detailed orthographic knowledge. We do not find support for the assumption that orthographic representations are more rapidly acquired when the reading system is better developed, as is the case in more advanced readers. This does not seem to be an issue of low statistical power, as there is no evidence of different trends by grade in the plotted raw data (see Figure 1).

### Implications

Our findings on the potential role of lexicality are relevant in light of the orthographic skeleton hypothesis (e.g., Wegener et al., 2018) and the related discussion about the relevance of the availability of meaning or context versus the availability of a phonological representation for orthographic form learning. Although we found some differences between words and pseudowords in the establishment of detailed orthographic representations, it is not likely that these lexicality effects result from the presence or absence of word meaning. As previous studies have shown that the availability of meaning does not lead to the formation of more detailed orthographic representations (e.g., Hogaboam & Perfetti, 1978; Share, 2004), the differences that we found on online and offline measures are not large enough to suggest otherwise. Likewise, context does not seem to play a major role either, as the sentences in our study were as context-neutral as possible and provided only clues as to whether the target item represented an animate or inanimate entity.

The availability of a phonological representation, however, seems more relevant. de Jong et al. (2009) showed in an orthographic learning study that homophones that differed by one letter from a target word were read much faster than non-homophone words that also differed by one letter from the target. This suggests that children might form an initial orthographic representation on the basis of the phonological representation that is already present in their mental lexicon. This initial orthographic representation then functions as a starting point for orthographic learning, resulting in faster learning of written word forms. Although this suggestion fits the higher spelling performance and shorter viewing times for orally known words compared to novel words, we found no evidence that it also leads to faster rates of orthographic learning, both concerning short-term activation and long-term retention. Therefore, more research is needed to further investigate how children might form and use orthographic expectations (e.g., using orthographic pattern knowledge) and how initial word-specific representations and orthographic knowledge in general is updated throughout learning.

There are also implications for theories on reading fluency development. Our findings of decreasing viewing times on target words in sentences across exposures are new within the context of orthographic learning. They are, however, consistent with previous findings within the larger eye-tracking framework, if we assume that our repeated-exposure paradigm constitutes in essence a word-frequency manipulation (e.g., see Rayner, 1998, for an overview). That is, target items become increasingly familiar during the course of the eye-tracking session, resulting in changes in reading processes. Most importantly, more or better knowledge of specific words translates to faster/more efficient processing of these words, and possibly of surrounding words as well (see Heister et al., 2012, for an overview).

### Limitations

In light of the semi-transparent nature of the Dutch orthography, a possible limitation might lie in the criteria for target selection. Due to the limited number of one-syllable words with irregular spelling but a known meaning for 6- to 7-year-old children, being unfamiliar with a word’s spelling was prioritised over being familiar with a word’s meaning in the final target selection. A downside of this choice may be that this might have influenced the possibility of finding lexicality effects for Grade 2 children, as some low-frequency words may have functioned as pseudowords. Yet, higher familiarity with the known words’ meanings could have artificially increased lexicality effects, implying that our current reporting of lexicality effects can be considered conservative. An important benefit of this approach to target selection is that there were no ceiling effects on the offline learning outcomes for orthographic learning. In addition, higher unfamiliarity with some of the words likely increased the need for orthographic learning, possibly because some of the children might not have had a phonological representation available to form an initial orthographic representation on. As such, there was more room for children to show actual learning of written word forms and thus a better opportunity to estimate learning curves across exposures.

Another possible limitation could be that the sentences we used in the experiment all followed a fixed template around the position where the target would be placed (i.e., article, adjective, noun, verb). A recent study about the potential effect of statistical regularities on sentence reading has shown that repeatedly encountering a specific syntactic structure influences both linguistic processing and oculomotor control, leading to fewer and shorter fixations (Snell & Theeuwes, 2020). However, adjusting for overall sequential effects showed that trial order did not have an influence (i.e., as indicated by an almost flat smooth) and the results for the learning effects were unchanged (see Online Supplement C, section 3).

Concerning task instruction, a limitation might be that all children were asked to read the sentences in the orthographic learning task aloud. This is a typical mode of reading for the beginning readers from Grade 2, but it may have been less natural for the more advanced readers from Grade 5. Although future research should reveal if our findings also generalise to silent reading conditions, our results are compatible with Wegener et al. (2018) who instructed children to read silently.

Finally, we acknowledge that our exploratory study contains many factors that may be relevant for orthographic learning, but that the number of items is not sufficient to detect small effects or to draw conclusions about the combined effects of these factors. Although the power to detect higher-order interactions is too low, our focus on two-way interactions with the number of exposures does allow us to detect moderate-to-large effects on learning rate that can be elaborated in future research. Through our approach towards model comparisons, aiming to settle on a random structure that is as rich as supported by the observed variability in the data, we have been able to illustrate that standard errors for the estimates do not become unduly small by removing negligible random slopes and correlations, and that they are highly stable across models (see model comparisons in Online Supplement B). As such, we are confident that we have sufficient power to detect simple interactions and draw conclusions that are informative and provide clear directions for follow-up research.

## Conclusion

Overall, our findings show that word- and person-level factors may play a role in orthographic learning and are therefore important to consider systematically in future studies.

In general, taking gaze duration as a proxy for reading speed, we can say that orthographic learning was found to increase the reading speed of initially unfamiliar target words throughout exposure. Effects of exposure, lexicality, and reading-skill level have been found, as well as some interactions between word- and person-level characteristics and the number of exposures. The overall pattern that emerges is one of longer gaze durations for what is more difficult and shorter gaze durations for what is easier. However, this does not lead to clearly discernible patterns in the amount of learning across exposures. In fact, learning rates and amounts seem to be highest where there is most to learn (i.e., novel words). Yet, interactions between reading-skill level and the number of exposures were generally absent. Therefore, we conclude that although the reading system of more advanced readers may be somewhat better equipped to deal with novel words, the reading systems of beginning and relatively advanced readers are equally efficient in gradually building up orthographic knowledge of specific words. In other words, based on this study, we found no evidence for faster formation of orthographic representations in a better developed reading system. How building orthographic knowledge of specific words may influence the wider processing cascade involved in fluent reading should be addressed in future studies.

## Supplemental Material

sj-pdf-1-qjp-10.1177_17470218211047420 – Supplemental material for Lexicality effects on orthographic learning in beginning and advanced readers of Dutch: An eye-tracking study Supplemental material, sj-pdf-1-qjp-10.1177_17470218211047420 for Lexicality effects on orthographic learning in beginning and advanced readers of Dutch: An eye-tracking study by Sietske van Viersen, Athanassios Protopapas, George K Georgiou, Rauno Parrila, Laoura Ziaka and Peter F de Jong in Quarterly Journal of Experimental Psychology

sj-pdf-2-qjp-10.1177_17470218211047420 – Supplemental material for Lexicality effects on orthographic learning in beginning and advanced readers of Dutch: An eye-tracking study Supplemental material, sj-pdf-2-qjp-10.1177_17470218211047420 for Lexicality effects on orthographic learning in beginning and advanced readers of Dutch: An eye-tracking study by Sietske van Viersen, Athanassios Protopapas, George K Georgiou, Rauno Parrila, Laoura Ziaka and Peter F de Jong in Quarterly Journal of Experimental Psychology

sj-pdf-3-qjp-10.1177_17470218211047420 – Supplemental material for Lexicality effects on orthographic learning in beginning and advanced readers of Dutch: An eye-tracking study Supplemental material, sj-pdf-3-qjp-10.1177_17470218211047420 for Lexicality effects on orthographic learning in beginning and advanced readers of Dutch: An eye-tracking study by Sietske van Viersen, Athanassios Protopapas, George K Georgiou, Rauno Parrila, Laoura Ziaka and Peter F de Jong in Quarterly Journal of Experimental Psychology

## Notes

1. Online Supplements B and C can be accessed at the Open Science Framework through the following link: https://osf.io/26nvz/?view_only=80e89df2569d4c04aa7bdbdefac796ff.
