# Supplementary material for: Lexicality effects on orthographic learning in beginning and advanced readers of Dutch: An eye-tracking study
Source: Q J Exp Psychol (Hove). 2021 Sep 28;75(6):1135–54. doi: 10.1177/17470218211047420 (PMC9016678; doi:10.1177/17470218211047420)
Supplement: sj-pdf-1-qjp-10.1177_17470218211047420 – Supplemental material for Lexicality effects on orthographic learning in beginning and advanced readers of Dutch: An eye-tracking study [file sj-pdf-1-qjp-10.1177_17470218211047420.pdf]

Online Supplement A

Table S.1

Original Sentence Templates in Dutch with English Translation and Word Count

#	Dutch	English	Count
Experimental sentences for animate targets			
1	Je kan een zeldzame _ zien in de dierentuin.	You can see a rare _ at the zoo.	9
2	De lome _ rust elke dag een tijdje uit.	The lazy _ rests for some time every day.	9
3	De hongerige _ ging daar zoeken naar eten.	Where did the hungry _ go to look for food?	8
4	Ik was blij dat de laatste _ verdween voor hij gepakt werd.	I was happy that the last _ disappeared before he was caught.	12
5	De stille _ knipperde met zijn ogen.	The silent _ blinked his eyes.	7
6	Ik vraag me af of de verdrietige _ terugkomt naar zijn familie.	I wonder if the sad _will return.	12
7	Moeder had een schattige kleine _ gezien op vakantie.	Mother saw a cute little _ when she was on holiday.	9
8	In de middag werd de oude _ gevoerd door zijn verzorger.	The old _ was fed by his keeper in the afternoon.	11
9	Naar een jonge _ kijken is altijd leuk.	Watching a young _ is always fun.	8
10	De vermoeide _ deed een dutje na de lange dag.	The tired _ had a good nap after the long day.	10
11	Een hongerige _ steelt soms eten van mensen.	The hungry _ sometimes steels food from people.	8
12	Bijna elke wilde _ kan op warme plekken leven.	Almost every wild _ can live in warm places.	9
13	Ik zag de sterke _ aanvallen toen haar jong bang was.	I watched the strong _ attack when her young was scared.	11
14	Een zieke _ houdt zichzelf moeilijk warm in de winter.	It is hard for a sick _ to keep himself warm during the winter.	10
15	Toen we een mooie _ zagen moesten we stoppen voor een foto.	When we saw a beautiful _ we had to stop for a picture.	12
16	Een wilde _ zorgt meestal voor problemen als huisdier.	A wild _ generally causes problems as a pet.	9
17	Ik moest om een grappige _ lachen laatst.	Last week, a funny _made me giggle.	8
18	Een boze _ keek me recht in mijn gezicht aan.	One angry _ looked me straight in the face.	10
19	Deze slimme _ zoekt graag overal naar eten.	This smart _ likes to look for food everywhere.	8
20	De bruine _ bewoog voorzichtig langs het hek.	The brown _ moved slowly alongside the fence.	8
21	Ik kon de luidruchtige _ horen in de verte.	I could hear the noisy _ in the distance.	9
22	De jonge _ sliep rustig zonder gestoord te worden.	The young _ was sleeping quietly without disturbance.	9
23	Mijn meester heeft een bedreigde _ gezien op de televisie.	My teacher saw an endangered _ on television.	10
24	Eenmaal volgroeid kan elke _ zwemmen in water.	Once fully grown, every _ can swim in water.	8
25	De kinderen willen over een bijzondere _ leren op school.	The children would like to learn about a special _ at school.	10

Online Supplement A

26	De bange _ ging ervandoor om zich te verstoppen.	The scared _ ran away to hide itself.	9
27	Ik was verrast dat ik een kleine _ zag op weg naar huis.	I was surprised I saw a small _ on my way home.	13
28	"De laatste tamme _ woont in een dierentuin" zei de juf.	Most tame _ are living in zoos, said the teacher	11
29	Ik zag een plaatje van een donkere _ staan in een boek.	I saw a picture of a dark _ in a book.	12
30	Het hart van de bange _ sprong op door een gek geluid.	The heart of the scared _ jumped when he heard a funny sound.	12
31	De gespikkelde _ zag ik voor het eerst bij Freek Vonk.	I first a speckled _ at Freek Vonk for the first time.	11
32	De snelle _ dronk veel water van de dorst.	The quick _ drank a lot of water because he was thirsty.	9
Experimental sentences for inanimate targets			
1	Als een grote _ valt maakt het veel geluid.	When a big _ falls it makes a lot of noise.	9
2	Ik zag dat de dikke _ past tussen twee kleinere.	I saw that the large _ fits between the two smaller ones.	10
3	Ik kan de laatste _ terugleggen bij de andere.	Can you put the last _ back with others?	9
4	Laat deze lelijke _ liggen in de doos.	Let this ugly _ stay in the box.	8
5	Ik kon de oude _ horen vallen achter de muur.	I could hear the old _ fall behind the wall.	10
6	Opeens zag ik een enorme _ slingeren boven de tafel.	Suddenly, I saw a huge _ swing above the table.	10
7	De beste _ maakt het mooiste geluid.	The best _ makes the most beautiful sound.	7
8	Laat niet de kleine _ glippen tussen je vingers.	Don't let the small _ slip between your fingers.	9
9	Jan heeft de zware _ neergelegd op de plank.	Bob put the heavy _ on top of the shelf.	9
10	De lange _ stuiterde weg toen hij op de grond viel.	The long _ bounced away when it hit the floor.	11
11	Toen Tim met een scherpe _ speelde bezeerde hij zijn kin.	When Tim was playing with a sharp _ he hurt his chin.	11
12	Op de schone _ zit toch nog een vlek.	There is still a spot on the clean _.	9
13	Moeder heeft de hete _ gekoeld en opgeruimd.	Mom cooled the hot _ down and stored it.	8
14	De jongen hielp mij de oude _ op te rapen van de grond.	The boy helped me pick up the old _ off the floor.	13
15	"Een goede _ werkt altijd" zei moeder.	A new _ always works, said mum.	7
16	"Kijk eens hoe deze dunne _ hangt aan de muur!" zei vader.	"Look how this thin _ is hanging on the wall!" said dad.	12
17	We moeten een nieuwe _ kopen voor in ons huis.	We need to buy a new _ in our house.	10
18	Ik was heel blij dat ik de verloren _ gevonden had.	I was very happy to find the lost _.	11
19	Ik kan de zware _ optillen omdat ik sterk ben.	I can lift a heavy _ because I am strong.	10
20	Gister zag ik vader een smalle _ verstoppen achter zijn rug.	Yesterday I saw dad hiding a thin _ behind his back.	11
21	"Niet met deze onhandige _ spelen" zei moeder.	"Do not play with this gawky _" said mom.	8

Online Supplement A

22	De meester heeft een harde _ gebruikt om de muur mee te raken.	The teacher used a hard _ to hit the wall.	13
23	De donkere _ bedekte het boek maar half.	The dark _ covered only half of the book.	8
24	We hebben een simpele _ gekocht voor als we gaan klussen.	We bought a plain _ for when we go building.	11
25	Moeder vroeg mij de glibberige _ neer te leggen in de gootsteen.	Mom asked me to put the oily _ in the sink.	12
26	Hij liet mij de overgebleven _ zoeken in de kast.	He let me search for the odd _ in the cupboard.	10
27	De rode _ ligt ergens in huis verstopt.	The red _ is hidden in the house somewhere.	8
28	De volgende _ knalde hard tegen het plafond.	The next _ popped loudly at the ceiling.	8
29	Een eenvoudige _ vinden is geen simpele klus.	Finding a simple _ is not an easy task	8
30	Een stevige _ maakt een gat in de vloer als je hem laat vallen.	A stiff _ makes a hole when it hits the floor.	14
31	De enige _ staat op zijn kop.	The only _ stands upside down.	7
32	De losse _ viel op een dag op de grond.	The lose _ fell on the ground one day.	10
Filler sentences (animate)			
1	De jonge raaf vloog snel naar zijn nest toe.	The young raven quickly flew back to its nest.	9
2	De zwarte kat liep langzaam over straat.	The black cat slowly walked on the street.	7
3	De mooie merel voedde haar jongen rupsen.	The beautiful blackbird fed her brood caterpillars.	7
4	Elke grote kameel trekt door de woestijn.	Every large camel travels through the desert.	7
5	De groene rups at zijn buikje vol met blaadjes.	The green caterpillar filled his belly with leafs.	9
6	De gemene wesp stak het meisje in haar arm.	The mean wasp stung the girl in her arm.	9
7	De kleine spin zat veilig in haar web te wachten.	The little spider was waiting safely in her web.	10
8	Het gevlekte kalf sprong vrolijk door de wei.	The spotted calf jumped happily through the meadow.	8
Filler sentences (inanimate)			
1	De kromme haak hing stevig aan het plafond.	The crooked hook was firmly attached to the ceiling.	8
2	De lange buis stak een heel eind uit de mand.	The long pipe stood out a long way out of the basket.	10
3	Vader liet de zware hamer vallen op de vloer.	Father let the heavy hammer drop on the floor.	9
4	De luide wekker rinkelde zo hard mogelijk.	The loud alarm clock rang as loudly as possible.	7
5	De roestige zaag kraakte tijdens het zagen.	The rusty saw creaked during sawing.	7
6	"Speel maar een liedje op je nieuwe fluit" zei moeder.	"Just play a song on your new flute" said mother.	10
7	Vader hing zijn smalle gordel aan een spijker aan de muur.	Father hung his narrow belt on a nail on the wall.	11
8	De lange mantel warmde Jip goed op.	The long cloak warmed Jip well.	7

Online Supplement A

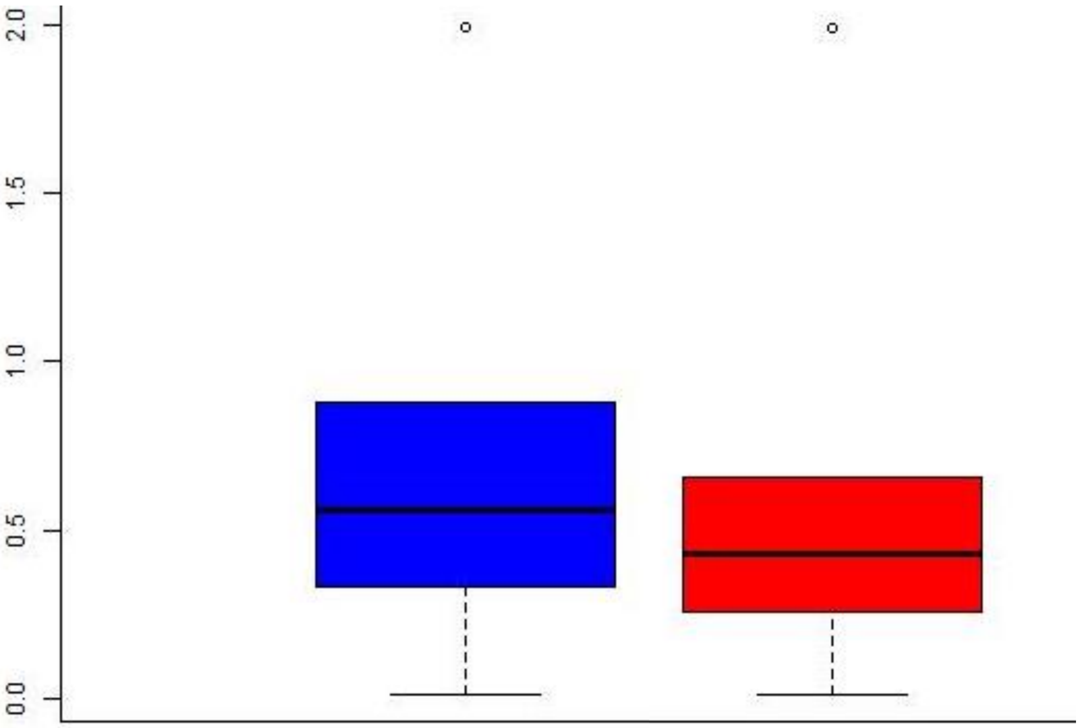

Figure S.2. Boxplots of minimum, 25th percentile (lower hinge), median, 75th percentile (upper hinge), and maximum values for drift error (in degrees of visual angle) in Grades 2 (blue) and 5 (red).

Table S.3

Descriptives for Drift Error (in degrees of visual angle) in both Grades

	Min	25th pct	Median	75th pct	Max
Grade 2	0.01	0.33	0.56	0.88	1.99
Grade 5	0.00	0.25	0.42	0.65	1.99

Note. pct = percentile.

Online Supplement A

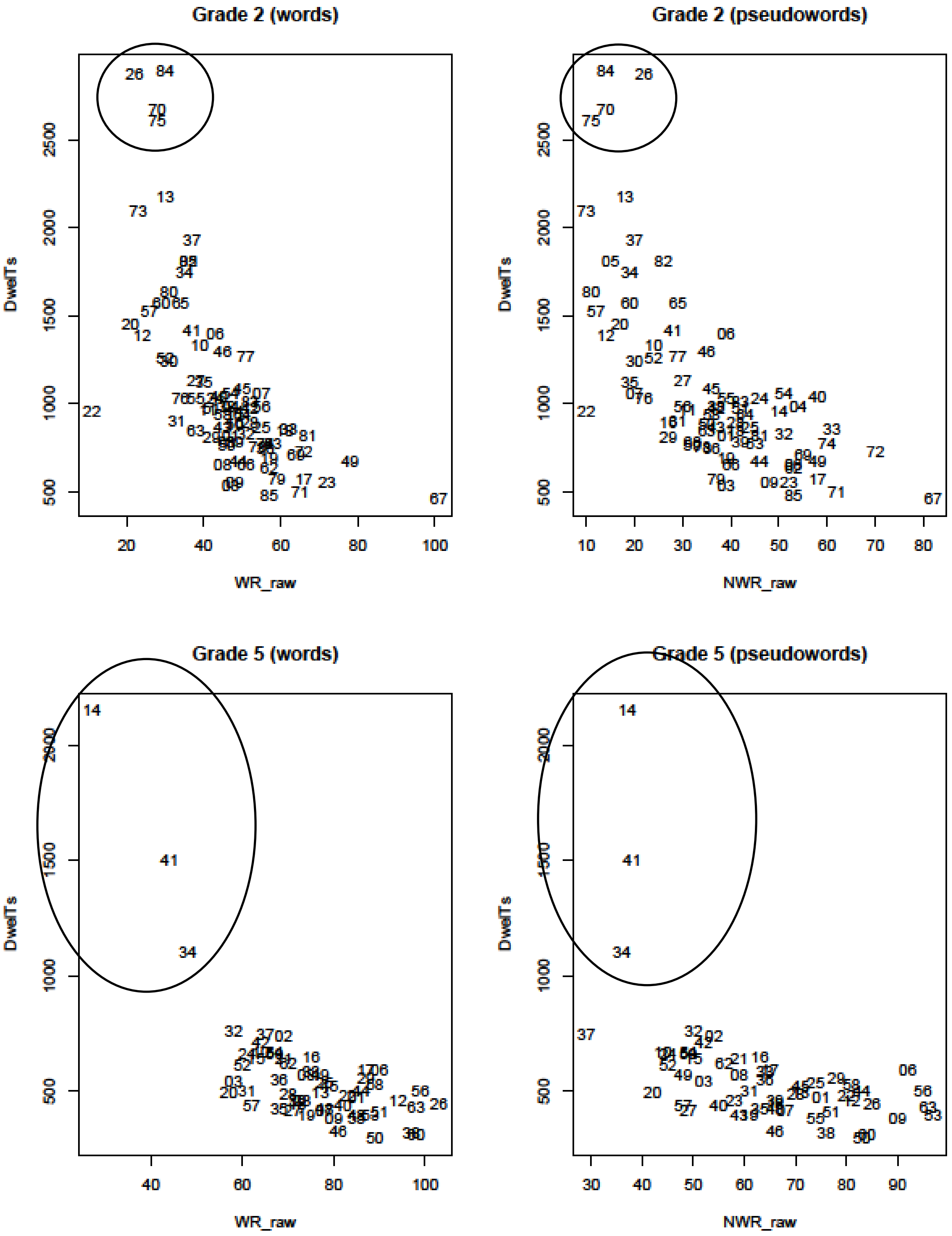

Figure S.4. Scatterplot of participants' total viewing times on the sentences and raw word reading scores split by grade. *Note.* The participants within the circle were considered outliers and excluded from further analyses.

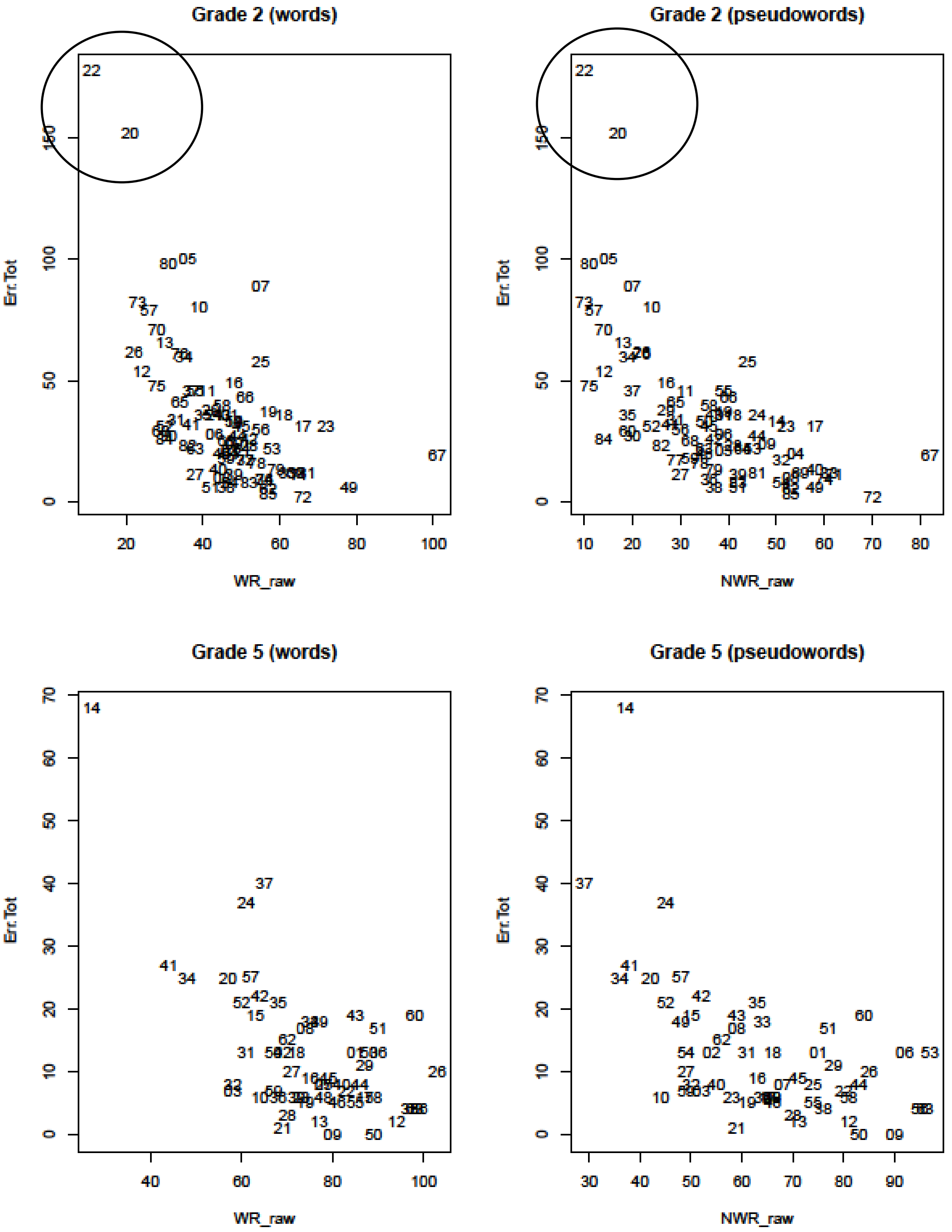

Figure S.5. Scatterplot of participants’ decoding errors on the sentences and raw word reading scores split by grade. *Note.* The participants within the circle were considered outliers and excluded from further analyses. Participant 14 in Grade 5 was already excluded based on its median dwell time, see Figure S.2.

Online Supplement A

Table S.6

Data clean up Online Measures

	Meeting		Removed[a]		Cumulative removed	
Variable	Count	%	Count	%	Count	%
Total viewing time < 80 ms	874	9.8	874	9.8	874	9.8
First-pass fixation duration < 80 ms	965	10.8				
First fixation duration < 80 ms	994	11.1				
Total viewing time > 6000 ms	44	0.5	44	0.4	918	10.2
First-pass fixation duration > 4000 ms	929	10.4				
First fixation duration > 3000 ms	880	9.8				
Decoding error on target	1189	13.3	1051	11.8	1969	22.0
Participant screening	576	6.4	347	3.8	2316	25.8

Note. [a] Number of removed fixations are less than matching because of overlap among criteria, i.e., some fixations have already been removed.
