# Supplementary material for: Lexicality effects on orthographic learning in beginning and advanced readers of Dutch: An eye-tracking study
Source: Q J Exp Psychol (Hove). 2021 Sep 28;75(6):1135–54. doi: 10.1177/17470218211047420 (PMC9016678; doi:10.1177/17470218211047420)
Supplement: sj-pdf-2-qjp-10.1177_17470218211047420 – Supplemental material for Lexicality effects on orthographic learning in beginning and advanced readers of Dutch: An eye-tracking study [file sj-pdf-2-qjp-10.1177_17470218211047420.pdf]

Orthographic learning: Offline measures analyses

Athanassios Protopapas

22 August 2021

Contents

1 Introduction **1**

2 Graphical display of data trends **2**

3 Orthographic choice **4**
3.1 Retrieve coefficients for model matrix 4
3.2 Fit model for lexicality and grade 5
3.3 Fit and compare reduced model 6
3.4 Model diagnostics 9
3.5 Effects graphs 13
3.6 Power considerations 14
3.7 Modeling the overall learning effect 15
3.8 Checking for effects of length 22

4 Spelling **37**
4.1 Fit model for lexicality and grade 37
4.2 Fit and compare reduced model 38
4.3 Model diagnostics 42
4.4 Effects graphs 45
4.5 Power considerations 47
4.6 Modeling the overall learning effect 47
4.7 Checking for effects of length 54

5 Final comments on observed effects and power **69**
5.1 Effects on orthographic learning 69
5.2 Statistical power 70

6 Session information **73**

1 Introduction

In this document we report analyses for offline outcome measures (orthographic choice and spelling) in the orthographic learning study of van Viersen et al. (manuscript submitted to the *Quarterly Journal of Experimental Psychology*)

```
library(lme4)
library(MASS)
library(effects)
library(DHARMa)
library(ggplot2)
library(nlme)
```

```
library(gmodels)
library(car)
```

```
load("som.Rda")
str(som)
```

```
## 'data.frame':    4063 obs. of  15 variables:
##  $ sID     : Factor w/ 127 levels "40010118","40030118",..: 1 1 1 1 1 1 1 1 1 1 ...
##  $ targetS : Factor w/ 32 levels "accu","beitel",..: 1 2 3 4 5 6 7 8 9 10 ...
##  $ spelitem: int  14 15 26 6 25 31 18 1 4 2 ...
##  $ reps    : Factor w/ 3 levels "0","2","6": 2 1 1 2 1 1 1 1 2 1 ...
##  $ spelaccu: int  0 0 1 0 0 0 0 1 0 1 ...
##  $ Nsyl2   : Factor w/ 2 levels "1","2": 2 2 2 1 2 2 1 1 1 1 ...
##  $ lex2    : Factor w/ 2 levels "p","w": 2 2 1 2 1 1 1 2 2 2 ...
##  $ orthitem: int  11 10 31 3 32 26 23 8 5 7 ...
##  $ orthaccu: int  1 1 1 1 0 0 1 1 1 1 ...
##  $ grade   : Factor w/ 2 levels "G2","G5": 1 1 1 1 1 1 1 1 1 1 ...
##  $ lex2C   : num  -0.5 -0.5 0.5 -0.5 0.5 0.5 0.5 -0.5 -0.5 -0.5 ...
##  $ Nsyl2C  : num  0.5 0.5 0.5 -0.5 0.5 0.5 -0.5 -0.5 -0.5 -0.5 ...
##  $ gradeC  : num  -0.5 -0.5 -0.5 -0.5 -0.5 -0.5 -0.5 -0.5 -0.5 -0.5 ...
##  $ reps2   : Factor w/ 3 levels "2","0","6": 1 2 2 1 2 2 2 2 1 2 ...
##  $ repsC   : num  NA -0.5 -0.5 NA -0.5 -0.5 -0.5 -0.5 NA -0.5 ...
```

There are two outcome measures, namely orthographic choice and spelling (binary variables `orthaccu` and `spelaccu`, respectively, coded per item/trial as 1=correct, 0=incorrect). Prior to these two tasks, children had been exposed to 0, 2, or 6 repetitions (variable `reps`) of each item (different combinations for different children). There are two groups of participants, namely children in Grades 2 and 5 (G2 vs. G5). There are two types of items, words (lexicality "w") and pseudowords (lexicality "p") (variable `lex2`). There are two word lengths, namely one or 2 syllables (variable `Nsyl2` with values 1 and 2). Each 2-level categorical predictor is coded into a numeric variable with values -0.5 and $+0.5$, indicated with a final letter C, to facilitate convergence and reduce the number of estimated parameters in the random structure. The extreme levels of exposure (0 vs. 6 repetitions) are also coded into a numeric variable (`repsC`) in the same way.

Considering all three levels of exposure, we want the models to test for successive differences, i.e., evidence for orthographic learning after 2 exposures ("early learning") or for difference between 2 and 6 exposures ("late learning"). We also want a grand-mean intercept so to be able to interpret coefficients as effects on differences. This is accomplished via `contr.sdif` from library `MASS`. However, to induce the random structure to also compute variances/covariances for the same contrast terms is not accomplished by default in `glmer`, so we use elements from the model matrix as shown by Kliegl (2014).

2 Graphical display of data trends

The following graph displays mean performance by aggregating data and calculating within-participants confidence intervals based on a multilevel model with unstructured covariance matrix following Baguley (2012) as explained here.

```
aggoitm <- aggregate(orthaccu~reps+lex2+grade+sID,som,mean,na.rm=T)
aggsitm <- aggregate(spelaccu~reps+lex2+grade+sID,som,mean,na.rm=T)
mlo.mod <- lme(orthaccu ~ 0 + grade:lex2:reps,
              random = list(sID = pdDiag(form = ~0 + grade), sID = pdBlocked(list( ~0+lex2, ~0+reps) ))
              data=aggoitm)
mls.mod <- lme(spelaccu ~ 0 + grade:lex2:reps,
               random = list(sID = pdDiag(form = ~0 + grade), sID = pdBlocked(list( ~0+lex2, ~0+reps) )
               data=aggsitm)
conf.level <- 0.95
```

```r
cio.mat <- ci(mlo.mod, confidence = conf.level)[, 2:3]
cis.mat <- ci(mls.mod, confidence = conf.level)[, 2:3]
tasks <- c("orthaccu","spelaccu")
aggdata <- reshape(direction="long",
                   data=merge(aggregate(orthaccu~reps+lex2+grade,aggoitm,mean,na.rm=T),
                              aggregate(spelaccu~reps+lex2+grade,aggsitm,mean,na.rm=T),
                   by=c("reps","lex2","grade")),
                   varying=tasks,
                   v.names="score",
                   timevar="task",times=tasks)
aggcidata <- cbind(aggdata,rbind(cio.mat,cis.mat))
names(aggcidata)[7:8] <- c("CIupper","CIlower")
```

```r
col1 <- "#4040FF"
col2 <- "#60A000"
pd <- position_dodge(0.1)
ggplot( aggcidata, aes(x=reps,y=score,group=grade)) +
  theme_bw() + # or theme_classic for no surrounding boxes
  ylim(0,1) + xlab("Repetition/exposure") + ylab("Proportion correct") +
  geom_line(aes(color=grade),size=1.2,position=pd) + scale_colour_manual(values=c(col1,col2)) +
  geom_errorbar(aes(ymin=CIlower,ymax=CIupper,colour=grade),width=0.15,position=pd,lwd=1.5) +
  geom_point(aes(color=grade),size=4,position=pd,fill="white",shape=21,stroke=3) +
  facet_grid(lex2~task, labeller=labeller(
    lex2=c("w" = "Words","p" = "Pseudowords"),
    task=c("orthaccu" = "Orthographic Choice","spelaccu" = "Spelling"))) +
  ggtitle("Orthographic choice") +
  ggtitle("") + labs(color="Grade") +
  theme(text=element_text(size=20)) +
  theme(strip.text=element_text(size=18)) +
  theme(legend.text=element_text(size=17)) +
  theme(axis.title.y = element_text(margin = margin(t = 0, r = 20, b = 0, l = 0))) +
  theme(axis.title.x = element_text(margin = margin(t = 15, r = 0, b = 0, l = 0))) +
  theme(legend.position=c(0.88,0.88),
        legend.background = element_rect(linetype = 1, size = 0.25, colour = 1))
```

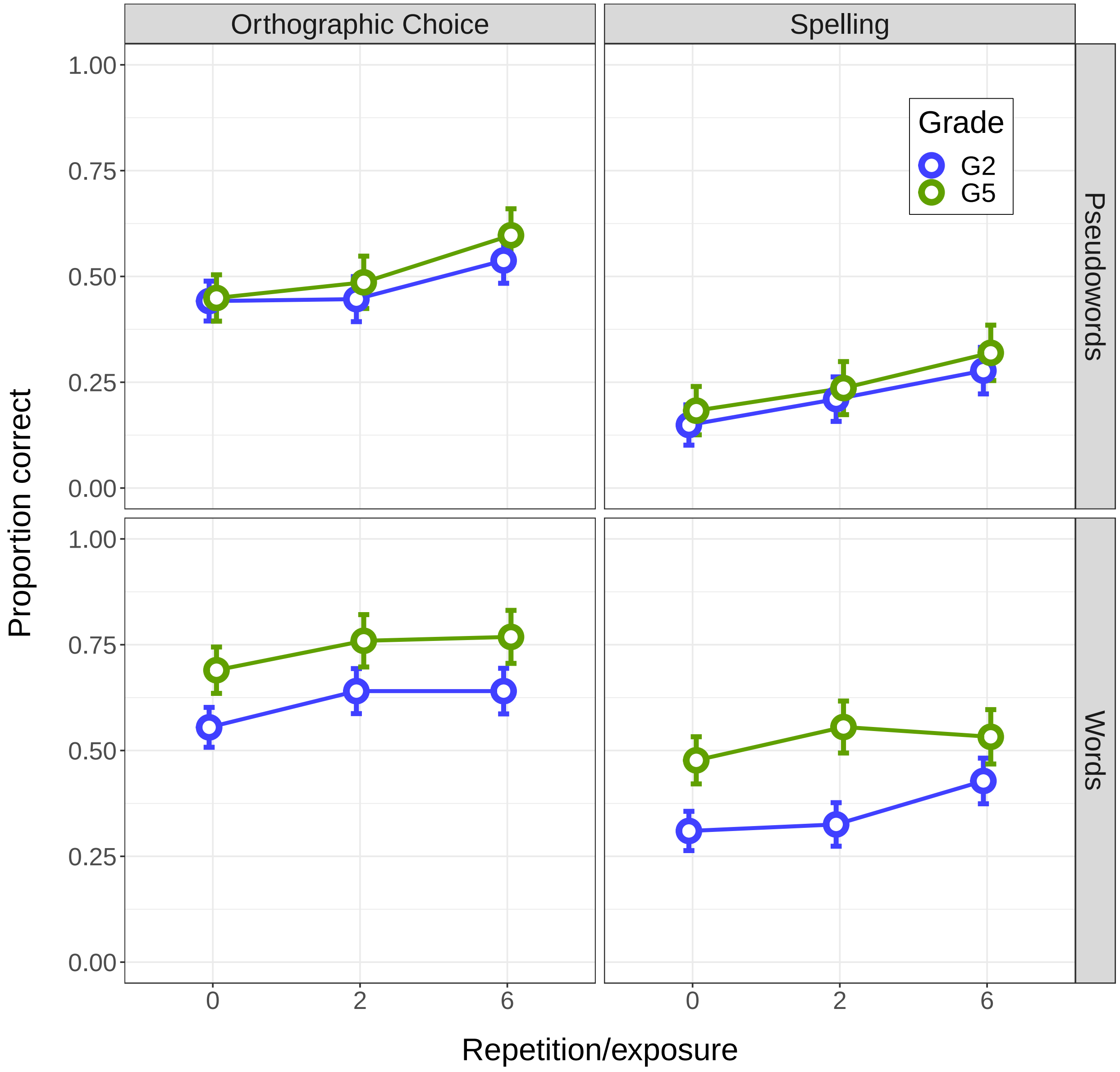

3 Orthographic choice

Orthographic choice accuracy per trial (item) is coded as 0 (incorrect) or 1 (correct).

3.1 Retrieve coefficients for model matrix

We first fit a model with minimal random structure to obtain the model matrix.

```
reps.contr.sdif <- contr.sdif(3)
colnames(reps.contr.sdif) <- c("2_0","6_2") # rename for legibility
contrasts( som$reps ) <- reps.contr.sdif
olfrm0 <- as.formula( "orthaccu ~ 1 + reps*lex2C*gradeC + (1|sID) + (1|targetS)" )
ol3c0 <- glmer( olfrm0, som, family=binomial, control=glmerControl(optCtrl=list(maxfun=1e5), optimizer =
```

```
## Warning in checkConv(attr(opt, "derivs"), opt$par, ctrl = control$checkConv, :
## Model failed to converge with max|grad| = 0.00398045 (tol = 0.002, component 1)
```

We then recover the columns associated with the simple successive differences

```
reps2_0 <- model.matrix(ol3c0)[,"reps2_0"] # 2 vs 0 reps
reps6_2 <- model.matrix(ol3c0)[,"reps6_2"] # 6 vs 2 reps
```

3.2 Fit model for lexicality and grade

Now we can specify the model with the full random structure including interactions between reps and each of the predictors. Note that grade only varies within item and lexicality only varies within participant.

```
olfrm1 <- as.formula( "orthaccu ~ 1 + reps*lex2C*gradeC + ((reps6_2+reps2_0)*lex2C|sID) + ((reps6_2+reps
ol3c1 <- glmer( olfrm1, som, family=binomial, control=glmerControl(optCtrl=list(maxfun=1e5), optimizer =
```

```
## boundary (singular) fit: see ?isSingular
```

```
print(summary(ol3c1),corr=F)
```

```
## Generalized linear mixed model fit by maximum likelihood (Laplace
##   Approximation) [glmerMod]
##  Family: binomial  ( logit )
## Formula: orthaccu ~ 1 + reps * lex2C * gradeC + ((reps6_2 + reps2_0) *
##     lex2C | sID) + ((reps6_2 + reps2_0) * gradeC | targetS)
##    Data: som
## Control: glmerControl(optCtrl = list(maxfun = 1e+05), optimizer = "nloptwrap")
##
##      AIC      BIC   logLik deviance df.resid
##   5002.4   5343.2  -2447.2   4894.4     4009
##
## Scaled residuals:
##     Min      1Q  Median      3Q     Max
## -5.4058 -0.8268  0.2941  0.7774  2.2556
##
## Random effects:
##  Groups  Name           Variance Std.Dev. Corr
##  sID     (Intercept)    0.03751  0.1937
##          reps6_2        0.21113  0.4595    0.42
##          reps2_0        0.04760  0.2182   -0.03 -0.27
##          lex2C          0.08383  0.2895   -0.58 -0.52  0.83
##          reps6_2:lex2C  0.55428  0.7445    0.71  0.84 -0.55 -0.88
##          reps2_0:lex2C  0.23969  0.4896   -0.57 -0.90  0.58  0.84 -0.98
##  targetS (Intercept)    0.72783  0.8531
##          reps6_2        0.18860  0.4343   -0.50
##          reps2_0        0.05526  0.2351   -0.33  0.39
##          gradeC         0.89988  0.9486    0.27  0.55  0.03
##          reps6_2:gradeC 0.54719  0.7397    0.10  0.04 -0.75 -0.11
##          reps2_0:gradeC 0.40810  0.6388   -0.65  0.82  0.81  0.29 -0.48
## Number of obs: 4063, groups:  sID, 127; targetS, 32
##
## Fixed effects:
##                       Estimate Std. Error z value Pr(>|z|)
## (Intercept)            0.50911    0.15827   3.217 0.001296 **
## reps2_0                0.20348    0.10753   1.892 0.058462 .
## reps6_2                0.24982    0.14519   1.721 0.085321 .
```

```
## lex2C                 -1.06128    0.31551  -3.364 0.000769 ***
## gradeC                 0.53525    0.19258   2.779 0.005446 **
## reps2_0:lex2C         -0.34080    0.21662  -1.573 0.115661
## reps6_2:lex2C          0.46342    0.28491   1.627 0.103833
## reps2_0:gradeC         0.06018    0.22757   0.264 0.791427
## reps6_2:gradeC         0.14450    0.27716   0.521 0.602111
## lex2C:gradeC          -0.72396    0.38170  -1.897 0.057870 .
## reps2_0:lex2C:gradeC  0.23184    0.45677   0.508 0.611751
## reps6_2:lex2C:gradeC -0.07258    0.54162  -0.134 0.893402
## ---
## Signif. codes:  0 '***' 0.001 '**' 0.01 '*' 0.05 '.' 0.1 ' ' 1
## optimizer (nloptwrap) convergence code: 0 (OK)
## boundary (singular) fit: see ?isSingular
```

The singular convergence indicates that the model is overparameterized, that is, there are too many parameters to estimate in the random structure in relation to the observed variability, but the model has converged and seems otherwise usable.

To examine the overall effect of factors with more than 2 levels (i.e., reps) we can use an ANOVA-style table (from library `car`):

```
Anova(ol3c1)
```

```
## Analysis of Deviance Table (Type II Wald chisquare tests)
##
## Response: orthaccu
##                        Chisq Df Pr(>Chisq)
## reps                 10.3810  2   0.005569 **
## lex2C                 4.9084  1   0.026726 *
## gradeC                6.6462  1   0.009937 **
## reps:lex2C            4.2325  2   0.120485
## reps:gradeC           0.8189  2   0.664024
## lex2C:gradeC          4.5180  1   0.033541 *
## reps:lex2C:gradeC     0.2940  2   0.863280
## ---
## Signif. codes:  0 '***' 0.001 '**' 0.01 '*' 0.05 '.' 0.1 ' ' 1
```

3.3 Fit and compare reduced model

Indeed the correlation parameters and some of the random effects variances are redundant, as removing them does not result in significantly inferior model fit.

We first remove correlations between random effects, the resulting model is no worse.

```
olfrm2 <- as.formula( "orthaccu ~ 1 + reps*lex2C*gradeC + ((reps6_2+reps2_0)*lex2C||sID) + ((reps6_2+re
ol3c2 <- glmer( olfrm2, som, family=binomial, control=glmerControl(optCtrl=list(maxfun=1e5), optimizer =
```

```
## boundary (singular) fit: see ?isSingular
```

```
print(summary(ol3c2),corr=F)
```

```
## Generalized linear mixed model fit by maximum likelihood (Laplace
##   Approximation) [glmerMod]
##  Family: binomial  ( logit )
## Formula: orthaccu ~ 1 + reps * lex2C * gradeC + ((reps6_2 + reps2_0) *
##     lex2C || sID) + ((reps6_2 + reps2_0) * gradeC || targetS)
##    Data: som
## Control: glmerControl(optCtrl = list(maxfun = 1e+05), optimizer = "nloptwrap")
```

```
##
##      AIC      BIC   logLik deviance df.resid
##   4968.5   5120.0  -2460.3   4920.5     4039
##
## Scaled residuals:
##     Min      1Q  Median      3Q     Max
## -5.4420 -0.8232  0.3026  0.7791  2.2174
##
## Random effects:
##  Groups    Name           Variance  Std.Dev.
##  sID       (Intercept)    3.427e-02 1.851e-01
##  sID.1     reps6_2        1.783e-01 4.223e-01
##  sID.2     reps2_0        3.152e-09 5.615e-05
##  sID.3     lex2C          6.877e-10 2.622e-05
##  sID.4     reps6_2:lex2C  8.229e-08 2.869e-04
##  sID.5     reps2_0:lex2C  0.000e+00 0.000e+00
##  targetS   (Intercept)    7.013e-01 8.375e-01
##  targetS.1 reps6_2        1.511e-01 3.888e-01
##  targetS.2 reps2_0        5.138e-02 2.267e-01
##  targetS.3 gradeC         7.549e-01 8.688e-01
##  targetS.4 reps6_2:gradeC 3.919e-01 6.260e-01
##  targetS.5 reps2_0:gradeC 7.162e-10 2.676e-05
## Number of obs: 4063, groups:  sID, 127; targetS, 32
##
## Fixed effects:
##                       Estimate Std. Error z value Pr(>|z|)
## (Intercept)           0.488102   0.154821   3.153 0.001618 **
## reps2_0               0.219573   0.097943   2.242 0.024972 *
## reps6_2               0.267192   0.131676   2.029 0.042441 *
## lex2C                -1.028309   0.307831  -3.341 0.000836 ***
## gradeC                0.487862   0.177336   2.751 0.005940 **
## reps2_0:lex2C        -0.337529   0.195880  -1.723 0.084863 .
## reps6_2:lex2C         0.389920   0.251675   1.549 0.121310
## reps2_0:gradeC        0.114426   0.177856   0.643 0.519990
## reps6_2:gradeC        0.116877   0.249577   0.468 0.639569
## lex2C:gradeC         -0.654092   0.348296  -1.878 0.060384 .
## reps2_0:lex2C:gradeC  0.097637   0.355712   0.274 0.783712
## reps6_2:lex2C:gradeC  0.003684   0.475710   0.008 0.993821
## ---
## Signif. codes:  0 '***' 0.001 '**' 0.01 '*' 0.05 '.' 0.1 ' ' 1
## optimizer (nloptwrap) convergence code: 0 (OK)
## boundary (singular) fit: see ?isSingular
```

```
anova(ol3c1,ol3c2)
```

```
## Data: som
## Models:
## ol3c2: orthaccu ~ 1 + reps * lex2C * gradeC + ((reps6_2 + reps2_0) * lex2C || sID) + ((reps6_2 + reps
## ol3c1: orthaccu ~ 1 + reps * lex2C * gradeC + ((reps6_2 + reps2_0) * lex2C | sID) + ((reps6_2 + reps2
##       npar    AIC    BIC  logLik deviance  Chisq Df Pr(>Chisq)
## ol3c2   24 4968.5 5120.0 -2460.3   4920.5
## ol3c1   54 5002.4 5343.2 -2447.2   4894.4 26.108 30     0.6696
```

We then iteratively remove random effects that are near zero, starting with higher-order ones. This eventually results in a model with nonsingular fit that is no worse than that of the original model (with full random

structure). Convergence is achieved using a different optimizer.

```
olfrm3 <- as.formula( "orthaccu ~ 1 + reps*lex2C*gradeC + (reps6_2||sID) + (reps2_0+reps6_2+gradeC||targ
ol3c3 <- glmer( olfrm3, som, family=binomial, control=glmerControl(optCtrl=list(maxfun=1e5), optimizer =
print(summary(ol3c3),corr=F)
```

```
## Generalized linear mixed model fit by maximum likelihood (Laplace
##   Approximation) [glmerMod]
##  Family: binomial  ( logit )
## Formula: orthaccu ~ 1 + reps * lex2C * gradeC + (reps6_2 || sID) + (reps2_0 +
##     reps6_2 + gradeC || targetS)
##    Data: som
## Control: glmerControl(optCtrl = list(maxfun = 1e+05), optimizer = "bobyqa")
##
##      AIC      BIC   logLik deviance df.resid
##   4959.1   5072.6  -2461.5   4923.1     4045
##
## Scaled residuals:
##     Min      1Q  Median      3Q     Max
## -5.4548 -0.8205  0.3043  0.7788  2.1308
##
## Random effects:
##  Groups    Name        Variance Std.Dev.
##  sID       (Intercept) 0.03327  0.1824
##  sID.1     reps6_2     0.17604  0.4196
##  targetS   (Intercept) 0.69803  0.8355
##  targetS.1 reps2_0     0.05251  0.2292
##  targetS.2 reps6_2     0.14876  0.3857
##  targetS.3 gradeC      0.75922  0.8713
## Number of obs: 4063, groups:  sID, 127; targetS, 32
##
## Fixed effects:
##                        Estimate Std. Error z value Pr(>|z|)
## (Intercept)             0.48616    0.15443   3.148  0.00164 **
## reps2_0                 0.21955    0.09808   2.238  0.02519 *
## reps6_2                 0.26410    0.13077   2.020  0.04343 *
## lex2C                  -1.02539    0.30708  -3.339  0.00084 ***
## gradeC                  0.48736    0.17750   2.746  0.00604 **
## reps2_0:lex2C          -0.33710    0.19614  -1.719  0.08568 .
## reps6_2:lex2C           0.38908    0.25002   1.556  0.11966
## reps2_0:gradeC          0.11517    0.17768   0.648  0.51688
## reps6_2:gradeC          0.12052    0.22107   0.545  0.58563
## lex2C:gradeC           -0.64708    0.34879  -1.855  0.06356 .
## reps2_0:lex2C:gradeC    0.09637    0.35537   0.271  0.78625
## reps6_2:lex2C:gradeC    0.02726    0.41569   0.066  0.94771
## ---
## Signif. codes:  0 '***' 0.001 '**' 0.01 '*' 0.05 '.' 0.1 ' ' 1
```

```
anova(ol3c1,ol3c3)
```

```
## Data: som
## Models:
## ol3c3: orthaccu ~ 1 + reps * lex2C * gradeC + (reps6_2 || sID) + (reps2_0 + reps6_2 + gradeC || targe
## ol3c1: orthaccu ~ 1 + reps * lex2C * gradeC + ((reps6_2 + reps2_0) * lex2C | sID) + ((reps6_2 + reps
##       npar    AIC    BIC  logLik deviance  Chisq Df Pr(>Chisq)
```

```
## ol3c3   18 4959.1 5072.6 -2461.5   4923.1
## ol3c1   54 5002.4 5343.2 -2447.2   4894.4 28.615 36     0.8046
```

Removing random effects parameters also reduces the estimated standard error for the fixed effects, which means the model is less conservative, leading to higher power and possibly an inflated Type-I error rate. Indeed, some effects that did not reach significance in the model with a full random structure are significant in the model with reduced random structure, such as the overall learning effect (comparisons between 0 and 2 and between 2 and 6 exposures).

Here is the Anova table for the reduced model:

```
Anova(ol3c3)
```

```
## Analysis of Deviance Table (Type II Wald chisquare tests)
##
## Response: orthaccu
##                      Chisq Df Pr(>Chisq)
## reps               14.4945  2  0.0007121 ***
## lex2C              10.3184  1  0.0013171 **
## gradeC              6.4421  1  0.0111448 *
## reps:lex2C          4.0132  2  0.1344425
## reps:gradeC         1.6805  2  0.4315946
## lex2C:gradeC        3.7543  1  0.0526721 .
## reps:lex2C:gradeC   0.1470  2  0.9291243
## ---
## Signif. codes:  0 '***' 0.001 '**' 0.01 '*' 0.05 '.' 0.1 ' ' 1
```

And here are the estimates of the full and reduced model, side by side for comparability: (full model on the left, and reduced model on the right)

```
cbind(round(coef(summary(ol3c1)),3),round(coef(summary(ol3c3)),3)) -> cb
colnames(cb) <- rep(c("Estim","Std.Err","z","p"),2)
cb
```

```
##                        Estim Std.Err      z     p  Estim Std.Err      z     p
## (Intercept)            0.509   0.158  3.217 0.001  0.486   0.154  3.148 0.002
## reps2_0                0.203   0.108  1.892 0.058  0.220   0.098  2.238 0.025
## reps6_2                0.250   0.145  1.721 0.085  0.264   0.131  2.020 0.043
## lex2C                 -1.061   0.316 -3.364 0.001 -1.025   0.307 -3.339 0.001
## gradeC                 0.535   0.193  2.779 0.005  0.487   0.177  2.746 0.006
## reps2_0:lex2C         -0.341   0.217 -1.573 0.116 -0.337   0.196 -1.719 0.086
## reps6_2:lex2C          0.463   0.285  1.627 0.104  0.389   0.250  1.556 0.120
## reps2_0:gradeC         0.060   0.228  0.264 0.791  0.115   0.178  0.648 0.517
## reps6_2:gradeC         0.145   0.277  0.521 0.602  0.121   0.221  0.545 0.586
## lex2C:gradeC          -0.724   0.382 -1.897 0.058 -0.647   0.349 -1.855 0.064
## reps2_0:lex2C:gradeC  0.232   0.457  0.508 0.612  0.096   0.355  0.271 0.786
## reps6_2:lex2C:gradeC -0.073   0.542 -0.134 0.893  0.027   0.416  0.066 0.948
```

Nevertheless, none of the interactions attain significance. This means that there is no evidence that either grade or lexicality affects the orthographic learning effect very much.

3.4 Model diagnostics

Residual-based diagnostics for fitted GLMMs can be produced using library DHARMa. We first obtain a simulation-based residual distribution.

```
so <- simulateResiduals(fittedModel=ol3c3,plot=F)
```

We can then directly test for overdispersion, uniformity etc.

```r
testUniformity(so)
```

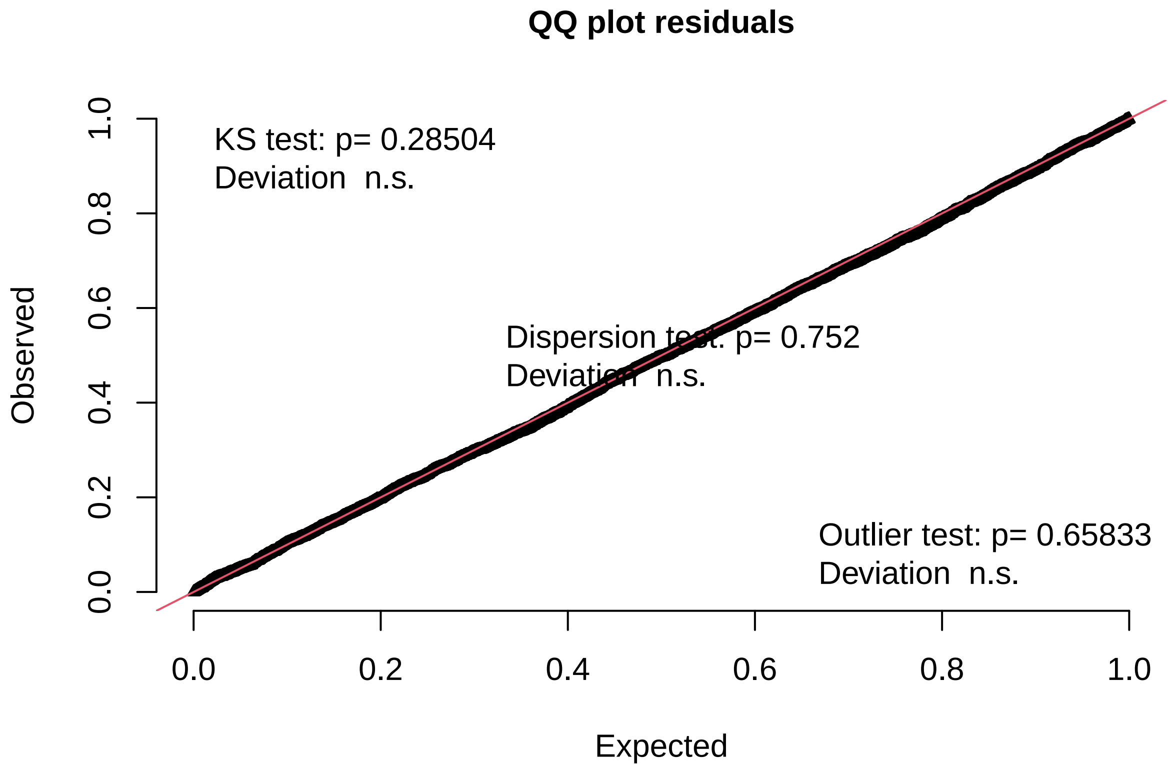


```
##
##  One-sample Kolmogorov-Smirnov test
##
## data:  simulationOutput$scaledResiduals
## D = 0.015473, p-value = 0.285
## alternative hypothesis: two-sided
```

```r
testOutliers(so)
```

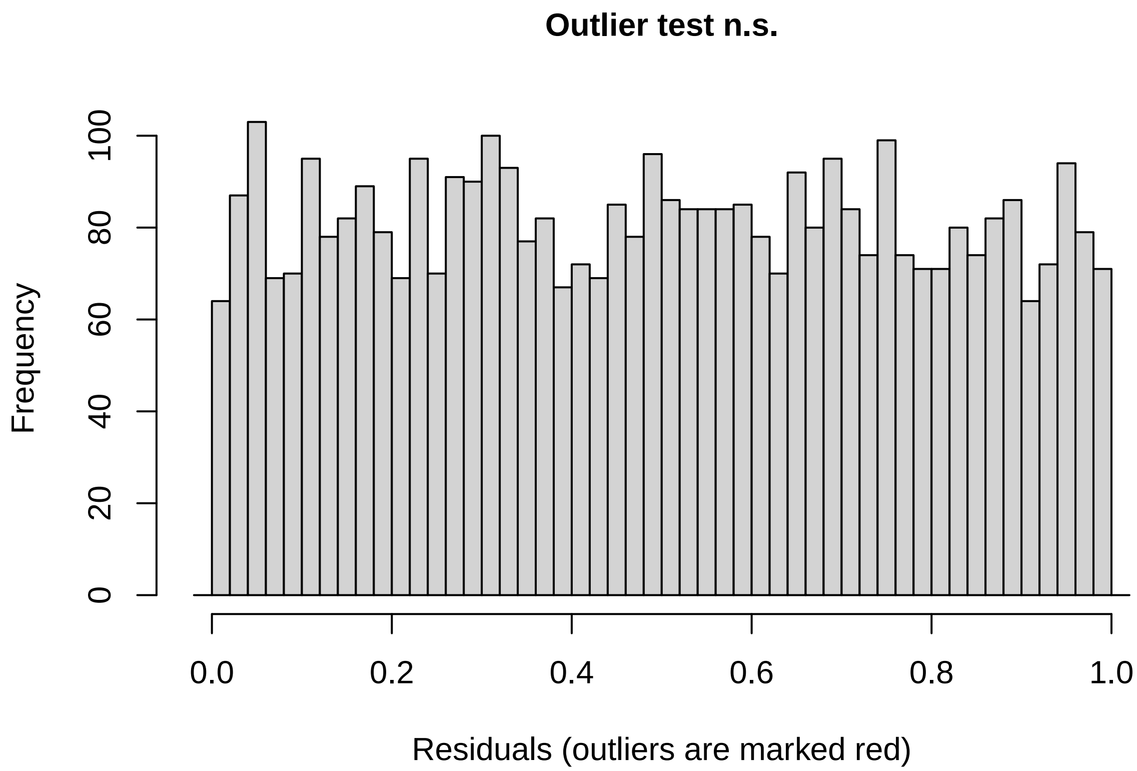


```
##
##  DHARMa outlier test based on exact binomial test with approximate
##  expectations
##
## data:  so
## outliers at both margin(s) = 29, observations = 4063, p-value = 0.6583
## alternative hypothesis: true probability of success is not equal to 0.007968127
## 95 percent confidence interval:
##  0.004785211 0.010234793
## sample estimates:
## frequency of outliers (expected: 0.00796812749003984 )
##                                            0.007137583
```

```
testDispersion(so)
```

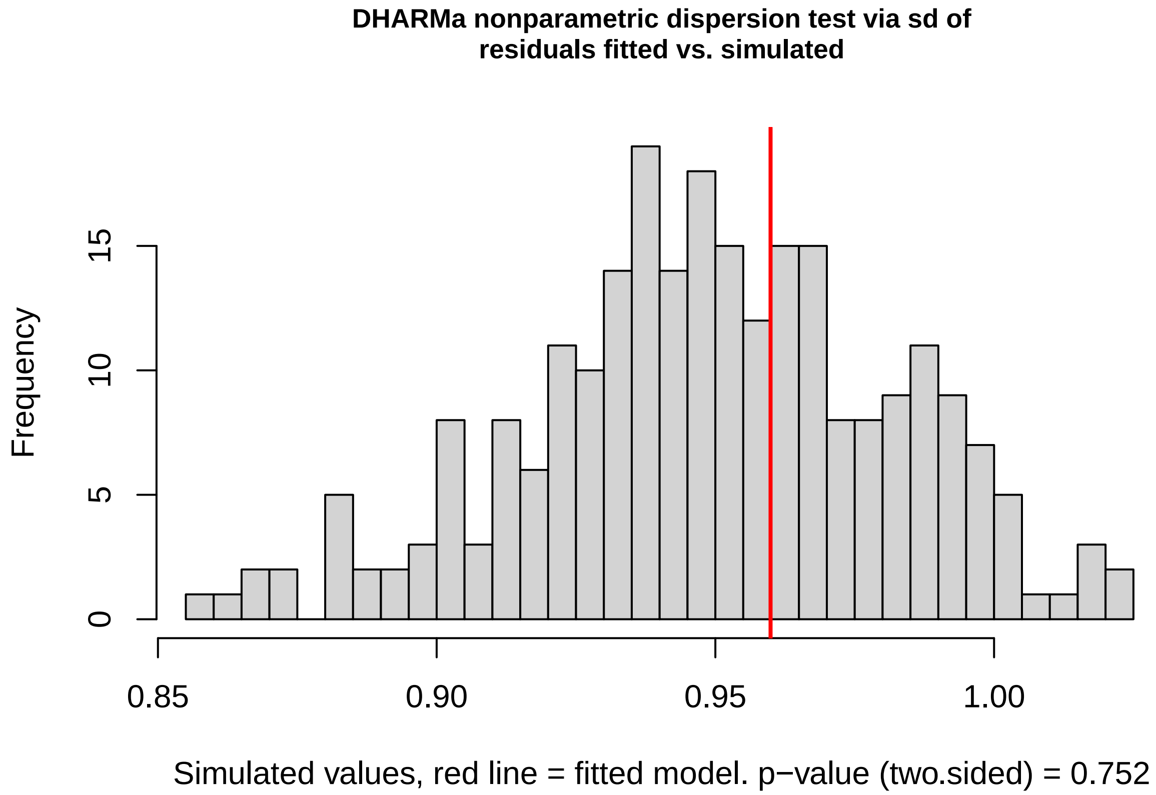


```
##
##  DHARMa nonparametric dispersion test via sd of residuals fitted vs.
##  simulated
##
## data:  simulationOutput
## dispersion = 1.0116, p-value = 0.752
## alternative hypothesis: two.sided
```

```
testZeroInflation(so)
```

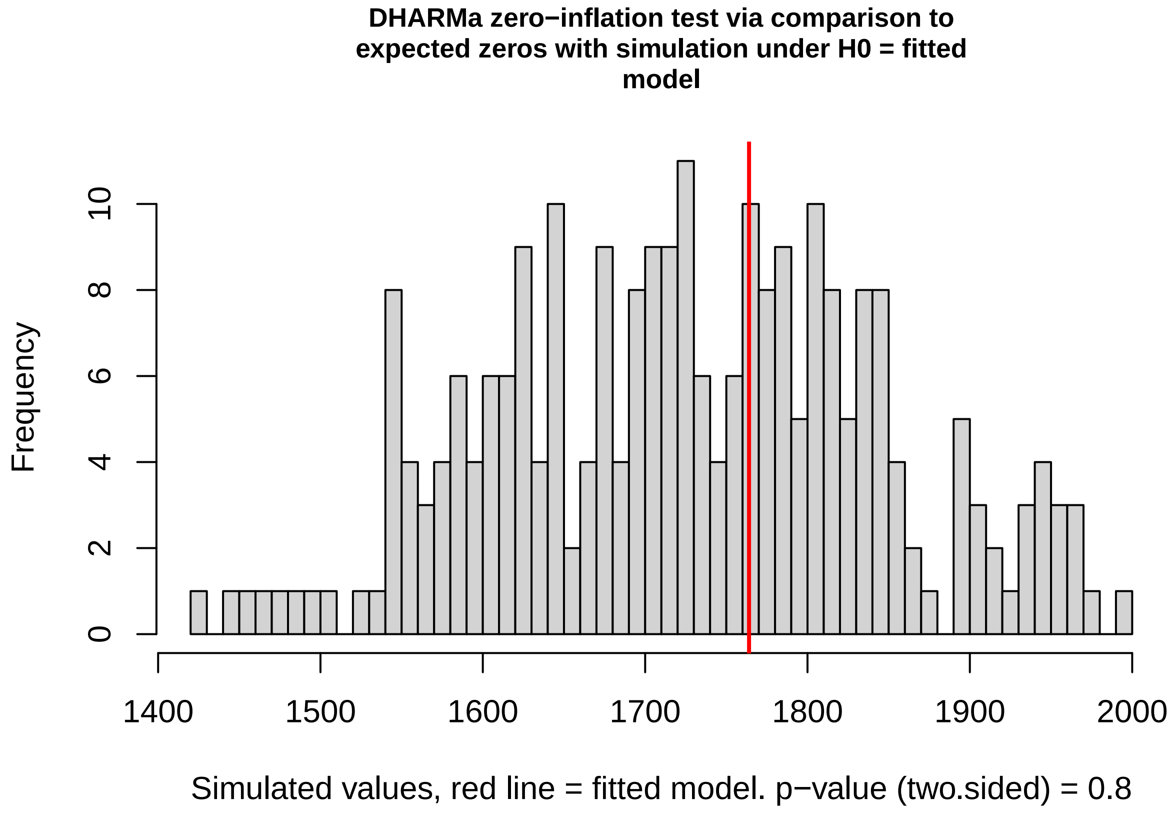


```
##
##  DHARMa zero-inflation test via comparison to expected zeros with
##  simulation under H0 = fitted model
##
## data:  simulationOutput
## ratioObsSim = 1.0217, p-value = 0.8
## alternative hypothesis: two.sided
```

These graphs and tests do not suggest any problems (indeed they look excellent).

3.5 Effects graphs

These are the effects from the full model.

Grades (rows) are: Grade 2 = -0.5, Grade 5 = $+0.5$.

Lexicality levels (columns) are: words = -0.5, pseudowords = $+0.5$.

```
plot(allEffects(ol3c1,xlevels=list(lex2C=c(-0.5,0.5),gradeC=c(-0.5,0.5))))
```

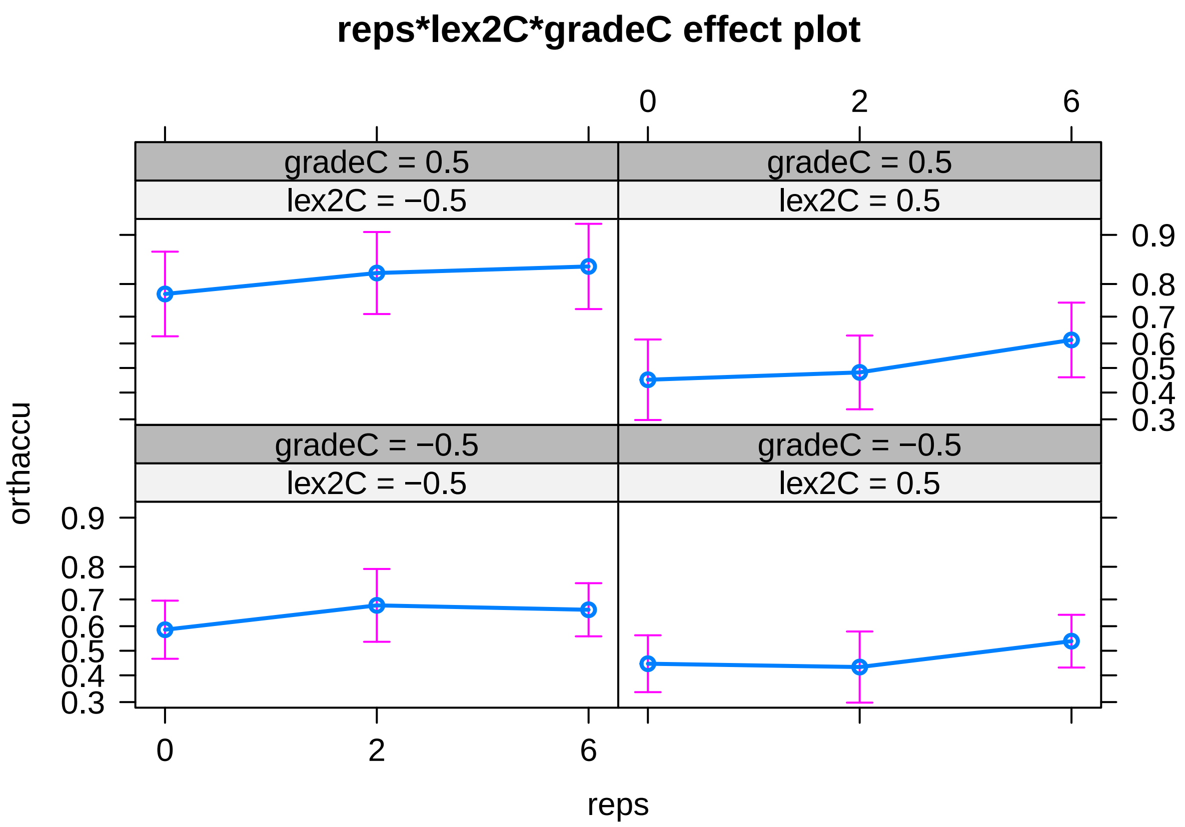

Consistent with the effect estimates, there is a small but clear and consistent learning effect (the upward slope of the lines, from 0 through 2 to 6 exposures), an effect of lexicality (higher accuracy for words, on the left column), and an effect of grade (higher accuracy by 5th graders, on the top row). The apparent slight trend for higher late than early learning for words (increasing slope for the 2-6 part on the right column but not on the left) corresponds to the non-significant interaction of lexicality with late learning (`reps6_2:lex2C`).

3.6 Power considerations

Based on the model with the full random structure, we see that the standard errors of the estimates associated with the learning effect are around .125 for the successive differences (reps2_0 and reps6_2), around .250 for their two-way interactions (with lexicality and grade), and around .500 for the three-way interactions.

Given that there is 50% power to detect an effect of about 2 SE, and 85% power to detect an effect of about 3 SE, this means that we would have sufficient power to detect a three-way interaction if its effect estimate was 1.5 or more. This is about 50% greater than the estimated main effect of lexicality. In terms of accuracy proportions, and given a grand-mean intercept of about 0.5 (hence average accuracy of 0.62), this means that the 1.5 three-way interaction effect would bring accuracy to 0.88 or 0.27, which seems like an unreasonable expectation. Thus we can conclude that we do not have sufficient power to detect a realistic three-way interaction.

However, the situation is somewhat better for two-way interactions. Turning to the well-fitting (and nonsingularly fitting) model, where standard errors for two-way interactions with number of exposures are around 0.2, this means we have 85% power to detect effects of magnitude 0.6 or greater. In terms of accuracy, and with reference to the same intercept (average accuracy 0.62), this means that a detectible two-way interaction effect should bring accuracy to 0.75 or 0.48. This seems like a not entirely unreasonable expectation. Thus, we can conclude that we have power to detect relatively large interaction effects that involve successive differences in number of exposures.

3.7 Modeling the overall learning effect

A more powerful approach to detecting interactions involves discarding the 2-exposures condition and modeling the total learning effect, that is, the difference between zero and six exposures. This is necessarily a larger effect than either of the intermediate ones modeled above, so it provides a higher-magnitude baseline over which interactions may be detected.

```
olfrmt <- as.formula( "orthaccu ~ 1 + repsC*lex2C*gradeC + (repsC*lex2C|sID) + (repsC*gradeC|targetS)" 
ol3ct <- glmer( olfrmt, som, family=binomial, control=glmerControl(optCtrl=list(maxfun=1e5), optimizer 
```

```
## boundary (singular) fit: see ?isSingular
```

```
print(summary(ol3ct),corr=F)
```

```
## Generalized linear mixed model fit by maximum likelihood (Laplace
##   Approximation) [glmerMod]
##  Family: binomial  ( logit )
## Formula: orthaccu ~ 1 + repsC * lex2C * gradeC + (repsC * lex2C | sID) +
##     (repsC * gradeC | targetS)
##    Data: som
## Control: glmerControl(optCtrl = list(maxfun = 1e+05), optimizer = "bobyqa")
##
##      AIC      BIC   logLik deviance df.resid
##   3786.0   3954.6  -1865.0   3730.0     3020
##
## Scaled residuals:
##     Min      1Q  Median      3Q     Max
## -4.7588 -0.8437  0.3455  0.8062  2.1753
##
## Random effects:
##  Groups  Name         Variance Std.Dev. Corr
##  sID     (Intercept)  0.05673  0.2382
##          repsC        0.19635  0.4431    0.63
##          lex2C        0.01110  0.1053   -0.02  0.76
##          repsC:lex2C  0.04656  0.2158    0.98  0.47 -0.21
##  targetS (Intercept)  0.66351  0.8146
##          repsC        0.26903  0.5187   -0.53
##          gradeC       0.79972  0.8943    0.41  0.27
##          repsC:gradeC 0.44394  0.6663   -0.43  0.71  0.19
## Number of obs: 3048, groups:  sID, 127; targetS, 32
##
## Fixed effects:
##                    Estimate Std. Error z value Pr(>|z|)
## (Intercept)          0.5001     0.1542   3.242  0.00119 **
## repsC                0.4528     0.1411   3.210  0.00133 **
## lex2C               -0.9062     0.3052  -2.970  0.00298 **
## gradeC               0.5429     0.1920   2.828  0.00468 **
## repsC:lex2C          0.1192     0.2692   0.443  0.65785
## repsC:gradeC         0.1947     0.2430   0.801  0.42291
## lex2C:gradeC        -0.7432     0.3733  -1.991  0.04653 *
## repsC:lex2C:gradeC   0.1736     0.4542   0.382  0.70229
## ---
## Signif. codes:  0 '***' 0.001 '**' 0.01 '*' 0.05 '.' 0.1 ' ' 1
## optimizer (bobyqa) convergence code: 0 (OK)
## boundary (singular) fit: see ?isSingular
```

Neither grade nor lexicality interacts significantly with exposure. But this fit is also singular, which suggests that the random structure has been overparameterized. So we refit after forcing the random effects to be uncorrelated.

```
olfrmt2 <- as.formula( "orthaccu ~ 1 + repsC*lex2C*gradeC + (repsC*lex2C||sID) + (repsC*gradeC||targetS
ol3ct2 <- glmer( olfrmt2, som, family=binomial, control=glmerControl(optCtrl=list(maxfun=1e5), optimize
```

```
## boundary (singular) fit: see ?isSingular
```

```
print(summary(ol3ct2),corr=F)
```

```
## Generalized linear mixed model fit by maximum likelihood (Laplace
##   Approximation) [glmerMod]
##  Family: binomial  ( logit )
## Formula: orthaccu ~ 1 + repsC * lex2C * gradeC + (repsC * lex2C || sID) +
##     (repsC * gradeC || targetS)
##    Data: som
## Control: glmerControl(optCtrl = list(maxfun = 1e+05), optimizer = "bobyqa")
##
##      AIC      BIC   logLik deviance df.resid
##   3776.3   3872.7  -1872.2   3744.3     3032
##
## Scaled residuals:
##     Min      1Q  Median      3Q     Max
## -3.6498 -0.8345  0.3350  0.8060  2.2597
##
## Random effects:
##  Groups    Name         Variance Std.Dev.
##  sID       (Intercept)  0.03035  0.1742
##  sID.1     repsC        0.10060  0.3172
##  sID.2     lex2C        0.00000  0.0000
##  sID.3     repsC:lex2C  0.00000  0.0000
##  targetS   (Intercept)  0.64466  0.8029
##  targetS.1 repsC        0.22435  0.4737
##  targetS.2 gradeC       0.65687  0.8105
##  targetS.3 repsC:gradeC 0.31808  0.5640
## Number of obs: 3048, groups:  sID, 127; targetS, 32
##
## Fixed effects:
##                     Estimate Std. Error z value Pr(>|z|)
## (Intercept)          0.47851    0.15059   3.178 0.001485 **
## repsC                0.47580    0.12700   3.747 0.000179 ***
## lex2C               -0.88195    0.29945  -2.945 0.003227 **
## gradeC               0.48273    0.17385   2.777 0.005492 **
## repsC:lex2C          0.07189    0.24719   0.291 0.771167
## repsC:gradeC         0.20066    0.21408   0.937 0.348610
## lex2C:gradeC        -0.64602    0.34176  -1.890 0.058725 .
## repsC:lex2C:gradeC   0.12597    0.41245   0.305 0.760055
## ---
## Signif. codes:  0 '***' 0.001 '**' 0.01 '*' 0.05 '.' 0.1 ' ' 1
## optimizer (bobyqa) convergence code: 0 (OK)
## boundary (singular) fit: see ?isSingular
```

```
anova(ol3ct,ol3ct2)
```

```
## Data: som
```

```
## Models:
## ol3ct2: orthaccu ~ 1 + repsC * lex2C * gradeC + (repsC * lex2C || sID) + (repsC * gradeC || targetS)
## ol3ct: orthaccu ~ 1 + repsC * lex2C * gradeC + (repsC * lex2C | sID) + (repsC * gradeC | targetS)
##        npar    AIC    BIC  logLik deviance  Chisq Df Pr(>Chisq)
## ol3ct2   16 3776.3 3872.7 -1872.2   3744.3
## ol3ct    28 3786.0 3954.6 -1865.0   3730.0 14.323 12     0.2805
```

Still singular fit, so we remove the negligible random effects.

```
olfrmt3 <- as.formula( "orthaccu ~ 1 + repsC*lex2C*gradeC + (repsC||sID) + (repsC*gradeC||targetS)" )
ol3ct3 <- glmer( olfrmt3, som, family=binomial, control=glmerControl(optCtrl=list(maxfun=1e5), optimize
print(summary(ol3ct3),corr=F)
```

```
## Generalized linear mixed model fit by maximum likelihood (Laplace
##   Approximation) [glmerMod]
##  Family: binomial  ( logit )
## Formula: orthaccu ~ 1 + repsC * lex2C * gradeC + (repsC || sID) + (repsC *
##     gradeC || targetS)
##    Data: som
## Control: glmerControl(optCtrl = list(maxfun = 1e+05), optimizer = "bobyqa")
##
##      AIC      BIC   logLik deviance df.resid
##   3772.3   3856.6  -1872.2   3744.3     3034
##
## Scaled residuals:
##     Min      1Q  Median      3Q     Max
## -3.6498 -0.8345  0.3350  0.8060  2.2597
##
## Random effects:
##  Groups    Name         Variance Std.Dev.
##  sID       (Intercept)  0.03036  0.1742
##  sID.1     repsC        0.10061  0.3172
##  targetS   (Intercept)  0.64466  0.8029
##  targetS.1 repsC        0.22435  0.4737
##  targetS.2 gradeC       0.65687  0.8105
##  targetS.3 repsC:gradeC 0.31808  0.5640
## Number of obs: 3048, groups:  sID, 127; targetS, 32
##
## Fixed effects:
##                    Estimate Std. Error z value Pr(>|z|)
## (Intercept)         0.47851    0.15059   3.178 0.001485 **
## repsC               0.47580    0.12700   3.746 0.000179 ***
## lex2C              -0.88196    0.29945  -2.945 0.003227 **
## gradeC              0.48273    0.17385   2.777 0.005492 **
## repsC:lex2C         0.07189    0.24718   0.291 0.771162
## repsC:gradeC        0.20066    0.21409   0.937 0.348618
## lex2C:gradeC       -0.64602    0.34178  -1.890 0.058735 .
## repsC:lex2C:gradeC  0.12597    0.41245   0.305 0.760042
## ---
## Signif. codes:  0 '***' 0.001 '**' 0.01 '*' 0.05 '.' 0.1 ' ' 1
```

```
anova(ol3ct,ol3ct3)
```

```
## Data: som
## Models:
## ol3ct3: orthaccu ~ 1 + repsC * lex2C * gradeC + (repsC || sID) + (repsC * gradeC || targetS)
```

```
## ol3ct: orthaccu ~ 1 + repsC * lex2C * gradeC + (repsC * lex2C | sID) + (repsC * gradeC | targetS)
##        npar    AIC    BIC  logLik deviance  Chisq Df Pr(>Chisq)
## ol3ct3   14 3772.3 3856.6 -1872.2   3744.3
## ol3ct    28 3786.0 3954.6 -1865.0   3730.0 14.323 14     0.4259
```

The latter model seems to have converged fine, and the fit is no worse than that of the model with the full random structure. Note that the standard errors for the interactions are—as expected—similar in magnitude as in the model with all three exposure conditions, thus the power is the same in terms of how large an interaction effect we can expect to be able to detect. Still, the estimated magnitudes of the two-way interactions are quite low, reinforcing the conclusion that grade and lexicality do not affect orthographic learning rate very much.

This does not imply that grade and lexicality do not affect orthographic learning. But it does suggest that their effects are at best modest in comparison with the overall learning rate.

Here are the model effects:

```
plot(allEffects(ol3ct3,xlevels=list(repsC=c(-0.5,0.5),lex2C=c(-0.5,0.5),gradeC=c(-0.5,0.5))))
```

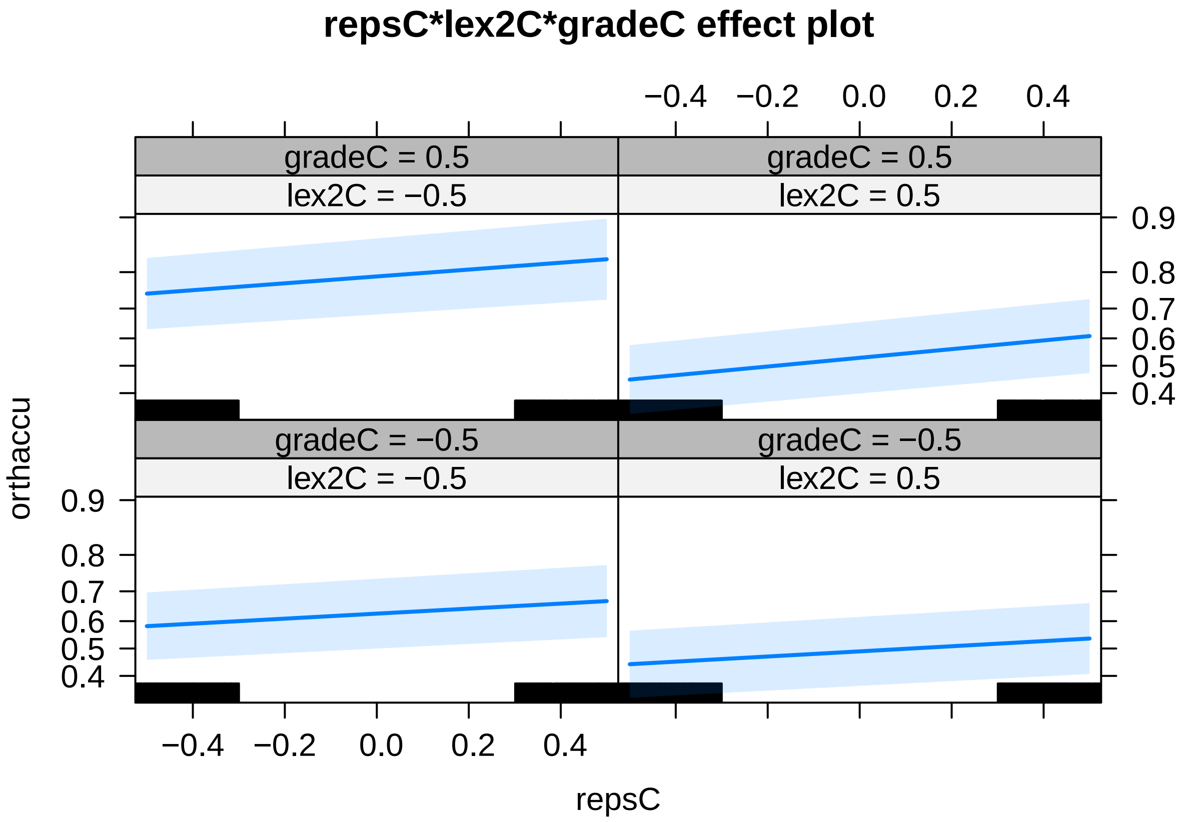

Reminder: Grade (rows), Grade 2 = –0.5, Grade 5 = +0.5; Lexicality (columns), words = –0.5, pseudowords = +0.5.

And here are the fit diagnostics:

```
sot <- simulateResiduals(fittedModel=ol3ct3,plot=F)
testUniformity(sot)
```

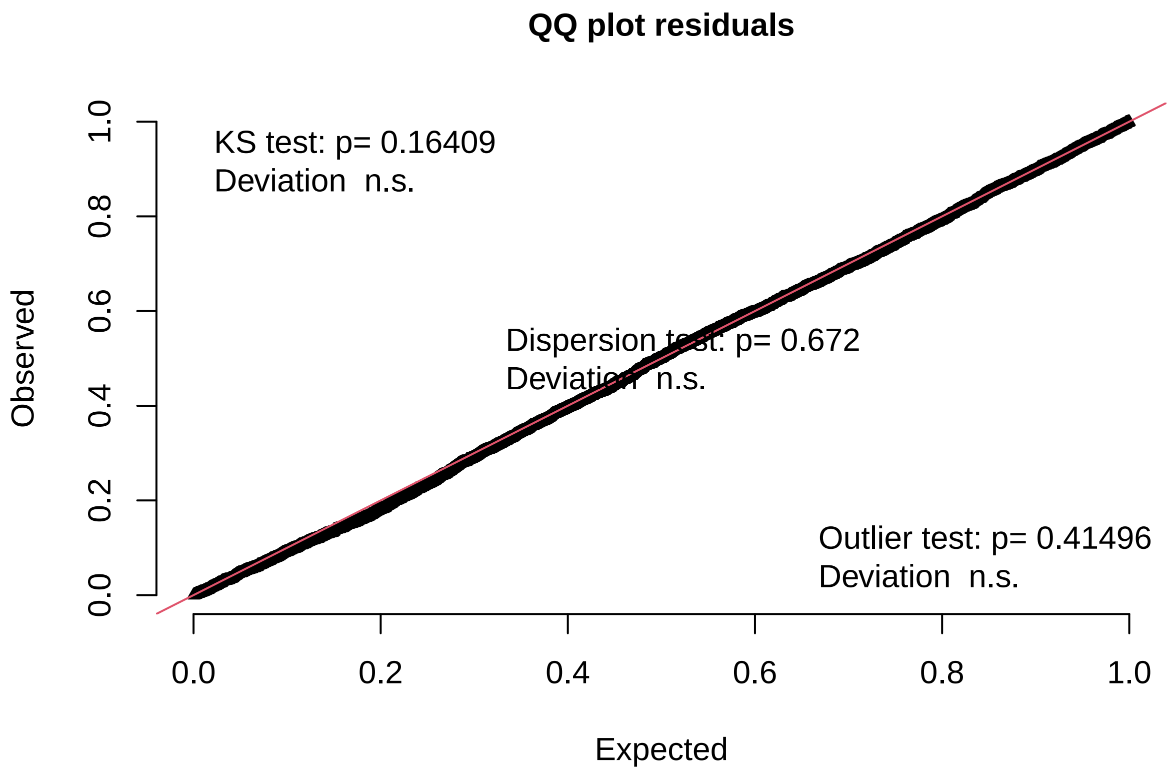


```
##
##  One-sample Kolmogorov-Smirnov test
##
## data:  simulationOutput$scaledResiduals
## D = 0.020251, p-value = 0.1641
## alternative hypothesis: two-sided
```

```
testOutliers(sot)
```

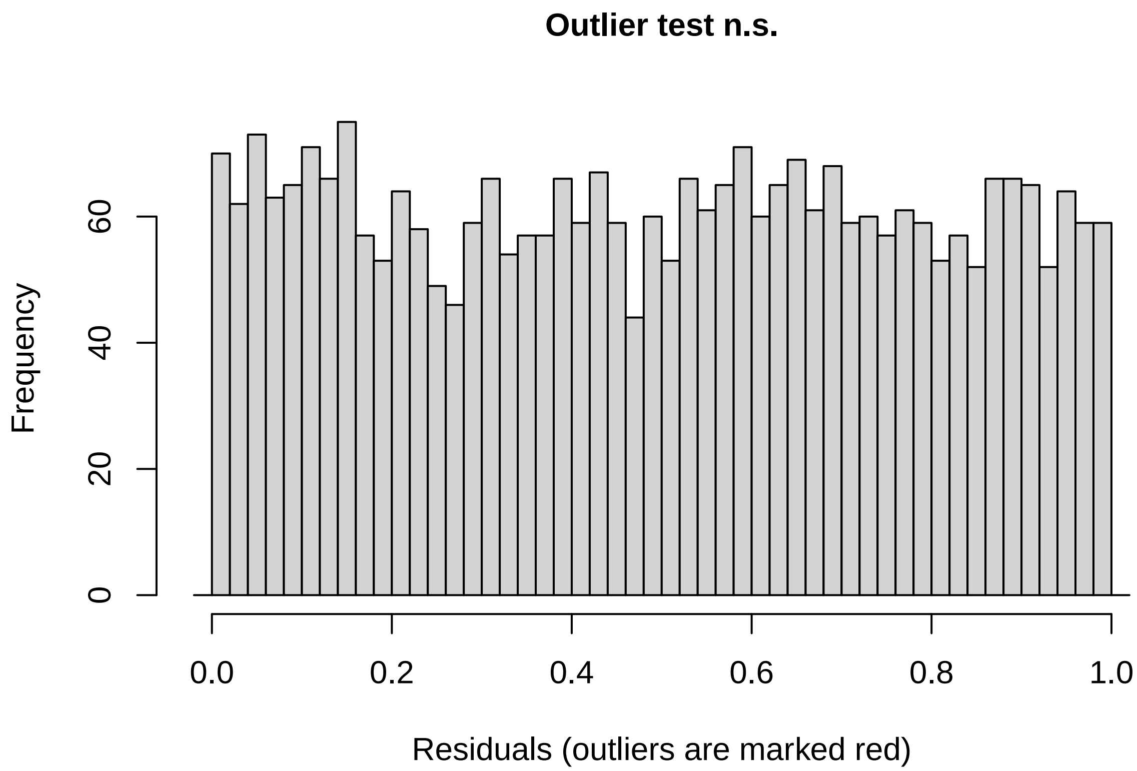


```
##
##  DHARMa outlier test based on exact binomial test with approximate
##  expectations
##
## data:  sot
## outliers at both margin(s) = 28, observations = 3048, p-value = 0.415
## alternative hypothesis: true probability of success is not equal to 0.007968127
## 95 percent confidence interval:
##  0.006112698 0.013249659
## sample estimates:
## frequency of outliers (expected: 0.00796812749003984 )
##                                           0.009186352
```

```
testDispersion(sot)
```

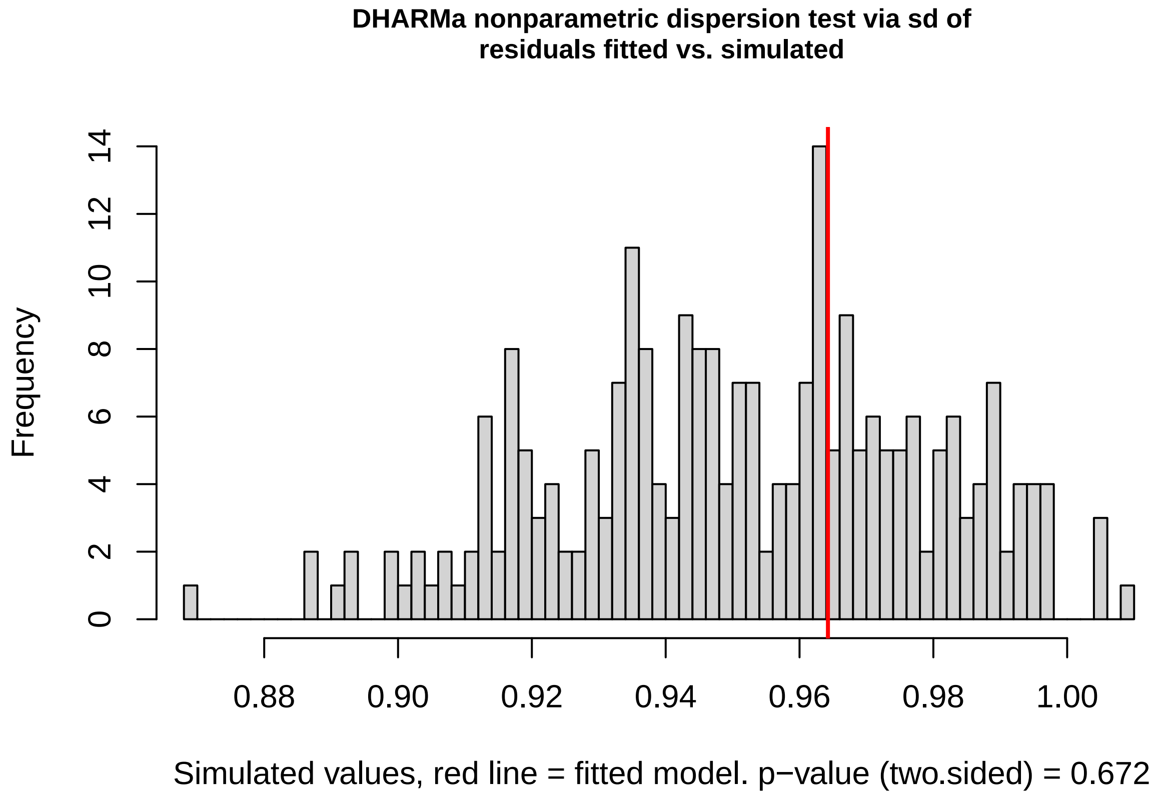


```
##
##  DHARMa nonparametric dispersion test via sd of residuals fitted vs.
##  simulated
##
## data:  simulationOutput
## dispersion = 1.0135, p-value = 0.672
## alternative hypothesis: two.sided
```

```
testZeroInflation(sot)
```

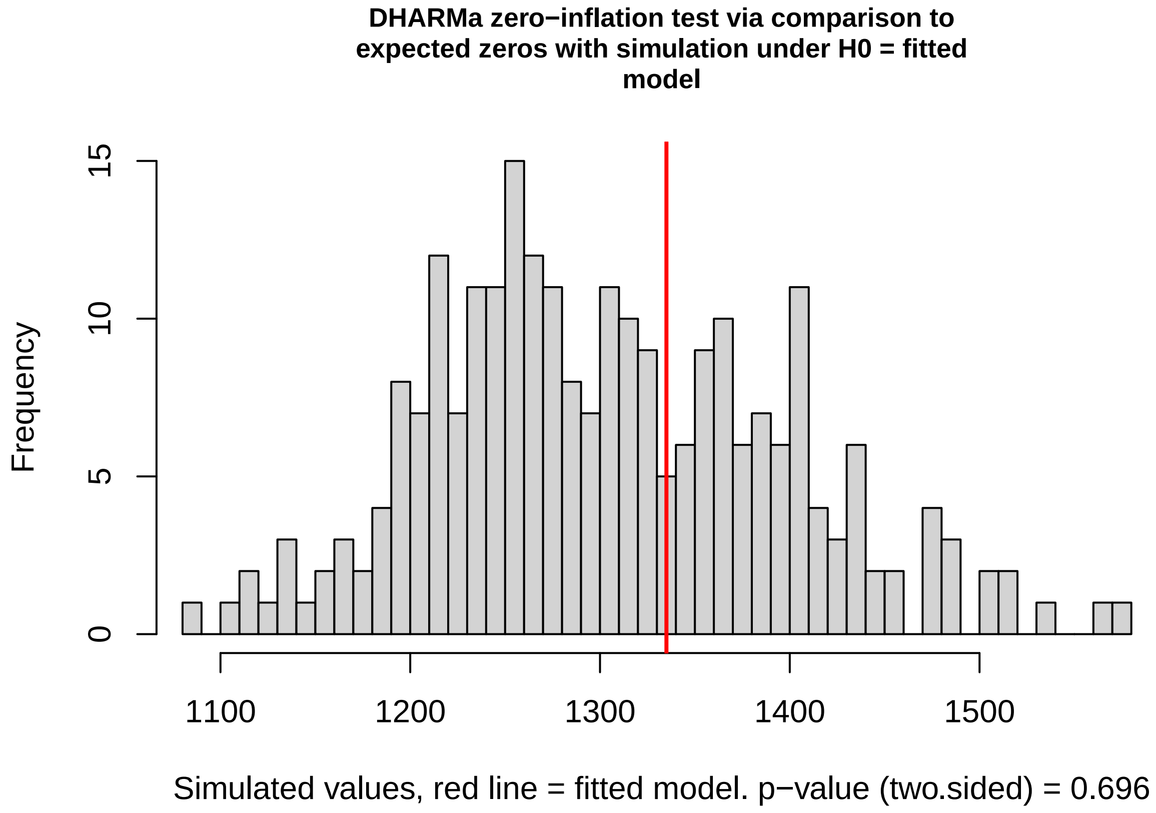


```
##
##  DHARMa zero-inflation test via comparison to expected zeros with
##  simulation under H0 = fitted model
##
## data:  simulationOutput
## ratioObsSim = 1.0236, p-value = 0.696
## alternative hypothesis: two.sided
```

3.8 Checking for effects of length

A model can also be fit to include effects of length (1 vs. 2 syllables). First we start with all factors fully interacting. We clearly do not have power to detect three- or four-way interactions, not to mention the difficulties trying to interpret them. But we can retain them in the model so that the main effects are more accurately estimated (regardless of significance testing considerations).

To achieve a somewhat manageable but not too restrictive random structure, we include random slopes for the interactions of all three factors (grade, lexicality, length) with exposure (reps), but no higher-order interactions. Fixed-effects factors are allowed to interact fully.

```
olfn2 <- as.formula("orthaccu ~ 1 + reps*lex2C*gradeC*Nsyl2C + (1|sID) + (0+(reps6_2+reps2_0)*lex2C|sID
oln2  <- glmer( olfn2, som, family=binomial, control=glmerControl(optCtrl=list(maxfun=1e5), optimizer =
```

```
## boundary (singular) fit: see ?isSingular
```

```
print(summary(oln2),corr=F)
```

```
## Generalized linear mixed model fit by maximum likelihood (Laplace
##   Approximation) [glmerMod]
```

```
##  Family: binomial  ( logit )
## Formula: orthaccu ~ 1 + reps * lex2C * gradeC * Nsyl2C + (1 | sID) + (0 +
##     (reps6_2 + reps2_0) * lex2C | sID) + (0 + (reps6_2 + reps2_0) *
##     Nsyl2C | sID) + (1 | targetS) + (0 + (reps6_2 + reps2_0) *
##     gradeC | targetS)
##    Data: som
## Control: glmerControl(optCtrl = list(maxfun = 1e+05), optimizer = "bobyqa")
##
##      AIC      BIC   logLik deviance df.resid
##   5021.2   5469.2  -2439.6   4879.2     3992
##
## Scaled residuals:
##     Min      1Q  Median      3Q     Max
## -4.1557 -0.8003  0.2855  0.7713  2.1446
##
## Random effects:
##  Groups    Name           Variance Std.Dev. Corr
##  sID       (Intercept)    0.028938 0.17011
##  sID.1     reps6_2        0.018311 0.13532
##            reps2_0        0.021599 0.14697  -0.74
##            lex2C          0.085938 0.29315  -0.86  0.98
##            reps6_2:lex2C  0.583096 0.76361   0.98 -0.86 -0.95
##            reps2_0:lex2C  0.284785 0.53365  -0.97  0.88  0.96 -1.00
##  sID.2     reps6_2        0.401069 0.63330
##            reps2_0        0.105427 0.32470  -0.73
##            Nsyl2C         0.008964 0.09468  -0.43  0.93
##            reps6_2:Nsyl2C 2.117770 1.45526   1.00 -0.70 -0.39
##            reps2_0:Nsyl2C 1.251914 1.11889  -0.91  0.94  0.76 -0.90
##  targetS   (Intercept)    0.651474 0.80714
##  targetS.1 reps6_2        0.126592 0.35580
##            reps2_0        0.030810 0.17553   1.00
##            gradeC         0.817199 0.90399   0.41  0.41
##            reps6_2:gradeC 0.552413 0.74325  -0.27 -0.27 -0.23
##            reps2_0:gradeC 0.322874 0.56822   0.93  0.93  0.53 -0.58
## Number of obs: 4063, groups:  sID, 127; targetS, 32
##
## Fixed effects:
##                             Estimate Std. Error z value Pr(>|z|)
## (Intercept)                  0.51156    0.15006   3.409 0.000652 ***
## reps2_0                      0.25589    0.10379   2.465 0.013683 *
## reps6_2                      0.25909    0.14102   1.837 0.066172 .
## lex2C                       -1.07093    0.29955  -3.575 0.000350 ***
## gradeC                       0.51772    0.18402   2.813 0.004902 **
## Nsyl2C                      -0.28563    0.29805  -0.958 0.337905
## reps2_0:lex2C               -0.44233    0.20370  -2.171 0.029894 *
## reps6_2:lex2C                0.46416    0.26459   1.754 0.079386 .
## reps2_0:gradeC               0.15716    0.22293   0.705 0.480836
## reps6_2:gradeC               0.12438    0.28463   0.437 0.662131
## lex2C:gradeC                -0.67666    0.36610  -1.848 0.064559 .
## reps2_0:Nsyl2C              -0.15143    0.22022  -0.688 0.491668
## reps6_2:Nsyl2C               0.42992    0.28432   1.512 0.130513
## lex2C:Nsyl2C                 0.87038    0.59573   1.461 0.144005
## gradeC:Nsyl2C                0.54482    0.36182   1.506 0.132124
## reps2_0:lex2C:gradeC         0.05073    0.43755   0.116 0.907701
```

```
## reps6_2:lex2C:gradeC        -0.05184    0.53494  -0.097 0.922801
## reps2_0:lex2C:Nsyl2C         0.50569    0.39273   1.288 0.197873
## reps6_2:lex2C:Nsyl2C        -0.93186    0.50425  -1.848 0.064600 .
## reps2_0:gradeC:Nsyl2C       -0.09363    0.46903  -0.200 0.841770
## reps6_2:gradeC:Nsyl2C        0.03000    0.57427   0.052 0.958344
## lex2C:gradeC:Nsyl2C         -0.54504    0.72256  -0.754 0.450656
## reps2_0:lex2C:gradeC:Nsyl2C -0.18495    0.84726  -0.218 0.827204
## reps6_2:lex2C:gradeC:Nsyl2C -0.56145    1.02044  -0.550 0.582181
## ---
## Signif. codes:  0 '***' 0.001 '**' 0.01 '*' 0.05 '.' 0.1 ' ' 1
## optimizer (bobyqa) convergence code: 0 (OK)
## boundary (singular) fit: see ?isSingular
```

This leads to singular fit due to overparameterized random structure. With subsequent reductions as above we can reach a reduced model with nonsingular fit.

```
olfn4 <- as.formula("orthaccu ~ 1 + reps*lex2C*gradeC*Nsyl2C + (reps6_2+reps6_2:Nsyl2C||sID) + (reps6_2
oln4  <- glmer( olfn4, som, family=binomial, control=glmerControl(optCtrl=list(maxfun=1e5), optimizer =
print(summary(oln4),corr=F)
```

```
## Generalized linear mixed model fit by maximum likelihood (Laplace
##   Approximation) [glmerMod]
##  Family: binomial  ( logit )
## Formula:
## orthaccu ~ 1 + reps * lex2C * gradeC * Nsyl2C + (reps6_2 + reps6_2:Nsyl2C ||
##     sID) + (reps6_2 + reps2_0 + gradeC || targetS)
##    Data: som
## Control: glmerControl(optCtrl = list(maxfun = 1e+05), optimizer = "bobyqa")
##
##      AIC      BIC   logLik deviance df.resid
##   4972.1   5167.7  -2455.0   4910.1     4032
##
## Scaled residuals:
##     Min      1Q  Median      3Q     Max
## -6.0197 -0.8173  0.2907  0.7782  2.1977
##
## Random effects:
##  Groups    Name           Variance Std.Dev.
##  sID       (Intercept)    0.03440  0.1855
##  sID.1     reps6_2        0.17695  0.4207
##  sID.2     reps6_2:Nsyl2C 0.22715  0.4766
##  targetS   (Intercept)    0.62812  0.7925
##  targetS.1 reps6_2        0.11792  0.3434
##  targetS.2 reps2_0        0.05248  0.2291
##  targetS.3 gradeC         0.67533  0.8218
## Number of obs: 4063, groups:  sID, 127; targetS, 32
##
## Fixed effects:
##                              Estimate Std. Error z value Pr(>|z|)
## (Intercept)                  0.489448   0.147251   3.324 0.000888 ***
## reps2_0                      0.237220   0.098981   2.397 0.016547 *
## reps6_2                      0.242402   0.127782   1.897 0.057828 .
## lex2C                       -1.031406   0.292572  -3.525 0.000423 ***
## gradeC                       0.477127   0.170229   2.803 0.005065 **
## Nsyl2C                      -0.313650   0.292181  -1.073 0.283057
```

```
## reps2_0:lex2C               -0.368087   0.197952  -1.859 0.062960 .
## reps6_2:lex2C                0.429415   0.243500   1.764 0.077814 .
## reps2_0:gradeC               0.105615   0.179539   0.588 0.556359
## reps6_2:gradeC               0.128229   0.222691   0.576 0.564740
## lex2C:gradeC                -0.624464   0.333727  -1.871 0.061320 .
## reps2_0:Nsyl2C              -0.180092   0.197876  -0.910 0.362758
## reps6_2:Nsyl2C               0.393840   0.247235   1.593 0.111165
## lex2C:Nsyl2C                 0.902419   0.584290   1.544 0.122475
## gradeC:Nsyl2C                0.486290   0.333021   1.460 0.144225
## reps2_0:lex2C:gradeC         0.107779   0.359110   0.300 0.764080
## reps6_2:lex2C:gradeC         0.005333   0.418939   0.013 0.989844
## reps2_0:lex2C:Nsyl2C         0.570192   0.396244   1.439 0.150152
## reps6_2:lex2C:Nsyl2C        -0.926697   0.487826  -1.900 0.057480 .
## reps2_0:gradeC:Nsyl2C       -0.177065   0.359034  -0.493 0.621892
## reps6_2:gradeC:Nsyl2C        0.067851   0.427562   0.159 0.873911
## lex2C:gradeC:Nsyl2C         -0.468401   0.665902  -0.703 0.481801
## reps2_0:lex2C:gradeC:Nsyl2C -0.010022   0.718455  -0.014 0.988870
## reps6_2:lex2C:gradeC:Nsyl2C -0.630186   0.837812  -0.752 0.451942
## ---
## Signif. codes:  0 '***' 0.001 '**' 0.01 '*' 0.05 '.' 0.1 ' ' 1
```

The model with reduced random structure is no worse than the original model with all random slopes:

```
anova(oln2,oln4)
```

```
## Data: som
## Models:
## oln4: orthaccu ~ 1 + reps * lex2C * gradeC * Nsyl2C + (reps6_2 + reps6_2:Nsyl2C || sID) + (reps6_2 +
## oln2: orthaccu ~ 1 + reps * lex2C * gradeC * Nsyl2C + (1 | sID) + (0 + (reps6_2 + reps2_0) * lex2C |
##      npar    AIC    BIC  logLik deviance  Chisq Df Pr(>Chisq)
## oln4   31 4972.1 5167.7 -2455.1   4910.1
## oln2   71 5021.2 5469.2 -2439.6   4879.2 30.853 40     0.8501
```

Here is its Anova table:

```
Anova(oln4)
```

```
## Analysis of Deviance Table (Type II Wald chisquare tests)
##
## Response: orthaccu
##                               Chisq Df Pr(>Chisq)
## reps                        15.1833  2  0.0005047 ***
## lex2C                       11.1638  1  0.0008341 ***
## gradeC                       6.8467  1  0.0088807 **
## Nsyl2C                       1.1683  1  0.2797488
## reps:lex2C                   4.2974  2  0.1166329
## reps:gradeC                  1.6393  2  0.4405922
## lex2C:gradeC                 3.8401  1  0.0500391 .
## reps:Nsyl2C                  1.8752  2  0.3915621
## lex2C:Nsyl2C                 2.3285  1  0.1270246
## gradeC:Nsyl2C                2.3212  1  0.1276201
## reps:lex2C:gradeC            0.1511  2  0.9272426
## reps:lex2C:Nsyl2C            3.7110  2  0.1563750
## reps:gradeC:Nsyl2C           0.3109  2  0.8560247
## lex2C:gradeC:Nsyl2C          0.3866  1  0.5341107
## reps:lex2C:gradeC:Nsyl2C     0.8733  2  0.6462109
## ---
```

```
## Signif. codes:  0 '***' 0.001 '**' 0.01 '*' 0.05 '.' 0.1 ' ' 1
```

And here are the estimates of the original and reduced model, side by side for comparability:

```
cbind(round(coef(summary(oln2)),3),round(coef(summary(oln4)),3)) -> cb24
colnames(cb24) <- rep(c("Estim","Std.Err","z","p"),2)
cb24[1:(nrow(cb24)-2),] # omit 4-way interactions to limit display width
```

```
##                          Estim Std.Err      z     p  Estim Std.Err      z     p
## (Intercept)              0.512   0.150  3.409 0.001  0.489   0.147  3.324 0.001
## reps2_0                  0.256   0.104  2.465 0.014  0.237   0.099  2.397 0.017
## reps6_2                  0.259   0.141  1.837 0.066  0.242   0.128  1.897 0.058
## lex2C                   -1.071   0.300 -3.575 0.000 -1.031   0.293 -3.525 0.000
## gradeC                   0.518   0.184  2.813 0.005  0.477   0.170  2.803 0.005
## Nsyl2C                  -0.286   0.298 -0.958 0.338 -0.314   0.292 -1.073 0.283
## reps2_0:lex2C           -0.442   0.204 -2.171 0.030 -0.368   0.198 -1.859 0.063
## reps6_2:lex2C            0.464   0.265  1.754 0.079  0.429   0.244  1.764 0.078
## reps2_0:gradeC           0.157   0.223  0.705 0.481  0.106   0.180  0.588 0.556
## reps6_2:gradeC           0.124   0.285  0.437 0.662  0.128   0.223  0.576 0.565
## lex2C:gradeC            -0.677   0.366 -1.848 0.065 -0.624   0.334 -1.871 0.061
## reps2_0:Nsyl2C          -0.151   0.220 -0.688 0.492 -0.180   0.198 -0.910 0.363
## reps6_2:Nsyl2C           0.430   0.284  1.512 0.131  0.394   0.247  1.593 0.111
## lex2C:Nsyl2C             0.870   0.596  1.461 0.144  0.902   0.584  1.544 0.122
## gradeC:Nsyl2C            0.545   0.362  1.506 0.132  0.486   0.333  1.460 0.144
## reps2_0:lex2C:gradeC     0.051   0.438  0.116 0.908  0.108   0.359  0.300 0.764
## reps6_2:lex2C:gradeC    -0.052   0.535 -0.097 0.923  0.005   0.419  0.013 0.990
## reps2_0:lex2C:Nsyl2C     0.506   0.393  1.288 0.198  0.570   0.396  1.439 0.150
## reps6_2:lex2C:Nsyl2C    -0.932   0.504 -1.848 0.065 -0.927   0.488 -1.900 0.057
## reps2_0:gradeC:Nsyl2C   -0.094   0.469 -0.200 0.842 -0.177   0.359 -0.493 0.622
## reps6_2:gradeC:Nsyl2C    0.030   0.574  0.052 0.958  0.068   0.428  0.159 0.874
## lex2C:gradeC:Nsyl2C     -0.545   0.723 -0.754 0.451 -0.468   0.666 -0.703 0.482
```

Importantly, note that the reduced model is not less conservative than the original model when it comes to detecting significant two-way interactions with reps. Indeed, an effect of lexicality on early learning that was significant in the original (exposures(2-0) × lexicality) is not significant in the reduced model, despite the lack of corresponding random slopes. In fact the estimates and standard errors for all terms are very similar in the two models. This is consistent with the idea that full random structures are important for controlling Type I error rate only when they are supported by the data; degenerate random structures only reduce power.

The analysis with the length factor included confirms that there are no major effects on orthographic learning rate by either variable; and the the variable most likely to have an effect (smaller than can be confidently detected by the present sample) is lexicality.

Effects and diagnostics for the reduced model follow:

```
plot(allEffects(oln4,xlevels=list(lex2C=c(-0.5,0.5),gradeC=c(-0.5,0.5),Nsyl2C=c(-0.5,0.5))))
```

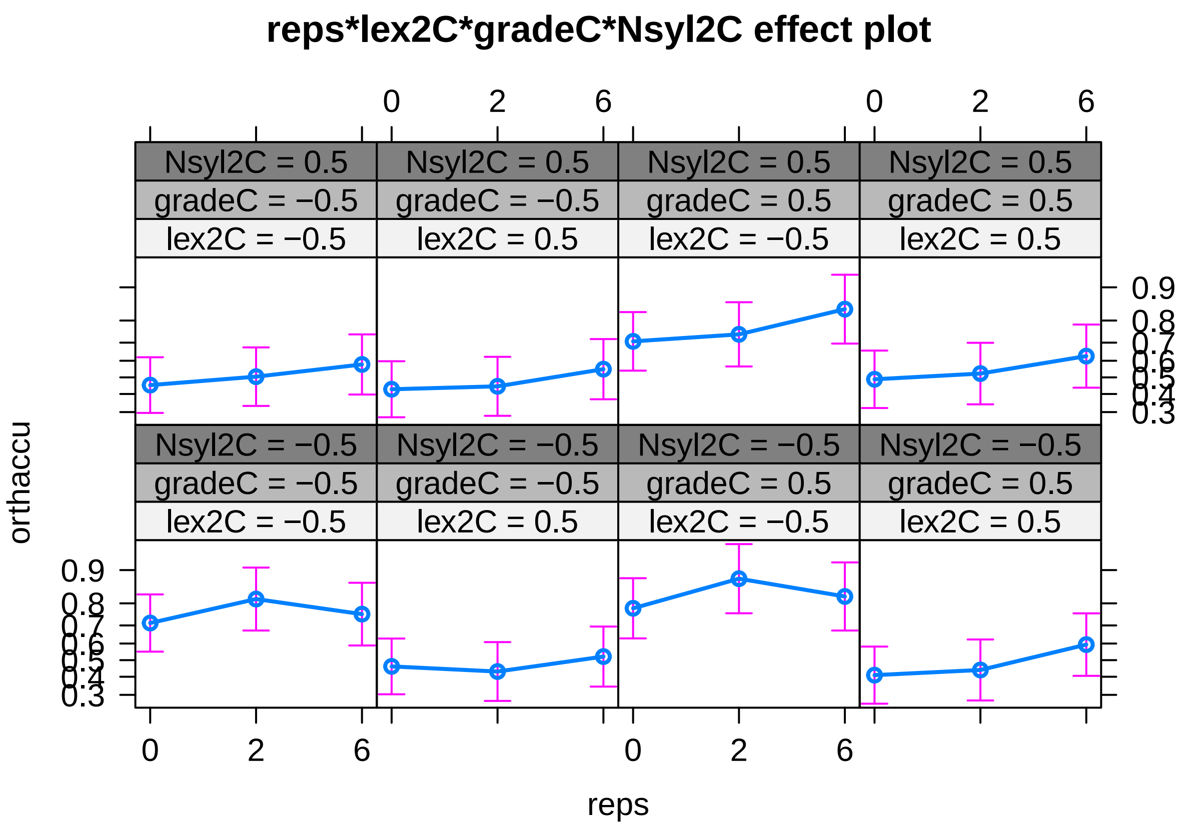

Reminder: Grade (left-right half), Grade 2 = −0.5, Grade 5 = +0.5; Lexicality (columns), words = −0.5, pseudowords = +0.5; Length (rows), 1 syllable = −0.5, 2 syllables +0.5.

And here are the fit diagnostics:

```
sot4 <- simulateResiduals(fittedModel=oln4,plot=F)
testUniformity(sot4)
```

```
## DHARMa:testOutliers with type = binomial may have inflated Type I error rates for integer-valued dis
```

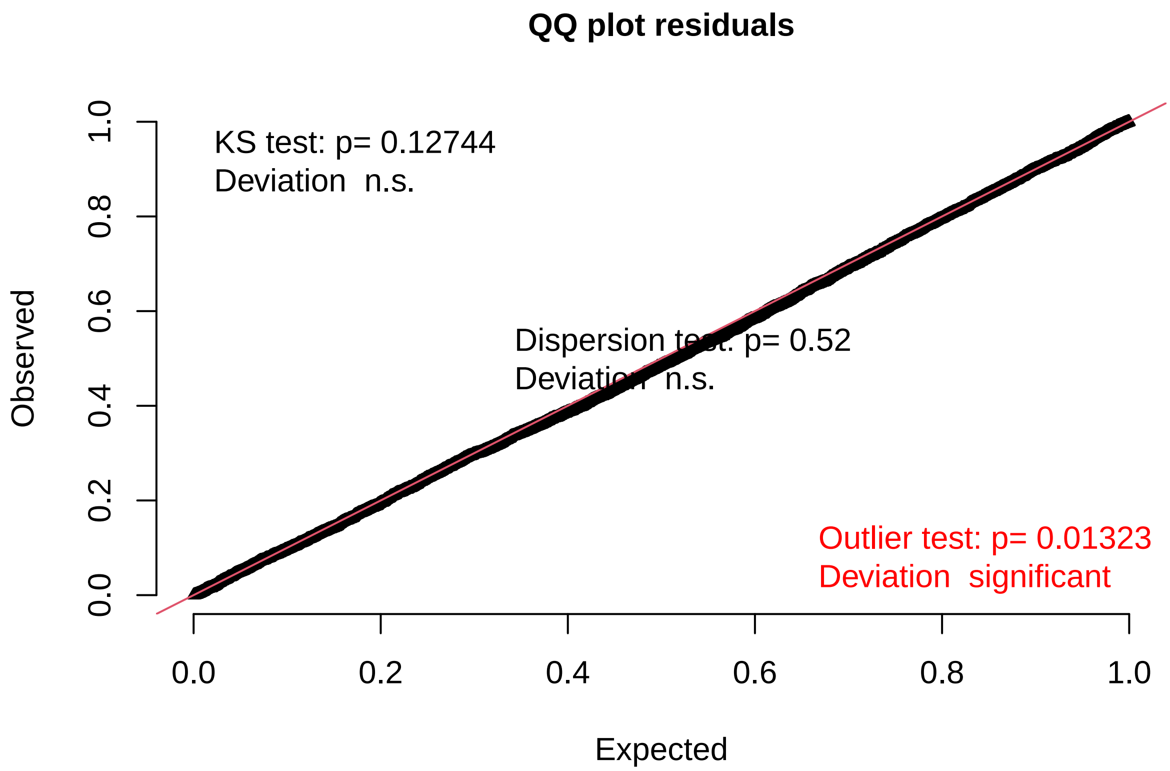


```
##
##  One-sample Kolmogorov-Smirnov test
##
## data:  simulationOutput$scaledResiduals
## D = 0.018406, p-value = 0.1274
## alternative hypothesis: two-sided
```

```
testOutliers(sot4,type="bootstrap",nBoot=1000)
```

```
## DHARMa::testOutliers: nBoot > nSim does not make much sense, thus changed to nBoot = nSim. If you wa
```

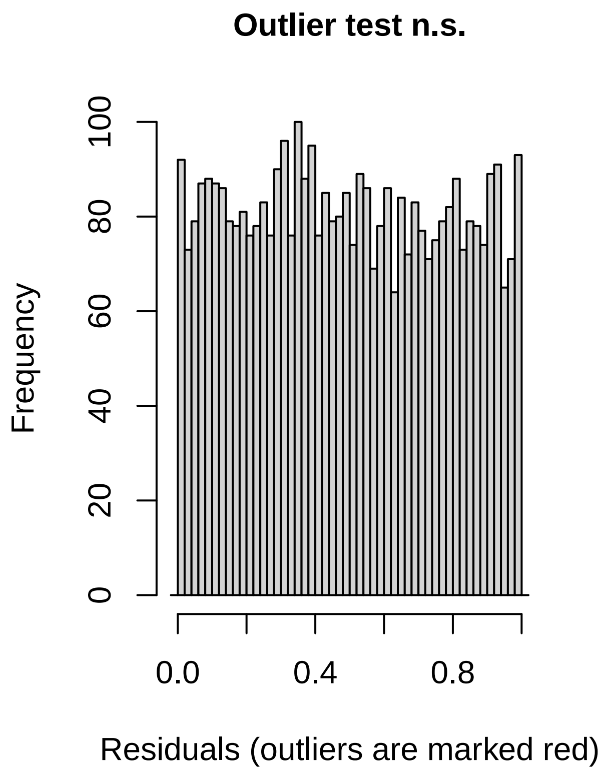

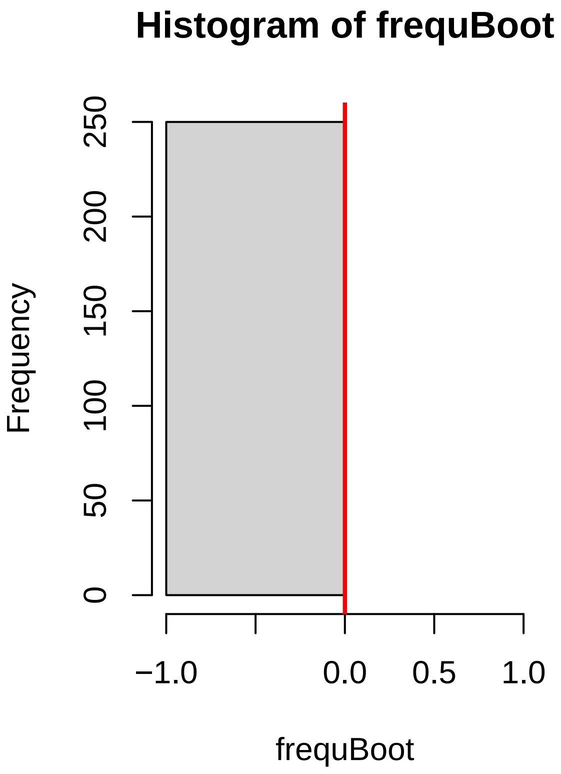


```
##
##  DHARMa bootstrapped outlier test
##
## data:  sot4
## outliers at both margin(s) = 0, observations = 4063, p-value = 1
## alternative hypothesis: two.sided
##  percent confidence interval:
##  0 0
## sample estimates:
## outlier frequency (expected: 0 )
##                                 0
```

```
testDispersion(sot4)
```

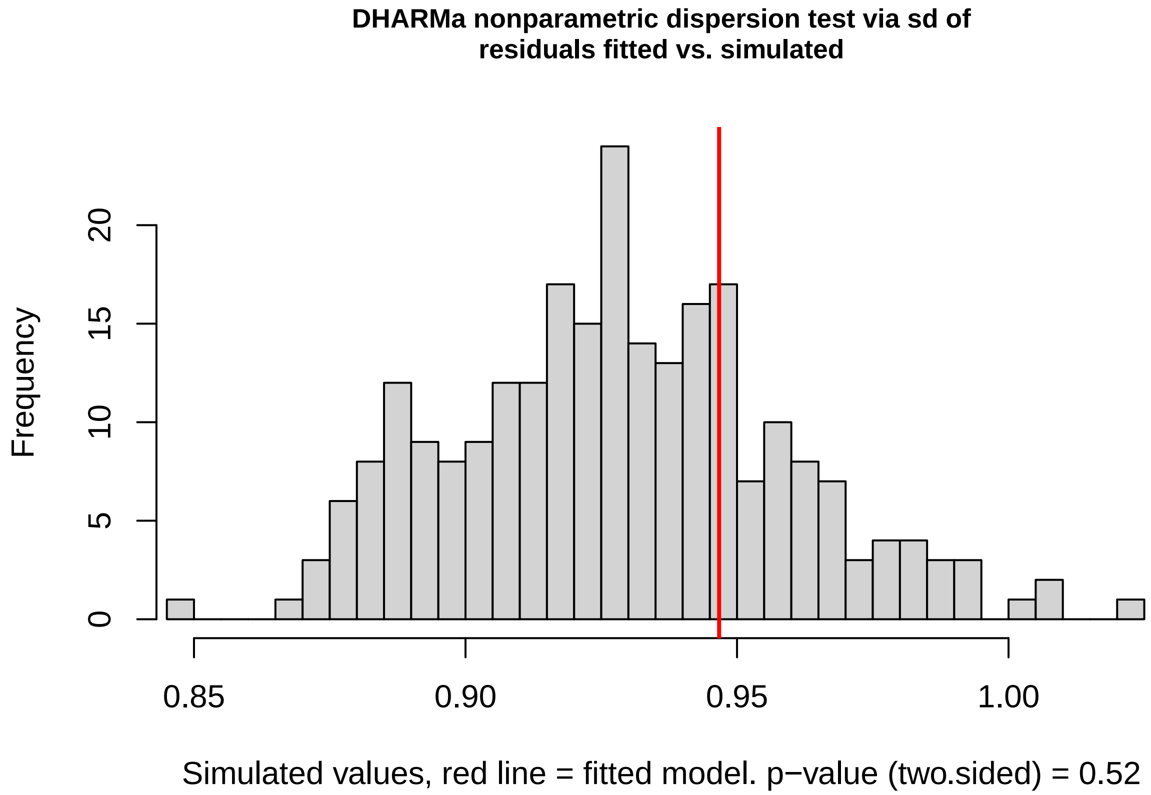


```
##
##  DHARMa nonparametric dispersion test via sd of residuals fitted vs.
##  simulated
##
## data:  simulationOutput
## dispersion = 1.0199, p-value = 0.52
## alternative hypothesis: two.sided
```

```
testZeroInflation(sot4)
```

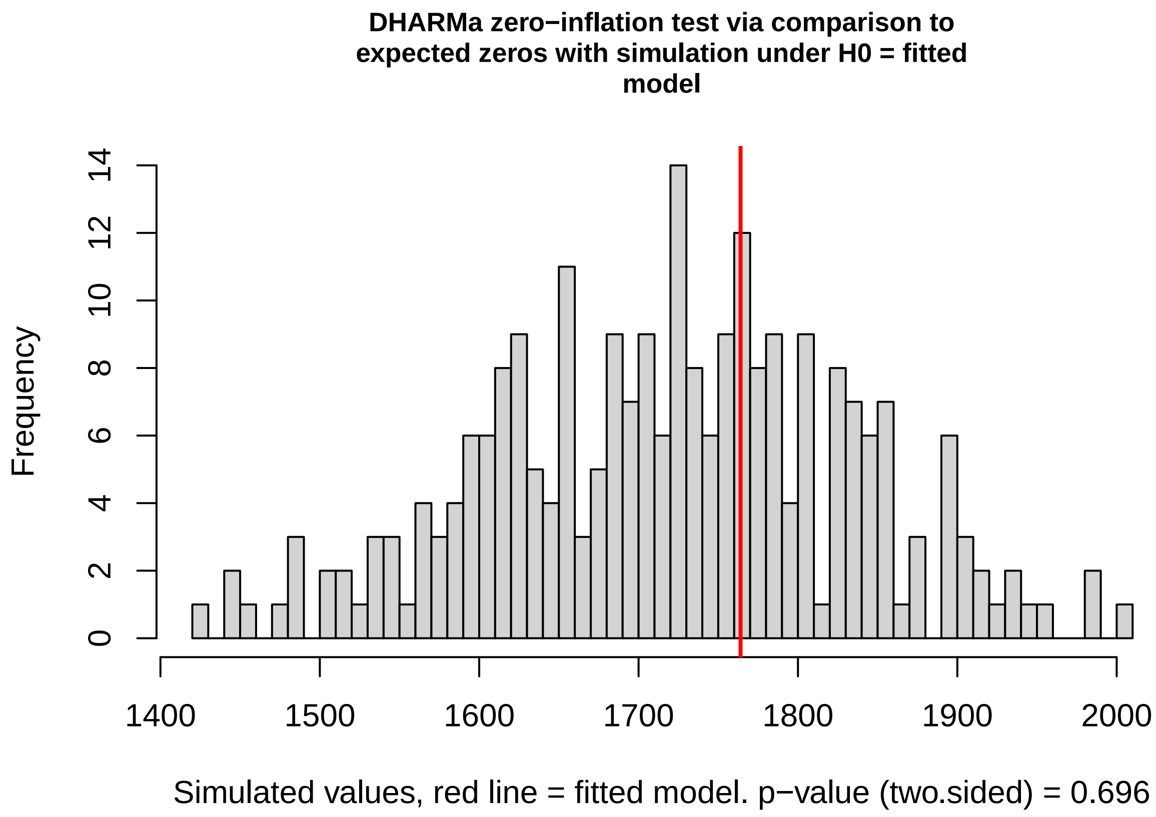


```
##
##  DHARMa zero-inflation test via comparison to expected zeros with
##  simulation under H0 = fitted model
##
## data:  simulationOutput
## ratioObsSim = 1.0262, p-value = 0.696
## alternative hypothesis: two.sided
```

We run a final check with the total learning effect (0 vs. 6 exposures) and the factor length included (negligible random effects removed).

```
olfn5 <- as.formula("orthaccu ~ 1 + repsC*lex2C*gradeC*Nsyl2C + (repsC||sID) + (repsC*gradeC||targetS)"
oln5  <- glmer( olfn5, som, family=binomial, control=glmerControl(optCtrl=list(maxfun=1e5), optimizer =
print(summary(oln5),corr=F)
```

```
## Generalized linear mixed model fit by maximum likelihood (Laplace
##   Approximation) [glmerMod]
##  Family: binomial  ( logit )
## Formula: orthaccu ~ 1 + repsC * lex2C * gradeC * Nsyl2C + (repsC || sID) +
##     (repsC * gradeC || targetS)
##    Data: som
## Control: glmerControl(optCtrl = list(maxfun = 1e+05), optimizer = "bobyqa")
##
##      AIC      BIC   logLik deviance df.resid
##   3781.5   3914.0  -1868.7   3737.5     3026
##
## Scaled residuals:
```

```
##     Min      1Q  Median      3Q     Max 
## -3.6081 -0.8348  0.3206  0.8060  2.2913 
## 
## Random effects:
##  Groups    Name        Variance Std.Dev.
##  sID       (Intercept) 0.03121  0.1767  
##  sID.1     repsC       0.10186  0.3192  
##  targetS   (Intercept) 0.59233  0.7696  
##  targetS.1 repsC       0.21225  0.4607  
##  targetS.2 gradeC      0.56891  0.7543  
##  targetS.3 repsC:gradeC 0.27380  0.5233  
## Number of obs: 3048, groups:  sID, 127; targetS, 32
## 
## Fixed effects:
##                            Estimate Std. Error z value Pr(>|z|)    
## (Intercept)                 0.47630    0.14502   3.284 0.001022 ** 
## repsC                       0.47056    0.12552   3.749 0.000178 ***
## lex2C                      -0.87810    0.28820  -3.047 0.002313 ** 
## gradeC                      0.47122    0.16575   2.843 0.004468 ** 
## Nsyl2C                     -0.21989    0.28777  -0.764 0.444807    
## repsC:lex2C                 0.07984    0.24408   0.327 0.743581    
## repsC:gradeC                0.19833    0.21082   0.941 0.346825    
## lex2C:gradeC               -0.62083    0.32512  -1.910 0.056196 .  
## repsC:Nsyl2C                0.20224    0.24414   0.828 0.407457    
## lex2C:Nsyl2C                0.64951    0.57557   1.128 0.259120    
## gradeC:Nsyl2C               0.50441    0.32426   1.556 0.119807    
## repsC:lex2C:gradeC          0.11710    0.40535   0.289 0.772672    
## repsC:lex2C:Nsyl2C         -0.35174    0.48879  -0.720 0.471770    
## repsC:gradeC:Nsyl2C        -0.14087    0.40605  -0.347 0.728644    
## lex2C:gradeC:Nsyl2C        -0.52290    0.64834  -0.807 0.419940    
## repsC:lex2C:gradeC:Nsyl2C -0.59669    0.81128  -0.735 0.462039    
## ---
## Signif. codes:  0 '***' 0.001 '**' 0.01 '*' 0.05 '.' 0.1 ' ' 1
```

Still no hint of a significant interaction with exposure, consistent with largely uniform orthographic learning rates across grade, lexicality, and length, at least as measured with the orthographic choice task.

Reminder: Grade (left-right half), Grade 2 = -0.5, Grade 5 = $+0.5$; Lexicality (columns), words = -0.5, pseudowords = $+0.5$; Length (rows), 1 syllable = -0.5, 2 syllables $+0.5$.

```
plot(allEffects(oln5,xlevels=list(repsC=c(-0.5,0.5),lex2C=c(-0.5,0.5),gradeC=c(-0.5,0.5),Nsyl2C=c(-0.5,
```

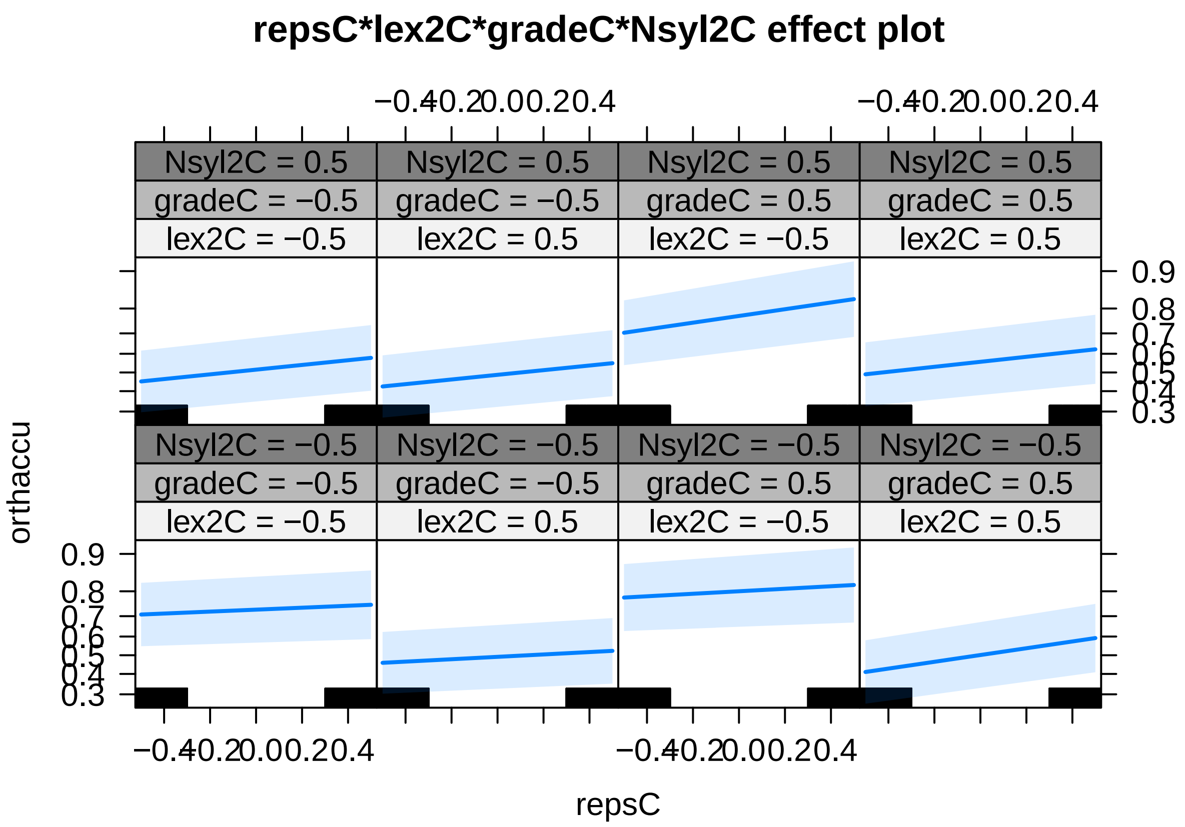


```
sot5 <- simulateResiduals(fittedModel=oln5,plot=F)
testUniformity(sot5)
```

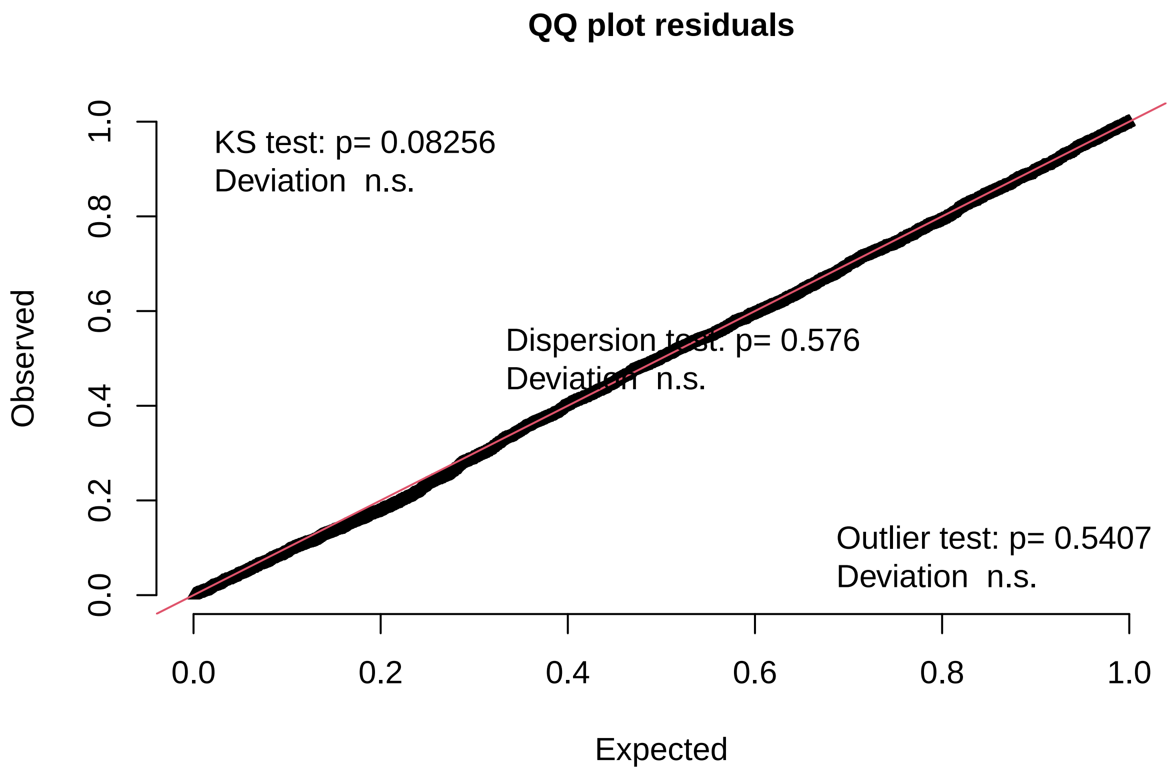


```
##
##  One-sample Kolmogorov-Smirnov test
##
## data:  simulationOutput$scaledResiduals
## D = 0.022866, p-value = 0.08256
## alternative hypothesis: two-sided
```

```
testOutliers(sot5)
```

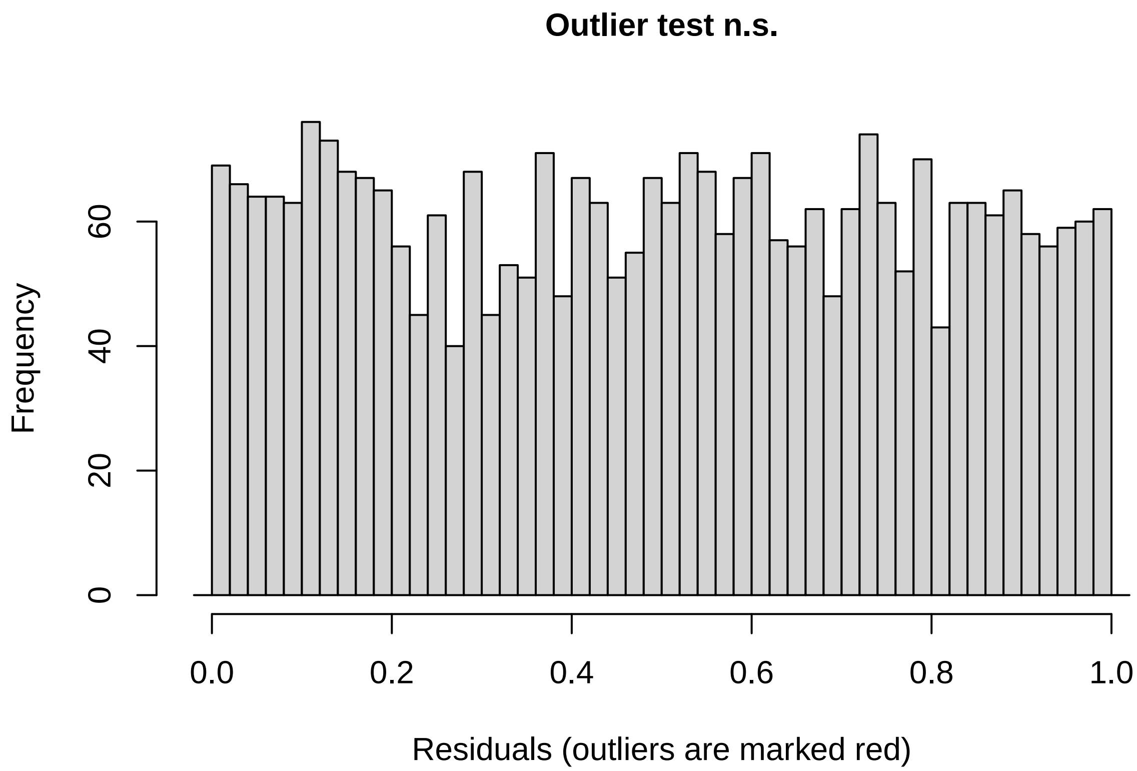


```
##
##  DHARMa outlier test based on exact binomial test with approximate
##  expectations
##
## data:  sot5
## outliers at both margin(s) = 27, observations = 3048, p-value = 0.5407
## alternative hypothesis: true probability of success is not equal to 0.007968127
## 95 percent confidence interval:
##  0.005845539 0.012862325
## sample estimates:
## frequency of outliers (expected: 0.00796812749003984 )
##                                            0.008858268
```

```
testDispersion(sot5)
```

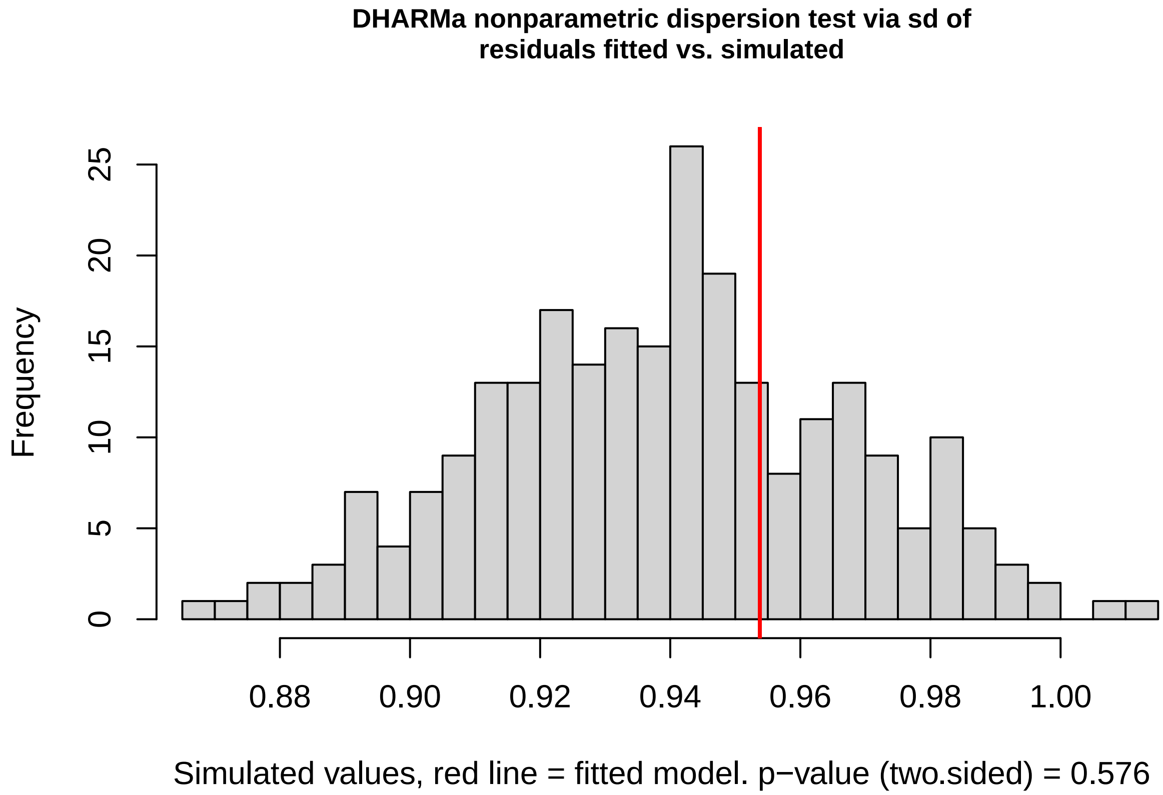


```
##
##  DHARMa nonparametric dispersion test via sd of residuals fitted vs.
##  simulated
##
## data:  simulationOutput
## dispersion = 1.0156, p-value = 0.576
## alternative hypothesis: two.sided
```

```
testZeroInflation(sot5)
```

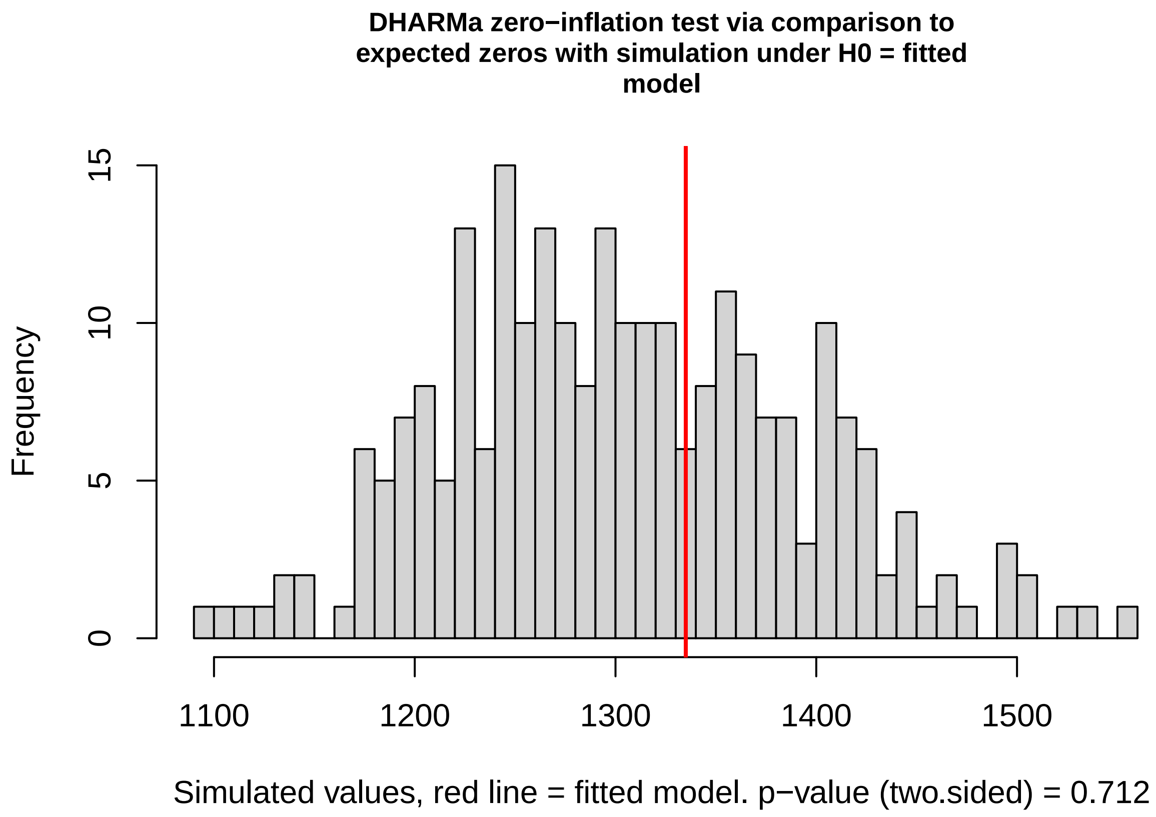


```
##
##  DHARMa zero-inflation test via comparison to expected zeros with
##  simulation under H0 = fitted model
##
## data:  simulationOutput
## ratioObsSim = 1.0229, p-value = 0.712
## alternative hypothesis: two.sided
```

---

4 Spelling

Spelling accuracy per trial (item) is coded as 0 (incorrect) or 1 (correct).

4.1 Fit model for lexicality and grade

The model with rich random structure includes interactions between reps and each of the predictors.

```
spfrm1 <- as.formula( "spelaccu ~ 1 + reps*lex2C*gradeC + ((reps6_2+reps2_0)*lex2C|sID) + ((reps6_2+reps
sp3c1 <- glmer( spfrm1, som, family=binomial, control=glmerControl(optCtrl=list(maxfun=1e5), optimizer =
```

```
## boundary (singular) fit: see ?isSingular
```

```
print(summary(sp3c1),corr=F)
```

```
## Generalized linear mixed model fit by maximum likelihood (Laplace
##   Approximation) [glmerMod]
##  Family: binomial  ( logit )
```

```
## Formula: spelaccu ~ 1 + reps * lex2C * gradeC + ((reps6_2 + reps2_0) *
##     lex2C | sID) + ((reps6_2 + reps2_0) * gradeC | targetS)
##    Data: som
## Control: glmerControl(optCtrl = list(maxfun = 1e+05), optimizer = "bobyqa")
##
##      AIC      BIC   logLik deviance df.resid
##   3907.9   4248.6  -1899.9   3799.9     4009
##
## Scaled residuals:
##     Min      1Q  Median      3Q     Max
## -3.8067 -0.5382 -0.2433  0.5317  5.1820
##
## Random effects:
##  Groups  Name           Variance Std.Dev. Corr
##  sID     (Intercept)    0.16894  0.4110
##          reps6_2        0.38195  0.6180    0.04
##          reps2_0        0.28502  0.5339   -0.26 -0.70
##          lex2C          0.29554  0.5436    0.71 -0.58  0.00
##          reps6_2:lex2C  0.65056  0.8066   -0.73 -0.46  0.85 -0.44
##          reps2_0:lex2C  0.48088  0.6935    0.71  0.42 -0.85  0.47 -1.00
##  targetS (Intercept)    2.32849  1.5259
##          reps6_2        0.05084  0.2255   -0.35
##          reps2_0        0.08812  0.2968   -0.89  0.02
##          gradeC         0.93278  0.9658   -0.23  0.87  0.11
##          reps6_2:gradeC 0.30639  0.5535    0.07 -0.38  0.37  0.10
##          reps2_0:gradeC 0.27979  0.5289   -0.96  0.31  0.96  0.34  0.20
## Number of obs: 4063, groups:  sID, 127; targetS, 32
##
## Fixed effects:
##                     Estimate Std. Error z value Pr(>|z|)
## (Intercept)          -1.1352     0.2787  -4.074 4.62e-05 ***
## reps2_0               0.3067     0.1482   2.070 0.038443 *
## reps6_2               0.4601     0.1490   3.089 0.002008 **
## lex2C                -1.5364     0.5538  -2.774 0.005535 **
## gradeC                0.7676     0.2153   3.564 0.000365 ***
## reps2_0:lex2C         0.3442     0.2711   1.270 0.204215
## reps6_2:lex2C         0.3390     0.2717   1.248 0.212094
## reps2_0:gradeC        0.2995     0.2756   1.087 0.277246
## reps6_2:gradeC       -0.4006     0.2956  -1.355 0.175372
## lex2C:gradeC         -0.5937     0.4131  -1.437 0.150672
## reps2_0:lex2C:gradeC  -0.2052     0.5036  -0.408 0.683578
## reps6_2:lex2C:gradeC   0.8077     0.5426   1.489 0.136597
## ---
## Signif. codes:  0 '***' 0.001 '**' 0.01 '*' 0.05 '.' 0.1 ' ' 1
## optimizer (bobyqa) convergence code: 0 (OK)
## boundary (singular) fit: see ?isSingular
```

The singular convergence indicates that the model is overparameterized, but the model has converged and seems otherwise usable.

4.2 Fit and compare reduced model

Again the correlation parameters and several random slopes are redundant, as removing them does not result in significantly inferior model fit.

We first remove correlations between random effects, the resulting model is no worse.

```
spfrm2 <- as.formula( "spelaccu ~ 1 + reps*lex2C*gradeC + ((reps6_2+reps2_0)*lex2C||sID) + ((reps6_2+rep
sp3c2 <- glmer( spfrm2, som, family=binomial, control=glmerControl(optCtrl=list(maxfun=1e5), optimizer =
```

```
## boundary (singular) fit: see ?isSingular
```

```
print(summary(sp3c2),corr=F)
```

```
## Generalized linear mixed model fit by maximum likelihood (Laplace
##   Approximation) [glmerMod]
##  Family: binomial  ( logit )
## Formula: spelaccu ~ 1 + reps * lex2C * gradeC + ((reps6_2 + reps2_0) *
##     lex2C || sID) + ((reps6_2 + reps2_0) * gradeC || targetS)
##    Data: som
## Control: glmerControl(optCtrl = list(maxfun = 1e+05), optimizer = "bobyqa")
##
##      AIC      BIC   logLik deviance df.resid
##   3884.2   4035.6  -1918.1   3836.2     4039
##
## Scaled residuals:
##     Min      1Q  Median      3Q     Max
## -4.4380 -0.5542 -0.2540  0.5458  4.9281
##
## Random effects:
##  Groups    Name           Variance  Std.Dev.
##  sID       (Intercept)    1.525e-01 3.905e-01
##  sID.1     reps6_2        5.184e-02 2.277e-01
##  sID.2     reps2_0        8.773e-02 2.962e-01
##  sID.3     lex2C          7.441e-02 2.728e-01
##  sID.4     reps6_2:lex2C  0.000e+00 0.000e+00
##  sID.5     reps2_0:lex2C  0.000e+00 0.000e+00
##  targetS   (Intercept)    2.302e+00 1.517e+00
##  targetS.1 reps6_2        0.000e+00 0.000e+00
##  targetS.2 reps2_0        0.000e+00 0.000e+00
##  targetS.3 gradeC         8.215e-01 9.064e-01
##  targetS.4 reps6_2:gradeC 4.097e-01 6.401e-01
##  targetS.5 reps2_0:gradeC 1.881e-09 4.337e-05
## Number of obs: 4063, groups:  sID, 127; targetS, 32
##
## Fixed effects:
##                        Estimate Std. Error z value Pr(>|z|)
## (Intercept)             -1.0813     0.2764  -3.912 9.15e-05 ***
## reps2_0                  0.2736     0.1071   2.554 0.010653 *
## reps6_2                  0.3713     0.1154   3.218 0.001291 **
## lex2C                   -1.4785     0.5484  -2.696 0.007018 **
## gradeC                   0.7298     0.2025   3.603 0.000314 ***
## reps2_0:lex2C            0.1996     0.2060   0.969 0.332727
## reps6_2:lex2C            0.2500     0.2267   1.103 0.270220
## reps2_0:gradeC           0.1816     0.2135   0.851 0.395008
## reps6_2:gradeC          -0.3394     0.2602  -1.305 0.192024
## lex2C:gradeC            -0.6368     0.3813  -1.670 0.094890 .
## reps2_0:lex2C:gradeC    -0.4880     0.4116  -1.186 0.235763
## reps6_2:lex2C:gradeC     0.8958     0.5146   1.741 0.081735 .
## ---
```

```
## Signif. codes:  0 '***' 0.001 '**' 0.01 '*' 0.05 '.' 0.1 ' ' 1
## optimizer (bobyqa) convergence code: 0 (OK)
## boundary (singular) fit: see ?isSingular
```

```
anova(sp3c1,sp3c2)
```

```
## Data: som
## Models:
## sp3c2: spelaccu ~ 1 + reps * lex2C * gradeC + ((reps6_2 + reps2_0) * lex2C || sID) + ((reps6_2 + rep
## sp3c1: spelaccu ~ 1 + reps * lex2C * gradeC + ((reps6_2 + reps2_0) * lex2C | sID) + ((reps6_2 + reps
##       npar    AIC    BIC  logLik deviance  Chisq Df Pr(>Chisq)
## sp3c2   24 3884.2 4035.6 -1918.1   3836.2
## sp3c1   54 3907.9 4248.6 -1900.0   3799.9 36.255 30     0.1998
```

We then remove iteratively random effects that are near zero, starting with higher-order ones. This eventually results in a model with nonsingular fit that is no worse than that of the original model.

```
spfrm3 <- as.formula( "spelaccu ~ 1 + reps*lex2C*gradeC + (reps6_2+reps2_0+lex2C||sID) + (gradeC||targe
sp3c3 <- glmer( spfrm3, som, family=binomial, control=glmerControl(optCtrl=list(maxfun=1e5), optimizer =
print(summary(sp3c3),corr=F)
```

```
## Generalized linear mixed model fit by maximum likelihood (Laplace
##   Approximation) [glmerMod]
##  Family: binomial  ( logit )
## Formula: spelaccu ~ 1 + reps * lex2C * gradeC + (reps6_2 + reps2_0 + lex2C ||
##     sID) + (gradeC || targetS)
##    Data: som
## Control: glmerControl(optCtrl = list(maxfun = 1e+05), optimizer = "bobyqa")
##
##      AIC      BIC   logLik deviance df.resid
##   3874.3   3987.9  -1919.1   3838.3     4045
##
## Scaled residuals:
##     Min      1Q  Median      3Q     Max
## -4.4800 -0.5497 -0.2550  0.5510  5.0674
##
## Random effects:
##  Groups    Name        Variance Std.Dev.
##  sID       (Intercept) 0.14961  0.3868
##  sID.1     reps6_2     0.05873  0.2423
##  sID.2     reps2_0     0.09167  0.3028
##  sID.3     lex2C       0.07536  0.2745
##  targetS   (Intercept) 2.28984  1.5132
##  targetS.1 gradeC      0.81103  0.9006
## Number of obs: 4063, groups:  sID, 127; targetS, 32
##
## Fixed effects:
##                       Estimate Std. Error z value Pr(>|z|)
## (Intercept)            -1.0778     0.2756  -3.910 9.22e-05 ***
## reps2_0                 0.2732     0.1072   2.547 0.010856 *
## reps6_2                 0.3779     0.1151   3.284 0.001023 **
## lex2C                  -1.4730     0.5471  -2.692 0.007094 **
## gradeC                  0.7322     0.2014   3.635 0.000278 ***
## reps2_0:lex2C           0.2009     0.2059   0.975 0.329382
## reps6_2:lex2C           0.2500     0.2257   1.108 0.267888
## reps2_0:gradeC          0.1810     0.2135   0.848 0.396669
```

```
## reps6_2:gradeC        -0.3408     0.2299  -1.482 0.138232
## lex2C:gradeC          -0.6347     0.3795  -1.673 0.094416 .
## reps2_0:lex2C:gradeC  -0.4884     0.4114  -1.187 0.235149
## reps6_2:lex2C:gradeC   0.8445     0.4512   1.872 0.061274 .
## ---
## Signif. codes:  0 '***' 0.001 '**' 0.01 '*' 0.05 '.' 0.1 ' ' 1
```

```
anova(sp3c1,sp3c3)
```

```
## Data: som
## Models:
## sp3c3: spelaccu ~ 1 + reps * lex2C * gradeC + (reps6_2 + reps2_0 + lex2C || sID) + (gradeC || targetS
## sp3c1: spelaccu ~ 1 + reps * lex2C * gradeC + ((reps6_2 + reps2_0) * lex2C | sID) + ((reps6_2 + reps2
##       npar    AIC    BIC  logLik deviance  Chisq Df Pr(>Chisq)
## sp3c3   18 3874.3 3987.9 -1919.1   3838.3
## sp3c1   54 3907.9 4248.6 -1900.0   3799.9 38.385 36     0.3619
```

Removing random effects parameters reduces somewhat the estimated standard error for the fixed effects associated with exposure, which suggests that the model is only slightly less conservative. No effects reach significance in the reduced model that did not already in the original. Both early and late learning (main effects reps2_0 and reps6_2, respectively) are statistically significant, but none of the interactions attain significance. This means that there is no evidence that either grade or lexicality affects the orthographic learning effect very much.

Here is its Anova table:

```
Anova(sp3c3)
```

```
## Analysis of Deviance Table (Type II Wald chisquare tests)
##
## Response: spelaccu
##                        Chisq Df  Pr(>Chisq)
## reps                 36.1637  2  1.403e-08 ***
## lex2C                 7.5258  1  0.0060823 **
## gradeC               13.7017  1  0.0002143 ***
## reps:lex2C            4.7389  2  0.0935311 .
## reps:gradeC           2.6332  2  0.2680417
## lex2C:gradeC          2.6109  1  0.1061293
## reps:lex2C:gradeC     3.5209  2  0.1719693
## ---
## Signif. codes:  0 '***' 0.001 '**' 0.01 '*' 0.05 '.' 0.1 ' ' 1
```

Here are the estimates of the original and reduced model, side by side for comparability:

```
cbind(round(coef(summary(sp3c1)),3),round(coef(summary(sp3c3)),3)) -> cbsp13
colnames(cbsp13) <- rep(c("Estim","Std.Err","z","p"),2)
cbsp13
```

```
##                         Estim Std.Err      z     p  Estim Std.Err      z     p
## (Intercept)            -1.135   0.279 -4.074 0.000 -1.078   0.276 -3.910 0.000
## reps2_0                 0.307   0.148  2.070 0.038  0.273   0.107  2.547 0.011
## reps6_2                 0.460   0.149  3.089 0.002  0.378   0.115  3.284 0.001
## lex2C                  -1.536   0.554 -2.774 0.006 -1.473   0.547 -2.692 0.007
## gradeC                  0.768   0.215  3.564 0.000  0.732   0.201  3.635 0.000
## reps2_0:lex2C           0.344   0.271  1.270 0.204  0.201   0.206  0.975 0.329
## reps6_2:lex2C           0.339   0.272  1.248 0.212  0.250   0.226  1.108 0.268
## reps2_0:gradeC          0.299   0.276  1.087 0.277  0.181   0.214  0.848 0.397
## reps6_2:gradeC         -0.401   0.296 -1.355 0.175 -0.341   0.230 -1.482 0.138
```

```
## lex2C:gradeC         -0.594   0.413 -1.437 0.151 -0.635   0.379 -1.673 0.094
## reps2_0:lex2C:gradeC -0.205   0.504 -0.408 0.684 -0.488   0.411 -1.187 0.235
## reps6_2:lex2C:gradeC  0.808   0.543  1.489 0.137  0.844   0.451  1.872 0.061
```

4.3 Model diagnostics

Residual-based diagnostics of the reduced model from DHARMa.

```
sosp3 <- simulateResiduals(fittedModel=sp3c3,plot=F)
testUniformity(sosp3)
```

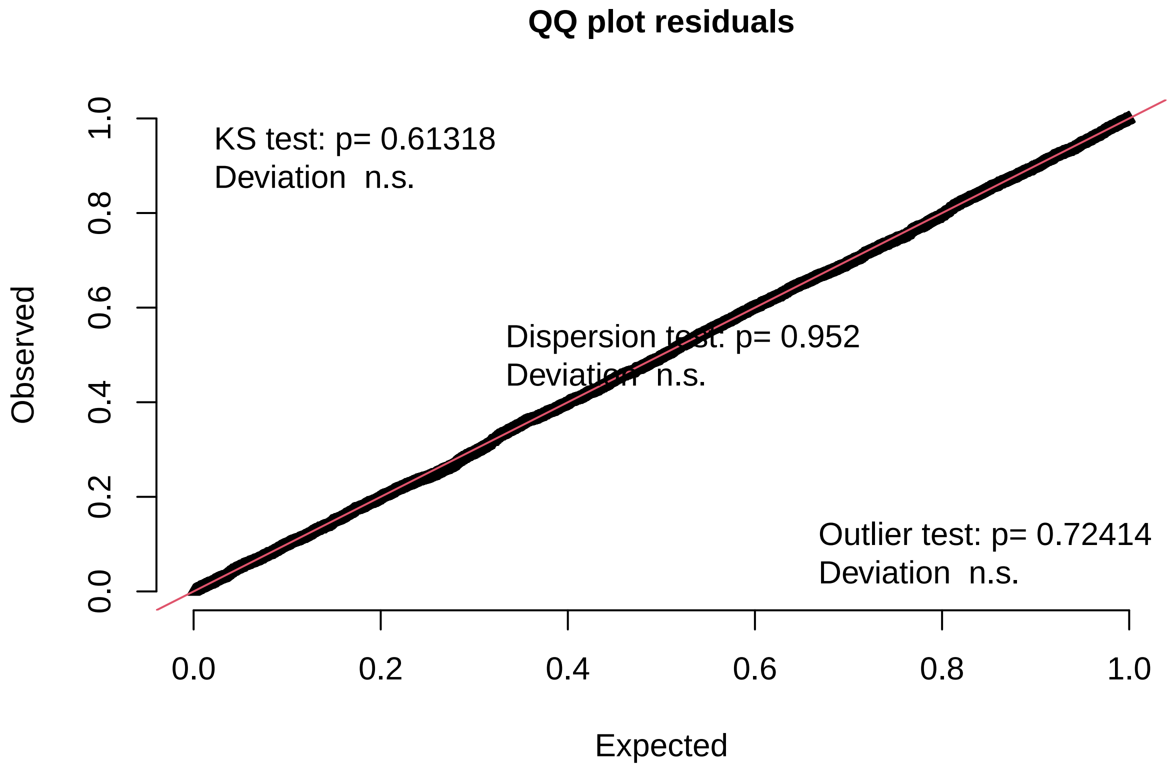


```
##
##  One-sample Kolmogorov-Smirnov test
##
## data:  simulationOutput$scaledResiduals
## D = 0.011897, p-value = 0.6132
## alternative hypothesis: two-sided
```

```
testOutliers(sosp3)
```

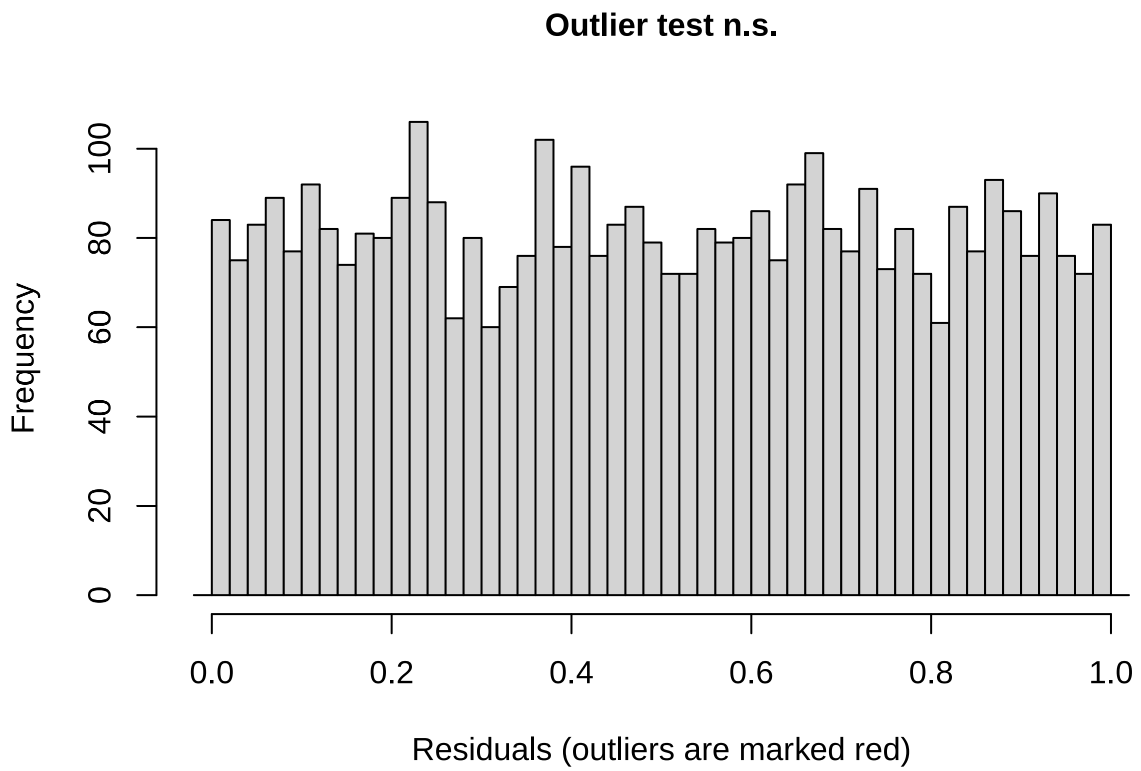


```
##
##  DHARMa outlier test based on exact binomial test with approximate
##  expectations
##
## data:  sosp3
## outliers at both margin(s) = 34, observations = 4063, p-value = 0.7241
## alternative hypothesis: true probability of success is not equal to 0.007968127
## 95 percent confidence interval:
##  0.005801985 0.011674261
## sample estimates:
## frequency of outliers (expected: 0.00796812749003984 )
##                                           0.008368201
```

```
testDispersion(sosp3)
```

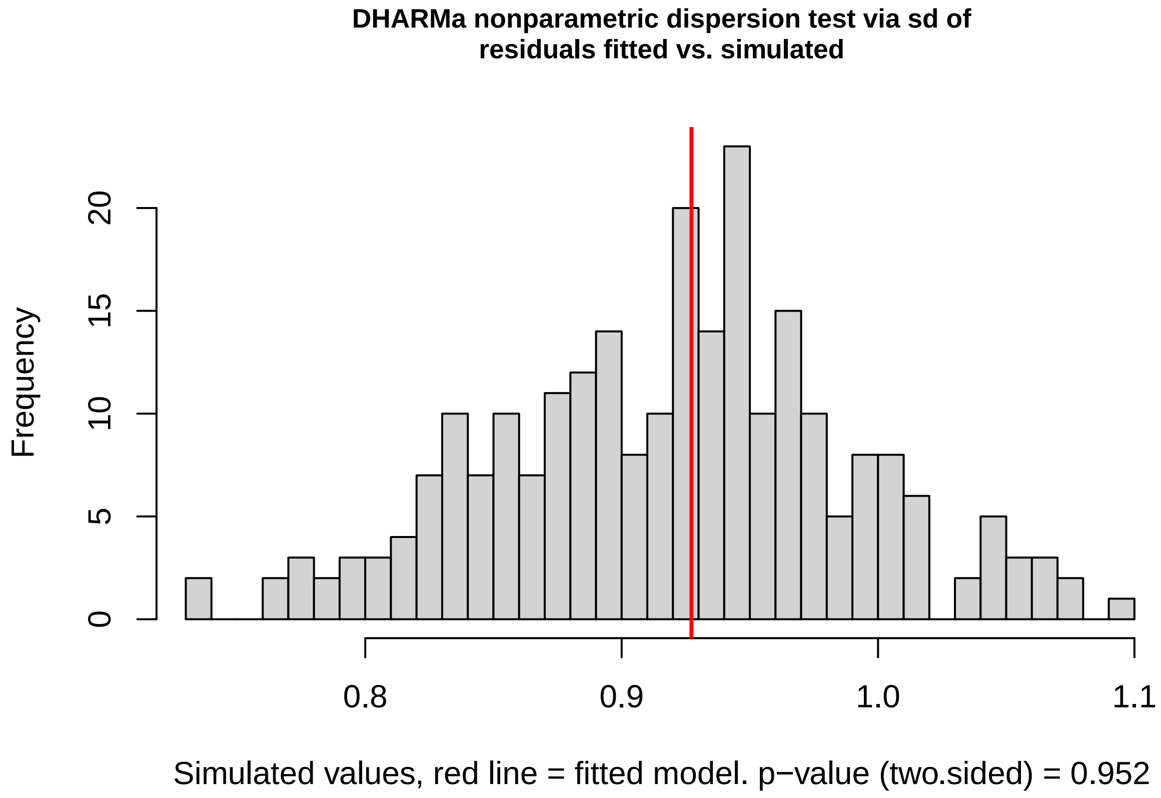


```
##
##  DHARMa nonparametric dispersion test via sd of residuals fitted vs.
##  simulated
##
## data:  simulationOutput
## dispersion = 1.0087, p-value = 0.952
## alternative hypothesis: two.sided
```

```
testZeroInflation(sosp3)
```

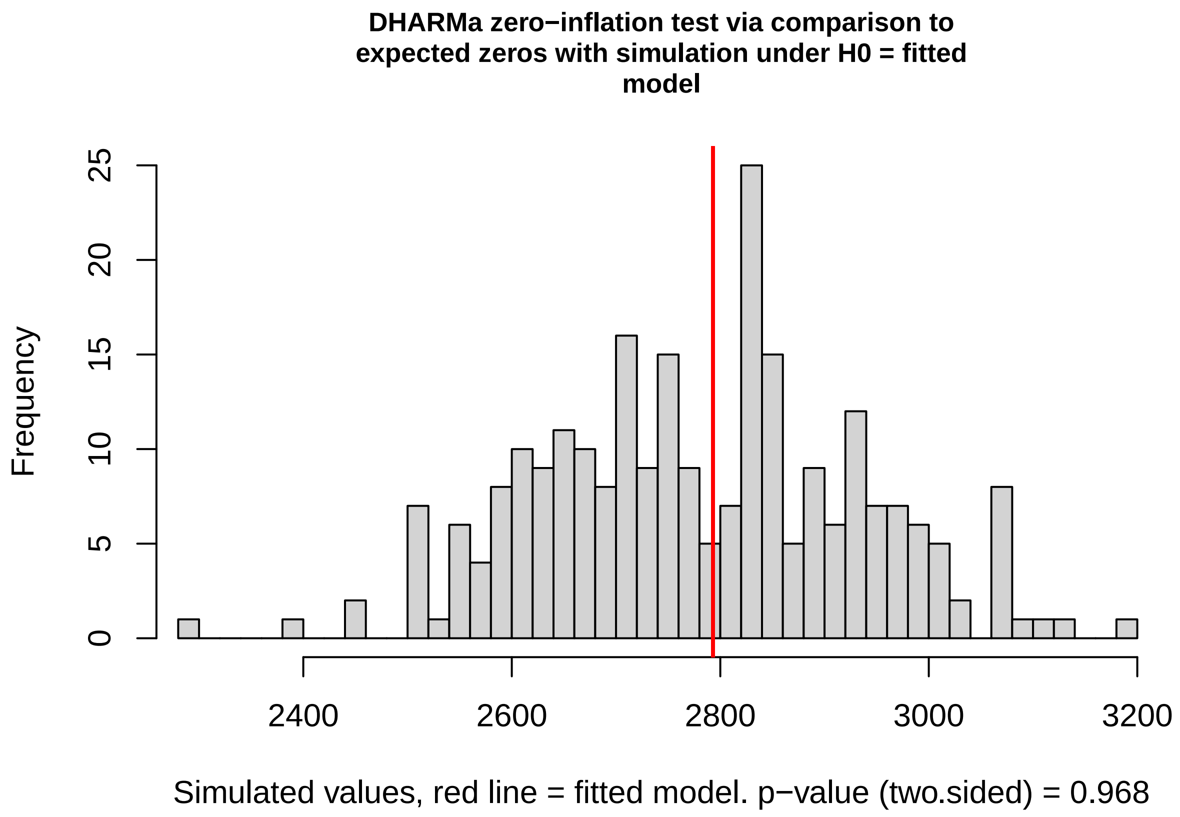


```
##
##  DHARMa zero-inflation test via comparison to expected zeros with
##  simulation under H0 = fitted model
##
## data:  simulationOutput
## ratioObsSim = 1.0043, p-value = 0.968
## alternative hypothesis: two.sided
```

These graphs and tests do not suggest any problems with dispersion or uniformity.

4.4 Effects graphs

These are the effects from the original model (not reduced).

```
plot(allEffects(sp3c1,xlevels=list(lex2C=c(-0.5,0.5),gradeC=c(-0.5,0.5))))
```

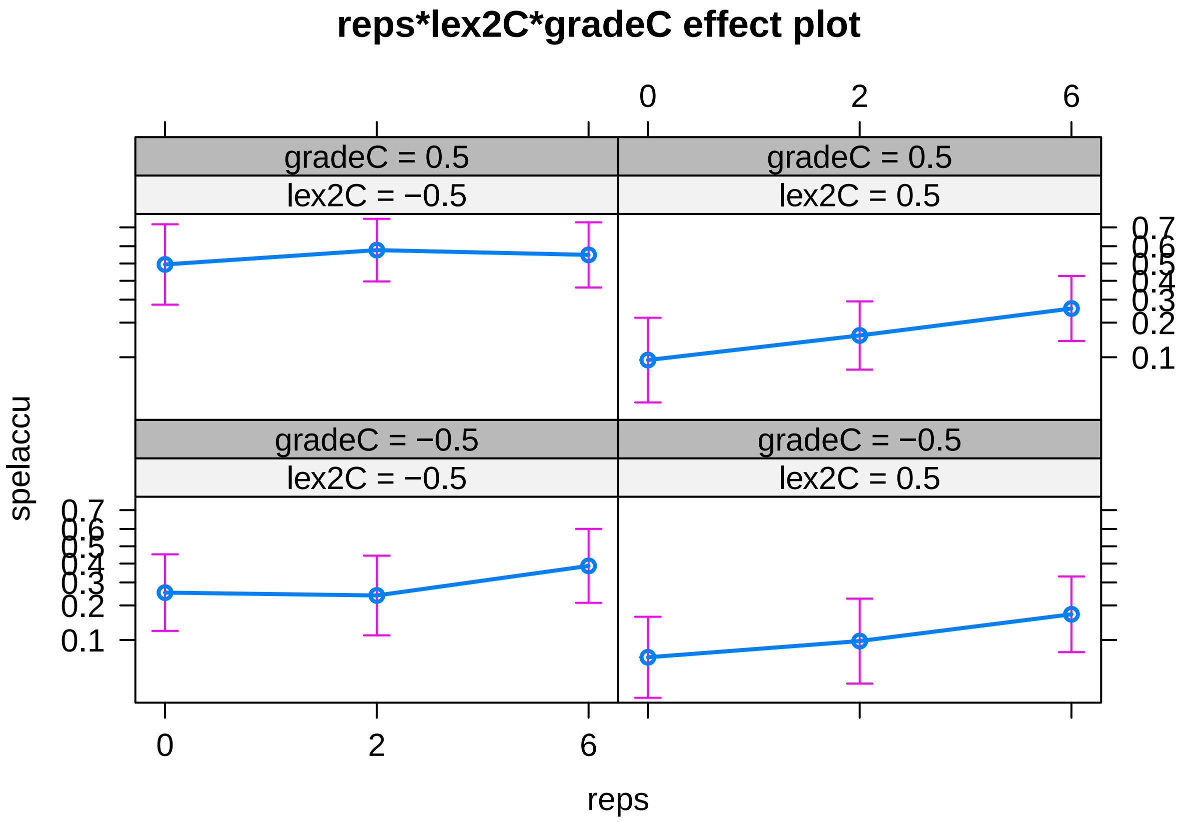

Grades (rows) are: Grade 2 = −0.5, Grade 5 = +0.5. Lexicality levels (columns) are: words = −0.5, pseudowords = +0.5.

And here are the effects from the reduced model (essentially identical).

```
plot(allEffects(sp3c3,xlevels=list(lex2C=c(-0.5,0.5),gradeC=c(-0.5,0.5))))
```

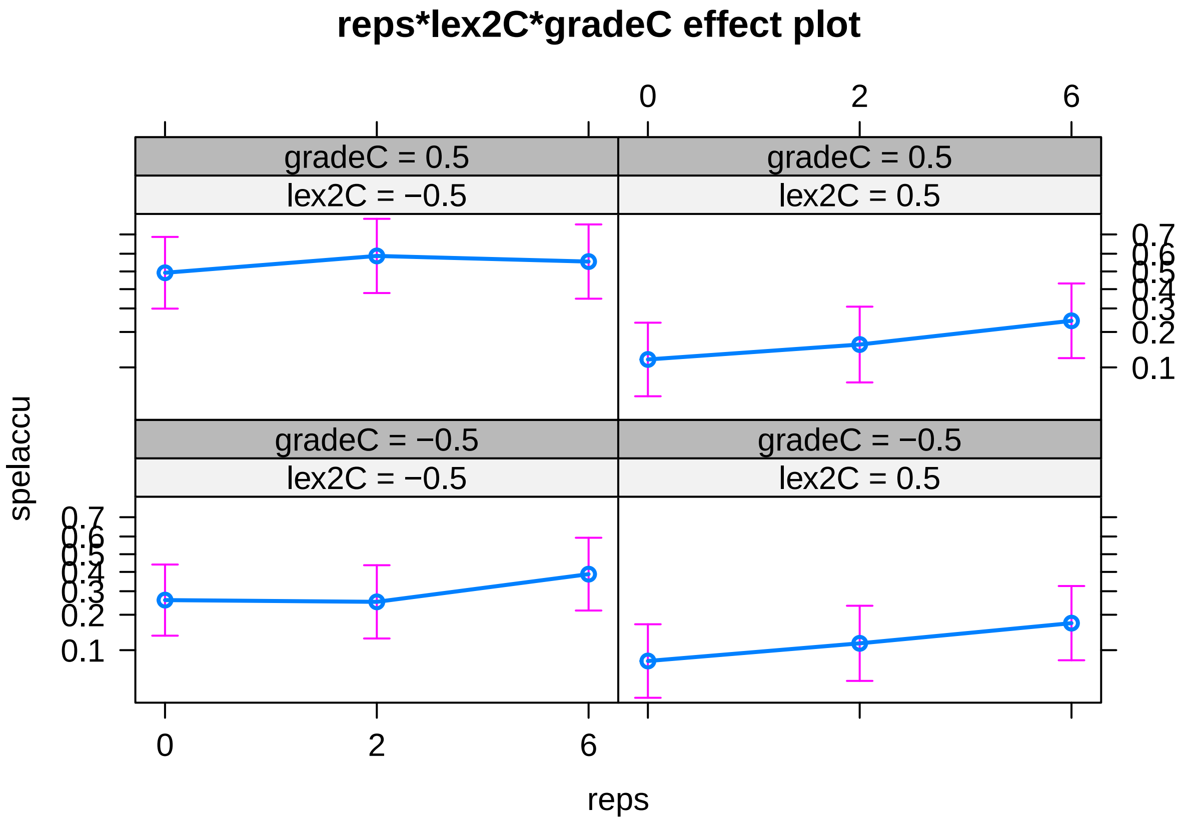

4.5 Power considerations

Looking at the preceding graphs and effects table, effects on spelling seem a bit larger and more consistent (across early and late phases) for pseudowords (right column). The estimates for interaction effects between lexicality and exposure are comparable in magnitude with the main effects of exposure, so quite sizeable, but so are the standard errors of the interaction estimates (around .27 for the full model and .22 for the reduced model), precluding significance.

Applying the same rationale as for the orthographic choice task, namely that power to detect an effect of about 3 SE is 85%, this means that we would have sufficient power to detect a two-way interaction with the reduced model if the effect estimate was .66 or more. This is about half the estimated main effect of lexicality and comparable to the overall learning effect (the sum of the main effects of early and late learning). In terms of accuracy, and given a grand-mean intercept of about -1.1 (hence average accuracy of 0.25), this means that the three-way interaction effect should bring accuracy to 0.39 or 0.15. Thus, we can conclude that we have sufficient power to detect relatively large interaction effects that involve successive differences in number of exposures.

4.6 Modeling the overall learning effect

As with the analysis of the orthographic choice outcome, a more powerful approach to detecting interactions with exposure for the spelling outcome involves discarding the 2-exposures condition and modeling the total learning effect, that is, the difference between zero and six exposures. This is necessarily a larger effect than either of the intermediate ones modeled above, so it provides a higher-magnitude baseline over which interactions may be detected.

```
spfrmt <- as.formula( "spelaccu ~ 1 + repsC*lex2C*gradeC + (repsC*lex2C|sID) + (repsC*gradeC|targetS)" )
sp3ct <- glmer( spfrmt, som, family=binomial, control=glmerControl(optCtrl=list(maxfun=1e5), optimizer =
```

```
## boundary (singular) fit: see ?isSingular
```

```
#load("sp3ct.Rda")
print(summary(sp3ct),corr=F)
```

```
## Generalized linear mixed model fit by maximum likelihood (Laplace
##   Approximation) [glmerMod]
##  Family: binomial  ( logit )
## Formula: spelaccu ~ 1 + repsC * lex2C * gradeC + (repsC * lex2C | sID) +
##     (repsC * gradeC | targetS)
##    Data: som
## Control: glmerControl(optCtrl = list(maxfun = 1e+05), optimizer = "bobyqa")
##
##      AIC      BIC   logLik deviance df.resid
##   2910.6   3079.2  -1427.3   2854.6     3020
##
## Scaled residuals:
##     Min      1Q  Median      3Q     Max
## -3.8136 -0.5402 -0.2429  0.5469  4.8450
##
## Random effects:
##  Groups  Name         Variance Std.Dev. Corr
##  sID     (Intercept)  0.21653  0.4653
##          repsC        0.17423  0.4174   -0.10
##          lex2C        0.15713  0.3964    0.00 -1.00
##          repsC:lex2C  0.02387  0.1545   -0.98  0.32 -0.22
##  targetS (Intercept)  2.32174  1.5237
##          repsC        0.09937  0.3152   -0.93
##          gradeC       0.90552  0.9516   -0.11  0.43
##          repsC:gradeC 0.71641  0.8464   -0.63  0.78  0.29
## Number of obs: 3048, groups:  sID, 127; targetS, 32
##
## Fixed effects:
##                     Estimate Std. Error z value Pr(>|z|)
## (Intercept)         -1.09449    0.28043  -3.903 9.51e-05 ***
## repsC                0.74335    0.13422   5.538 3.06e-08 ***
## lex2C               -1.46499    0.55508  -2.639  0.00831 **
## gradeC               0.62653    0.22264   2.814  0.00489 **
## repsC:lex2C          0.66130    0.24906   2.655  0.00793 **
## repsC:gradeC        -0.07201    0.28054  -0.257  0.79743
## lex2C:gradeC        -0.38551    0.41552  -0.928  0.35351
## repsC:lex2C:gradeC  0.61159    0.52580   1.163  0.24476
## ---
## Signif. codes:  0 '***' 0.001 '**' 0.01 '*' 0.05 '.' 0.1 ' ' 1
## optimizer (bobyqa) convergence code: 0 (OK)
## boundary (singular) fit: see ?isSingular
```

Lexicality (but not grade) interacts significantly with exposure in this model. This fit is also singular, which suggests that the random structure has been overparameterized. So we refit after forcing the random effects to be uncorrelated and iteratively removing the negligible random effects.

```
spfrmt3 <- as.formula( "spelaccu ~ 1 + repsC*lex2C*gradeC + (repsC+lex2C||sID) + (gradeC||targetS)" )
sp3ct3 <- glmer( spfrmt3, som, family=binomial, control=glmerControl(optCtrl=list(maxfun=1e5), optimize
print(summary(sp3ct3),corr=F)
```

```
## Generalized linear mixed model fit by maximum likelihood (Laplace
```

```
##   Approximation) [glmerMod]
##  Family: binomial  ( logit )
## Formula: spelaccu ~ 1 + repsC * lex2C * gradeC + (repsC + lex2C || sID) +
##     (gradeC || targetS)
##    Data: som
## Control: glmerControl(optCtrl = list(maxfun = 1e+05), optimizer = "bobyqa")
##
##      AIC      BIC   logLik deviance df.resid
##   2893.7   2972.0  -1433.9   2867.7     3035
##
## Scaled residuals:
##     Min      1Q  Median      3Q     Max
## -3.9982 -0.5359 -0.2537  0.5481  5.1688
##
## Random effects:
##  Groups    Name        Variance Std.Dev.
##  sID       (Intercept) 0.2143   0.4629
##  sID.1     repsC       0.1052   0.3243
##  sID.2     lex2C       0.0737   0.2715
##  targetS   (Intercept) 2.3364   1.5285
##  targetS.1 gradeC      0.8516   0.9228
## Number of obs: 3048, groups:  sID, 127; targetS, 32
##
## Fixed effects:
##                    Estimate Std. Error z value Pr(>|z|)
## (Intercept)         -1.0706     0.2809  -3.812 0.000138 ***
## repsC                0.6657     0.1059   6.288 3.22e-10 ***
## lex2C               -1.4205     0.5552  -2.559 0.010509 *
## gradeC               0.6274     0.2160   2.905 0.003678 **
## repsC:lex2C          0.4673     0.2013   2.321 0.020292 *
## repsC:gradeC        -0.1551     0.2099  -0.739 0.459870
## lex2C:gradeC        -0.3781     0.4003  -0.944 0.344931
## repsC:lex2C:gradeC   0.3751     0.4016   0.934 0.350342
## ---
## Signif. codes:  0 '***' 0.001 '**' 0.01 '*' 0.05 '.' 0.1 ' ' 1
```

```
anova(sp3ct,sp3ct3)
```

```
## Data: som
## Models:
## sp3ct3: spelaccu ~ 1 + repsC * lex2C * gradeC + (repsC + lex2C || sID) + (gradeC || targetS)
## sp3ct: spelaccu ~ 1 + repsC * lex2C * gradeC + (repsC * lex2C | sID) + (repsC * gradeC | targetS)
##        npar    AIC    BIC  logLik deviance  Chisq Df Pr(>Chisq)
## sp3ct3   13 2893.7 2972.0 -1433.9   2867.7
## sp3ct    28 2910.6 3079.2 -1427.3   2854.6 13.104 15     0.5943
```

The latter model seems to have converged fine, and the fit is no worse than that of the model with the fuller random structure. Standard errors for the interactions are similar in magnitude as in the model with all three exposure conditions. The interaction between lexicality and exposure remains statistically significant, suggesting that lexicality affects orthographic learning rate such that there is a larger overall effect of exposure on learning novel spellings than on learning spelling of potentially/partially known words.

Here are the estimates of the original and reduced model, side by side for comparability:

```
cbind(round(coef(summary(sp3ct)),3),round(coef(summary(sp3ct3)),3)) -> cbspt13
colnames(cbspt13) <- rep(c("Estim","Std.Err","z","p"),2)
```

```
cbspt13
```

```
##                        Estim Std.Err      z     p  Estim Std.Err      z     p
## (Intercept)           -1.094   0.280 -3.903 0.000 -1.071   0.281 -3.812 0.000
## repsC                  0.743   0.134  5.538 0.000  0.666   0.106  6.288 0.000
## lex2C                 -1.465   0.555 -2.639 0.008 -1.420   0.555 -2.559 0.011
## gradeC                 0.627   0.223  2.814 0.005  0.627   0.216  2.905 0.004
## repsC:lex2C            0.661   0.249  2.655 0.008  0.467   0.201  2.321 0.020
## repsC:gradeC          -0.072   0.281 -0.257 0.797 -0.155   0.210 -0.739 0.460
## lex2C:gradeC          -0.386   0.416 -0.928 0.354 -0.378   0.400 -0.944 0.345
## repsC:lex2C:gradeC     0.612   0.526  1.163 0.245  0.375   0.402  0.934 0.350
```

Here are the model effects:

```
plot(allEffects(sp3ct3,xlevels=list(repsC=c(-0.5,0.5),lex2C=c(-0.5,0.5),gradeC=c(-0.5,0.5))))
```

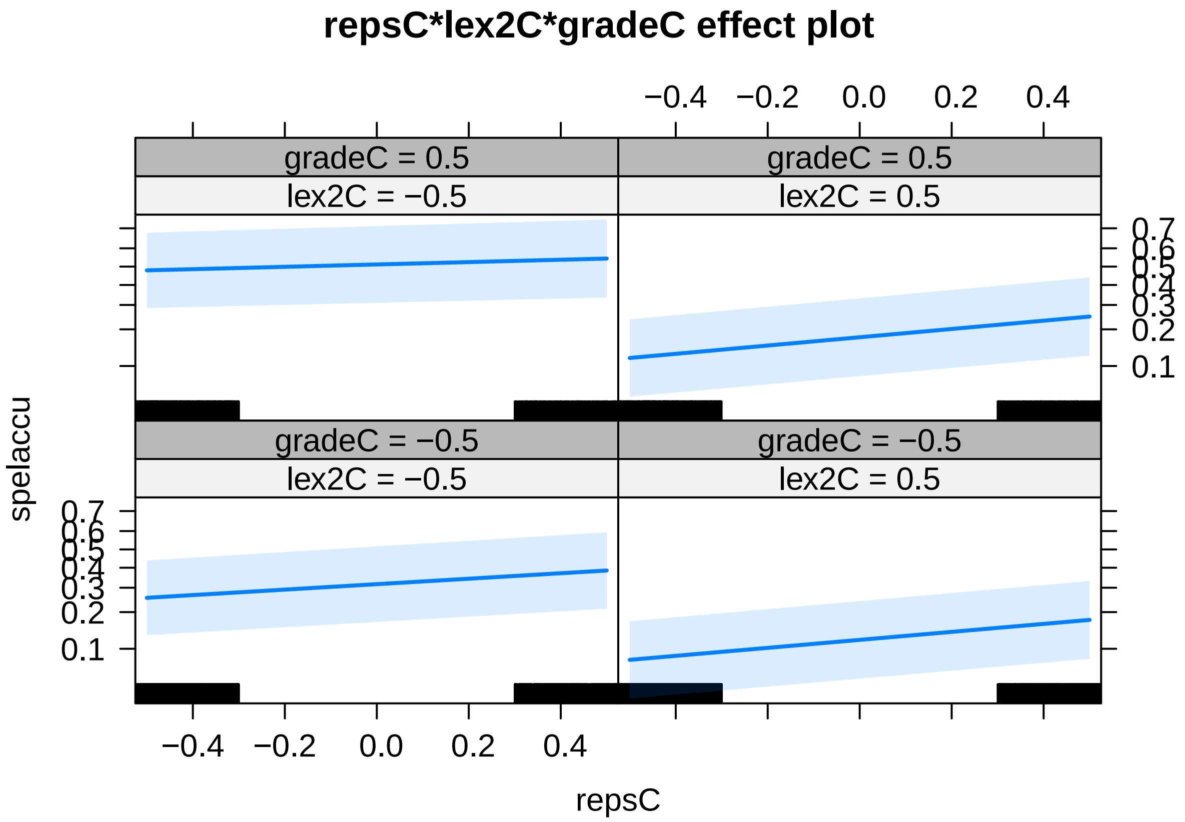

The steeper slopes on the right (i.e., for pseudowords) are quite evident and consistent with the significant interaction between lexicality and exposure (with a just-detectable interaction effect of 0.47).

And here are the fit diagnostics:

```
sst <- simulateResiduals(fittedModel=sp3ct3,plot=F)
testUniformity(sst)
```

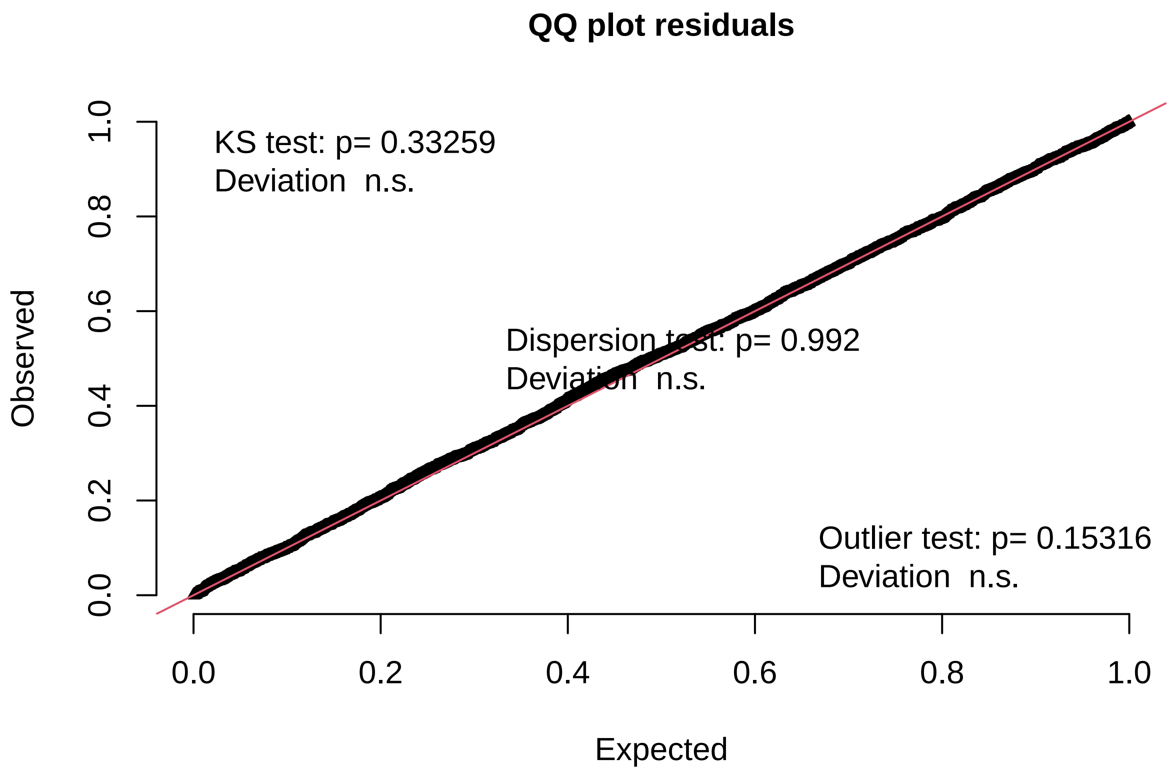


```
##
##  One-sample Kolmogorov-Smirnov test
##
## data:  simulationOutput$scaledResiduals
## D = 0.017133, p-value = 0.3326
## alternative hypothesis: two-sided
```

```
testOutliers(sst)
```

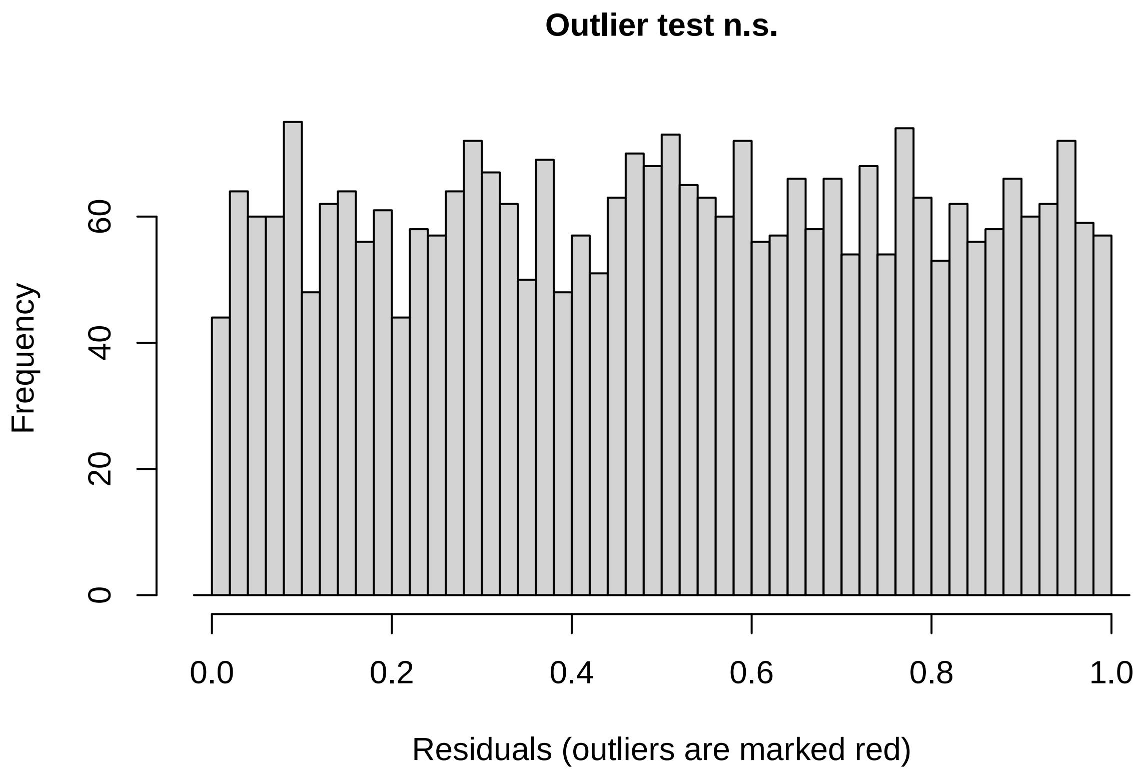


```
##
##  DHARMa outlier test based on exact binomial test with approximate
##  expectations
##
## data:  sst
## outliers at both margin(s) = 17, observations = 3048, p-value = 0.1532
## alternative hypothesis: true probability of success is not equal to 0.007968127
## 95 percent confidence interval:
##  0.003252314 0.008915030
## sample estimates:
## frequency of outliers (expected: 0.00796812749003984 )
##                                            0.005577428
```

```
testDispersion(sst)
```

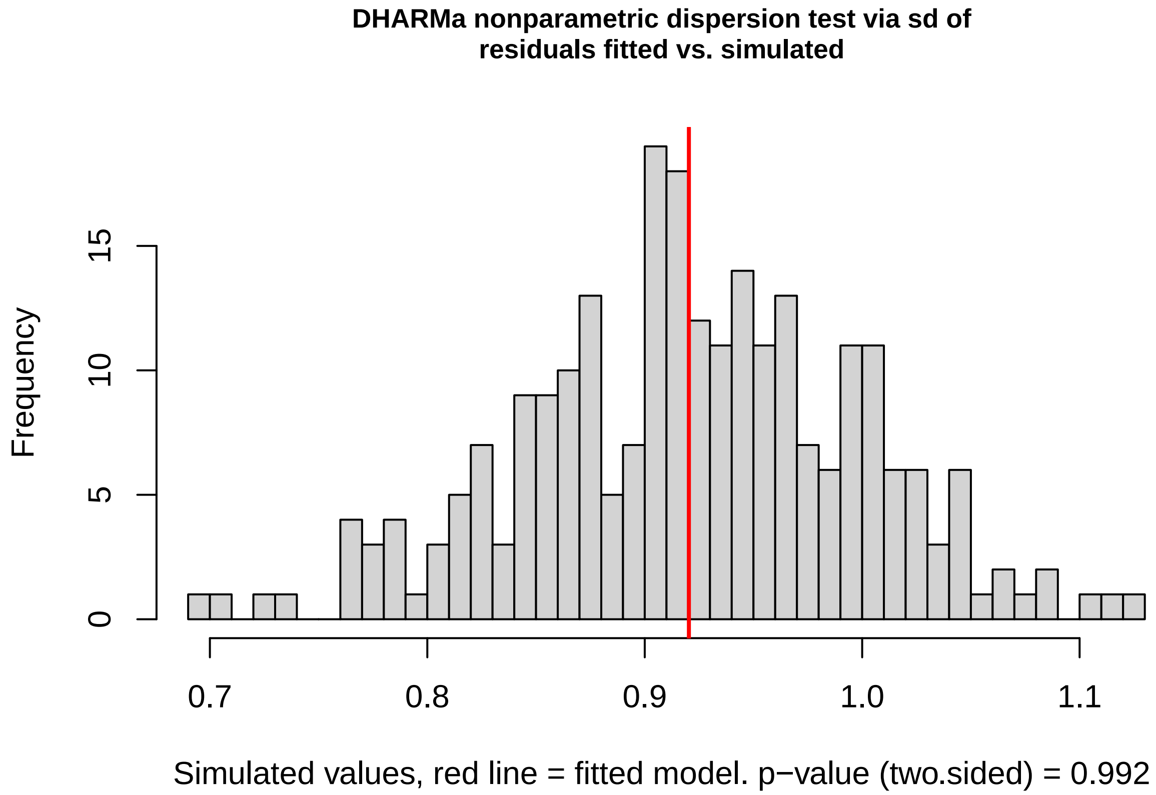


```
##
##  DHARMa nonparametric dispersion test via sd of residuals fitted vs.
##  simulated
##
## data:  simulationOutput
## dispersion = 0.9989, p-value = 0.992
## alternative hypothesis: two.sided
```

```
testZeroInflation(sst)
```

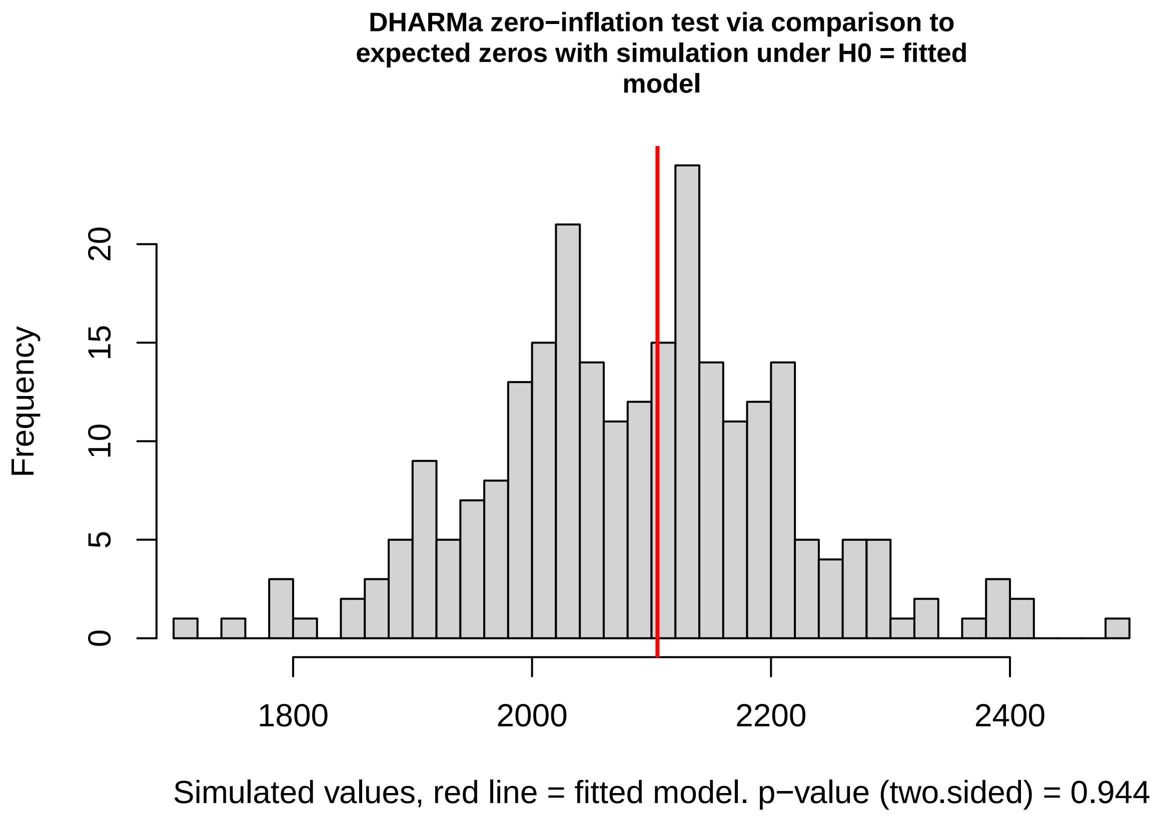


```
##
##  DHARMa zero-inflation test via comparison to expected zeros with
##  simulation under H0 = fitted model
##
## data:  simulationOutput
## ratioObsSim = 1.0085, p-value = 0.944
## alternative hypothesis: two.sided
```

4.7 Checking for effects of length

Models can also be fit to include effects of length, starting with all factors fully interacting. Random slopes for the interactions of all three factors (grade, lexicality, length) with exposure (reps) are included, but no higher-order interactions. Fixed-effects factors are allowed to interact fully.

```
spfn2 <- as.formula("spelaccu ~ 1 + reps*lex2C*gradeC*Nsyl2C + (1|sID) + (0+(reps6_2+reps2_0)*lex2C|sID)
spn2  <- glmer( spfn2, som, family=binomial, control=glmerControl(optCtrl=list(maxfun=1e5), optimizer =
```

```
## boundary (singular) fit: see ?isSingular
```

```
print(summary(spn2),corr=F)
```

```
## Generalized linear mixed model fit by maximum likelihood (Laplace
##   Approximation) [glmerMod]
##  Family: binomial  ( logit )
## Formula: spelaccu ~ 1 + reps * lex2C * gradeC * Nsyl2C + (1 | sID) + (0 +
##     (reps6_2 + reps2_0) * lex2C | sID) + (0 + (reps6_2 + reps2_0) *
##     Nsyl2C | sID) + (1 | targetS) + (0 + (reps6_2 + reps2_0) *
##     gradeC | targetS)
```

```
##    Data: som
## Control: glmerControl(optCtrl = list(maxfun = 1e+05), optimizer = "bobyqa")
##
##      AIC      BIC   logLik deviance df.resid
##   3930.4   4378.4  -1894.2   3788.4     3992
##
## Scaled residuals:
##     Min      1Q  Median      3Q     Max
## -4.2867 -0.5259 -0.2435  0.4970  5.0674
##
## Random effects:
##  Groups    Name           Variance Std.Dev. Corr
##  sID       (Intercept)    0.162867 0.40357
##  sID.1     reps6_2        0.162829 0.40352
##            reps2_0        0.190639 0.43662  -0.35
##            lex2C          0.215021 0.46370  -0.71 -0.42
##            reps6_2:lex2C  0.781650 0.88411  -0.53  0.98 -0.22
##            reps2_0:lex2C  0.635918 0.79744   0.40 -1.00  0.36 -0.99
##  sID.2     reps6_2        0.405233 0.63658
##            reps2_0        0.251360 0.50136  -0.94
##            Nsyl2C         0.068953 0.26259   0.98 -0.99
##            reps6_2:Nsyl2C 1.092414 1.04519   0.96 -0.82  0.90
##            reps2_0:Nsyl2C 0.232427 0.48211  -0.02 -0.31  0.16 -0.29
##  targetS   (Intercept)    2.357362 1.53537
##  targetS.1 reps6_2        0.004626 0.06802
##            reps2_0        0.036651 0.19145  -1.00
##            gradeC         0.773205 0.87932  -0.17  0.17
##            reps6_2:gradeC 0.249190 0.49919  -0.89  0.89  0.61
##            reps2_0:gradeC 0.087436 0.29570  -0.97  0.97 -0.08  0.75
## Number of obs: 4063, groups:  sID, 127; targetS, 32
##
## Fixed effects:
##                           Estimate Std. Error z value Pr(>|z|)
## (Intercept)               -1.13117    0.27989  -4.041 5.31e-05 ***
## reps2_0                    0.24999    0.13190   1.895 0.058052 .
## reps6_2                    0.37530    0.13879   2.704 0.006848 **
## lex2C                     -1.52283    0.55629  -2.737 0.006191 **
## gradeC                     0.74412    0.20081   3.706 0.000211 ***
## Nsyl2C                     0.04505    0.55465   0.081 0.935271
## reps2_0:lex2C              0.22822    0.24115   0.946 0.343951
## reps6_2:lex2C              0.23859    0.25281   0.944 0.345291
## reps2_0:gradeC             0.20795    0.25506   0.815 0.414904
## reps6_2:gradeC            -0.30375    0.29071  -1.045 0.296089
## lex2C:gradeC              -0.68118    0.38088  -1.788 0.073700 .
## reps2_0:Nsyl2C            -0.03251    0.22881  -0.142 0.887018
## reps6_2:Nsyl2C             0.56611    0.25658   2.206 0.027356 *
## lex2C:Nsyl2C               1.11403    1.10783   1.006 0.314608
## gradeC:Nsyl2C              0.86645    0.37456   2.313 0.020711 *
## reps2_0:lex2C:gradeC      -0.53661    0.46432  -1.156 0.247812
## reps6_2:lex2C:gradeC       0.98791    0.53120   1.860 0.062915 .
## reps2_0:lex2C:Nsyl2C       0.07101    0.44790   0.159 0.874039
## reps6_2:lex2C:Nsyl2C       0.26581    0.47484   0.560 0.575619
## reps2_0:gradeC:Nsyl2C      0.73162    0.44659   1.638 0.101371
## reps6_2:gradeC:Nsyl2C     -0.49072    0.54136  -0.906 0.364695
```

```
## lex2C:gradeC:Nsyl2C         -0.41783    0.74226  -0.563 0.573486
## reps2_0:lex2C:gradeC:Nsyl2C -0.31685    0.87517  -0.362 0.717321
## reps6_2:lex2C:gradeC:Nsyl2C -1.42649    1.01342  -1.408 0.159251
## ---
## Signif. codes:  0 '***' 0.001 '**' 0.01 '*' 0.05 '.' 0.1 ' ' 1
## optimizer (bobyqa) convergence code: 0 (OK)
## boundary (singular) fit: see ?isSingular
```

This leads to singular fit due to overparameterized random structure. With subsequent reductions as above we can reach a reduced model with nonsingular fit.

```
spfn4 <- as.formula("spelaccu ~ 1 + reps*lex2C*gradeC*Nsyl2C + (1+reps2_0+reps6_2+lex2C||sID) + (1+grad
spn4  <- glmer( spfn4, som, family=binomial, control=glmerControl(optCtrl=list(maxfun=1e5), optimizer =
print(summary(spn4),corr=F)
```

```
## Generalized linear mixed model fit by maximum likelihood (Laplace
##   Approximation) [glmerMod]
##  Family: binomial  ( logit )
## Formula: spelaccu ~ 1 + reps * lex2C * gradeC * Nsyl2C + (1 + reps2_0 +
##     reps6_2 + lex2C || sID) + (1 + gradeC || targetS)
##    Data: som
## Control: glmerControl(optCtrl = list(maxfun = 1e+05), optimizer = "bobyqa")
##
##      AIC      BIC   logLik deviance df.resid
##   3876.4   4065.7  -1908.2   3816.4     4033
##
## Scaled residuals:
##     Min      1Q  Median      3Q     Max
## -4.5982 -0.5467 -0.2571  0.5475  6.0506
##
## Random effects:
##  Groups    Name        Variance Std.Dev.
##  sID       (Intercept) 0.15412  0.3926
##  sID.1     reps2_0     0.09061  0.3010
##  sID.2     reps6_2     0.07725  0.2779
##  sID.3     lex2C       0.08084  0.2843
##  targetS   (Intercept) 2.20323  1.4843
##  targetS.1 gradeC      0.69136  0.8315
## Number of obs: 4063, groups:  sID, 127; targetS, 32
##
## Fixed effects:
##                              Estimate Std. Error z value Pr(>|z|)
## (Intercept)                 -1.087946   0.270807  -4.017 5.88e-05 ***
## reps2_0                      0.258652   0.108062   2.394 0.016686 *
## reps6_2                      0.363463   0.117199   3.101 0.001927 **
## lex2C                       -1.476233   0.537221  -2.748 0.005998 **
## gradeC                       0.712316   0.192360   3.703 0.000213 ***
## Nsyl2C                       0.060304   0.536088   0.112 0.910435
## reps2_0:lex2C                0.208903   0.207728   1.006 0.314581
## reps6_2:lex2C                0.239669   0.228809   1.047 0.294886
## reps2_0:gradeC               0.178233   0.215187   0.828 0.407517
## reps6_2:gradeC              -0.345838   0.234169  -1.477 0.139709
## lex2C:gradeC                -0.618476   0.359071  -1.722 0.084991 .
## reps2_0:Nsyl2C              -0.042249   0.205955  -0.205 0.837466
## reps6_2:Nsyl2C               0.583218   0.228662   2.551 0.010755 *
```

```
## lex2C:Nsyl2C                 1.090684   1.072014   1.017 0.308955
## gradeC:Nsyl2C                0.801021   0.355295   2.255 0.024163 *
## reps2_0:lex2C:gradeC        -0.485801   0.414971  -1.171 0.241725
## reps6_2:lex2C:gradeC         0.927392   0.457438   2.027 0.042626 *
## reps2_0:lex2C:Nsyl2C        -0.004583   0.412442  -0.011 0.991133
## reps6_2:lex2C:Nsyl2C         0.318776   0.457409   0.697 0.485855
## reps2_0:gradeC:Nsyl2C        0.628604   0.412014   1.526 0.127089
## reps6_2:gradeC:Nsyl2C       -0.502146   0.457021  -1.099 0.271882
## lex2C:gradeC:Nsyl2C         -0.391176   0.710305  -0.551 0.581829
## reps2_0:lex2C:gradeC:Nsyl2C -0.379346   0.824841  -0.460 0.645586
## reps6_2:lex2C:gradeC:Nsyl2C -1.319832   0.914532  -1.443 0.148971
## ---
## Signif. codes:  0 '***' 0.001 '**' 0.01 '*' 0.05 '.' 0.1 ' ' 1
```

The model with reduced random structure is no worse than the original model with all random slopes:

```
anova(spn2,spn4)
```

```
## Data: som
## Models:
## spn4: spelaccu ~ 1 + reps * lex2C * gradeC * Nsyl2C + (1 + reps2_0 + reps6_2 + lex2C || sID) + (1 + g
## spn2: spelaccu ~ 1 + reps * lex2C * gradeC * Nsyl2C + (1 | sID) + (0 + (reps6_2 + reps2_0) * lex2C |
##      npar    AIC    BIC  logLik deviance  Chisq Df Pr(>Chisq)
## spn4   30 3876.4 4065.7 -1908.2   3816.4
## spn2   71 3930.4 4378.4 -1894.2   3788.4 27.965 41     0.9398
```

There is no appreciable difference between the reduced model and the original model when it comes to detecting significant two-way interactions with reps.

Here is its Anova table:

```
Anova(spn4)
```

```
## Analysis of Deviance Table (Type II Wald chisquare tests)
##
## Response: spelaccu
##                              Chisq Df Pr(>Chisq)
## reps                       35.2710  2  2.193e-08 ***
## lex2C                       7.7063  1  0.0055029 **
## gradeC                     14.7051  1  0.0001257 ***
## Nsyl2C                      0.0042  1  0.9483741
## reps:lex2C                  4.6364  2  0.0984502 .
## reps:gradeC                 2.8501  2  0.2405000
## lex2C:gradeC                2.9563  1  0.0855424 .
## reps:Nsyl2C                 8.4234  2  0.0148214 *
## lex2C:Nsyl2C                1.0229  1  0.3118416
## gradeC:Nsyl2C               4.2801  1  0.0385603 *
## reps:lex2C:gradeC           3.6425  2  0.1618205
## reps:lex2C:Nsyl2C           1.1454  2  0.5640141
## reps:gradeC:Nsyl2C          2.7096  2  0.2579957
## lex2C:gradeC:Nsyl2C         0.1665  1  0.6832226
## reps:lex2C:gradeC:Nsyl2C  4.5900  2  0.1007635
## ---
## Signif. codes:  0 '***' 0.001 '**' 0.01 '*' 0.05 '.' 0.1 ' ' 1
```

Here are the estimates of the original and reduced model, side by side for comparability:

```r
cbind(round(coef(summary(spn2)),3),round(coef(summary(spn4)),3)) -> cbspn24
colnames(cbspn24) <- rep(c("Estim","Std.Err","z","p"),2)
cbspn24[1:(nrow(cbspn24)-2),] # omit 4-way interactions to limit display width
```

```
##                         Estim Std.Err      z     p  Estim Std.Err      z     p
## (Intercept)            -1.131   0.280 -4.041 0.000 -1.088   0.271 -4.017 0.000
## reps2_0                 0.250   0.132  1.895 0.058  0.259   0.108  2.394 0.017
## reps6_2                 0.375   0.139  2.704 0.007  0.363   0.117  3.101 0.002
## lex2C                  -1.523   0.556 -2.737 0.006 -1.476   0.537 -2.748 0.006
## gradeC                  0.744   0.201  3.706 0.000  0.712   0.192  3.703 0.000
## Nsyl2C                  0.045   0.555  0.081 0.935  0.060   0.536  0.112 0.910
## reps2_0:lex2C           0.228   0.241  0.946 0.344  0.209   0.208  1.006 0.315
## reps6_2:lex2C           0.239   0.253  0.944 0.345  0.240   0.229  1.047 0.295
## reps2_0:gradeC          0.208   0.255  0.815 0.415  0.178   0.215  0.828 0.408
## reps6_2:gradeC         -0.304   0.291 -1.045 0.296 -0.346   0.234 -1.477 0.140
## lex2C:gradeC           -0.681   0.381 -1.788 0.074 -0.618   0.359 -1.722 0.085
## reps2_0:Nsyl2C         -0.033   0.229 -0.142 0.887 -0.042   0.206 -0.205 0.837
## reps6_2:Nsyl2C          0.566   0.257  2.206 0.027  0.583   0.229  2.551 0.011
## lex2C:Nsyl2C            1.114   1.108  1.006 0.315  1.091   1.072  1.017 0.309
## gradeC:Nsyl2C           0.866   0.375  2.313 0.021  0.801   0.355  2.255 0.024
## reps2_0:lex2C:gradeC   -0.537   0.464 -1.156 0.248 -0.486   0.415 -1.171 0.242
## reps6_2:lex2C:gradeC    0.988   0.531  1.860 0.063  0.927   0.457  2.027 0.043
## reps2_0:lex2C:Nsyl2C    0.071   0.448  0.159 0.874 -0.005   0.412 -0.011 0.991
## reps6_2:lex2C:Nsyl2C    0.266   0.475  0.560 0.576  0.319   0.457  0.697 0.486
## reps2_0:gradeC:Nsyl2C   0.732   0.447  1.638 0.101  0.629   0.412  1.526 0.127
## reps6_2:gradeC:Nsyl2C  -0.491   0.541 -0.906 0.365 -0.502   0.457 -1.099 0.272
## lex2C:gradeC:Nsyl2C    -0.418   0.742 -0.563 0.573 -0.391   0.710 -0.551 0.582
```

The analysis of spelling performance with the length factor included suggests that there is a length effect on orthographic learning rate, namely greater late learning for longer (2-syllable) items.

Effects and diagnostics for the reduced model follow:

```r
plot(allEffects(spn4,xlevels=list(lex2C=c(-0.5,0.5),gradeC=c(-0.5,0.5),Nsyl2C=c(-0.5,0.5))))
```

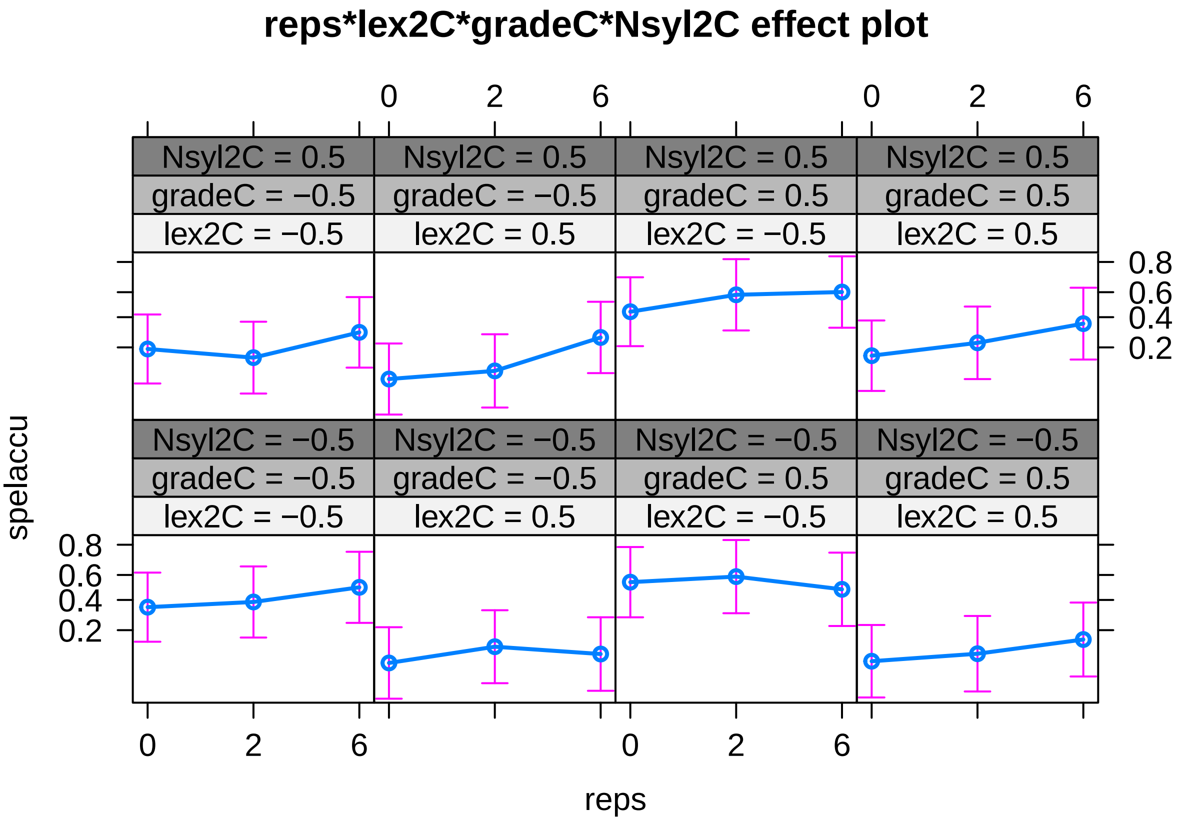

Reminder: Grade (left-right half), Grade 2 = −0.5, Grade 5 = +0.5; Lexicality (columns), words = −0.5, pseudowords = +0.5; Length (rows), 1 syllable = −0.5, 2 syllables +0.5.

And here are the fit diagnostics:

```
sspt4 <- simulateResiduals(fittedModel=spn4,plot=F)
testUniformity(sspt4)
```

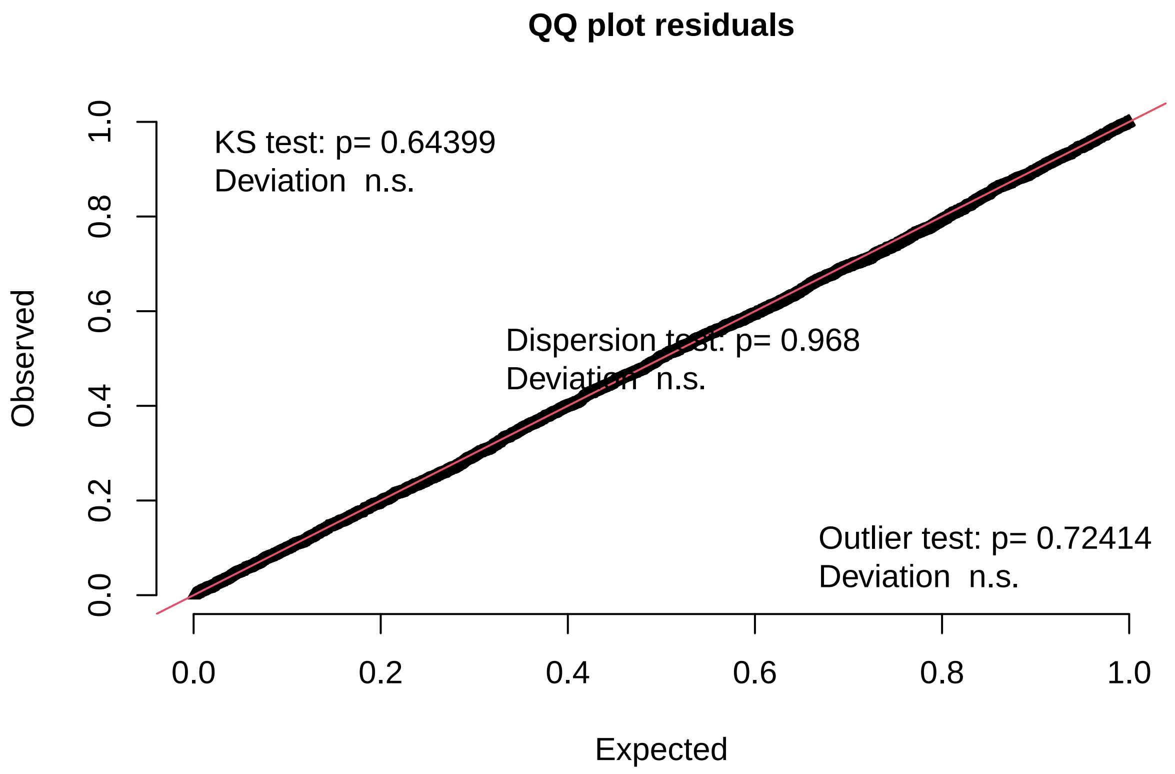


```
##
##  One-sample Kolmogorov-Smirnov test
##
## data:  simulationOutput$scaledResiduals
## D = 0.01161, p-value = 0.644
## alternative hypothesis: two-sided
```

```
testOutliers(sspt4)
```

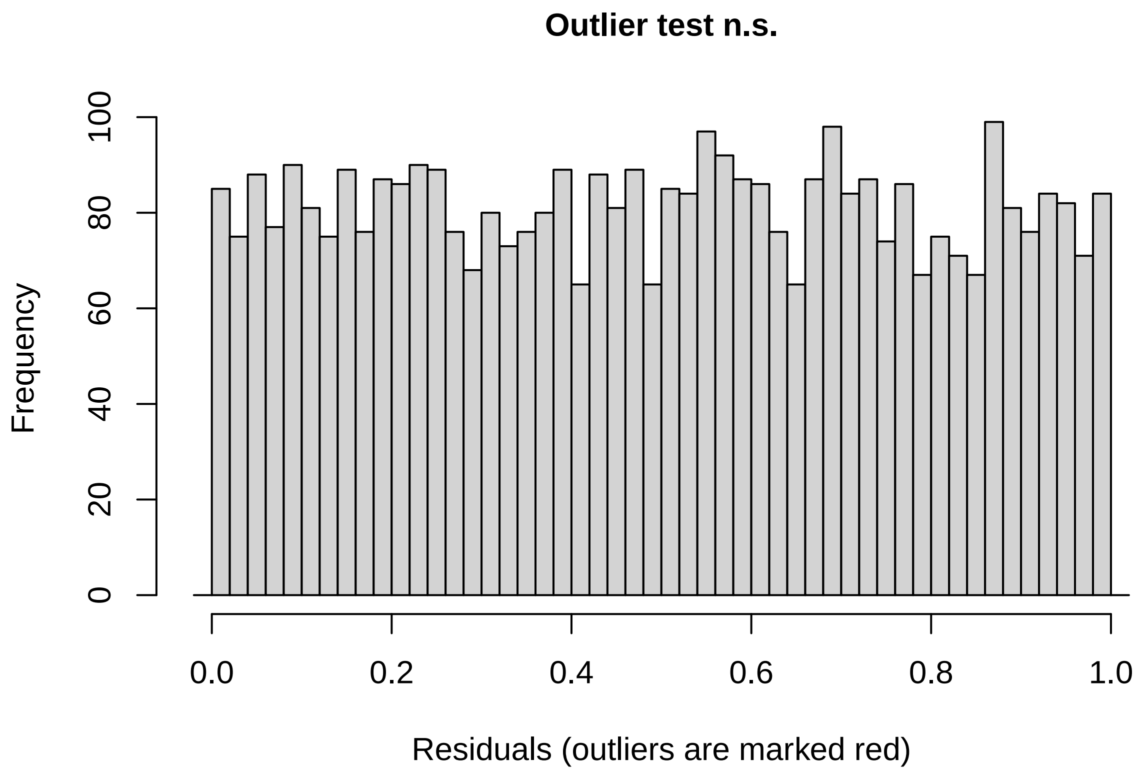


```
##
##  DHARMa outlier test based on exact binomial test with approximate
##  expectations
##
## data:  sspt4
## outliers at both margin(s) = 34, observations = 4063, p-value = 0.7241
## alternative hypothesis: true probability of success is not equal to 0.007968127
## 95 percent confidence interval:
##  0.005801985 0.011674261
## sample estimates:
## frequency of outliers (expected: 0.00796812749003984 )
##                                            0.008368201
```

```
testDispersion(sspt4)
```

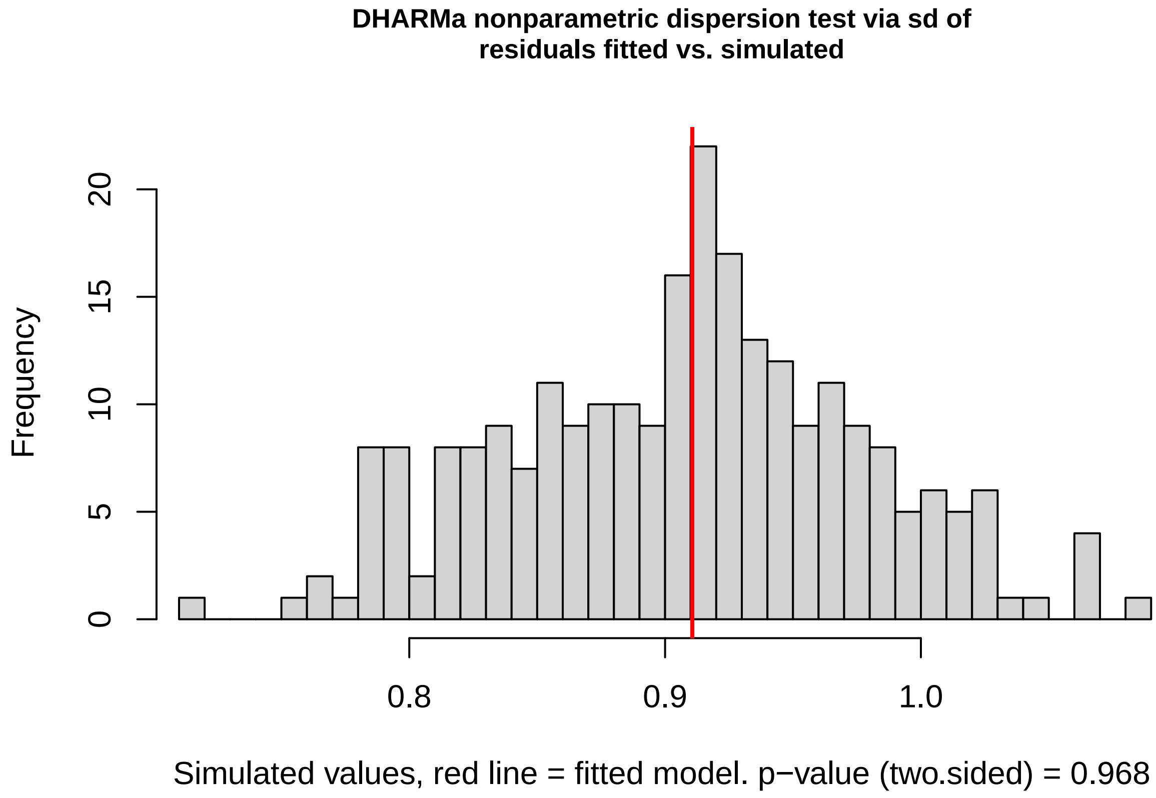


```
##
##  DHARMa nonparametric dispersion test via sd of residuals fitted vs.
##  simulated
##
## data:  simulationOutput
## dispersion = 1.004, p-value = 0.968
## alternative hypothesis: two.sided
```

```
testZeroInflation(sspt4)
```

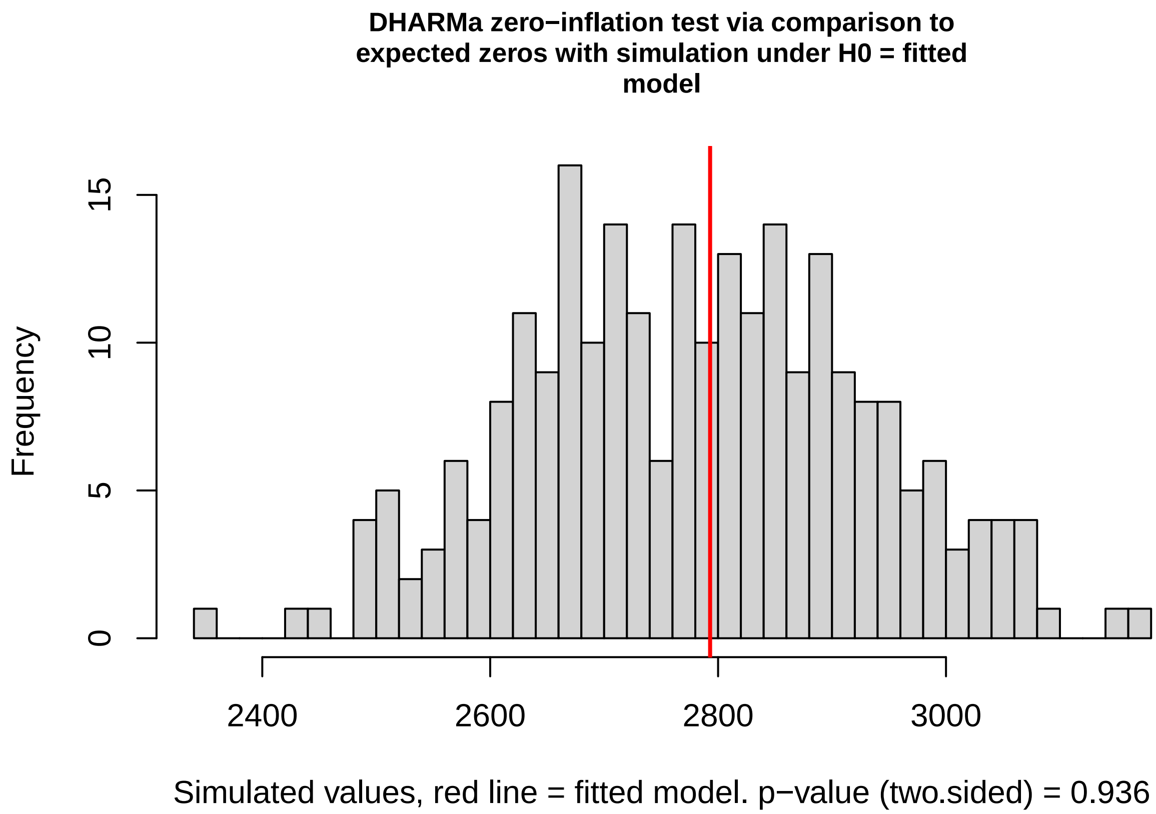


```
##
##  DHARMa zero-inflation test via comparison to expected zeros with
##  simulation under H0 = fitted model
##
## data:  simulationOutput
## ratioObsSim = 1.005, p-value = 0.936
## alternative hypothesis: two.sided
```

We run a final check with the total learning effect (0 vs. 6 exposures) and the length factor included (iterative trimming not shown).

```
spfn5 <- as.formula("spelaccu ~ 1 + repsC*lex2C*gradeC*Nsyl2C + (1+repsC+lex2C||sID) + (1+gradeC||targe
spn5  <- glmer( spfn5, som, family=binomial, control=glmerControl(optCtrl=list(maxfun=1e5), optimizer =
print(summary(spn5),corr=F)
```

```
## Generalized linear mixed model fit by maximum likelihood (Laplace
##   Approximation) [glmerMod]
##  Family: binomial  ( logit )
## Formula: spelaccu ~ 1 + repsC * lex2C * gradeC * Nsyl2C + (1 + repsC +
##     lex2C || sID) + (1 + gradeC || targetS)
##    Data: som
## Control: glmerControl(optCtrl = list(maxfun = 1e+05), optimizer = "bobyqa")
##
##      AIC      BIC   logLik deviance df.resid
##   2894.1   3020.5  -1426.0   2852.1     3027
##
## Scaled residuals:
```

```
##     Min      1Q  Median      3Q     Max
## -4.2729 -0.5284 -0.2505  0.5287  5.2832
##
## Random effects:
##  Groups    Name        Variance Std.Dev.
##  sID       (Intercept) 0.2207   0.4698
##  sID.1     repsC       0.1152   0.3393
##  sID.2     lex2C       0.0776   0.2786
##  targetS   (Intercept) 2.2620   1.5040
##  targetS.1 gradeC      0.7803   0.8833
## Number of obs: 3048, groups:  sID, 127; targetS, 32
##
## Fixed effects:
##                           Estimate Std. Error z value Pr(>|z|)
## (Intercept)                -1.0792     0.2769  -3.898 9.70e-05 ***
## repsC                       0.6379     0.1073   5.944 2.78e-09 ***
## lex2C                      -1.4237     0.5468  -2.603  0.00923 **
## gradeC                      0.6066     0.2115   2.868  0.00413 **
## Nsyl2C                      0.1568     0.5458   0.287  0.77393
## repsC:lex2C                 0.4676     0.2038   2.294  0.02179 *
## repsC:gradeC               -0.1709     0.2129  -0.803  0.42199
## lex2C:gradeC               -0.3514     0.3894  -0.902  0.36685
## repsC:Nsyl2C                0.5435     0.2024   2.686  0.00724 **
## lex2C:Nsyl2C                1.0519     1.0910   0.964  0.33498
## gradeC:Nsyl2C               0.6558     0.3860   1.699  0.08935 .
## repsC:lex2C:gradeC          0.4742     0.4067   1.166  0.24368
## repsC:lex2C:Nsyl2C          0.3142     0.4051   0.776  0.43789
## repsC:gradeC:Nsyl2C         0.1412     0.4037   0.350  0.72654
## lex2C:gradeC:Nsyl2C        -0.5508     0.7712  -0.714  0.47506
## repsC:lex2C:gradeC:Nsyl2C  -1.7280     0.8092  -2.135  0.03272 *
## ---
## Signif. codes:  0 '***' 0.001 '**' 0.01 '*' 0.05 '.' 0.1 ' ' 1
```

This model confirms both interactions of exposure seen previously, namely with lexicality (more learning for pseudowords) and length (more learning for longer items).

```
plot(allEffects(spn5,xlevels=list(repsC=c(-0.5,0.5),lex2C=c(-0.5,0.5),gradeC=c(-0.5,0.5),Nsyl2C=c(-0.5,
```

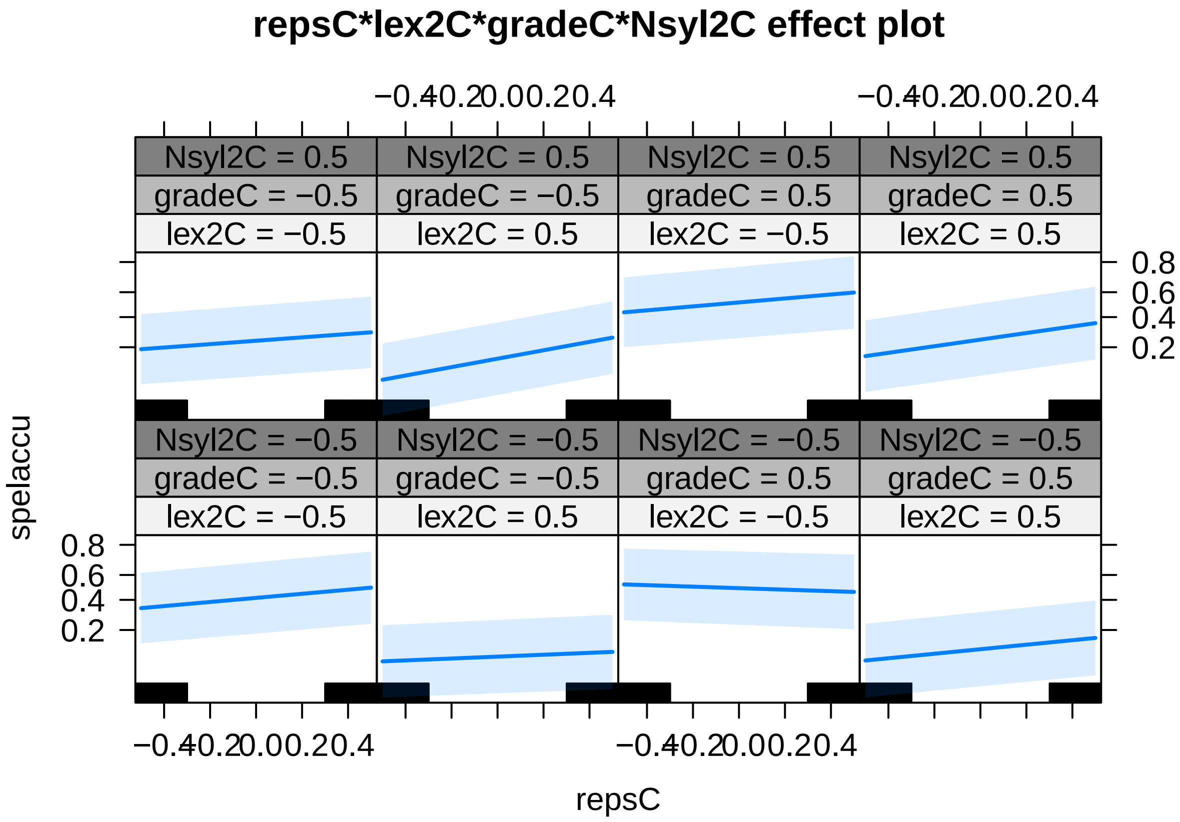


```r
sspt5 <- simulateResiduals(fittedModel=spn5,plot=F)
testUniformity(sspt5)
```

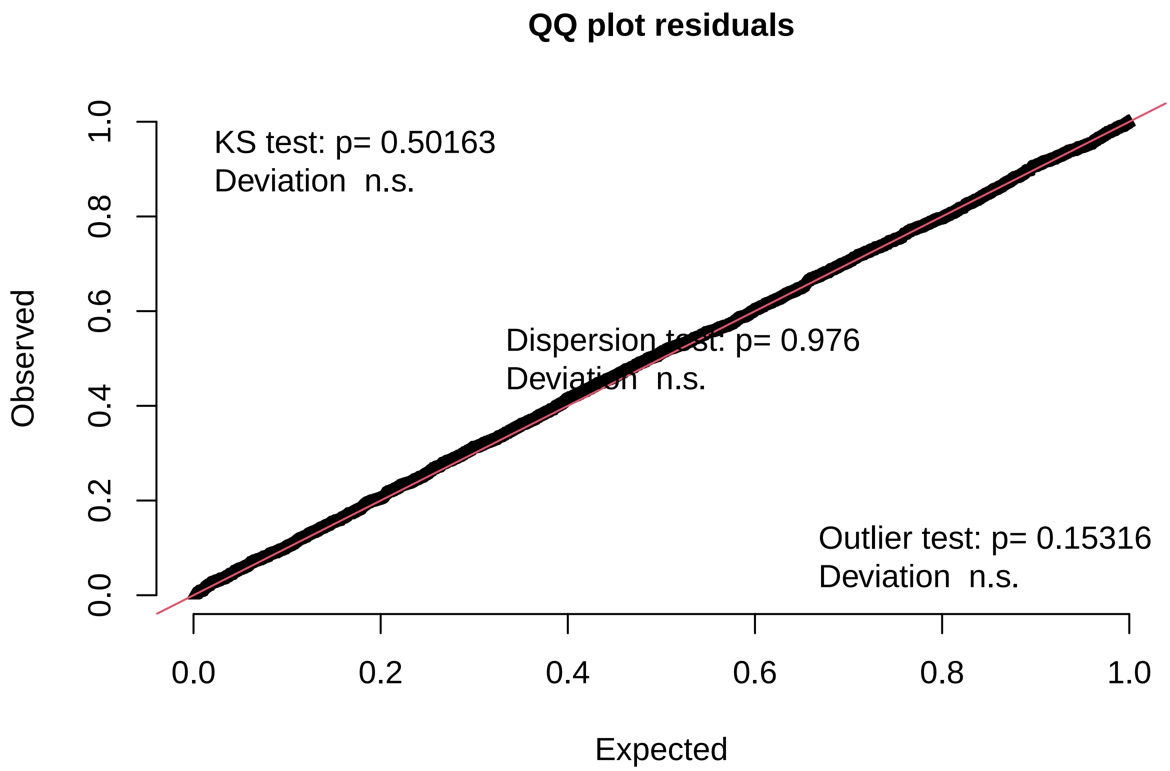


```
##
##  One-sample Kolmogorov-Smirnov test
##
## data:  simulationOutput$scaledResiduals
## D = 0.014971, p-value = 0.5016
## alternative hypothesis: two-sided
```

```
testOutliers(sspt5)
```

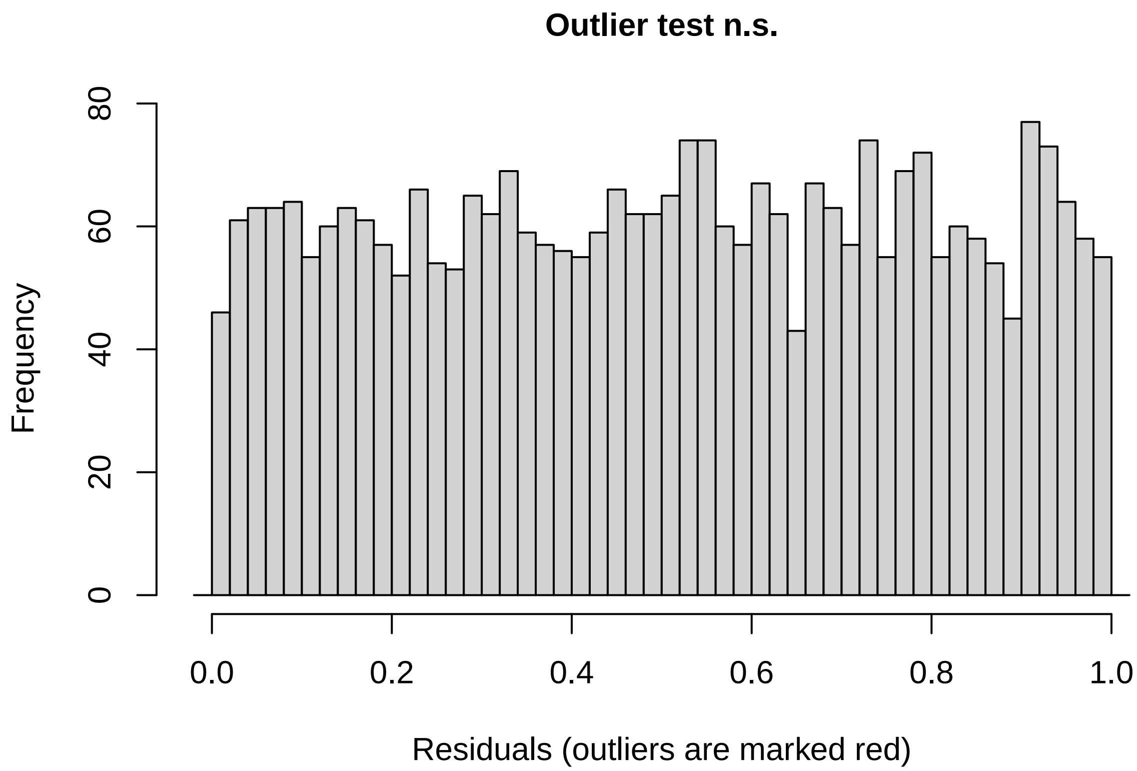


```
##
##  DHARMa outlier test based on exact binomial test with approximate
##  expectations
##
## data:  sspt5
## outliers at both margin(s) = 17, observations = 3048, p-value = 0.1532
## alternative hypothesis: true probability of success is not equal to 0.007968127
## 95 percent confidence interval:
##  0.003252314 0.008915030
## sample estimates:
## frequency of outliers (expected: 0.00796812749003984 )
##                                           0.005577428
```

```
testDispersion(sspt5)
```

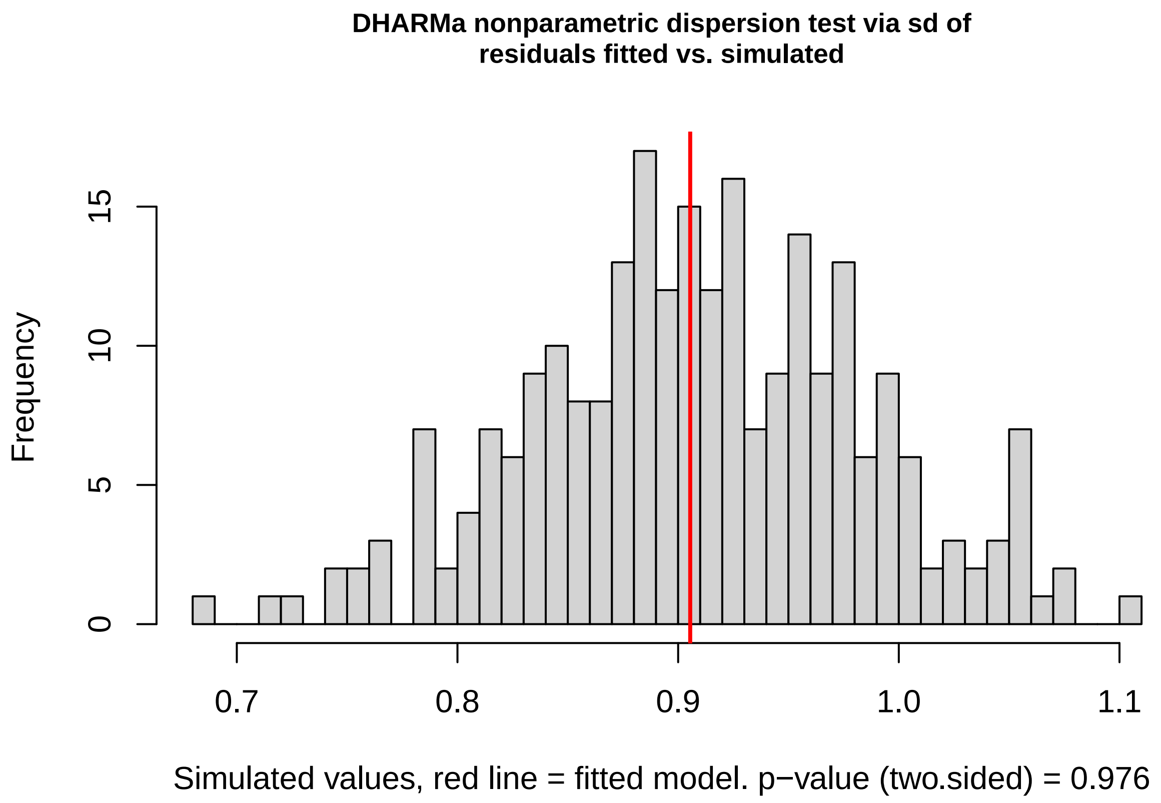


```
##
##  DHARMa nonparametric dispersion test via sd of residuals fitted vs.
##  simulated
##
## data:  simulationOutput
## dispersion = 0.99578, p-value = 0.976
## alternative hypothesis: two.sided
```

```
testZeroInflation(sspt5)
```

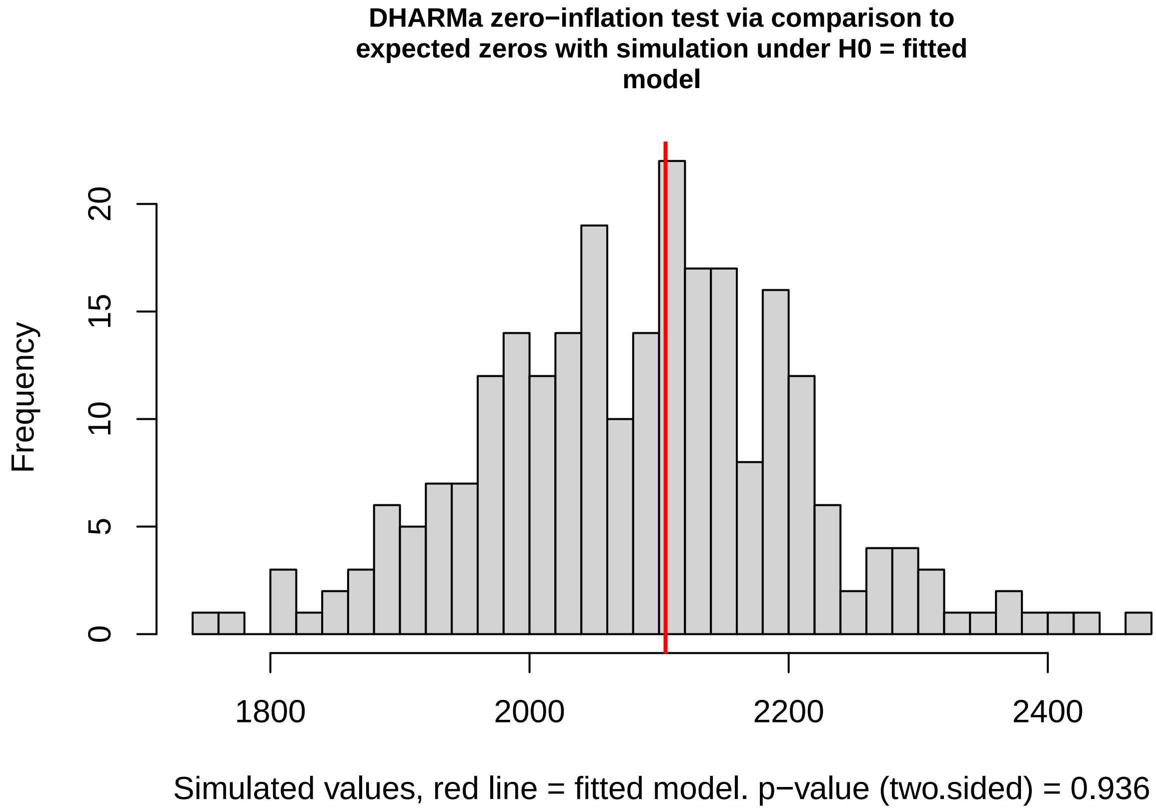


```
##
##  DHARMa zero-inflation test via comparison to expected zeros with
##  simulation under H0 = fitted model
##
## data:  simulationOutput
## ratioObsSim = 1.0091, p-value = 0.936
## alternative hypothesis: two.sided
```

5 Final comments on observed effects and power

5.1 Effects on orthographic learning

The findings from analyzing orthographic choice performance did not reveal any reliable, statistically significant and substantial influence of grade, lexicality, or length on learning rate (early, late, or overall). In contrast, the findings from analyzing spelling performance revealed effects of both lexicality and length on overall learning rate. For lexicality, effects on individual phases (early and late) were not large enough to attain statistical significance. For length, it seems that the effect originates primarily in late learning.

Turning to the effect estimates themselves, without considering significance, we see that mean orthographic choice performance was about 0.62 (model Intercept about 0.5). The effect of grade was about 0.5 (i.e., taking the intercept into account, from 0.56 for G2 to 0.68 for G5). The effect of lexicality was about 1 (i.e., from 0.5 for pseudowords to 0.73 for words). The effect of length was about −0.3 (i.e., from 0.59 for bisyllabic words to 0.66 for monosyllables). The early learning effect (i.e., after 2 exposures) was about 0.25; the late learning effect (between 2 and 6 exposures) was similar. The estimated effect of lexicality on learning was between −0.4 (early) and +0.4 (late). These effects are numerically substantial but largely cancel out when we consider the overall learning, because it seems that words have an early advantage whereas pseudowords

have a late advantage, such that over the course of six exposures there is little overall difference in learning by lexicality. The estimated effect of grade on learning was uniformly lower, around 0.12. The estimated effect of length on learning was between −0.15 (early) and +0.4 (late), partially cancelling out over the six exposures, with short words possibly having a slight early advantage and long words a larger late advantage.

A similar analysis of the spelling task reveals mean performance about 0.25 (model Intercept about −1.1). The effect of grade was about 0.7 (i.e., taking the intercept into account, from 0.19 for G2 to 0.32 for G5). The effect of lexicality was about 1.5 (i.e., from 0.14 for pseudowords to 0.41 for words). The effect of length was negligible, about 0.05. The early learning effect (i.e., after 2 exposures) was about 0.25; the late learning effect (between 2 and 6 exposures) was about 0.37, nearly one standard error higher. The estimated effect of lexicality on learning was about 0.23 for both early and late learning. The estimated effect of grade on learning was uneven, around 0.2 for early learning but −0.3 for late learning, consistent with an early advantage for Grade 5 and a late advantage for Grade 2. The estimated effect of length on learning was also uneven, negligible (around −0.04) for early learning but around .57 for late learning, consistent with a late advantage for longer words.

Considering both tasks, some of these effects are quite substantial, but given the necessarily greater standard error for interactions than for simple effects, a much larger set of items and much larger sample will be needed to confidently detect them. Thus future studies can focus on specific effects and rely on targeted item sets designed to reveal learning differences, for example, depending on word knowledge alone (i.e., lexicality); or, alternatively, item length alone.

5.2 Statistical power

There are many factors considered simultaneously in this exploratory study, and not enough items to be able to draw conclusions about their combined effects. Power for higher-order interactions is simply too low; unfortunately it was not feasible to lengthen the procedure by adding more trials while working with these ages and within the school setting. Thus the conclusions are tentative and primarily indicative; still, they are informative to the extent clear directions for follow-up research can be reliably identified.

Focusing on simple interactions with exposure allows us to detect effects on learning rate that can be elaborated in future research. Working through model comparisons to settle on random structures that are sufficiently supported by the data, we found that standard errors for the estimates do not become unduly small by removing negligible random slopes and correlation. In fact, the following table shows that the standard errors of the two-way interactions are very stable across models with full vs. reduced random structure and with or without length effects included. (The effect estimates themselves are also fairly stable across models.) This suggests that our power to detect these effects is not substantially affected by exactly how the model is set up or how many factors it contains as long as the random structure is as rich as supported by the observed variability in the data.

```
mdlist <- c("ol3c1","ol3c3","oln2","oln4", "ol3ct","ol3ct3","oln5",
            "sp3c1","sp3c3","spn2","spn4", "sp3ct","sp3ct3","spn5"  )
nmlist <- c("OC26:max","OC26:min","OC26/len:max","OC26/len:min",
            "OCov:max","OCov:min","OCov/len:min",
            "SP26:max","SP26:min","SP26/len:max","SP26/len:min",
            "SPov:max","SPov:min","SPov/len:min")
nm <- length(mdlist)
fdl <- data.frame()
for (imdl in 1:nm) {
  mdm <- mdlist[imdl][[1]]
  mdl <- eval(as.name(mdm) )
  mdn <- nmlist[imdl]
  if (imdl %in% c(1:4,8:11)) { leff <- c("reps2_0","reps6_2") } else { leff <- c("repsC") }
  for (eff in c("lex2C","gradeC")) {
    for (eeff in leff) {
      fdl <- rbind(fdl,
```

```
                    cbind(
                      data.frame(Task=substr(mdn,1,2),
                                 Model=mdm,
                                 Mod.Typ=substr(mdn,3,nchar(mdn)),
                                 Lex.eff=eff,
                                 Reps.eff=eeff),
                      round(t(coef(summary(mdl))[
                        paste(eeff,eff,sep=":"),c("Estimate","Std. Error")
                        ]),3)))
    }
  }
}
print(fdl[order(fdl$Task,fdl$Lex.eff,fdl$Reps.eff),])
```

```
##    Task  Model    Mod.Typ Lex.eff Reps.eff Estimate Std. Error
## 3    OC  ol3c1     26:max  gradeC  reps2_0    0.060      0.228
## 7    OC  ol3c3     26:min  gradeC  reps2_0    0.115      0.178
## 11   OC   oln2 26/len:max  gradeC  reps2_0    0.157      0.223
## 15   OC   oln4 26/len:min  gradeC  reps2_0    0.106      0.180
## 4    OC  ol3c1     26:max  gradeC  reps6_2    0.145      0.277
## 8    OC  ol3c3     26:min  gradeC  reps6_2    0.121      0.221
## 12   OC   oln2 26/len:max  gradeC  reps6_2    0.124      0.285
## 16   OC   oln4 26/len:min  gradeC  reps6_2    0.128      0.223
## 18   OC  ol3ct     ov:max  gradeC    repsC    0.195      0.243
## 20   OC ol3ct3     ov:min  gradeC    repsC    0.201      0.214
## 22   OC   oln5 ov/len:min  gradeC    repsC    0.198      0.211
## 1    OC  ol3c1     26:max   lex2C  reps2_0   -0.341      0.217
## 5    OC  ol3c3     26:min   lex2C  reps2_0   -0.337      0.196
## 9    OC   oln2 26/len:max   lex2C  reps2_0   -0.442      0.204
## 13   OC   oln4 26/len:min   lex2C  reps2_0   -0.368      0.198
## 2    OC  ol3c1     26:max   lex2C  reps6_2    0.463      0.285
## 6    OC  ol3c3     26:min   lex2C  reps6_2    0.389      0.250
## 10   OC   oln2 26/len:max   lex2C  reps6_2    0.464      0.265
## 14   OC   oln4 26/len:min   lex2C  reps6_2    0.429      0.244
## 17   OC  ol3ct     ov:max   lex2C    repsC    0.119      0.269
## 19   OC ol3ct3     ov:min   lex2C    repsC    0.072      0.247
## 21   OC   oln5 ov/len:min   lex2C    repsC    0.080      0.244
## 25   SP  sp3c1     26:max  gradeC  reps2_0    0.299      0.276
## 29   SP  sp3c3     26:min  gradeC  reps2_0    0.181      0.214
## 33   SP   spn2 26/len:max  gradeC  reps2_0    0.208      0.255
## 37   SP   spn4 26/len:min  gradeC  reps2_0    0.178      0.215
## 26   SP  sp3c1     26:max  gradeC  reps6_2   -0.401      0.296
## 30   SP  sp3c3     26:min  gradeC  reps6_2   -0.341      0.230
## 34   SP   spn2 26/len:max  gradeC  reps6_2   -0.304      0.291
## 38   SP   spn4 26/len:min  gradeC  reps6_2   -0.346      0.234
## 40   SP  sp3ct     ov:max  gradeC    repsC   -0.072      0.281
## 42   SP sp3ct3     ov:min  gradeC    repsC   -0.155      0.210
## 44   SP   spn5 ov/len:min  gradeC    repsC   -0.171      0.213
## 23   SP  sp3c1     26:max   lex2C  reps2_0    0.344      0.271
## 27   SP  sp3c3     26:min   lex2C  reps2_0    0.201      0.206
## 31   SP   spn2 26/len:max   lex2C  reps2_0    0.228      0.241
## 35   SP   spn4 26/len:min   lex2C  reps2_0    0.209      0.208
## 24   SP  sp3c1     26:max   lex2C  reps6_2    0.339      0.272
## 28   SP  sp3c3     26:min   lex2C  reps6_2    0.250      0.226
```

```
## 32   SP   spn2 26/len:max   lex2C  reps6_2    0.239      0.253
## 36   SP   spn4 26/len:min   lex2C  reps6_2    0.240      0.229
## 39   SP  sp3ct      ov:max   lex2C    repsC    0.661      0.249
## 41   SP sp3ct3      ov:min   lex2C    repsC    0.467      0.201
## 43   SP   spn5 ov/len:min   lex2C    repsC    0.468      0.204
```

In this table, OC=orthographic choice, SP=spelling; 26 = models with a three-level exposure variable (successive differences reps2_0 and reps6_2), ov = models with an overall learning effect (repsC); "len" refers to models with length effects included; "max" refers to the model with the richer/full random structure and "min" to the models with reduced random structure (nonsingular convergence); Lex.eff and Reps.eff are the components of the two-way interaction for which an effect and standard error are shown. All these are just individually picked values from the model summaries already shown above.

In comparison, here is the absolute simplest model for orthographic choice, with only two-way interactions (and no length effects) included in the fixed-effects part, and the justified random slopes in the random-effects part. The effect estimates and their standard errors are about the same as those in the richer models listed above, consistent with no beneficial effect on power of fitting impoverished models to the same data.

```
olf99 <- as.formula( "orthaccu ~ 1 + reps*(lex2C+gradeC) + (reps6_2||sID) + (reps6_2+reps2_0+gradeC||ta
ol99 <- glmer( olf99, som, family=binomial, control=glmerControl(optCtrl=list(maxfun=1e5), optimizer =
print(summary(ol99),corr=F)
```

```
## Generalized linear mixed model fit by maximum likelihood (Laplace
##   Approximation) [glmerMod]
##  Family: binomial  ( logit )
## Formula:
## orthaccu ~ 1 + reps * (lex2C + gradeC) + (reps6_2 || sID) + (reps6_2 +
##     reps2_0 + gradeC || targetS)
##    Data: som
## Control: glmerControl(optCtrl = list(maxfun = 1e+05), optimizer = "nloptwrap")
##
##      AIC      BIC   logLik deviance df.resid
##   4956.8   5051.4  -2463.4   4926.8     4048
##
## Scaled residuals:
##     Min      1Q  Median      3Q     Max
## -5.2073 -0.8214  0.3013  0.7747  2.1751
##
## Random effects:
##  Groups    Name        Variance Std.Dev.
##  sID       (Intercept) 0.03333  0.1826
##  sID.1     reps6_2     0.17757  0.4214
##  targetS   (Intercept) 0.69037  0.8309
##  targetS.1 reps6_2     0.14947  0.3866
##  targetS.2 reps2_0     0.05291  0.2300
##  targetS.3 gradeC      0.86270  0.9288
## Number of obs: 4063, groups:  sID, 127; targetS, 32
##
## Fixed effects:
##                Estimate Std. Error z value Pr(>|z|)
## (Intercept)     0.48389    0.15360   3.150 0.001631 **
## reps2_0         0.22053    0.09783   2.254 0.024183 *
## reps6_2         0.26507    0.13047   2.032 0.042195 *
## lex2C          -1.01504    0.30531  -3.325 0.000885 ***
## gradeC          0.47949    0.18619   2.575 0.010016 *
## reps2_0:lex2C  -0.34606    0.19277  -1.795 0.072625 .
```

```
## reps6_2:lex2C   0.38628    0.24559   1.573 0.115750
## reps2_0:gradeC  0.11752    0.17636   0.666 0.505188
## reps6_2:gradeC  0.12297    0.21912   0.561 0.574672
## ---
## Signif. codes:  0 '***' 0.001 '**' 0.01 '*' 0.05 '.' 0.1 ' ' 1
```

6 Session information

```
sessionInfo()
```

```
## R version 4.1.0 (2021-05-18)
## Platform: x86_64-w64-mingw32/x64 (64-bit)
## Running under: Windows 10 x64 (build 19042)
##
## Matrix products: default
##
## locale:
## [1] LC_COLLATE=English_United States.1252
## [2] LC_CTYPE=English_United States.1252
## [3] LC_MONETARY=English_United States.1252
## [4] LC_NUMERIC=C
## [5] LC_TIME=English_United States.1252
##
## attached base packages:
## [1] stats     graphics  grDevices utils     datasets  methods   base
##
## other attached packages:
##  [1] car_3.0-10     gmodels_2.18.1 nlme_3.1-152   ggplot2_3.3.3  DHARMa_0.4.3
##  [6] effects_4.2-0  carData_3.0-4  MASS_7.3-54    lme4_1.1-27    Matrix_1.3-3
##
## loaded via a namespace (and not attached):
##  [1] Rcpp_1.0.6        lattice_0.20-44   gtools_3.8.2      assertthat_0.2.1
##  [5] digest_0.6.27     foreach_1.5.1     utf8_1.2.1        R6_2.5.0
##  [9] cellranger_1.1.0  survey_4.0        evaluate_0.14     pillar_1.6.1
## [13] rlang_0.4.11      curl_4.3.1        readxl_1.3.1      minqa_1.2.4
## [17] data.table_1.14.0 gdata_2.18.0      nloptr_1.2.2.2    rmarkdown_2.8
## [21] splines_4.1.0     stringr_1.4.0     foreign_0.8-81    munsell_0.5.0
## [25] compiler_4.1.0    xfun_0.23         pkgconfig_2.0.3   htmltools_0.5.1.1
## [29] mitools_2.4       nnet_7.3-16       insight_0.14.1    tidyselect_1.1.1
## [33] tibble_3.1.2      rio_0.5.26        codetools_0.2-18  fansi_0.5.0
## [37] crayon_1.4.1      dplyr_1.0.6       withr_2.4.2       grid_4.1.0
## [41] gtable_0.3.0      lifecycle_1.0.0   DBI_1.1.1         magrittr_2.0.1
## [45] scales_1.1.1      zip_2.2.0         stringi_1.6.1     ellipsis_0.3.2
## [49] generics_0.1.0    vctrs_0.3.8       boot_1.3-28       openxlsx_4.2.3
## [53] iterators_1.0.13  tools_4.1.0       forcats_0.5.1     glue_1.4.2
## [57] purrr_0.3.4       hms_1.1.0         abind_1.4-5       survival_3.2-11
## [61] yaml_2.2.1        colorspace_2.0-1  knitr_1.33        haven_2.4.1
```
