# Supplementary material for: Lexicality effects on orthographic learning in beginning and advanced readers of Dutch: An eye-tracking study
Source: Q J Exp Psychol (Hove). 2021 Sep 28;75(6):1135–54. doi: 10.1177/17470218211047420 (PMC9016678; doi:10.1177/17470218211047420)
Supplement: sj-pdf-3-qjp-10.1177_17470218211047420 – Supplemental material for Lexicality effects on orthographic learning in beginning and advanced readers of Dutch: An eye-tracking study [file sj-pdf-3-qjp-10.1177_17470218211047420.pdf]

# Orthographic learning: Online measures analysis (eye tracking)

Athanassios Protopapas

25 August 2021

## Contents

**1 Introduction** **1**

**2 Analysis of gaze duration (first pass)** **2**
2.1 Graphical display of data trends . . . 2
2.2 General modeling considerations . . . 3
2.3 Full model . . . 4
2.4 Excluding length from the smooth interaction . . . 5
2.5 Model diagnostics and effects . . . 7
2.6 Plot of summed effects . . . 8
2.7 Excluding length entirely (model reported in published article) . . . 10
2.7.1 Model diagnostics and effects . . . 11
2.7.2 Plot of summed effects . . . 13

**3 Sequential effects** **14**
3.1 Model diagnostics and effects . . . 16

**4 Analysis of dwell time (total)** **18**
4.1 Graphical display of data trends . . . 18
4.2 Full model . . . 19
4.3 Excluding length . . . 20
4.4 Model diagnostics and effects . . . 22
4.5 Plot of summed effects . . . 24

**5 Session information** **25**

# 1 Introduction

In this document we report analyses for online outcome measures (eye tracking variables, namely gaze duration and dwell time) in the orthographic learning study of van Viersen et al. (manuscript submitted to *Quarterly Journal of Experimental Psychology*)

```
library(mgcv)
library(parallel)
library(itsadug)
library(car)
```

```
load("rdat.Rda")
str(rdat)
```

```
## 'data.frame':    6644 obs. of  18 variables:
##  $ sID        : int  40010118 40010118 40010118 40010118 40010118 40010118 40010118 40010118 4001011
```

```
##  $ sentID     : Factor w/ 64 levels "s1001","s1002",..: 52 51 34 44 5 22 18 50 46 8 ...
##  $ targID     : Factor w/ 32 levels "p1109","p1110",..: 25 10 25 25 1 1 17 9 13 1 ...
##  $ TRIAL      : int  65 72 82 69 41 6 54 55 19 68 ...
##  $ grade      : Factor w/ 2 levels "G2","G5": 1 1 1 1 1 1 1 1 1 1 ...
##  $ targreps   : int  6 2 6 6 6 6 6 6 6 6 ...
##  $ lex        : Factor w/ 2 levels "p","w": 2 1 2 2 1 1 2 1 1 1 ...
##  $ Nsyl       : int  1 1 1 1 1 1 1 1 2 1 ...
##  $ gradeC     : num  -0.5 -0.5 -0.5 -0.5 -0.5 -0.5 -0.5 -0.5 -0.5 -0.5 ...
##  $ lexC       : num  -0.5 0.5 -0.5 -0.5 0.5 0.5 -0.5 0.5 0.5 0.5 ...
##  $ NsylC      : num  -0.5 -0.5 -0.5 -0.5 -0.5 -0.5 -0.5 -0.5 0.5 -0.5 ...
##  $ repN       : int  4 2 6 5 3 1 5 5 2 5 ...
##  $ gradelex   : Factor w/ 4 levels "G2p","G2w","G5p",..: 2 1 2 2 1 1 2 1 1 1 ...
##  $ gradesyl   : Factor w/ 4 levels "G21","G22","G51",..: 1 1 1 1 1 1 1 1 2 1 ...
##  $ lexsyl     : Factor w/ 4 levels "p1","p2","w1",..: 3 1 3 3 1 1 3 1 2 1 ...
##  $ gradelexsyl: Factor w/ 8 levels "G2p1","G2p2",..: 3 1 3 3 1 1 3 1 2 1 ...
##  $ fDwelTl    : num  6.83 6.73 4.98 6.83 6.91 ...
##  $ DwelTl     : num  6.83 6.73 7.63 6.83 7.14 ...
```

```
nc <- detectCores()
cl <- makeCluster(nc)
col1 <- "#4040FF"
col2 <- "#60A000"
```

# 2 Analysis of gaze duration (first pass)

The main variable of interest is exposure: Participants were repeatedly exposed to a set of items (words and pseudowords) in the context of some template sentences. Some items were encountered twice and others six times. We are interested in the evolution of looking behavior (here, gaze duration) as a function of exposure, reflecting the effect of recent experience with the same item, cumulatively referred to as “learning curve” (or “learning effect”). In addition, we are interested in the potential modulation of this learning curve by item factors (lexicality, length) and participant factors (grade).

## 2.1 Graphical display of data trends

The following graph displays mean gaze duration for each repetition, split by grade and lexicality. These are standard box plots displaying the median, 1st and 3rd quartiles, and min/max contingent on distance from the central half. Note that there are twice as many data points going into the first couple of repetitions compared to the following 4, as half of the items were only presented twice whereas the other half were presented six times. Overall the data are fairly noisy and learning trends are difficult to discern.

```
meangd <- aggregate(fDwelTl~sID+grade+lex+repN,rdat,mean)
par(mfrow=c(2,2))
par(oma=c(2.5,2,0,0))
par(mar=c(0.1,2,1.95,0.1))
par(mgp=c(1.25,0.25,0))
par(tcl= -0.25)
boxplot( exp(fDwelTl)~repN, data=meangd, xaxt="n", main="Grade 2, words",
         ylim=c(0,1750), xlim=c(0.5,6.5), xlab="", ylab="",
         subset= grade=="G2" & lex=="w",
         boxwex=0.3, at=1:6,
         col="lightblue")
axis(1,at=1:6,labels=rep("",6))
boxplot( exp(fDwelTl)~repN, data=meangd, xaxt="n", main="Grade 2, pseudowords",
         ylim=c(0,1750), xlim=c(0.5,6.5), xlab="", ylab="",
```

```
         subset= grade=="G2" & lex=="p",
         boxwex=0.3, at=1:6,
         col="lightblue")
axis(1,at=1:6,labels=rep("",6))
boxplot( exp(fDwelTl)~repN, data=meangd, xaxt="n", main="Grade 5, words", xpd=NA,
         ylim=c(0,1750), xlim=c(0.5,6.5), xlab="", ylab="",
         subset= grade=="G5" & lex=="w",
         boxwex=0.3, at=1:6,
         col="lightblue")
axis(1,at=1:6)
boxplot( exp(fDwelTl)~repN, data=meangd, xaxt="n", main="Grade 5, pseudowords", xpd=NA,
         ylim=c(0,1750), xlim=c(0.5,6.5), xlab="", ylab="",
         subset= grade=="G5" & lex=="p",
         boxwex=0.3, at=1:6,
         col="lightblue")
axis(1,at=1:6)
mtext("Repetition/Exposure",1,outer=T,line=1.5)
mtext("Gaze duration on target (ms)",2,outer=T)
```

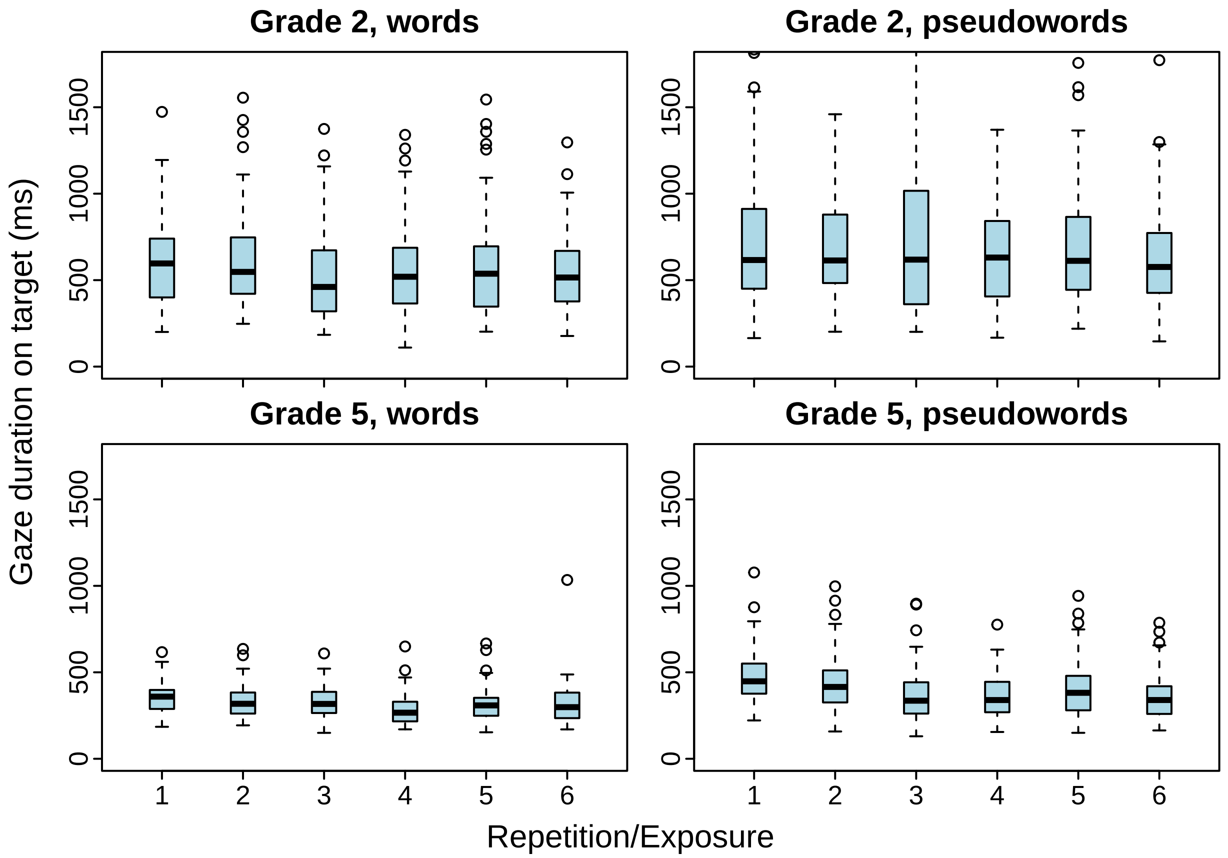


## 2.2 General modeling considerations

The learning curve is modeled as a "smooth term" in generalized additive mixed-effects models (GAMMs), which are a special form of regression analysis. The curve is "penalized" to remain smooth and thereby avoid overfitting. In general, the dependent variable is modeled as a sum of fixed effects (in the usual sense of standard linear regression) plus some random effects (intercepts—and possibly slopes—for sampled factors), plus some smooth terms for the effect of exposure. The fixed effects correspond to the average effects

(across exposures) of grade, lexicality, and length, and their interactions. The random effects correspond to individual deviations from the average for certain grouping factors (here, participants, sentences, and target items, i.e., words). The smooth terms correspond to curves modeling the continuous effect of exposure. A significant fixed effect, as usual, means the effect estimate is statistically distinguishable from zero. A significant smooth term, however, means the curve is not a flat line. Thus, significant smooth terms correspond to different shapes of curves. Interactions between factors and continuous effects are modeled as additional curves, one for each level of the factor. For example, if a model with two grade-specific smooth terms, in addition to the overall learning curve, is significantly better (in model comparison) than a model without these additional smooth terms, this means that grade affects the shape of the learning curve, in other words there is a grade × exposure interaction. Additional smooth terms can be defined for more levels or for combinations of levels across factors (effectively higher-order interactions).

## 2.3 Full model

We first fit a model in which the smooth for number of exposures (`repN`) is complemented by 8 smooths accounting for the 3-way interaction of grade×lexicality×length. These correspond to the 8 levels of factor `gradelexsyl`, which was made by concatenating the factors for grade, lexicaly, and length (2 each). The fixed effects are in fact difference-coded: `gradeC` is −0.5 for Grade 2 and +0.5 for Grade 5; `lexC` is −0.5 for words and +0.5 for pseudowords; and `NsylC` is −0.5 for monosyllables and +0.5 for bisyllables. The random factors are participants (`sID`), sentence templates (`sentID`), and target items (`targID`). We add random slopes for length interacting with lexicality for participants.

We include all available data points for all items. Because half of the items were encountered twice and half were encountered six times, this means there are twice as many data points for the first and second exposure as there are for the subsequent four exposures.

```
fbm0d <- bam( fDwelTl ~ 1 + gradeC + lexC + NsylC + gradeC*lexC*NsylC +
                s(repN,bs="tp",k=6) +
                s(repN,by=gradelexsyl,bs="tp",k=6,m=1) +
                s(sID,bs="re") + s(sentID,bs="re") + s(targID,bs="re") +
                s(Nsyl,lex,sID,bs="re",m=1),
              cluster=cl,
              data=rdat
)
summary(fbm0d)
```

```
##
## Family: gaussian
## Link function: identity
##
## Formula:
## fDwelTl ~ 1 + gradeC + lexC + NsylC + gradeC * lexC * NsylC +
##     s(repN, bs = "tp", k = 6) + s(repN, by = gradelexsyl, bs = "tp",
##     k = 6, m = 1) + s(sID, bs = "re") + s(sentID, bs = "re") +
##     s(targID, bs = "re") + s(Nsyl, lex, sID, bs = "re", m = 1)
##
## Parametric coefficients:
##                   Estimate Std. Error t value Pr(>|t|)
## (Intercept)       16.47277    2.26295   7.279 3.75e-13 ***
## gradeC             5.10253    1.22096   4.179 2.97e-05 ***
## lexC               1.87949    2.44603   0.768    0.442
## NsylC              0.41977    0.94832   0.443    0.658
## gradeC:lexC        0.95110    1.31997   0.721    0.471
## gradeC:NsylC      -0.04368    0.51249  -0.085    0.932
## lexC:NsylC         1.22639    1.63239   0.751    0.453
```

```
## gradeC:lexC:NsylC  0.84798    0.88250   0.961    0.337
## ---
## Signif. codes:  0 '***' 0.001 '**' 0.01 '*' 0.05 '.' 0.1 ' ' 1
##
## Approximate significance of smooth terms:
##                                 edf Ref.df      F  p-value
## s(repN)                   2.713e+00  3.271 14.376  < 2e-16 ***
## s(repN):gradelexsylG2p1 2.389e-05  5.000  0.000  0.91420
## s(repN):gradelexsylG2p2 2.112e-05  5.000  0.000  0.95625
## s(repN):gradelexsylG2w1 1.784e+00  5.000  0.932  0.03635 *
## s(repN):gradelexsylG2w2 8.413e-05  5.000  0.000  0.43564
## s(repN):gradelexsylG5p1 8.595e-05  5.000  0.000  0.44057
## s(repN):gradelexsylG5p2 2.411e+00  5.000  1.668  0.00773 **
## s(repN):gradelexsylG5w1 2.335e-05  5.000  0.000  0.88719
## s(repN):gradelexsylG5w2 3.317e-05  5.000  0.000  0.67170
## s(sID)                    9.362e-01  1.000 19.975 7.61e-05 ***
## s(sentID)                 3.638e+01 63.000  1.389  < 2e-16 ***
## s(targID)                 2.132e+01 28.000  3.270  < 2e-16 ***
## s(Nsyl,lex,sID)           3.628e-01  2.000  0.260  0.24713
## ---
## Signif. codes:  0 '***' 0.001 '**' 0.01 '*' 0.05 '.' 0.1 ' ' 1
##
## R-sq.(adj) =  0.169   Deviance explained = 17.9%
## fREML = 7250.8  Scale est. = 0.52515   n = 6541
```

In this model, we have significant fixed-effect terms including the three-way interaction, a significant smooth term for the main learning effect, plus two more significant smooth terms, consistent with interactions. However, before interpreting we need to check if the 3-way interaction is really necessary.

## 2.4 Excluding length from the smooth interaction

We subsequently fit a reduced model in which the overall smooth for number of exposures (i.e., overall learning effect, `s(repN)`) is complemented by four smooths for the 2-way interaction between grade and lexicality, that is, excluding an interaction with length. We then compare this to the full model. To do this we form a new 4-level factor `gradelex` by concatenating the levels of grade and lexicality.

```
fbm0g <- bam( fDwelTl ~ 1 + gradeC + lexC + NsylC + gradeC*lexC*NsylC +
                s(repN,bs="tp",k=6) +
                s(repN,by=gradelex,bs="tp",k=6,m=1) + # removed syl
                s(sID,bs="re") + s(sentID,bs="re") + s(targID,bs="re") +
                s(Nsyl,lex,sID,bs="re",m=1),
              cluster=cl,
              data=rdat
)
summary(fbm0g)
```

```
##
## Family: gaussian
## Link function: identity
##
## Formula:
## fDwelTl ~ 1 + gradeC + lexC + NsylC + gradeC * lexC * NsylC +
##     s(repN, bs = "tp", k = 6) + s(repN, by = gradelex, bs = "tp",
##     k = 6, m = 1) + s(sID, bs = "re") + s(sentID, bs = "re") +
##     s(targID, bs = "re") + s(Nsyl, lex, sID, bs = "re", m = 1)
```

```
##
## Parametric coefficients:
##                    Estimate Std. Error t value Pr(>|t|)
## (Intercept)       16.46472    2.26383   7.273 3.93e-13 ***
## gradeC             5.09789    1.22144   4.174 3.04e-05 ***
## lexC               1.90354    2.46046   0.774    0.439
## NsylC              0.42232    0.95573   0.442    0.659
## gradeC:lexC        0.96315    1.32775   0.725    0.468
## gradeC:NsylC      -0.04243    0.51648  -0.082    0.935
## lexC:NsylC         1.24241    1.64201   0.757    0.449
## gradeC:lexC:NsylC  0.85701    0.88768   0.965    0.334
## ---
## Signif. codes:  0 '***' 0.001 '**' 0.01 '*' 0.05 '.' 0.1 ' ' 1
##
## Approximate significance of smooth terms:
##                            edf Ref.df      F  p-value
## s(repN)              2.500e+00  3.032 11.031 6.24e-07 ***
## s(repN):gradelexG2p 1.895e-05  5.000  0.000  0.98952
## s(repN):gradelexG2w 2.341e-05  5.000  0.000  0.89635
## s(repN):gradelexG5p 2.431e+00  5.000  1.785  0.00509 **
## s(repN):gradelexG5w 2.570e-05  5.000  0.000  0.87270
## s(sID)              9.358e-01  1.000 19.916 8.03e-05 ***
## s(sentID)           3.646e+01 63.000  1.398  < 2e-16 ***
## s(targID)           2.134e+01 28.000  3.282  < 2e-16 ***
## s(Nsyl,lex,sID)     3.670e-01  2.000  0.264  0.24613
## ---
## Signif. codes:  0 '***' 0.001 '**' 0.01 '*' 0.05 '.' 0.1 ' ' 1
##
## R-sq.(adj) =  0.169   Deviance explained = 17.8%
## fREML = 7252.2  Scale est. = 0.5256    n = 6541
```

```
compareML(fbm0g,fbm0d)
```

```
## fbm0g: fDwelTl ~ 1 + gradeC + lexC + NsylC + gradeC * lexC * NsylC +
##     s(repN, bs = "tp", k = 6) + s(repN, by = gradelex, bs = "tp",
##     k = 6, m = 1) + s(sID, bs = "re") + s(sentID, bs = "re") +
##     s(targID, bs = "re") + s(Nsyl, lex, sID, bs = "re", m = 1)
##
## fbm0d: fDwelTl ~ 1 + gradeC + lexC + NsylC + gradeC * lexC * NsylC +
##     s(repN, bs = "tp", k = 6) + s(repN, by = gradelexsyl, bs = "tp",
##     k = 6, m = 1) + s(sID, bs = "re") + s(sentID, bs = "re") +
##     s(targID, bs = "re") + s(Nsyl, lex, sID, bs = "re", m = 1)

## Warning in sprintf(" ", h1): one argument not used by format ' '

##
## Chi-square test of fREML scores
## -----
##   Model    Score Edf Difference    Df p.value Sig.
## 1 fbm0g 7252.164  18
## 2 fbm0d 7250.797  22      1.367 4.000   0.603
##
## AIC difference: 2.25, model fbm0d has lower AIC.

## Warning in compareML(fbm0g, fbm0d): Only small difference in fREML...
```

The difference between the two models is not significant, therefore we retain the latter, simpler model. Note

that this still includes fixed effects (and interactions) of grade, lexicalty, and length, as all of these factors affect gaze time – but not all of them significantly affect the rate at which gaze time diminishes with additional exposures: In particular, length does not affect the shape of this learning curve, as we could leave it out of the smooth terms without loss of fit. Only one of the additional smooth terms turned out to be statistically significant, namely the combination of Grade 5 and pseudowords. This means that the other 3 combinations of lexicality and grade do not differ from the overall learning effect modeled by the general term.

## 2.5 Model diagnostics and effects

```
par(mar=c(3,3,2,0.2))
par(mgp=c(1.5,0.4,0))
par(tcl= -0.2)
par(mfrow=c(2,2))
gam.check(fbm0g)
```

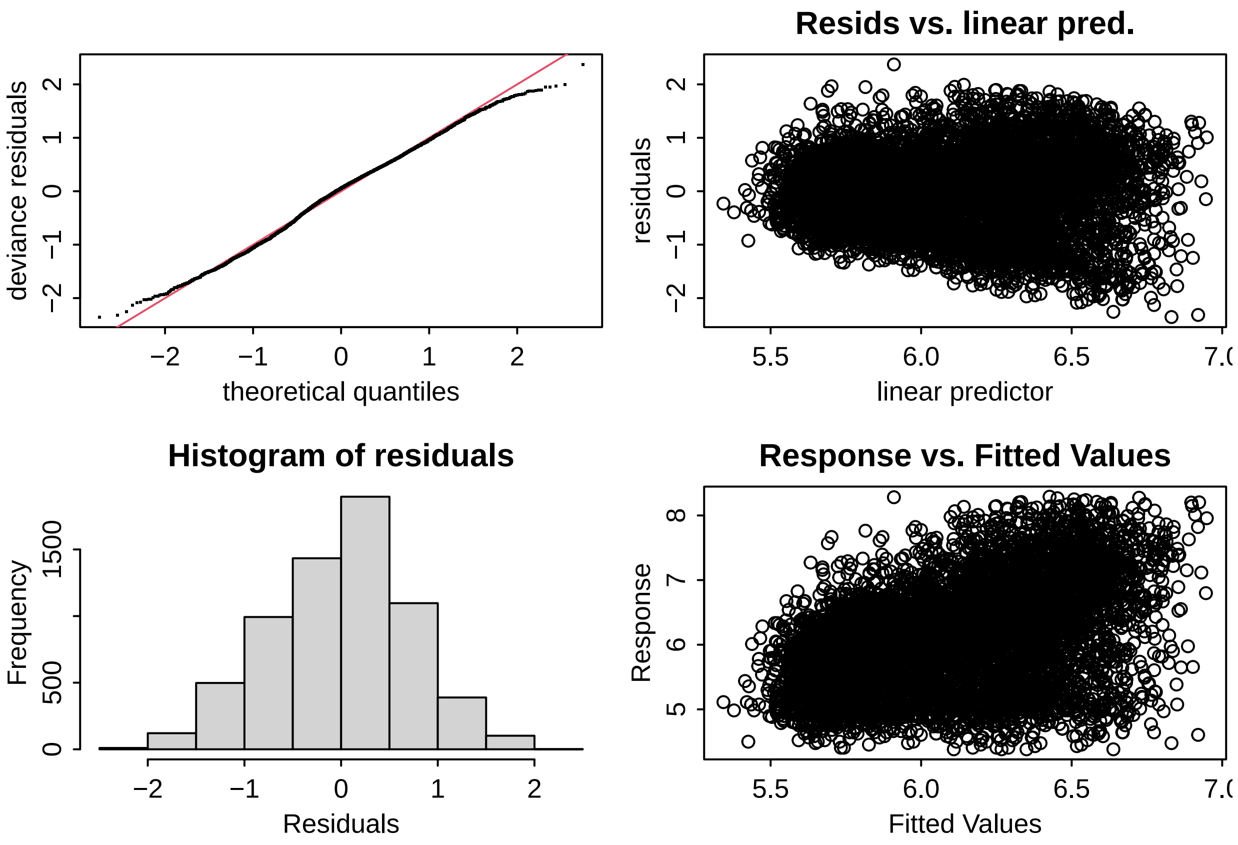


```
##
## Method: fREML   Optimizer: perf newton
## full convergence after 18 iterations.
## Gradient range [-9.688862e-06,7.60139e-08]
## (score 7252.164 & scale 0.5256022).
## Hessian positive definite, eigenvalue range [8.80218e-06,3266.138].
## Model rank =  132 / 132
##
## Basis dimension (k) checking results. Low p-value (k-index<1) may
## indicate that k is too low, especially if edf is close to k'.
##
```

```
##                            k'      edf k-index p-value
## s(repN)              5.00e+00 2.50e+00    1.00    0.42
## s(repN):gradelexG2p 5.00e+00 1.89e-05    1.00    0.46
## s(repN):gradelexG2w 5.00e+00 2.34e-05    1.00    0.44
## s(repN):gradelexG5p 5.00e+00 2.43e+00    1.00    0.41
## s(repN):gradelexG5w 5.00e+00 2.57e-05    1.00    0.46
## s(sID)               1.00e+00 9.36e-01    0.87  <2e-16 ***
## s(sentID)            6.40e+01 3.65e+01      NA      NA
## s(targID)            3.20e+01 2.13e+01      NA      NA
## s(Nsyl,lex,sID)      2.00e+00 3.67e-01      NA      NA
## ---
## Signif. codes:  0 '***' 0.001 '**' 0.01 '*' 0.05 '.' 0.1 ' ' 1
```

```
par(mfrow=c(1,1))
```

```
par(mar=c(3,3,2,0.2))
par(mgp=c(1.5,0.4,0))
par(tcl= -0.2)
plot.gam(fbm0g,page=1,scheme=1)
```

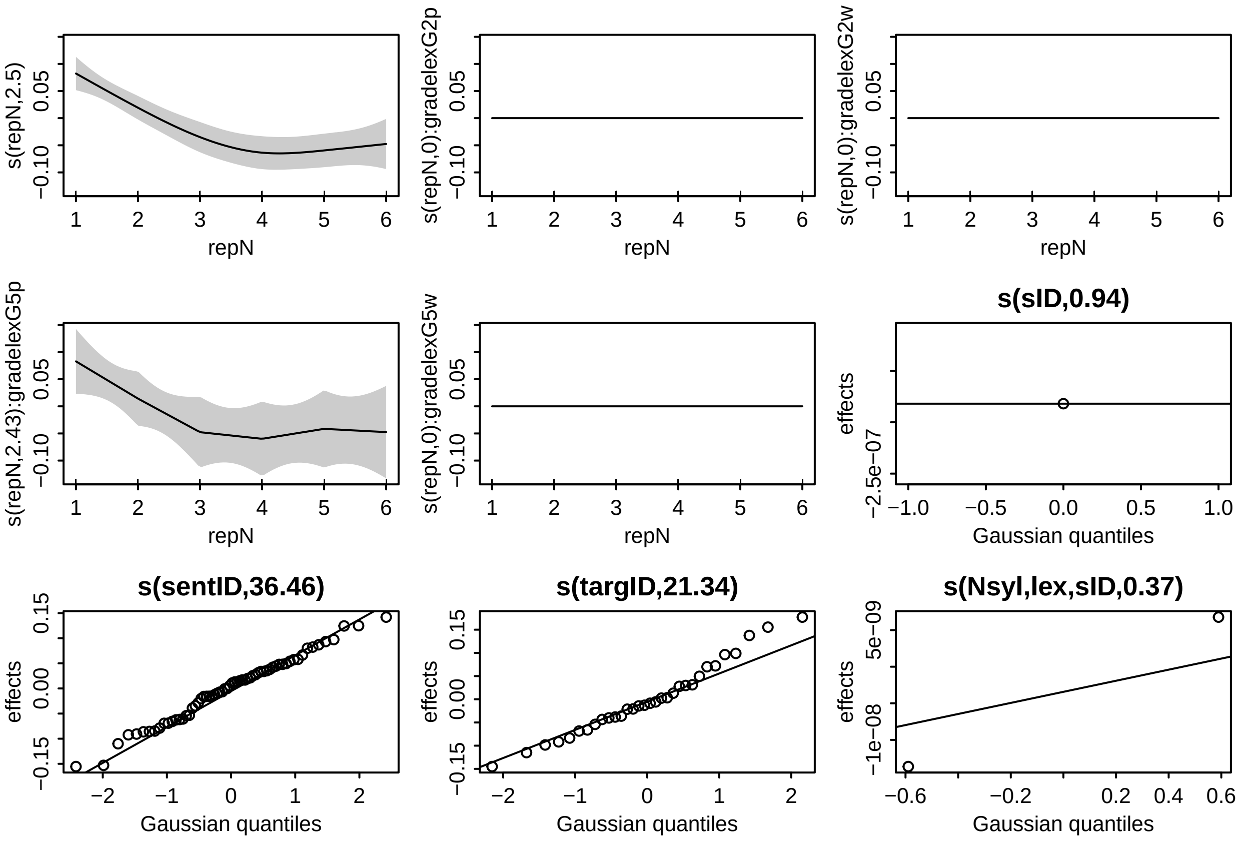


## 2.6 Plot of summed effects

In this plot we can see the summed effects of the fixed factors and smooths. Note the different shape of the curve for Grade 5 pseudowords during the first few of exposures, consistent with faster learning rate in this condition. We do not break down smooths by length because length did not interact significantly with exposure.

```
ylm <- c(0,800)
par(mfcol=c(1,1))
par(oma=c(1.2,1.1,0,0))
par(mar=c(2.0,2.0,1.25,0.01))
par(mgp=c(2.0,0.5,0))
par(tcl= -0.25)
first <- TRUE
for (lx in c("w","p")) {
  for (gd in c("G2","G5")) {
  plot_smooth(fbm0g,view="repN",
             cond=list(gradeC=0.5-(gd=="G2"),lexC=0.5-(lx=="w"),NsylC=0,gradelex=paste(gd,lx,sep="")),
             rm.ranef=c("s(sID)","s(sentID)","s(targID)","s(Nsyl,lex,sID)"),
             se=0,#1.96,
             print.summary=F,ylim=ylm,transform=exp,
             xlab="Repetition/exposure",
             ylab="Gaze duration on target",
             lwd=3,col=ifelse(gd=="G2",col1,col2),lty=ifelse(lx=="w",1,2),cex.lab=1.25,cex.axis=1.1,
             xpd=NA,hide.label=T,yaxs="i",rug=F,
             add= first!=TRUE)
  first <- FALSE
  grid(nx=NA,ny=8,col="gray50")
  }
}
legend(4,230,c("G2 Words","G2 Pseudowords","G5 Words","G5 Pseudowords"),col=rep(c(col1,col2),each=2),lty
```

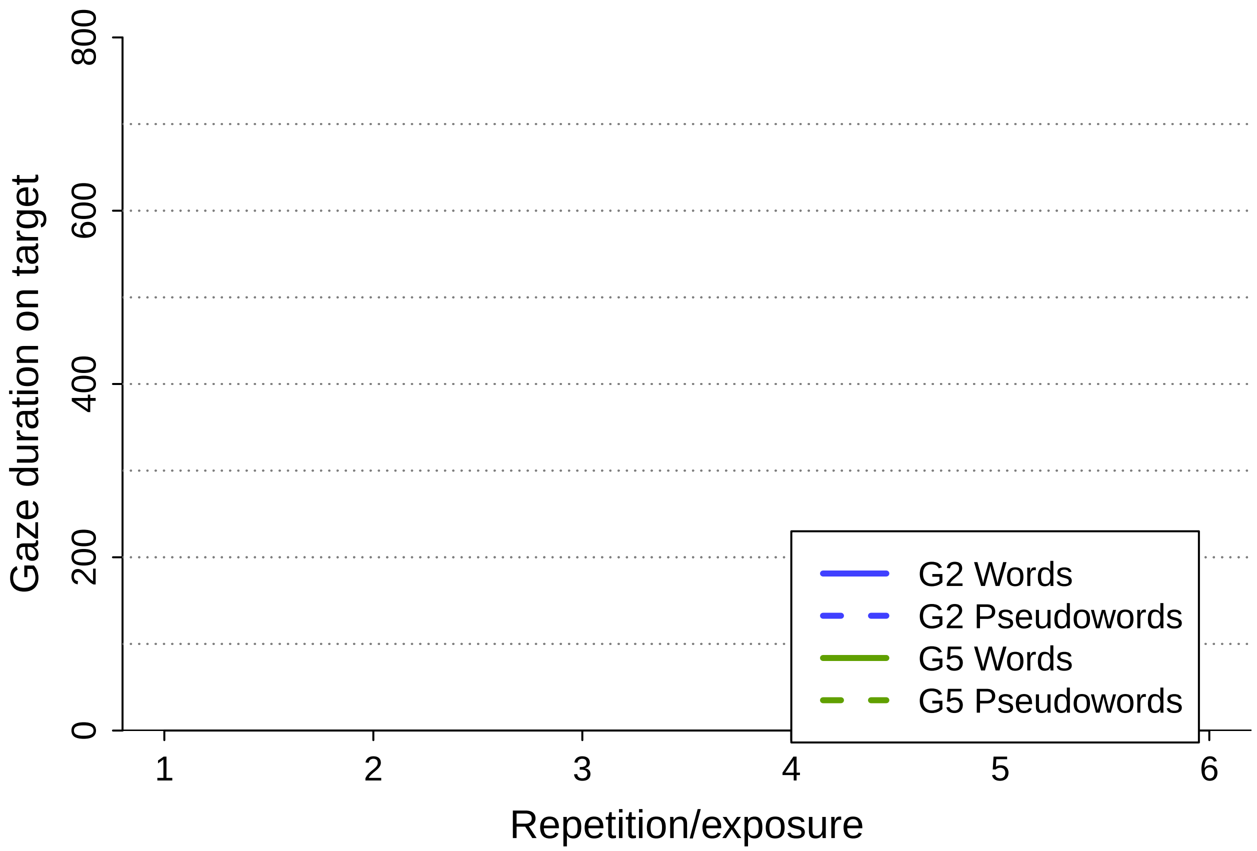

## 2.7 Excluding length entirely (model reported in published article)

We subsequently fit a model leaving out the length factor entirely, for simplicity, given that length does not affect the outcome of primary interest here, namely the rate of learning (i.e., the smooth curve of diminishing gaze duration).

```
fbm0g2 <- bam( fDwelTl ~ 1 + gradeC*lexC +
                s(repN,bs="tp",k=6) +
                s(repN,by=gradelex,bs="tp",k=6,m=1) + # removed syl
                s(sID,bs="re") + s(sentID,bs="re") + s(targID,bs="re") +
                s(lex,sID,bs="re",m=1),
              cluster=cl,
              data=rdat
)
summary(fbm0g2)
```

```
##
## Family: gaussian
## Link function: identity
##
## Formula:
## fDwelTl ~ 1 + gradeC * lexC + s(repN, bs = "tp", k = 6) + s(repN,
##     by = gradelex, bs = "tp", k = 6, m = 1) + s(sID, bs = "re") +
##     s(sentID, bs = "re") + s(targID, bs = "re") + s(lex, sID,
##     bs = "re", m = 1)
##
## Parametric coefficients:
##             Estimate Std. Error t value Pr(>|t|)
## (Intercept)   16.558      2.271   7.291 3.44e-13 ***
## gradeC         5.146      1.225   4.200 2.70e-05 ***
## lexC           4.087      3.478   1.175    0.240
## gradeC:lexC    2.146      1.876   1.143    0.253
## ---
## Signif. codes:  0 '***' 0.001 '**' 0.01 '*' 0.05 '.' 0.1 ' ' 1
##
## Approximate significance of smooth terms:
##                            edf Ref.df      F  p-value
## s(repN)              2.494e+00  3.025 10.901 7.05e-07 ***
## s(repN):gradelexG2p 2.235e-05  5.000  0.000  0.98873
## s(repN):gradelexG2w 2.419e-05  5.000  0.000  0.87524
## s(repN):gradelexG5p 2.424e+00  5.000  1.795  0.00525 **
## s(repN):gradelexG5w 2.629e-05  5.000  0.000  0.87664
## s(sID)              8.982e-01  1.000 18.966  0.00173 **
## s(sentID)           3.634e+01 63.000  1.394  < 2e-16 ***
## s(targID)           2.690e+01 30.000  9.427  < 2e-16 ***
## s(lex,sID)          6.115e-01  2.000  0.694  0.14227
## ---
## Signif. codes:  0 '***' 0.001 '**' 0.01 '*' 0.05 '.' 0.1 ' ' 1
##
## R-sq.(adj) =  0.165   Deviance explained = 17.5%
## fREML = 7271.6  Scale est. = 0.52767   n = 6541
```

```
compareML(fbm0g,fbm0g2)
```

```
## fbm0g: fDwelTl ~ 1 + gradeC + lexC + NsylC + gradeC * lexC * NsylC +
##     s(repN, bs = "tp", k = 6) + s(repN, by = gradelex, bs = "tp",
```

```
##     k = 6, m = 1) + s(sID, bs = "re") + s(sentID, bs = "re") +
##     s(targID, bs = "re") + s(Nsyl, lex, sID, bs = "re", m = 1)
##
## fbm0g2: fDwelTl ~ 1 + gradeC * lexC + s(repN, bs = "tp", k = 6) + s(repN,
##     by = gradelex, bs = "tp", k = 6, m = 1) + s(sID, bs = "re") +
##     s(sentID, bs = "re") + s(targID, bs = "re") + s(lex, sID,
##     bs = "re", m = 1)
```

```
## Warning in sprintf("***", h1): one argument not used by format '***'
```

```
##
## Chi-square test of fREML scores
## -----
##    Model     Score Edf Difference    Df   p.value Sig.
## 1 fbm0g2 7271.637  14
## 2  fbm0g 7252.164  18     19.473 4.000 7.149e-08  ***
##
## AIC difference: -25.85, model fbm0g has lower AIC.
```

The difference between the two models is significant, of course, given the significant fixed effect of length (and significant interactions) in the previous model. However, the difference in $R^2$ and "explained deviance" is less than 2%, and there is no appreciable change in the pattern of results for any other effect (fixed or smooth).

### 2.7.1 Model diagnostics and effects

```r
par(mar=c(3,3,2,0.2))
par(mgp=c(1.5,0.4,0))
par(tcl= -0.2)
par(mfrow=c(2,2))
gam.check(fbm0g2)
```

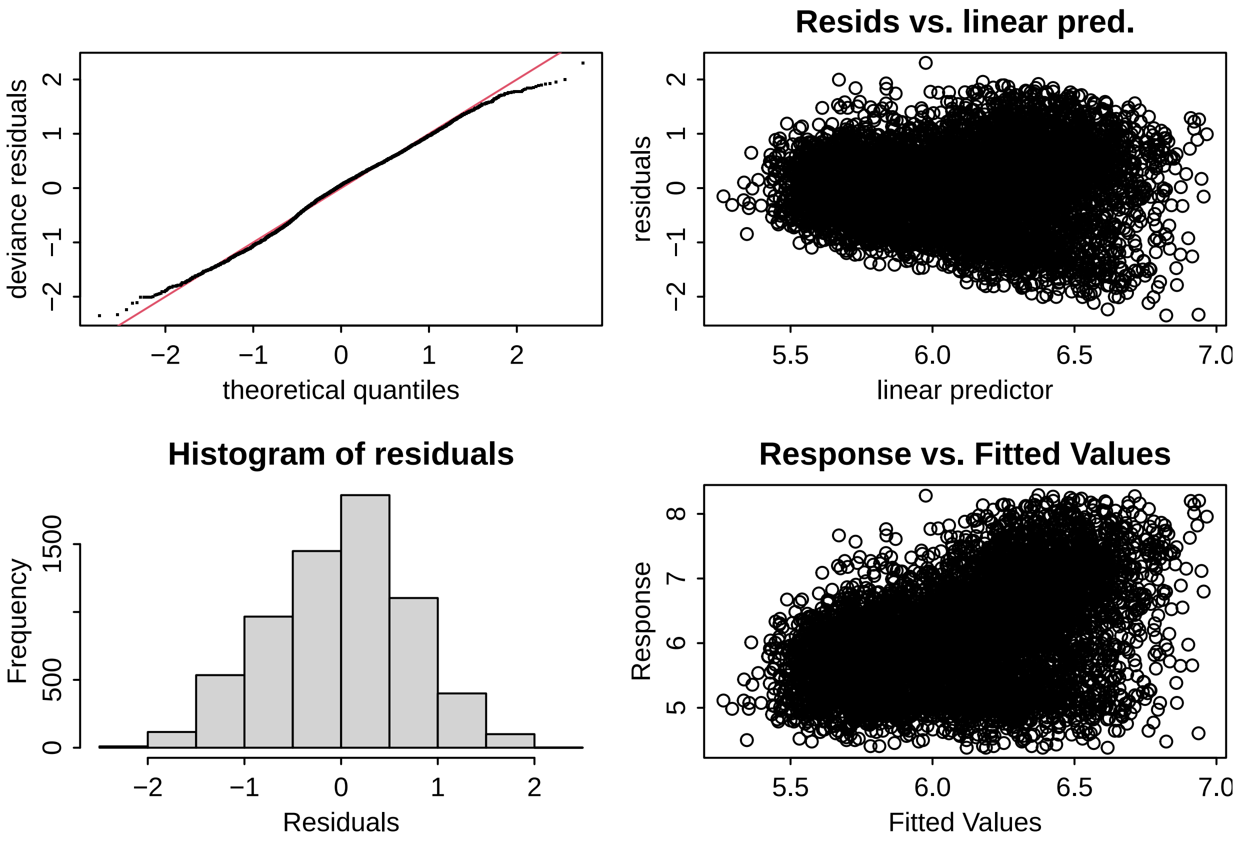


```
##
## Method: fREML   Optimizer: perf newton
## full convergence after 18 iterations.
## Gradient range [-1.035483e-05,8.037805e-09]
## (score 7271.637 & scale 0.5276694).
## Hessian positive definite, eigenvalue range [9.1272e-06,3268.158].
## Model rank =  128 / 128
##
## Basis dimension (k) checking results. Low p-value (k-index<1) may
## indicate that k is too low, especially if edf is close to k'.
##
##                            k'      edf k-index p-value
## s(repN)               5.00e+00 2.49e+00    0.99    0.18
## s(repN):gradelexG2p 5.00e+00 2.24e-05    0.99    0.22
## s(repN):gradelexG2w 5.00e+00 2.42e-05    0.99    0.28
## s(repN):gradelexG5p 5.00e+00 2.42e+00    0.99    0.23
## s(repN):gradelexG5w 5.00e+00 2.63e-05    0.99    0.24
## s(sID)                1.00e+00 8.98e-01    0.90  <2e-16 ***
## s(sentID)             6.40e+01 3.63e+01      NA      NA
## s(targID)             3.20e+01 2.69e+01      NA      NA
## s(lex,sID)            2.00e+00 6.11e-01      NA      NA
## ---
## Signif. codes:  0 '***' 0.001 '**' 0.01 '*' 0.05 '.' 0.1 ' ' 1
```

```
par(mfrow=c(1,1))
```

```
par(mar=c(3,3,2,0.2))
par(mgp=c(1.5,0.4,0))
par(tcl= -0.2)
plot.gam(fbm0g2,page=1,scheme=1)
```

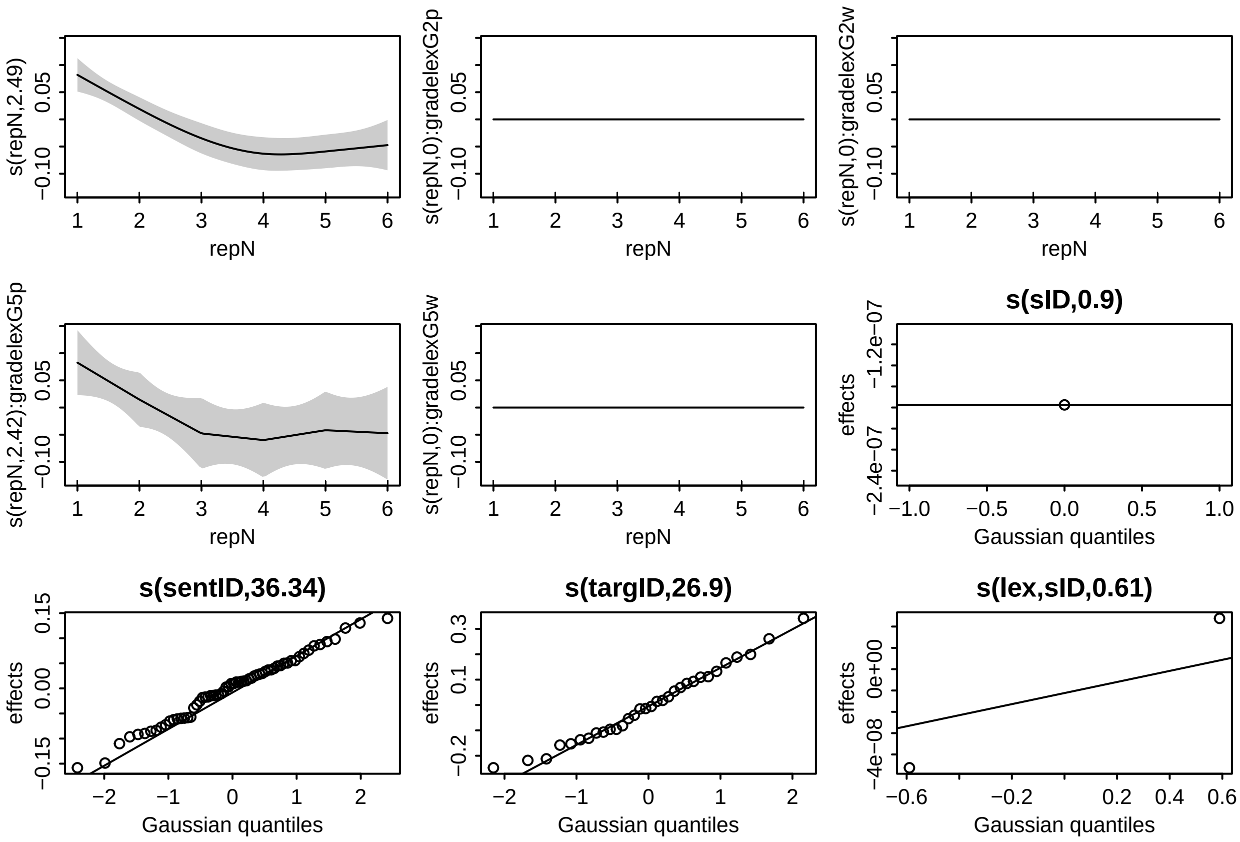


### 2.7.2 Plot of summed effects

In this plot we can see the summed effects of the fixed factors and smooths. Note that the shape of the curves is essentially identical to those from the model including fixed effects of length above. Because the interpretation is not affected and this model is simpler to present (and consistent with the models for the offline task analyses), **this is the model that is presented in the manuscript.**

```
ylm <- c(0,800)
par(mfcol=c(1,1))
par(oma=c(1.2,1.1,0,0))
par(mar=c(2.0,2.0,1.25,0.01))
par(mgp=c(2.0,0.5,0))
par(tcl= -0.25)
first <- TRUE
for (lx in c("w","p")) {
  for (gd in c("G2","G5")) {
  plot_smooth(fbm0g2,view="repN",
              cond=list(gradeC=0.5-(gd=="G2"),lexC=0.5-(lx=="w"),gradelex=paste(gd,lx,sep="")),
              rm.ranef=c("s(sID)","s(sentID)","s(targID)","s(lex,sID)"),
              se=0,#1.96,
              print.summary=F,ylim=ylm,transform=exp,
```

```
            xlab="Repetition/exposure",
            ylab="Gaze duration on target",
            lwd=3,col=ifelse(gd=="G2",col1,col2),lty=ifelse(lx=="w",1,2),cex.lab=1.25,cex.axis=1.1,
            xpd=NA,hide.label=T,yaxs="i",rug=F,
            add= first!=TRUE)
  first <- FALSE
  grid(nx=NA,ny=8,col="gray50")
  }
}
legend(4,230,c("G2 Words","G2 Pseudowords","G5 Words","G5 Pseudowords"),col=rep(c(col1,col2),each=2),lt
```

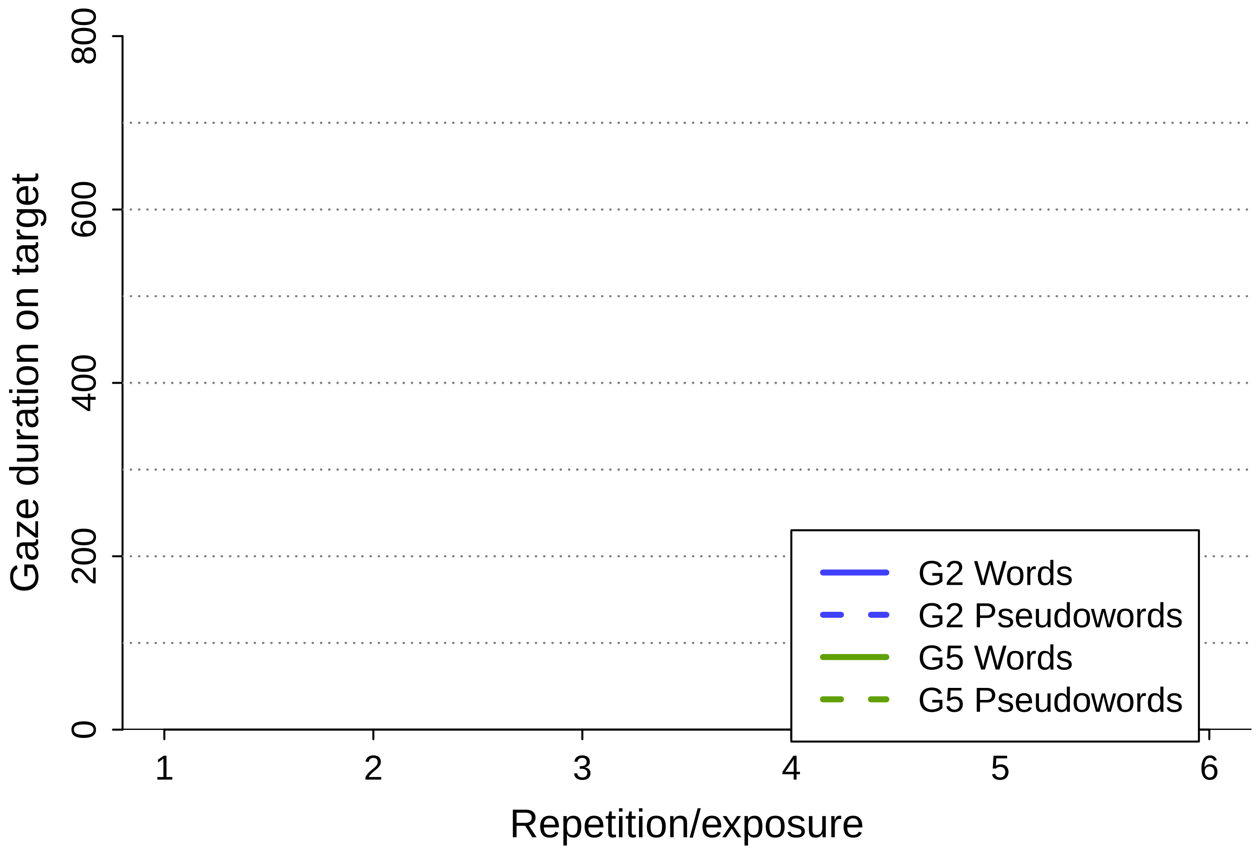


## 3 Sequential effects

Could the observed learning effects be due to participants becoming increasingly familiarized with the largely fixed sentence structure of our stimuli? We refit the model with an additional smooth of trial order, to see if that affects the learning effect.

```
fbm0gs <- bam( fDwelTl ~ 1 + gradeC + lexC + NsylC + gradeC*lexC*NsylC +
              s(repN,bs="tp",k=6) +
              s(TRIAL,bs="tp",k=8) +
              s(repN,by=gradelex,bs="tp",k=6,m=1) +
              s(sID,bs="re") + s(sentID,bs="re") + s(targID,bs="re") +
              s(Nsyl,lex,sID,bs="re",m=1),
            cluster=cl,
            data=rdat
```

```
)
summary(fbm0gs)
```

```
##
## Family: gaussian
## Link function: identity
##
## Formula:
## fDwelT1 ~ 1 + gradeC + lexC + NsylC + gradeC * lexC * NsylC +
##     s(repN, bs = "tp", k = 6) + s(TRIAL, bs = "tp", k = 8) +
##     s(repN, by = gradelex, bs = "tp", k = 6, m = 1) + s(sID,
##     bs = "re") + s(sentID, bs = "re") + s(targID, bs = "re") +
##     s(Nsyl, lex, sID, bs = "re", m = 1)
##
## Parametric coefficients:
##                   Estimate Std. Error t value Pr(>|t|)
## (Intercept)       16.49215    2.26330   7.287 3.55e-13 ***
## gradeC             5.11266    1.22115   4.187 2.87e-05 ***
## lexC               1.62931    2.28529   0.713    0.476
## NsylC              0.38542    0.86700   0.445    0.657
## gradeC:lexC        0.81431    1.23327   0.660    0.509
## gradeC:NsylC      -0.06334    0.46870  -0.135    0.893
## lexC:NsylC         1.05877    1.52534   0.694    0.488
## gradeC:lexC:NsylC  0.75714    0.82490   0.918    0.359
## ---
## Signif. codes:  0 '***' 0.001 '**' 0.01 '*' 0.05 '.' 0.1 ' ' 1
##
## Approximate significance of smooth terms:
##                         edf Ref.df      F  p-value
## s(repN)             2.441e+00  2.963 12.367  < 2e-16 ***
## s(TRIAL)            1.000e+00  1.000  5.458  0.01951 *
## s(repN):gradelexG2p 1.691e-05  5.000  0.000  0.97524
## s(repN):gradelexG2w 1.910e-05  5.000  0.000  0.92668
## s(repN):gradelexG5p 2.447e+00  5.000  1.816  0.00475 **
## s(repN):gradelexG5w 2.173e-05  5.000  0.000  0.87886
## s(sID)              9.396e-01  1.000 20.249 4.79e-05 ***
## s(sentID)           3.661e+01 63.000  1.411  < 2e-16 ***
## s(targID)           2.134e+01 28.000  3.281  < 2e-16 ***
## s(Nsyl,lex,sID)     3.142e-01  2.000  0.214  0.25832
## ---
## Signif. codes:  0 '***' 0.001 '**' 0.01 '*' 0.05 '.' 0.1 ' ' 1
##
## R-sq.(adj) =  0.169   Deviance explained = 17.8%
## fREML = 7252.9  Scale est. = 0.52523   n = 6541
```

```
compareML(fbm0g,fbm0gs)
```

```
## fbm0g: fDwelT1 ~ 1 + gradeC + lexC + NsylC + gradeC * lexC * NsylC +
##     s(repN, bs = "tp", k = 6) + s(repN, by = gradelex, bs = "tp",
##     k = 6, m = 1) + s(sID, bs = "re") + s(sentID, bs = "re") +
##     s(targID, bs = "re") + s(Nsyl, lex, sID, bs = "re", m = 1)
##
## fbm0gs: fDwelT1 ~ 1 + gradeC + lexC + NsylC + gradeC * lexC * NsylC +
##     s(repN, bs = "tp", k = 6) + s(TRIAL, bs = "tp", k = 8) +
```

```
##     s(repN, by = gradelex, bs = "tp", k = 6, m = 1) + s(sID,
##     bs = "re") + s(sentID, bs = "re") + s(targID, bs = "re") +
##     s(Nsyl, lex, sID, bs = "re", m = 1)
##
## Model fbm0g preferred: lower fREML score (0.754), and lower df (2.000).
## -----
##    Model    Score Edf Difference     Df
## 1 fbm0gs 7252.918  20
## 2  fbm0g 7252.164  18      0.754 -2.000
##
## AIC difference: 3.53, model fbm0gs has lower AIC.
```

```
## Warning in compareML(fbm0g, fbm0gs): Only small difference in fREML...
```

The difference between the two models is very small. Although trial order has a significant effect, including this does not alter the learning effect or its interaction with grade by lexicality, that is, the greater learning effect for fifth graders with pseudowords.

### 3.1 Model diagnostics and effects

```
par(mar=c(3,3,2,0.2))
par(mgp=c(1.5,0.4,0))
par(tcl= -0.2)
par(mfrow=c(2,2))
gam.check(fbm0gs)
```

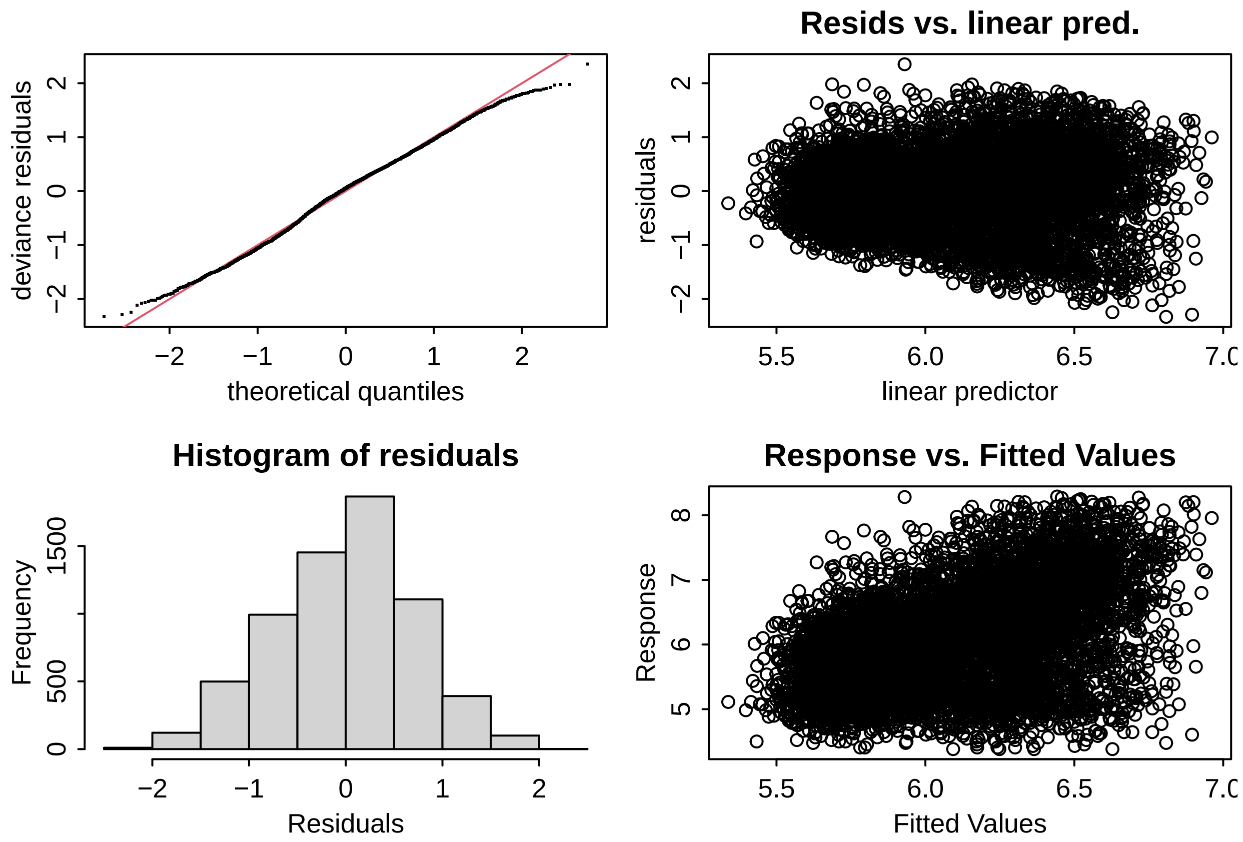


```
##
```

```
## Method: fREML   Optimizer: perf newton
## full convergence after 19 iterations.
## Gradient range [-8.262013e-06,2.020161e-07]
## (score 7252.918 & scale 0.5252258).
## Hessian positive definite, eigenvalue range [7.563377e-06,3265.639].
## Model rank =  139 / 139
##
## Basis dimension (k) checking results. Low p-value (k-index<1) may
## indicate that k is too low, especially if edf is close to k'.
##
##                            k'      edf k-index p-value
## s(repN)              5.00e+00 2.44e+00    1.01    0.68
## s(TRIAL)             7.00e+00 1.00e+00    1.02    0.96
## s(repN):gradelexG2p 5.00e+00 1.69e-05    1.01    0.70
## s(repN):gradelexG2w 5.00e+00 1.91e-05    1.01    0.68
## s(repN):gradelexG5p 5.00e+00 2.45e+00    1.01    0.70
## s(repN):gradelexG5w 5.00e+00 2.17e-05    1.01    0.69
## s(sID)               1.00e+00 9.40e-01    0.91  <2e-16 ***
## s(sentID)            6.40e+01 3.66e+01      NA      NA
## s(targID)            3.20e+01 2.13e+01      NA      NA
## s(Nsyl,lex,sID)      2.00e+00 3.14e-01      NA      NA
## ---
## Signif. codes:  0 '***' 0.001 '**' 0.01 '*' 0.05 '.' 0.1 ' ' 1
```

```r
par(mfrow=c(1,1))
```

```r
par(mar=c(3,3,2,0.2))
par(mgp=c(1.5,0.4,0))
par(tcl= -0.2)
plot.gam(fbm0gs,page=1,scheme=1)
```

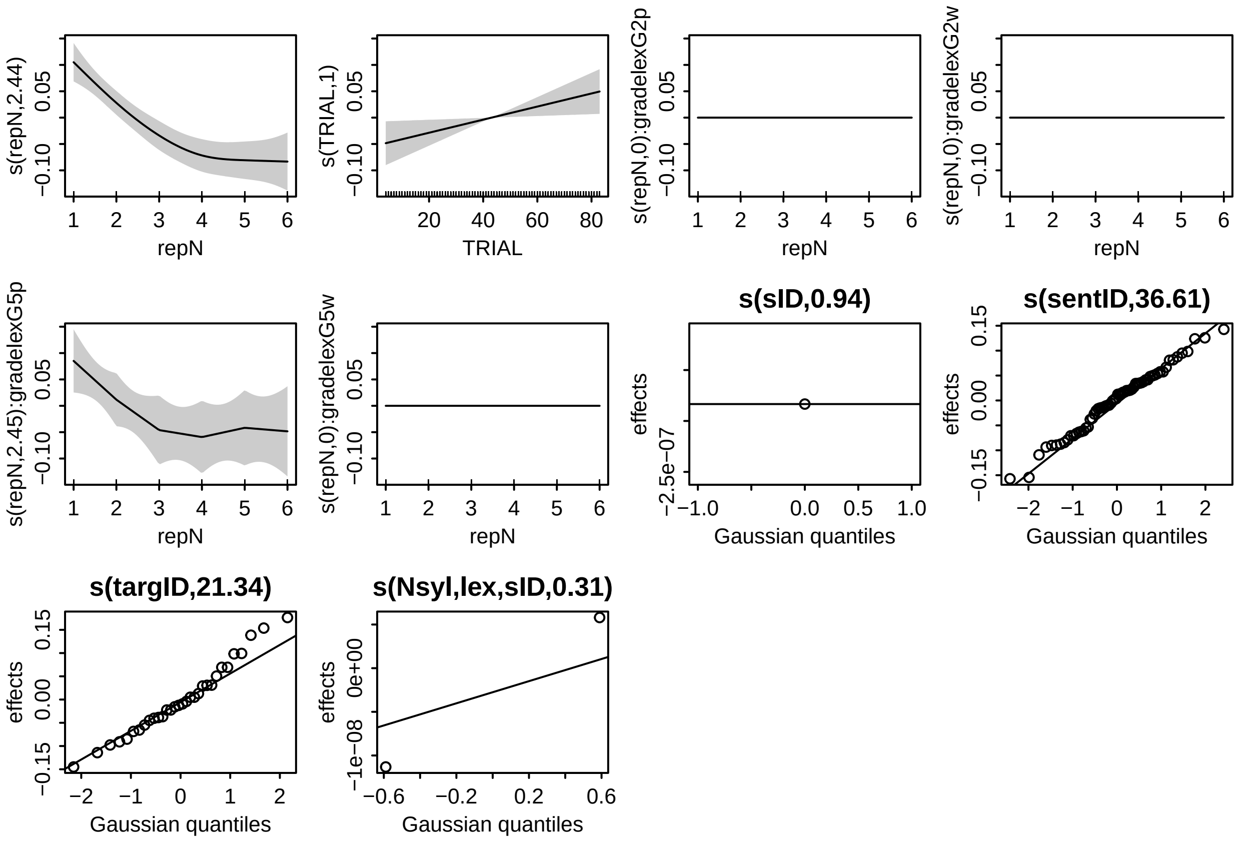


# 4 Analysis of dwell time (total)

For comparison, we also analyze total dwell time (potentially including more than first pass) in the same way.

## 4.1 Graphical display of data trends

The following graph displays mean dwell time on target for each repetition, split by grade and lexicality. Trends are more discernible here (compared to first-pass gaze duration) due to the larger learning effect.

```
meandt <- aggregate(DwelTl~sID+grade+lex+repN,rdat,mean)
par(mfrow=c(2,2))
par(oma=c(2.5,2,0,0))
par(mar=c(0.1,2,1.95,0.1))
par(mgp=c(1.25,0.25,0))
par(tcl= -0.25)
boxplot( exp(DwelTl)~repN, data=meandt, xaxt="n", main="Grade 2, words",
         ylim=c(0,2250), xlim=c(0.5,6.5), xlab="", ylab="",
         subset= grade=="G2" & lex=="w",
         boxwex=0.3, at=1:6,
         col="lightblue")
axis(1,at=1:6,labels=rep("",6))
boxplot( exp(DwelTl)~repN, data=meandt, xaxt="n", main="Grade 2, pseudowords",
         ylim=c(0,2250), xlim=c(0.5,6.5), xlab="", ylab="",
         subset= grade=="G2" & lex=="p",
         boxwex=0.3, at=1:6,
```

```
        col="lightblue")
axis(1,at=1:6,labels=rep("",6))
boxplot( exp(DwelTl)~repN, data=meandt, xaxt="n", main="Grade 5, words", xpd=NA,
        ylim=c(0,2250), xlim=c(0.5,6.5), xlab="", ylab="",
        subset= grade=="G5" & lex=="w",
        boxwex=0.3, at=1:6,
        col="lightblue")
axis(1,at=1:6)
boxplot( exp(DwelTl)~repN, data=meandt, xaxt="n", main="Grade 5, pseudowords", xpd=NA,
        ylim=c(0,2250), xlim=c(0.5,6.5), xlab="", ylab="",
        subset= grade=="G5" & lex=="p",
        boxwex=0.3, at=1:6,
        col="lightblue")
axis(1,at=1:6)
mtext("Repetition/Exposure",1,outer=T,line=1.5)
mtext("Dwell time on target (ms)",2,outer=T)
```

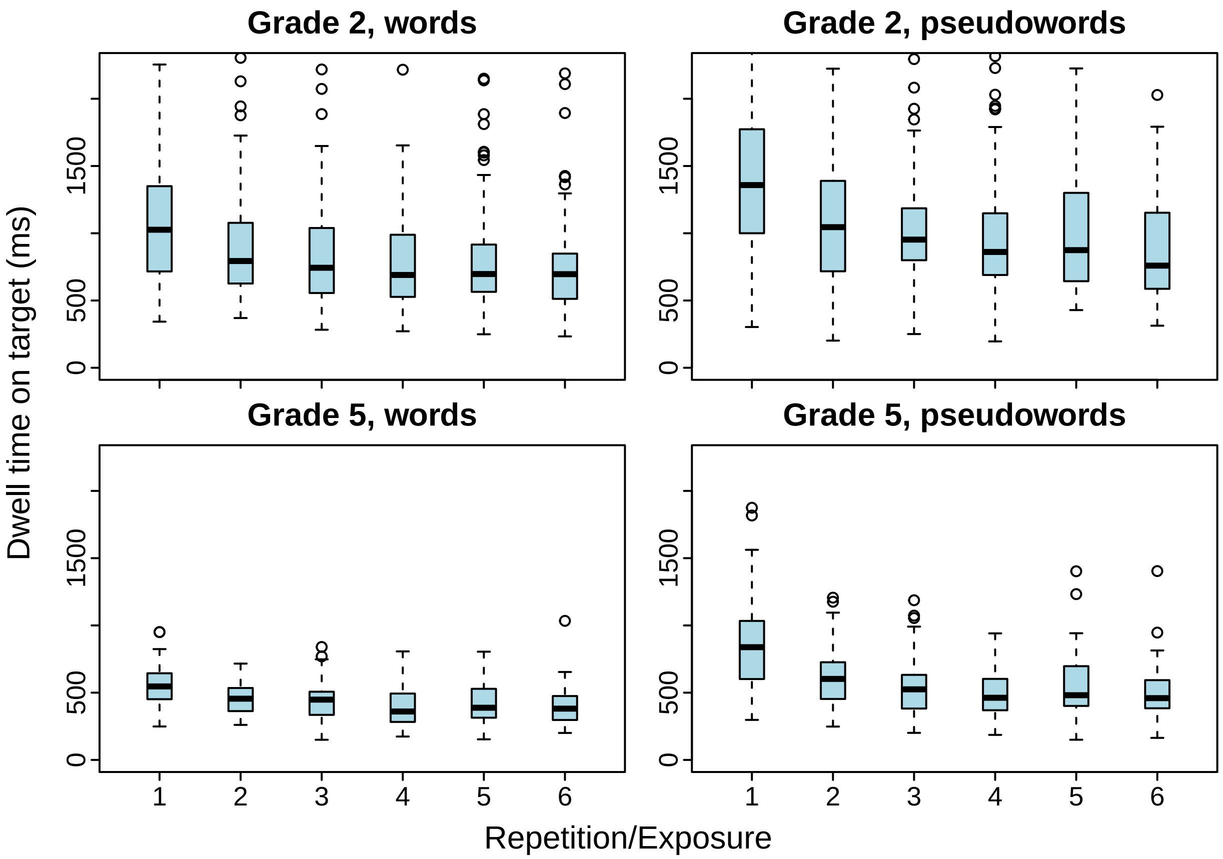


## 4.2 Full model

As with gaze duration, we first allow all three factors to interact with exposure: grade, lexicality, and length.

```
bm0d <- bam( DwelTl ~ 1 + gradeC + lexC + NsylC + gradeC*lexC*NsylC +
                s(repN,bs="tp",k=6) +
                s(repN,by=gradelexsyl,bs="tp",k=6,m=1) +
                s(sID,bs="re") + s(sentID,bs="re") + s(targID,bs="re") +
                s(Nsyl,lex,sID,bs="re",m=1),
```

```
              cluster=cl,
              data=rdat
)
summary(bm0d)
```

```
##
## Family: gaussian
## Link function: identity
##
## Formula:
## DwelT1 ~ 1 + gradeC + lexC + NsylC + gradeC * lexC * NsylC +
##     s(repN, bs = "tp", k = 6) + s(repN, by = gradelexsyl, bs = "tp",
##     k = 6, m = 1) + s(sID, bs = "re") + s(sentID, bs = "re") +
##     s(targID, bs = "re") + s(Nsyl, lex, sID, bs = "re", m = 1)
##
## Parametric coefficients:
##                     Estimate Std. Error t value Pr(>|t|)
## (Intercept)         17.58484    1.97198   8.917  < 2e-16 ***
## gradeC               5.38361    1.06395   5.060 4.31e-07 ***
## lexC                 0.27137    0.06827   3.975 7.12e-05 ***
## NsylC                0.27581    0.05955   4.632 3.69e-06 ***
## gradeC:lexC          0.02933    0.03663   0.801    0.423
## gradeC:NsylC        -0.15918    0.03189  -4.991 6.15e-07 ***
## lexC:NsylC           0.16305    0.11909   1.369    0.171
## gradeC:lexC:NsylC    0.08276    0.06379   1.297    0.195
## ---
## Signif. codes:  0 '***' 0.001 '**' 0.01 '*' 0.05 '.' 0.1 ' ' 1
##
## Approximate significance of smooth terms:
##                                edf Ref.df      F p-value
## s(repN)                  3.603e+00  4.192 60.805 < 2e-16 ***
## s(repN):gradelexsylG2p1 2.122e-05  5.000  0.000 0.77168
## s(repN):gradelexsylG2p2 1.456e+00  5.000  0.578 0.09384 .
## s(repN):gradelexsylG2w1 5.553e-01  5.000  0.153 0.21631
## s(repN):gradelexsylG2w2 2.362e-05  5.000  0.000 0.69886
## s(repN):gradelexsylG5p1 3.930e-01  5.000  0.098 0.25504
## s(repN):gradelexsylG5p2 3.138e+00  5.000  2.642 0.00131 **
## s(repN):gradelexsylG5w1 1.885e-05  5.000  0.000 0.82794
## s(repN):gradelexsylG5w2 1.745e-05  5.000  0.000 0.88277
## s(sID)                  9.692e-01  1.000 31.581 < 2e-16 ***
## s(sentID)               1.909e+01 63.000  0.440 0.01215 *
## s(targID)               2.594e+01 28.000 13.269 < 2e-16 ***
## s(Nsyl,lex,sID)         9.657e-05  2.000  0.000 0.55999
## ---
## Signif. codes:  0 '***' 0.001 '**' 0.01 '*' 0.05 '.' 0.1 ' ' 1
##
## R-sq.(adj) =  0.305   Deviance explained = 31.1%
## fREML = 6461.9  Scale est. = 0.39975   n = 6644
```

## 4.3 Excluding length

We then remove the interaction of length with exposure and compare the two models.

```r
bm0g <- bam( DwelT1 ~ 1 + gradeC + lexC + NsylC + gradeC*lexC*NsylC +
                s(repN,bs="tp",k=6) +
                s(repN,by=gradelex,bs="tp",k=6,m=1) + # removed syl
                s(sID,bs="re") + s(sentID,bs="re") + s(targID,bs="re") +
                s(Nsyl,lex,sID,bs="re",m=1),
              cluster=cl,
              data=rdat
)
summary(bm0g)
```

```
##
## Family: gaussian
## Link function: identity
##
## Formula:
## DwelT1 ~ 1 + gradeC + lexC + NsylC + gradeC * lexC * NsylC +
##     s(repN, bs = "tp", k = 6) + s(repN, by = gradelex, bs = "tp",
##     k = 6, m = 1) + s(sID, bs = "re") + s(sentID, bs = "re") +
##     s(targID, bs = "re") + s(Nsyl, lex, sID, bs = "re", m = 1)
##
## Parametric coefficients:
##                     Estimate Std. Error t value Pr(>|t|)
## (Intercept)         17.58801    1.97291   8.915  < 2e-16 ***
## gradeC               5.38503    1.06445   5.059 4.33e-07 ***
## lexC                 0.27109    0.07193   3.769 0.000165 ***
## NsylC                0.27607    0.06002   4.600 4.31e-06 ***
## gradeC:lexC          0.02847    0.03863   0.737 0.461082
## gradeC:NsylC        -0.15936    0.03217  -4.955 7.43e-07 ***
## lexC:NsylC           0.16318    0.12003   1.360 0.174030
## gradeC:lexC:NsylC    0.08239    0.06434   1.281 0.200388
## ---
## Signif. codes:  0 '***' 0.001 '**' 0.01 '*' 0.05 '.' 0.1 ' ' 1
##
## Approximate significance of smooth terms:
##                            edf Ref.df      F p-value
## s(repN)              3.651e+00  4.238 65.046  <2e-16 ***
## s(repN):gradelexG2p 5.375e-05  5.000  0.000  0.5048
## s(repN):gradelexG2w 3.448e-05  5.000  0.000  0.6801
## s(repN):gradelexG5p 2.601e+00  5.000  1.945  0.0043 **
## s(repN):gradelexG5w 2.959e-05  5.000  0.000  0.7620
## s(sID)               9.692e-01  1.000 31.570  <2e-16 ***
## s(sentID)            1.924e+01 63.000  0.444  0.0115 *
## s(targID)            2.593e+01 28.000 13.249  <2e-16 ***
## s(Nsyl,lex,sID)      1.359e-04  2.000  0.000  0.5634
## ---
## Signif. codes:  0 '***' 0.001 '**' 0.01 '*' 0.05 '.' 0.1 ' ' 1
##
## R-sq.(adj) =  0.304   Deviance explained =   31%
## fREML = 6463.2  Scale est. = 0.40016   n = 6644
```

```r
compareML(bm0g,bm0d)
```

```
## bm0g: DwelT1 ~ 1 + gradeC + lexC + NsylC + gradeC * lexC * NsylC +
##     s(repN, bs = "tp", k = 6) + s(repN, by = gradelex, bs = "tp",
```

```
##     k = 6, m = 1) + s(sID, bs = "re") + s(sentID, bs = "re") +
##     s(targID, bs = "re") + s(Nsyl, lex, sID, bs = "re", m = 1)
##
## bm0d: DwelTl ~ 1 + gradeC + lexC + NsylC + gradeC * lexC * NsylC +
##     s(repN, bs = "tp", k = 6) + s(repN, by = gradelexsyl, bs = "tp",
##     k = 6, m = 1) + s(sID, bs = "re") + s(sentID, bs = "re") +
##     s(targID, bs = "re") + s(Nsyl, lex, sID, bs = "re", m = 1)

## Warning in sprintf(" ", h1): one argument not used by format ' '

##
## Chi-square test of fREML scores
## -----
##   Model    Score Edf Difference    Df p.value Sig.
## 1  bm0g 6463.210  18
## 2  bm0d 6461.941  22      1.268 4.000   0.638
##
## AIC difference: -3.28, model bm0g has lower AIC.

## Warning in compareML(bm0g, bm0d): Only small difference in fREML...
```

Again, there is no significant difference. The pattern of significant terms is exactly the same as for gaze duration.

## 4.4 Model diagnostics and effects

```
par(mar=c(3,3,2,0.2))
par(mgp=c(1.5,0.4,0))
par(tcl= -0.2)
par(mfrow=c(2,2))
gam.check(bm0g)
```

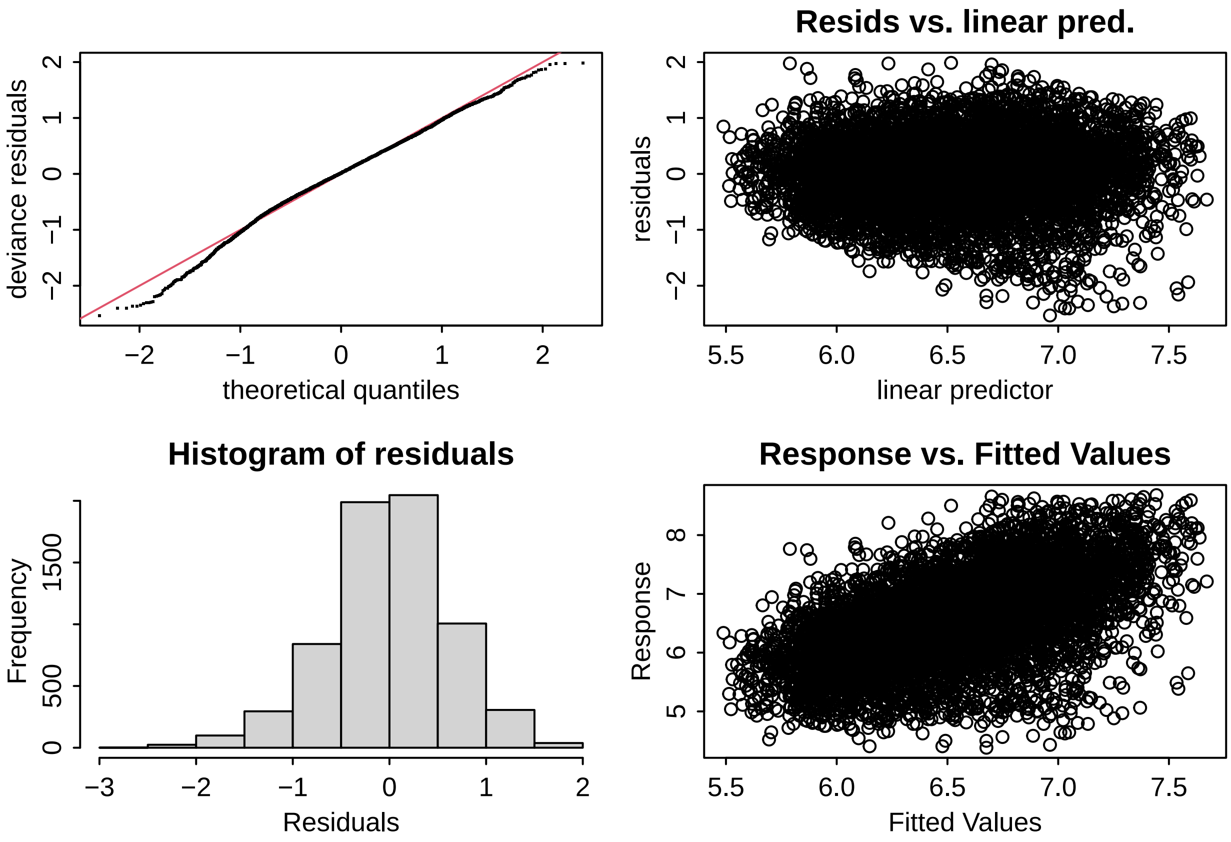


```
##
## Method: fREML   Optimizer: perf newton
## full convergence after 22 iterations.
## Gradient range [-3.848021e-05,2.9472e-05]
## (score 6463.21 & scale 0.4001587).
## Hessian positive definite, eigenvalue range [8.791695e-06,3317.58].
## Model rank =  132 / 132
##
## Basis dimension (k) checking results. Low p-value (k-index<1) may
## indicate that k is too low, especially if edf is close to k'.
##
##                             k'      edf k-index p-value
## s(repN)               5.00e+00 3.65e+00    0.99    0.32
## s(repN):gradelexG2p 5.00e+00 5.37e-05    0.99    0.34
## s(repN):gradelexG2w 5.00e+00 3.45e-05    0.99    0.31
## s(repN):gradelexG5p 5.00e+00 2.60e+00    0.99    0.24
## s(repN):gradelexG5w 5.00e+00 2.96e-05    0.99    0.27
## s(sID)                1.00e+00 9.69e-01    0.83  <2e-16 ***
## s(sentID)             6.40e+01 1.92e+01      NA      NA
## s(targID)             3.20e+01 2.59e+01      NA      NA
## s(Nsyl,lex,sID)       2.00e+00 1.36e-04      NA      NA
## ---
## Signif. codes:  0 '***' 0.001 '**' 0.01 '*' 0.05 '.' 0.1 ' ' 1
```

```
par(mfrow=c(1,1))
```

```
par(mar=c(3,3,2,0.2))
par(mgp=c(1.5,0.4,0))
par(tcl= -0.2)
plot.gam(bm0g,page=1,scheme=1)
```

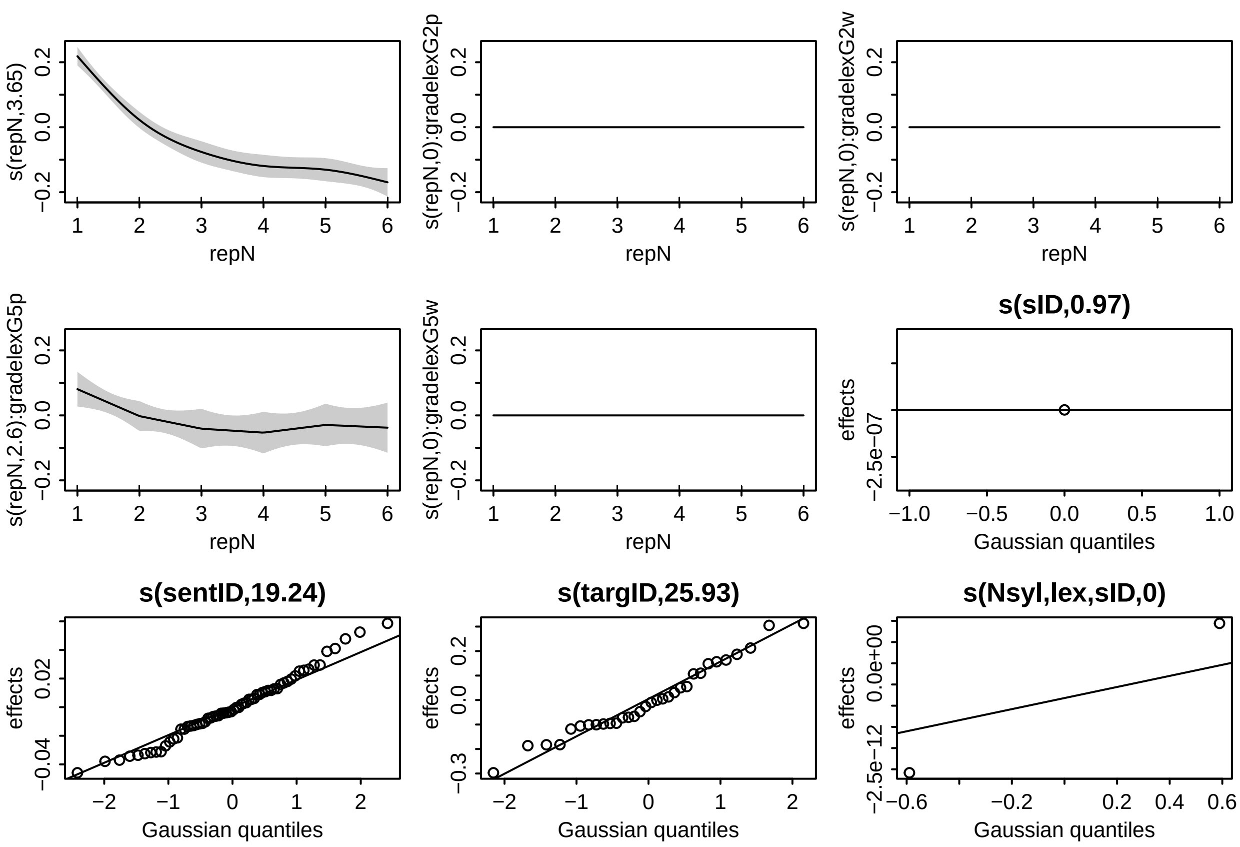


## 4.5 Plot of summed effects

```
ylm <- c(0,1500)
par(mfcol=c(1,1))
par(oma=c(1.2,1.1,0,0))
par(mar=c(2.0,2.0,1.25,0.01))
par(mgp=c(2.0,0.5,0))
par(tcl= -0.25)
first <- TRUE
for (lx in c("w","p")) {
  for (gd in c("G2","G5")) {
  plot_smooth(bm0g,view="repN",
              cond=list(gradeC=0.5-(gd=="G2"),lexC=0.5-(lx=="w"),NsylC=0,gradelex=paste(gd,lx,sep="")),
              rm.ranef=c("s(sID)","s(sentID)","s(targID)","s(Nsyl,lex,sID)"),
              se=0,#1.96,
              print.summary=F,ylim=ylm,transform=exp,
              xlab="Repetition/exposure",
              ylab="Dwell time on target",
              lwd=3,col=ifelse(gd=="G2",col1,col2),lty=ifelse(lx=="w",1,2),cex.lab=1.25,cex.axis=1.1,
              xpd=NA,hide.label=T,yaxs="i",rug=F,
```

```
                add= first!=TRUE)
  first <- FALSE
  grid(nx=NA,ny=15,col="gray50")
  }
}
legend(4,1450,c("G2 Words","G2 Pseudowords","G5 Words","G5 Pseudowords"),col=rep(c(col1,col2),each=2),l
```

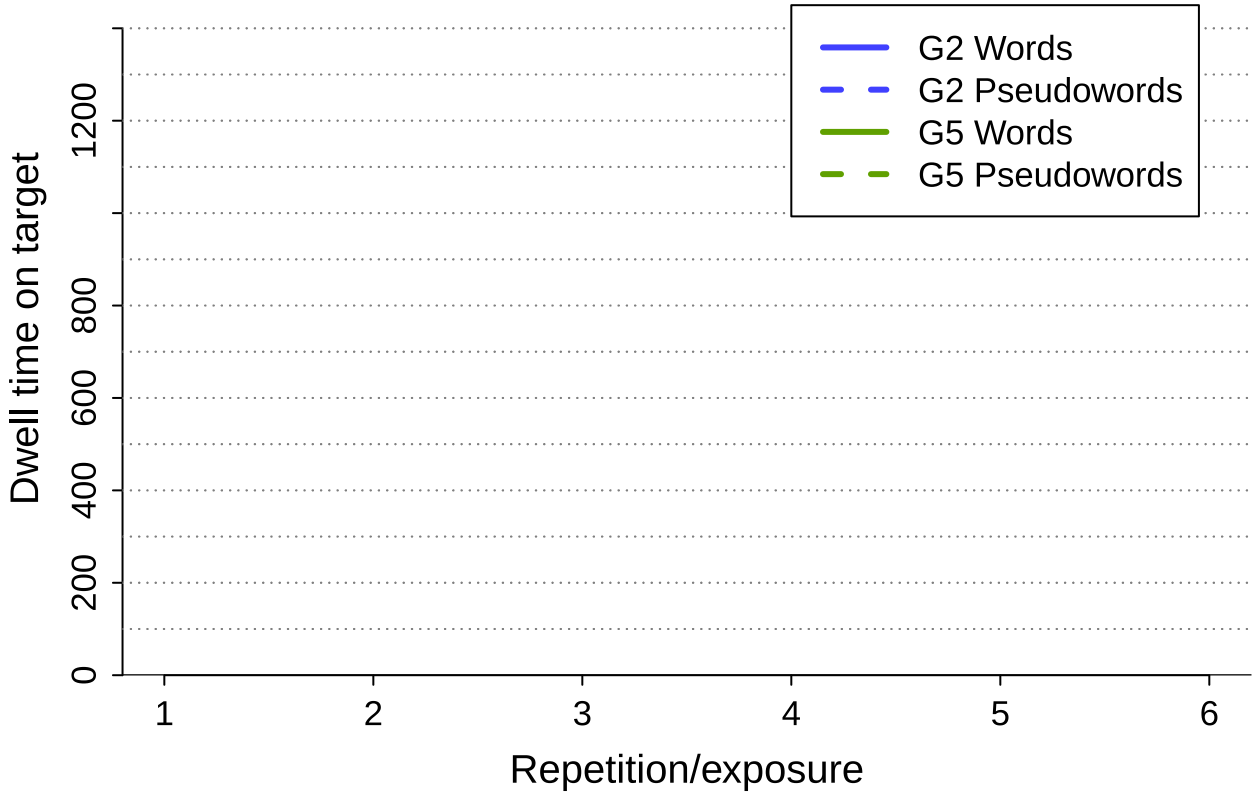


The pattern of results is identical to that with (first-pass) gaze duration, except the effects are larger (and of course durations are longer).

## 5 Session information

```
sessionInfo()
```

```
## R version 4.1.0 (2021-05-18)
## Platform: x86_64-w64-mingw32/x64 (64-bit)
## Running under: Windows 10 x64 (build 19042)
##
## Matrix products: default
##
## locale:
## [1] LC_COLLATE=English_United States.1252
## [2] LC_CTYPE=English_United States.1252
## [3] LC_MONETARY=English_United States.1252
## [4] LC_NUMERIC=C
```

```
## [5] LC_TIME=English_United States.1252
##
## attached base packages:
## [1] parallel  stats     graphics  grDevices utils     datasets  methods
## [8] base
##
## other attached packages:
## [1] car_3.0-10        carData_3.0-4     itsadug_2.4       plotfunctions_1.4
## [5] mgcv_1.8-35       nlme_3.1-152
##
## loaded via a namespace (and not attached):
##  [1] zip_2.2.0         Rcpp_1.0.6        cellranger_1.1.0  compiler_4.1.0
##  [5] pillar_1.6.1      forcats_0.5.1     tools_4.1.0       digest_0.6.27
##  [9] evaluate_0.14     lifecycle_1.0.0   tibble_3.1.2      lattice_0.20-44
## [13] pkgconfig_2.0.3   rlang_0.4.11      openxlsx_4.2.3    Matrix_1.3-3
## [17] curl_4.3.1        yaml_2.2.1        haven_2.4.1       xfun_0.23
## [21] rio_0.5.26        stringr_1.4.0     knitr_1.33        vctrs_0.3.8
## [25] hms_1.1.0         grid_4.1.0        data.table_1.14.0 fansi_0.5.0
## [29] readxl_1.3.1      foreign_0.8-81    rmarkdown_2.8     magrittr_2.0.1
## [33] htmltools_0.5.1.1 ellipsis_0.3.2    splines_4.1.0     abind_1.4-5
## [37] utf8_1.2.1        stringi_1.6.1     crayon_1.4.1
```
